# Genome-wide global identification of NRF2 binding sites in A549 non-small cell lung cancer cells by ChIP-Seq reveals NRF2 regulation of genes involved in focal adhesion pathways

**DOI:** 10.18632/aging.102590

**Published:** 2019-12-28

**Authors:** Akhileshwar Namani, Kaihua Liu, Shengcun Wang, Xihang Zhou, Yijiao Liao, Hongyan Wang, Xiu Jun Wang, Xiuwen Tang

**Affiliations:** 1 Department of Biochemistry and Department of Thoracic Surgery of the First Affiliated Hospital, Zhejiang University School of Medicine, Zhejiang University, Hangzhou 310003, PR China; 2 Department of Pharmacology and Cancer Institute of the Second Affiliated Hospital, Zhejiang University School of Medicine, Zhejiang University, Hangzhou 310058, PR China

**Keywords:** nuclear factor erythroid-derived-2-like 2, non-small-cell lung cancer, ChIP-seq, microarray, focal adhesion

## Abstract

Nuclear factor erythroid-derived-2-like 2(NRF2) regulates its downstream genes through binding with antioxidant responsive elements in their promoter regions. Hyperactivation of NRF2 results in oncogenesis and drug resistance in various cancers including non-small cell lung cancer (NSCLC). However, identification of the genes and pathways regulated by NRF2 in NSCLC warrants further investigation. We investigated the global NRF2 genomic binding sites using the high-throughput ChIP-Seq technique in KEAP1 (Kelch-like ECH-associated protein 1)-mutated A549 (NSCLC) cells. We next carried out an integrated analysis of the ChIP-Seq data with transcriptomic data from A549 cells with NRF2-knockdown and RNA-Seq data from TCGA patients with altered KEAP1 to identify downstream and clinically-correlated genes respectively. Furthermore, we applied transcription factor enrichment analysis, generated a protein-protein interaction network, and used kinase enrichment analysis. Moreover, functional annotation of NRF2 binding sites using DAVID v7 identified the genes involved in focal adhesion. Putative focal adhesion genes regulated by NRF2 were validated using qRT-PCR. Further, we selected one novel conserved focal adhesion gene regulated by NRF2–LAMC1 (laminin subunit gamma 1) and validated it using a reporter assay. Overall, the identification of NRF2 target genes paves the way for identifying the molecular mechanism of NRF2 signaling in NSCLC development and therapy. Moreover, our data highlight the complexity of the pathways regulated by NRF2 in lung tumorigenesis.

## INTRODUCTION

Lung cancer is the leading cause of cancer mortality in men and women around the world, and ~85% of all lung cancer cases are non-small cell lung carcinoma (NSCLC) [1]. Despite the advances in surgical and chemo/radiation therapies, the prognosis and overall survival remain very poor [2].

The transcription factor NFE2L2 (nuclear factor erythroid-derived-2-like 2), also known as NRF2, is a master regulator of the antioxidant response pathway. Under homeostatic conditions, NRF2 binds to its inhibitor KEAP1 (Kelch-like ECH-associated protein-1). KEAP1 acts as a scaffolding protein for NRF2 and Cullin-3 E3 ubiquitin ligase (CUL3), which promotes the ubiquitin-mediated proteasomal degradation of NRF2 [3]. Under oxidative stress, NRF2 dissociates from the KEAP1 repression, translocates into the nucleus and heterodimerizes with small Maf protein. This complex transactivates the downstream antioxidant responsive element (ARE) gene battery to maintain cellular homeostasis [4]. Due to its role in cellular defense and cytoprotection in cancer, NRF2 has become a major therapeutic target for cancer chemoprevention and a wide range of novel natural and synthetic inducers have been identified, some of which are currently undergoing clinical trials [5, 6]. On the other hand, somatic mutations of the KEAP1 and NRF2 genes and KEAP1 hypermethylation in lung and other cancers result in the constitutive activation of NRF2 and the increased expression of cytoprotective genes which promote cancer cell survival, tumor promotion, and resistance against chemotherapy or radiotherapy [7–9]. Growing evidences suggest that NRF2 regulates key genes in metabolic pathways such as glycolysis, the pentose phosphate pathway, the tricarboxylic acid cycle, and fatty acid synthesis [10, 11].

Chromatin immunoprecipitation followed by next-generation sequencing is an effective technique for the identification of specific transcription factor binding sites (TFBSs) [12]. Previous reports of NRF2 binding at the genome-wide level in mouse embryonic fibroblasts [13], mouse hepatoma cells [14] and human lymphoblastoid cell lines [15] have revealed several known and putative genes and pathways regulated by NRF2. However, little is known about how the binding patterns of NRF2 differ between normal and cancer cells, specifically in KEAP1-mutated A549 (NSCLC) cells. Since NRF2 is overexpressed due to a KEAP1 loss-of-function mutation in human A549 cells, we used this cell line to identify the NRF2 binding pattern and its transcriptional activity. This analysis aimed to elucidate the relationships between NRF2 and disease progression and provide insight into NRF2-mediated cancer progression/tumorigenesis by identifying novel genes and pathways regulated by NRF2. Besides, we carried out an integrated analysis of our ChIP-Seq and publicly-available transcriptomics data from NRF2 knockdown A549 cells, and from patients with TCGA (The Cancer Genome Atlas) lung adenocarcinoma (LUAD) to identify the transcriptional network of NRF2-regulated genes and pathways. Our ChIP-Seq results revealed a direct association of NRF2 with a set of genes involved in novel pathways such as focal adhesion in lung cancer cells.

## RESULTS

### Genomic occupancy of NRF2 binding sites in A549 cells

To examine the genome-wide distribution of NRF2 binding sites in lung cancer cells, we performed ChIP-Seq of human A549 NSCLC cells. The ChIPed DNA was sequenced and mapped using an Illumina sequencer. The mapped reads were aligned using Bowtie version 0.12.7-1. From this, we identified a total of 8,619,855 aligned tags for control DNA (Supplementary Figure 1A), and 1,494,203 for input DNA (Supplementary Figure 1B and Table 1).

**Table 1:** Genome alignment distribution of our ChIP-Seq data.

**DNA**	**Chromosome length**	**Sense reads**	**Antisense reads**	**Total aligned reads**
Control	3095693983	4307103	4312752	8619855
Input	3095693983	746866	747337	1494203

We then used HOMER software [16] to perform the peak calling step to detect the ChIP-Seq peaks. After HOMER analysis, we found 23,257 tags in peaks with ~1.56% IP efficiency. Then we identified 2,395 strongly-enriched genome-wide NRF2 binding sites with 150-bp peak size using tags on both strands (Supplementary Table 1). The minimum distance maintained between peaks was 300 bp.

**Figure 1:**
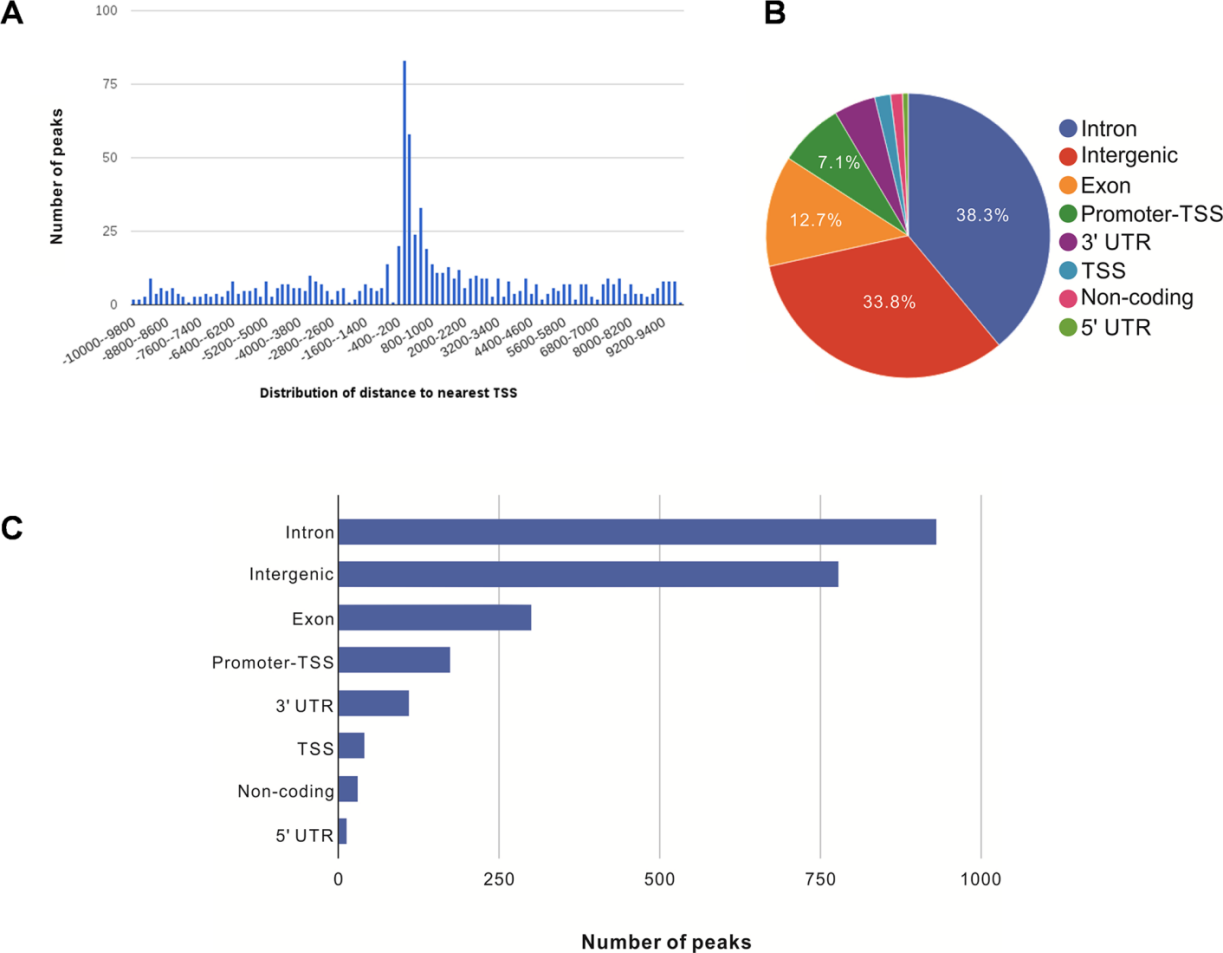
**Genome-wide annotation of NRF2-binding sites and response elements in A549 NSCLC cells.** (**A**) Distribution of NRF2 transcription factor binding sites relative to the nearest TSS across the human genome (x-axis, number of peaks in the genome; y-axis, distance relative to the TSS from –10 KB to +10 KB). (**B**) Pie chart of the percentages of NRF2-binding sites according to peak location across different genomic regions of the human genome. (**C**) Bar chart of the numbers of NRF2-binding sites categorized according to peak location across the human genome.

We further annotated the genomic locations of 2,395 NRF2 binding sites using the ChIPSeek web tool [17]. The annotated genes showed a wide distribution pattern in which 782 binding sites were located within 10 kb of a transcription start site (TSS) (Figure 1A). In total, 170 sites (7.1%) were located proximal to the TSS region. 917 (38.3%) were in introns, 809 (33.8%) were intergenic, 303 (12.7%) were in exons, 15 (0.6%) were in 5' untranslated regions (UTRs), 39 were in TSSs (1.6%), and 113 (4.7%) were in 3' UTRs. The remaining 29 binding sites (1.2%) were located in non-coding regions (Figure 1B, 1C). These results showed that the majority of NRF2 binding sites are located in introns, followed by intergenic regions, exons, promoter-TSSs and to a lesser degree in 3' UTRs, TSSs, non-coding regions, and 5' UTRs. Our genomic annotation of NRF2 transcription factor binding sites (TFBSs) showed that NRF2 also bound at the introns and intergenic regions.

### Known and *de novo* motif analysis of NRF2 binding sites

**Figure 2:**
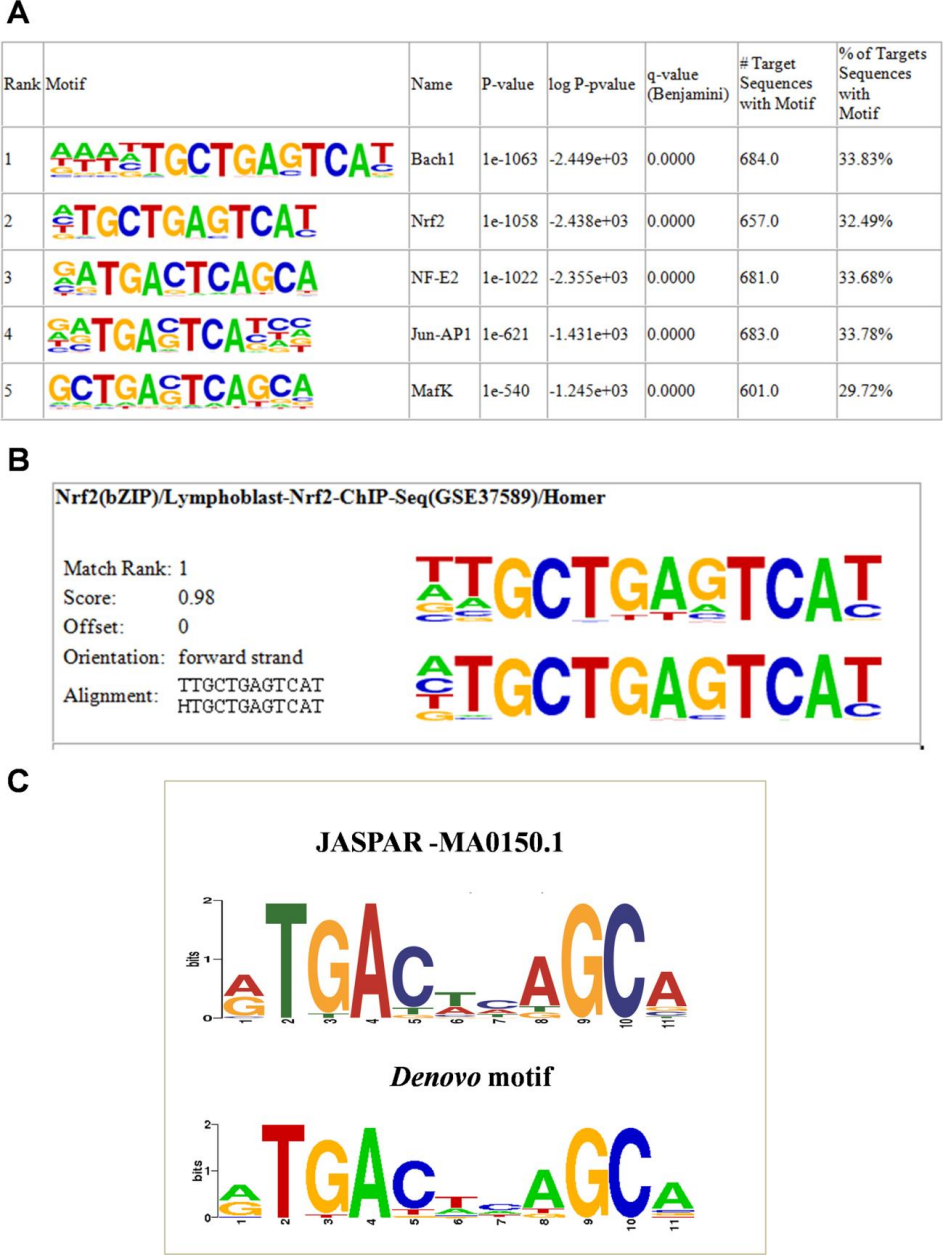
**NRF2 TFBS motif enrichment analysis.** (**A**) Enrichment of known motifs (target motifs *vs.* background known motifs) showing the top-ranked motif logos. (**B**) Logo showing the top ranked *de novo* motif identified using HOMER. (**C**) STAMP analysis results showing the logo of the *de novo* motif identified by HOMER (lower) highly matched the NFE2L2-JASPAR binding motif (upper).

To determine whether the human NRF2 binding regions in A549 cells have their unique ARE, we used the HOMER known and *de novo* motif discovery algorithm. Motifs were sorted based on p-values. As expected, the enrichment results for known motifs were strongest for the bZIP family TFBSs (Figure 2A). The results consisted of motifs derived from previously-published ChIP-Seq experiments on Bach1, NRF2, NF-E2, Jun-AP1, and MafK, among others. (Supplementary Table 2). Interestingly, the results for *de novo* motifs showed that 34.47% (697/2,395) of the target sequences contained the 12-bp consensus NRF2 ARE (ATGACTCAGCAA) among all TFBSs, with a p-value of 1e-1057 (Figure 2B). We then compared the *de novo* motif with the original ARE motif using the motif comparison tool STAMP [18]. The HOMER query motif (matrix) against databases of known motifs (JASPAR) in STAMP analysis ranked the NRF2 TFBS as number 1 and it showed greatest similarity with the consensus NRF2 ARE sequence (TGACNNNGC) [19–21] with a significant E value cutoff (0.0000e+00) (Figure 2C). Thus, *de novo* motif analysis strongly suggested that NRF2 specifically binds to its target DNA through a well-accepted ARE sequence and transactivates its downstream genes.

### TFBS overrepresentation of NRF2-binding sites

We then investigated the overrepresentation of NRF2 binding sites among TFBSs using the web tool TRAP (transcription factor affinity prediction) [22]. TRAP analysis identified NRF2 and other TFBSs (Table 2). This result is consistent with previous reports on NRF2 and activator protein–1 (AP-1) binding sites where both transcription factors overlap with their binding sites [23]. Of important note, other TFBSs (Pax2, FOXA1, Foxa2, SOX10, FOXD1, Sox17, HNF1B, and CEBPA) included the NRF2 TFBS, indicating the possibility of NRF2 interaction with these proteins. We are performing further experiments to test our hypothesis.

**Table 2:** TFBS over-representation in the NRF2 ChIP-Seq binding profiles using TRAP analysis

**#/ Rank**	**Combined_P**	**Corrected_P**	**Matrix_ID**	**Matrix_name**
1	0	0	MA0150.1	NFE2L2
2	0	0	MA0099.2	AP1
3	1.07E-218	4.20E-217	MA0067.1	Pax2
4	3.79E-55	1.12E-53	MA0148.1	FOXA1
5	1.49E-50	3.52E-49	MA0047.2	Foxa2
6	1.38E-41	2.71E-40	MA0442.1	SOX10
7	1.73E-33	2.92E-32	MA0031.1	FOXD1
8	2.46E-29	3.62E-28	MA0078.1	Sox17
9	6.72E-28	8.81E-27	MA0153.1	HNF1B
10	1.56E-27	1.68E-26	MA0102.2	CEBPA

### Overview of the binding pattern of known NRF2 target genes in A549 NSCLC cells

**Figure 3:**
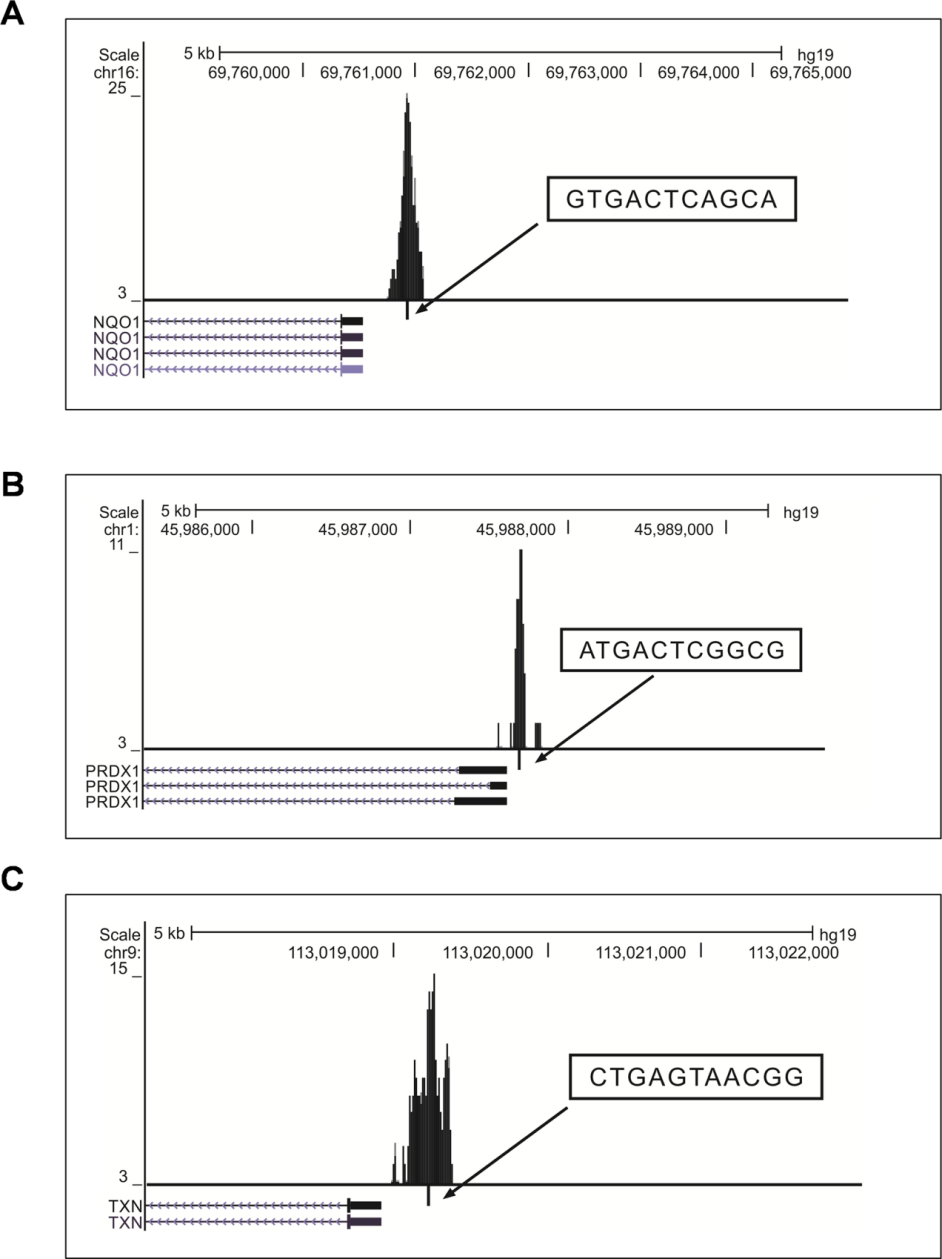
**Visualization of NRF2 binding sites obtained from the UCSC genome browser (version hg19).** (**A**–**C**) Locations of AREs in the promoter regions of the known NRF2 target genes NQO1 (**A**), PRDX1 (**B**), and TXN (**C**). The peaks represent the 150-bp binding regions identified from our ChIP-Seq results (boxes ARE sequences; ticks, ARE positions; blocks, coding exons; horizontal lines with arrows connecting exons represent introns).

To determine the binding pattern of the previously-known classic NRF2 target genes listed in review articles [24–27], we shortlisted genes that bound at the promoter TSS region of the NRF2 TFBS (Supplementary Table 3). We found well-known NRF2-regulated genes [NAD(P)H dehydrogenase, quinone 1 (NQO1), glutamate-cysteine ligase, modifier subunit (GCLM), thioredoxin (TXN), ferrochelatase (FECH), peroxiredoxin 1 (PRDX1), aldo-keto reductase family 1, member B10 (aldose reductase), glutathione reductase (GSR), and glutathione peroxidase 2 (gastrointestinal) (GPX2)] that bound to the TSS promoter region (Figure 3). However heme oxygenase (decycling) 1 (HMOX1) was not bound to the TSS promoter region, but the binding sites were present in the intergenic and exon regions in this cell line. We next determined whether the binding pattern of the known genes was similar to the previously-reported regulatory regions of human promoters. We found the exact binding pattern for GCLM, GPX2, MAFG, and SRXN1 with the same AREs (see Table 3), while NQO1, PRDX1, and TXN showed differential binding patterns in their promoter regions.

**Table 3:** Known human NRF2 ARE genes and their binding patterns in the promoter regions of our TFBS data.

**Gene symbol**	ARE sequence*	**ChIP-Seq binding site**	**Reference**
GCLM	AGACAATGACTAAGCAGAAAT	Overlapping	[25]
GPX2	CCAGGATGACTTAGCAAAAAC	Overlapping	[26]
MAFG	TCACGCTGACTCAGCACATTG	Overlapping	[25]
SRXN1	CCAGGGTGAGTCGGCAAAGCC	Overlapping	[27]
NQO1	TTCTGCTGAGTCACTGTGACT	No overlap	[27]
PRDX1	CCGGAATGACTCGGCGCTTTC	No overlap	[25]
TXN	AAGTGCTGAGTAACGGTGACC	No overlap	[27]

*11 bp ARE highlighted in bold

### NRF2–TFBS network analysis using the X2K database

Our ChIP-Seq data identified 2,051 coding and non-coding regions that represented 2,395 binding sites. We then identified the cell-signaling networks associated with these genes. To decode the networks associated with the NRF2 TFBS, we used the computational tool eXpression2Kinases (X2K). Notably, the X2K transcription factor enrichment analysis accurately revealed NRF2 as the top predicted transcription factor regulating the TFBS-encoded genes (Supplementary Figure 2A). The protein-protein interaction expansion analysis using the Genes2Networks algorithm identified the sub-network of connected transcription factors that regulated the TFBS-encoded genes (Supplementary Figure 2B). Finally, kinase enrichment analysis showed that the main kinases driving the expression of TFBS-encoded genes included the known NRF2-regulated kinases MAPKs, ERK, and GSK3B (Supplementary Figure 2C, 2D).

### Integrated ChIP-Seq and microarray data analysis of NRF2 target genes revealed the role of NRF2 in metabolism

To achieve a more effective TFBS analysis of the functions and gene-regulatory mechanisms of NRF2, we analyzed our NRF2 ChIP-Seq data in combination with GSE28230 microarray data [10]. Besides, this analysis allowed for a better understanding of the mechanisms that determine the role of NRF2 in carcinogenesis. We carried out the integrated analysis of 2,395 ChIP-Seq TFBSs and 1,629 down-regulated genes from microarray data using Venny [28] and obtained 253 overlapping genes (Supplementary Figure 3). The complete list of overlapping genes can be found in Supplementary Table 4. We then performed KEGG pathway analysis of the overlapping genes using the DAVID v7 [29] web server to identify novel pathways regulated by NRF2. Among the top biological pathways identified with p<0.05, porphyrin and chlorophyll metabolism remained the most significant associated pathways, followed by metabolism of xenobiotics by cytochrome P450, pentose phosphate pathway, glutathione metabolism, fructose and mannose metabolism, arachidonic acid metabolism, glycolysis/gluconeogenesis, pyruvate metabolism, ascorbate and aldarate metabolism, pentose and glucuronate interconversions, and steroid hormone biosynthesis. Interestingly, among the 13 pathways identified in KEGG analysis, 12 were associated with metabolism, indicating crucial roles of NRF2 in the metabolic pathways of lung cancer cells (Supplementary Table 5). Besides, known NRF2 target genes such as GPX2, GSR, GCLC, GCLM, FECH, HMOX1, and AKR1C3 were found in different KEGG pathways. Thus, integrated analysis of ChIP-Seq and gene expression microarray data in lung cancer cells revealed a strong correlation of NRF2-regulated genes involved in carbon metabolism and further confirmed the reproducibility of our results and those of others.

### Clinical correlation of NRF2 TFBS genes in TCGA LUAD patients

To investigate clinical correlations of the NRF2 TFBS with genes affected by genetic KEAP1 alterations (mutation and deletion) in LUAD patients, we carried out an integrated analysis of the two sets of genes using Venny [28]. This combinatorial analysis showed that 26 NRF2 target genes (fold-change > 1.5) were significantly overexpressed in the data from patients with alterations compared to the wild-type. This hyperactivation occurred through the upregulation of NRF2 signaling in samples with altered KEAP1 (Supplementary Table 6).

### Novel signaling pathways associated with NRF2-bound target genes

**Figure 4:**
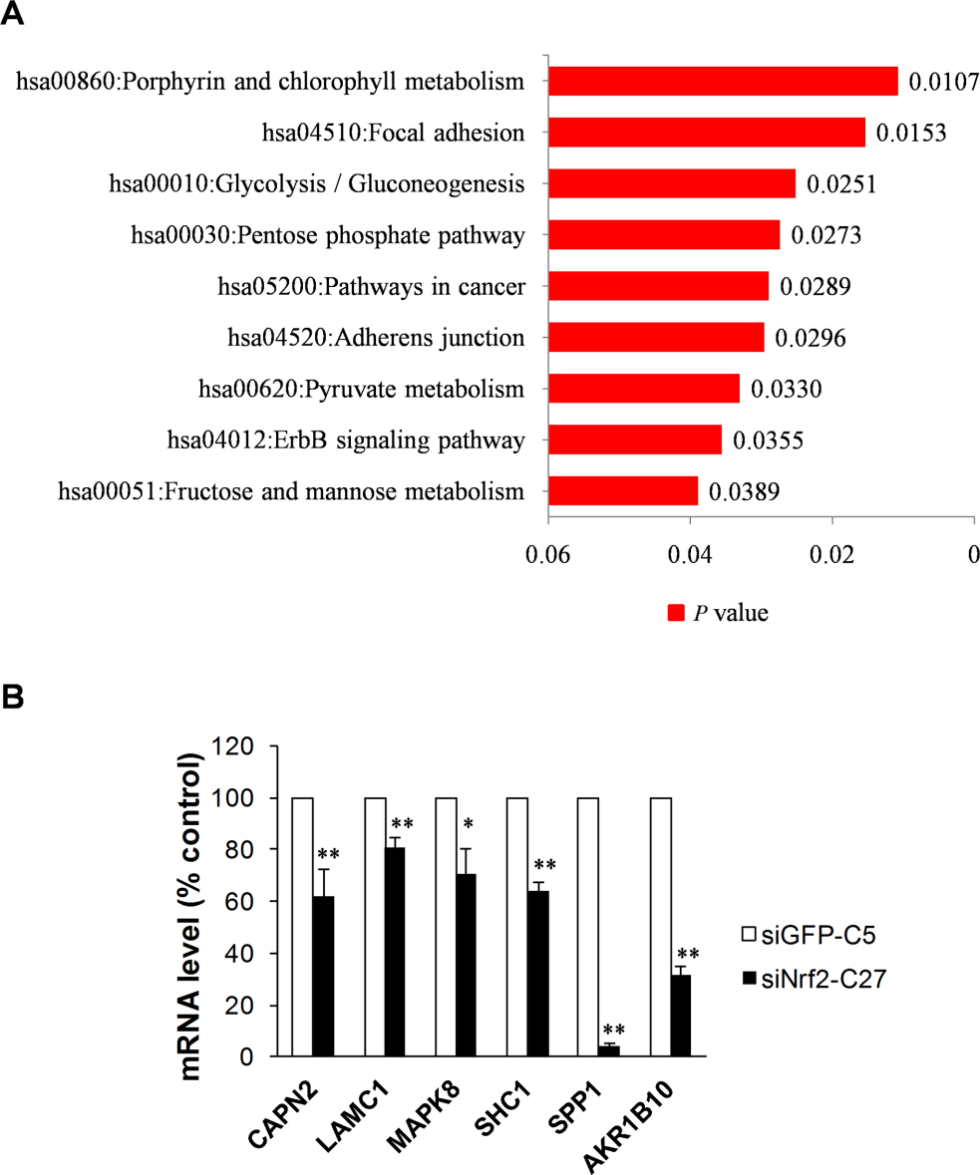
**Functional annotation and validation of novel NRF2-regulated genes using qRT-PCR.** (**A**) Bar chart of KEGG pathways associated with the NRF2 TFBS (P<0.05). (**B**) NRF2-driven focal adhesion pathway genes downregulated in NRF2-knockdown A549 cells (18S rRNA served as an internal control; value for siGFP-C5 cells was set at 100%; *p<0.05, **p<0.01).

We uploaded the list of 2,395 NRF2 TFBS genes into the functional annotation tool DAVID v7 [29] to obtain the KEGG signaling pathways associated with NRF2 in human A549 cells. As a result, we obtained the most significant pathways porphyrin and chlorophyll metabolism (p = 0.01) followed by focal adhesion. Other prominent pathways included glycolysis/gluconeogenesis, pentose phosphate pathway, and ABC transporters, consistent with the known interactions of NRF2. In addition, pathways in cancer, adherens junction, pyruvate metabolism, ErbB signaling pathway, and fructose and mannose metabolism were also identified with p < 0.05 (Figure 4A).

Interestingly, previously unrecognized NRF2-regulated pathways were identified among the nine KEGG pathways identified using DAVID v7 (see Supplementary Table 7). The newly-identified target genes in the KEGG analysis were involved in the focal adhesion and adherens junctions pathways. These two pathways play vital roles in the invasion, migration, and metastasis of tumor cells [30, 31]. The influence of these pathways and their protein expression on cancers, especially on the development and progression of lung carcinoma, is complex.

### NRF2 regulates genes involved in focal adhesion pathways in A549 lung cancer cells

Interestingly, DAVID-KEGG pathway analyses of our ChIP-Seq genes identified a set of 31 candidate genes with 42 binding profiles involved in the focal adhesion pathway. Binding sites in the genes CAPN2, LAMC1, MAPK8, SHC1, SPDYA, and SPP1 were located in promoter-TSS regions and others were located distal to the TSS. Since the binding potency of NRF2 is very high in the promoter regions of genes, we selected those located in the promoter regions for TFBS validation.

We next used qRT-PCR to assess the mRNA expression levels of all these genes in siNRF2-C27 and siGFP-C5cells, which are stable NRF2-siRNA knockdown and control A549 NSCLC cells, respectively. Five of the six genes – CAPN2, LAMC1, MAPK8, SHC1, and SPP1 – had reduced mRNA expression in the NRF2 knockdown cells, and SPDYA did not show any activity (Figure 4B). Thus, the qRT-PCR results suggested that NRF2 regulates the focal adhesion pathway in A549 lung cancer cells through its transcriptional activity.

### LAMC1 – a novel conserved gene regulated by NRF2 in NSCLC

**Figure 5:**
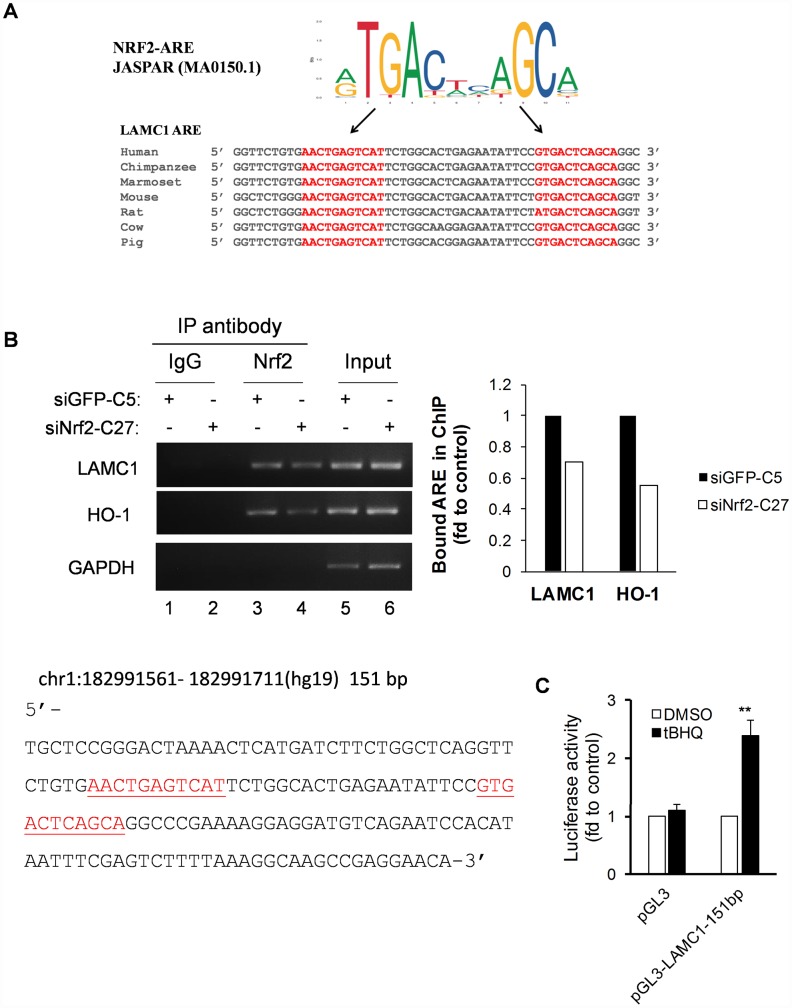
**Conservative ARE analysis and luciferase reporter analysis of the LAMC1 gene.** (**A**) Comparative transcription factor binding site analysis of the LAMC1 gene across different mammalian species shows highly-conserved NRF2 AREs. (**B**) Left, siNRF2-C27 and siGFP-C5 cells subjected to ChIP analysis with anti-Nrf2. Right, the relative ability of NRF2 to bind to the ARE site (value of NRF2 in siGFP-C5 cells set at 1; PCR reactions were not saturated; results are from at least 3 separate experiments; HO-1 served as positive control; GAPDH served as negative control). (**C**) tBHQ increased 151-bp LAMC1 promoter (sequence at left; red indicates ARE sequences)–luciferase activity in MCF7 cells. The plasmid pGL3-LAMC1-151bp was transfected into MCF7 cells in combination with pRL-TK for 24 h. Dual luciferase activity was measured after treatment with tBHQ (20 μM) for 6 h. Control, DMSO treatment for the same plasmid was set at 1 (mean ± SD, n=3; **p <0.01).

To identify potential AREs within the human *LAMC1* gene promoter, we performed *in silico* analysis using the TRAP web tool [22]. A total of two putative ARE sites was identified within 151-bp of the NRF2 TFBS sequence (Figure 5A). Furthermore, comparative promoter alignment analysis of the *LAMC1* binding site sequences across different species using ConTra v3 [32] identified evolutionarily conserved ARE sequences in many species including the mouse (Figure 5A). To further confirm the binding of NRF2 to the promoter region of human *LAMC1*, we carried out ChIP assays in siNRF2-C27 and siGFP-C5 cells. As expected, the NRF2 knockdown reduced its binding to the ARE site in the HO-1 promoter (Figure 5B, lane 4). Importantly, it’s binding to the ARE site in the *LAMC1* promoter was also markedly compromised in siNRF2-C27 cells (Figure 5B, lane 4). To validate the functionality of the AREs in the human *LAMC1* promoter, we cloned the 151 bp of the human NRF2 binding site in the LAMC1 promoter region into the pGL3 luciferase reporter vector. We transfected the pGL3-LAMC1-151bp reporter construct into MCF-7 cells and treated them with 20μM of the NRF2 activator tertiary butylhydroquinone (tBHQ). Interestingly, the transfectants resulted in a 2.4-fold induction of luciferase activity compared to the control pGL3-treated transfectants (Figure 5C). These results suggested that the presence of functional AREs within this genomic region that is regulated in an NRF2-dependent manner. Thus, NRF2 transcriptionally regulates the expression of the LAMC1 gene in NSCLC (Figure 5B, 5C).

## DISCUSSION

Although NRF2 is crucial for chemoprevention, some studies have shown that it is also involved in oncogenesis and drug and/or radio-resistance in many cancers [33, 34]. Due to the “double-edged sword” nature of the NRF2 pathway, it has been considered as an important therapeutic target in many cancers, and the inhibition of NRF2 overexpression could pave the way for inhibiting tumor growth and drug resistance [35, 36]. Also, NRF2 regulates the genes involved in core carbon metabolism pathways such as the pentose phosphate pathway, the tricarboxylic acid cycle, and glycolysis in NSCLC [10, 11]. The recent lines of evidence revealed that NRF2 inhibits the heme- and Fbxo22-mediated degradation of Bach1 by inducing Ho1, and activates the lung cancer metastasis [37]. Interestingly, antioxidants activate the NRF2 that leads to the reduction of free heme levels and stabilization and activation of BACH1. Activated BACH1 increases the transactivation of glycolysis genes such as *Hexokinase 2* and *Gapdh* which stimulates the KRAS-driven lung cancer metastasis [38]. Lee et al*.* [39] showed that BACH1 expression has been increased in triple-negative breast cancer (TNBC) and it decreases the glucose utilization in the tricarboxylic acid cycle. Moreover, BACH1 negatively regulates the transcription of electron transport chain (ETC) genes in TNBC. NRF2 activation also depends on Fructosamine-3-kinase de-glycation. In the absence of Fructosamine-3-kinase, NRF2 is glycated and binds to the small MAF proteins and increases the transactivation of its target genes in liver and lung cancer cells [40].

Previous NRF2–ChIP-Seq studies in normal cell lines have identified the genes involved in cell proliferation [13], adipogenesis [15], NADPH generation [14], and heme metabolism [41]. Here, we used HOMER software to predict TFBSs in the genome of A549 NSCLC cells and identified 2,395 regulatory genomic regions encoding known and novel NRF2-regulated genes. Among the ChIP-Seq binding sites located at the promoters of genes, our results found many known NRF2-regulated genes with their cis-regulatory elements, consistent with previous reports. Furthermore, our ChIP-Seq data showed that the gene binding sites at the TSS promoter comprised ~7% of all binding sites, while most of the binding sites were located in intronic and intergenic regions. Previous reports on A549 cells demonstrated the role of NRF2 in metabolic reprogramming and identified the genes involved in the pentose phosphate pathway and tricarboxylic acid cycle [10, 11], consistent with our data.

The ChIP-Seq results for A549 NSCLC cells surprisingly identified a direct association of NRF2 with the set of genes involved in a novel pathway – focal adhesion which plays major roles in the cell invasion, migration and metastasis of non-small cell lung cancer. NRF2 has been shown to interact with SPP1 in human malignant glioma [42] and Nrf2^−/−^ lung tumors [43]. Lu et al*.* reported that SPP1 increases the expression of HO-1 *via* the activation and enhanced accumulation of NRF2 in the nucleus that ultimately induces cell migration and invasion in glioma cells [42]. However, Satoh et al. reported higher mRNA abundance in Nrf2^−/−^ tumors than in Nrf2^+/+^ lung tumors in mice and hypothesized that Nrf2 negatively regulates the gene expression of Spp1 [43]. Here, we found that SPP1 expression was downregulated in NRF2-knockdown A549 cells, suggesting that NRF2 positively regulates the SPP1 expression in NSCLC cells through its 11-bp consensus ARE. Altogether, these reports suggest that a feedback loop exists between NRF2 and SPP1 in cancer cells. Another study revealed that NRF2 regulated the expression of p66Shc (SHC1 isoform) in human lung cancer cells [44]. They showed that the demethylation of the NRF2 binding site in the p66Shc promoter is required for transactivation and proposed a negative feedback loop between p66Shc and Nrf2 [44]. JNK1 protein, which is encoded by the MAPK8 gene, up-regulates the Nrf2 transactivation activity in HepG2 cells [45]. It is well known that the Jun N-terminal kinase pathway is one of the complex stress-activated protein kinase pathways involved in various signaling pathways, is deregulated in different cancers in humans and mice and is also associated with proliferation, differentiation, survival, and migration in cancer development [46, 47].

It has been reported that calpain-2 (CAPN2) regulates invadopodia and breast cancer invasion [48], promotes tumor growth and the proliferation of cancer cells in the mammary carcinoma through PI3K-Akt-FoxO-p27(Kip1) signaling [49], and is required for glioblastoma cell invasion [50]. In addition, higher expression of CAPN2 is associated with pancreatic cancer [51] and the resistance to platinum-based adjuvant chemotherapy in ovarian cancer [52]. Similarly, higher expression of LAMC1 is associated with a shorter tumor recurrence time and decreased patient survival in meningiomas [53], cancer cell migration, invasion in human prostate cancer cells [54], and tumor progression of the endometrioid carcinoma [55]. Moreover, treatment with LAMC1 peptide C-16 increases the pulmonary metastases of B16-F10 mouse melanoma cells [56]. Of note, overexpression of LAMC1 increases the tumor cell invasion and migration and predicts the poor prognosis in hepatocellular carcinoma [57]. Despite the tumorigenic role of LAMC1 in different cancers, its potential regulatory mechanism needs to be investigated. In the present study, the qPCR, ChIP-qPCR and reporter assay results revealed that LAMC1 was a direct target of NRF2 and it possessed two functional ARE sequences. Our study specifically identified the conserved ARE sites of the *LAMC1* gene across different species. We speculate that hyperactivation of NRF2 in LUAD may lead to over-expression of LAMC1 that ultimately enhances the tumor cell invasion, migration, and metastasis and inhibition of NRF2 pathway is a promising approach for the lung cancer therapy [35, 58]. However, the functions of LAMC1 need to be elucidated further. A very recent study from Bai et al*,* 2019 [59], showed that hyperlipidemic drug-simvastatin reduces cell proliferation and induces apoptosis in breast cancer cells by up-regulating miR-140-5p *via* activation of the transcription factor NRF1. They concluded that LAMC1 is one of the target genes of miR-140-5p and NRF1 contributes to the expression of the ARE-dependent miR-140.

Interestingly, our functional annotation analysis of TFBS genes identified two other important pathways –the ErbB signaling pathway and fructose and mannose metabolism – which play major roles in oncogenesis. The ErbB family of receptor tyrosine kinases is often dysregulated and aberrantly activated in different kinds of tumors. These receptors are considered to be important specific targets for anti-cancer therapeutics due to their localization in the transmembrane region of the cell [60]. NSCLC is one of the most reported among others that harbor epidermal growth factor receptor (EGFR) mutations [61]. It has been shown that EGFR activation elevates NRF2 and the expression of its downstream genes and increases cell proliferation in NSCLC [62]. Our ChIP-Seq results also showed a close association of the ErbB signaling pathway with NRF2 in NSCLC. Of note, a recent study has shown that fructose metabolism affects the growth of liver metastases through metabolic reprogramming in colon cancer metastasis to the liver [63]. ALDOA (aldolase, fructose-bisphosphate A), one of the NRF2-regulated genes identified in our ChIP-Seq results, promotes the metastasis of lung cancer by activating the HIF-1α/MMP9 axis [64]. Thus, the interplay between the NRF2 signaling pathway and other pathways may drive the progression of lung cancer.

Genetic alterations in KEAP1, NRF2, and CUL3 such as somatic mutations, copy number variations, and epigenetic changes are the major reasons for the gain-of-function and aberrant activation of NRF2-regulated pathways in human cancers and are often correlated with poor prognosis and survival [65, 66]. The publicly accessible TCGA-LUAD [67] molecular data sets reported ~17% KEAP1 mutations in patients. Combining the NRF2 TFBS data with upregulated genes in KEAP1 altered patients provides importantly clinically correlated genes for the identification of biomarkers. In our results, we found 26 NRF2 target genes that are significantly upregulated in KEAP1 altered patients as compared with wild type (other than KEAP1 mutations). These genes may serve as possible biomarkers to detect NRF2 activity in NSCLC.

**Figure 6:**
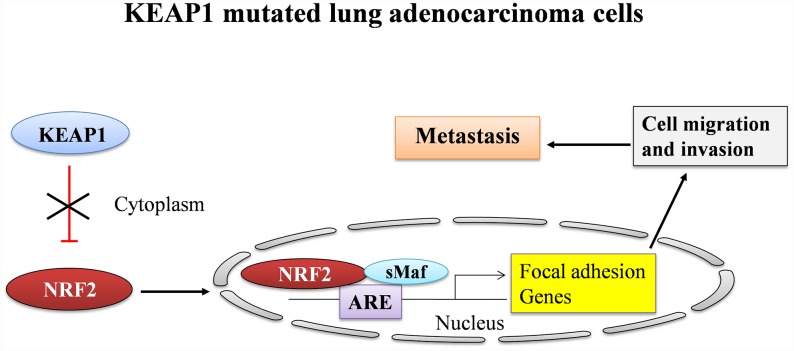
**A graphical summary showing the regulation of focal adhesion genes by NRF2 in LUAD.**

Altogether, our results suggest the fundamental process of how NRF2 participates in the direct regulation of genes involved in invasion, migration, and metastasis in A549 NSCLC cells (Figure 6). However, the detailed molecular mechanisms of this regulation during cancer progression require further investigation. Finally, the complex role of NRF2 in the development of lung cancer makes it a critical target for the development of novel therapeutics. Notably, our results provide compelling evidence that the inhibition of NRF2 and downregulation of its genes involved in cancer progression may offer a therapeutic avenue for the treatment of NSCLC.

## MATERIALS AND METHODS

### Chemicals and cell cultures

Unless otherwise stated, all chemicals were from Sigma–Aldrich Co., Ltd. (Shanghai, China), and all antibodies were from Santa Cruz Biotechnology (Shanghai, China). The A549 (non-small cell lung cancer, NSCLC) and MCF7 cell lines were purchased from the American Type Culture Collection (China). All cells were cultured at 37°C in 95% air and 5% CO_2_, and passaged every 3–4 days. All media supplements for cell cultures were from Invitrogen (China).

### Stable siRNA knockdown cell lines

siNRF2-C27, an A549-derived cell line stably expressing NRF2-siRNA, was developed in this laboratory and described previously [35]. siGFP-C5, an A549-derived cell line stably expressing GFP-siRNA, was also developed in this laboratory [35] and was used as a control cell line.

### Chromatin immunoprecipitation

Chromatin immunoprecipitation (ChIP) experiments were carried out as described previously [35]. Briefly, cells were seeded in 100-mm dishes at 2 × 10^6^ cells/dish then cross-linked with 1% formaldehyde and immunoprecipitation was performed overnight with Nrf2-specific antibody (Santa Cruz Biotechnology) and IgG as control following the instructions with the immunoprecipitation kit (Upperstate, Shanghai, China). The PCR primers for the GAPDH promoter (negative control) were provided in the kit. The PCR primers for the ARE site in the human HO-1 promoter were as described previously [35]. To validate the ARE site in the human LAMC1 promoter, the forward primer 5'-TGCTCCGGGACTAAAACTCAT-3' and the reverse primer 5'-GTTCCTCGGCTTGCCTTTAAAA-3' were used for PCR. The PCRs were not saturated.

### ChIP-Seq analysis

The sequencing and generation of short DNA reads of immunoprecipitated samples were carried out at Genenergy Inc. (Shanghai, China) using an Illumina Hiseq 2000. Reads were mapped against the human reference genome hg19 with Bowtie 0.12.7-1 (bowtie-bio.sourceforge.net); a maximum of 2 mismatches were allowed in the seed. The ChIP-seq data of raw and aligned files have been deposited in NCBI Gene Expression Omnibus, accession number GSE141497.

### Peak calling

The significant peaks (NRF2 binding sites) of mapped reads were identified using the program ‘findPeaks’ from the freely-available software suite HOMER (Hypergeometric Optimization of Motif EnRichment) [16] with the following parameters: false discovery rate = 0.001, Poisson p-value over input required = 1.00e-04, fold over input required = 4.00, maximum fold under expected unique positions for tags = 2.00, and normalization to 10 million mapped tags per experiment. We also used the web-based ChIP-Seq analysis tool ChIPseek [17] to obtain the genomic annotation and visualization of NRF2 TFBSs.

### De novo motif discovery analysis

We used the‘findMotifsGenome.pl’ program of the HOMER software package to perform *de novo* motif discovery as well as identification of the enriched sequence motifs within the ChIP-Seq peak regions. Enrichment of *de novo* motifs was determined by comparing all 2,022 target sequences with all 46,776 background sequences using the cumulative hypergeometric distribution algorithm. We further submitted the *de novo* motif matrix to the STAMP website to assess the similarity between known candidate motifs [18].

### Network analysis of NRF2 TFBSs

We uploaded the gene symbols identified from the ChIP-Seq results into the eXpression2Kinases (X2K) web tool [68] for transcription factor enrichment analysis, to generate a protein-protein interaction network, and for kinase enrichment analysis.

### *In Silico* analysis

The Identification of putative NRF2 AREs in the 151-bp binding region of human LAMC1 was carried out using the TRAP web tool [22]. This program calculates the total binding affinity of each transcription factor to the desired sequence and compares the binding affinity to the distribution in the background model (Human and others) by "hit-based" ranking using p-values <0.05. We used the JASPAR matrix MA0150.1 [69] to identify the NRF2-AREs in TRAP analysis. The NRF2 binding region of the LAMC1 promoter (151 bp) at chr1:182991561 to 182991711(hg19) was used for the TRAP analysis. In addition, comparative genomics-species alignment was also performed using the ConTra v3 web tool [32].

### Integrated analysis of ChIP-Seq data with NRF2 knockdown microarray and RNA-Seq data from TCGA LUAD patients

We used publicly-available microarray data from NRF2-knockdown A549 cells retrieved from NCBI Gene Expression Omnibus (accession number GSE28230) [10] to perform integrated gene expression profiling of NRF2 in lung cancer. We used GeneSpring GX software (Agilent) to analyze the GSE28230 microarray data and obtained differentially-regulated genes with a 1.25-fold change. We next obtained overlapping genes by combing our ChIP-Seq TFBS list with genes downregulated 1.25-fold using the biostatistical web tool Venny 2.0 [28]. We also performed the integrated analysis of ChIP-Seq data with RNA-Seq data from TCGA LUAD [67] patients with altered KEAP1 as described previously [66].

### Functional pathway analysis

The ChIP-Seq TFBSs containing gene symbols were uploaded into the DAVID bioinformatics web tool [70] as described previously [66].

### Validation of ChIP-Seq data using qRT-PCR

Isolation of total RNA and qRT-PCR were performed as described previously [35, 71]. The primers were synthesized by TaKaRa Biotechnology (Dalian, China). qRT-PCR using the validated SYBR® Green assays were carried out on a LightCycler® 480 instrument (Roche, Mannheim, Germany). The primer sequences are listed in Supplementary Table 8. Each assay was performed in triplicate. The results were analyzed with 480II Real Time PCR System software (Roche, Shanghai, China). The 18S rRNA level served as an internal standard.

### Plasmid

pGL3-LAMC1-151bp is a luciferase reporter plasmid with the 151-bp human LAMC1 promoter. The construct was made by amplification of the 151-bp DNA fragment from human LAMC1 (hg19, chr1:182991561-182991711) (upstream to the TSS) by PCR with the forward primer 5'-ATGGTACCTGCTCCGGGACTAAAACTCA-3' and the reverse primer 5'-TACTCGAGTGTTCCTCGGCTTGCCTTTA-3'. The amplified DNA fragment was subsequently cloned into the pGL3-promoter vector (Promega, Shanghai, China) *via* the *Xho*I and *Kpn*I restriction sites and verified by DNA sequencing.

### Analysis of luciferase reporter gene activity

The luciferase reporter plasmid pGL3-LAMC1-151bp was transiently transfected using Lipofectamine 2000 (Invitrogen) as described previously [35]. The pRL-TK plasmid, which encodes *Renilla* luciferase, was used as a control for transfection efficiency. The dual luciferase activity was determined using the Dual-luciferase Reporter Assay System (Promega) as described in the manufacturer’s instructions.

### Statistical analysis

Statistical comparisons were performed with unpaired Student’s t-tests. A value of p<0.05 was considered statistically significant.

## Supplementary Material

Supplementary Figure

Supplementary Tables

Supplementary Table 1

Supplementary Table 2

Supplementary Table 3

Supplementary Table 4
