# Supplementary material for: Genome-wide global identification of NRF2 binding sites in A549 non-small cell lung cancer cells by ChIP-Seq reveals NRF2 regulation of genes involved in focal adhesion pathways
Source: Aging (Albany NY). 2019 Dec 28;11(24):12600–23. doi: 10.18632/aging.102590 (PMC6949066; doi:10.18632/aging.102590)
Supplement: Supplementary Figure [file aging-11-102590-s006..pdf]

SUPPLEMENTARY FIGURES

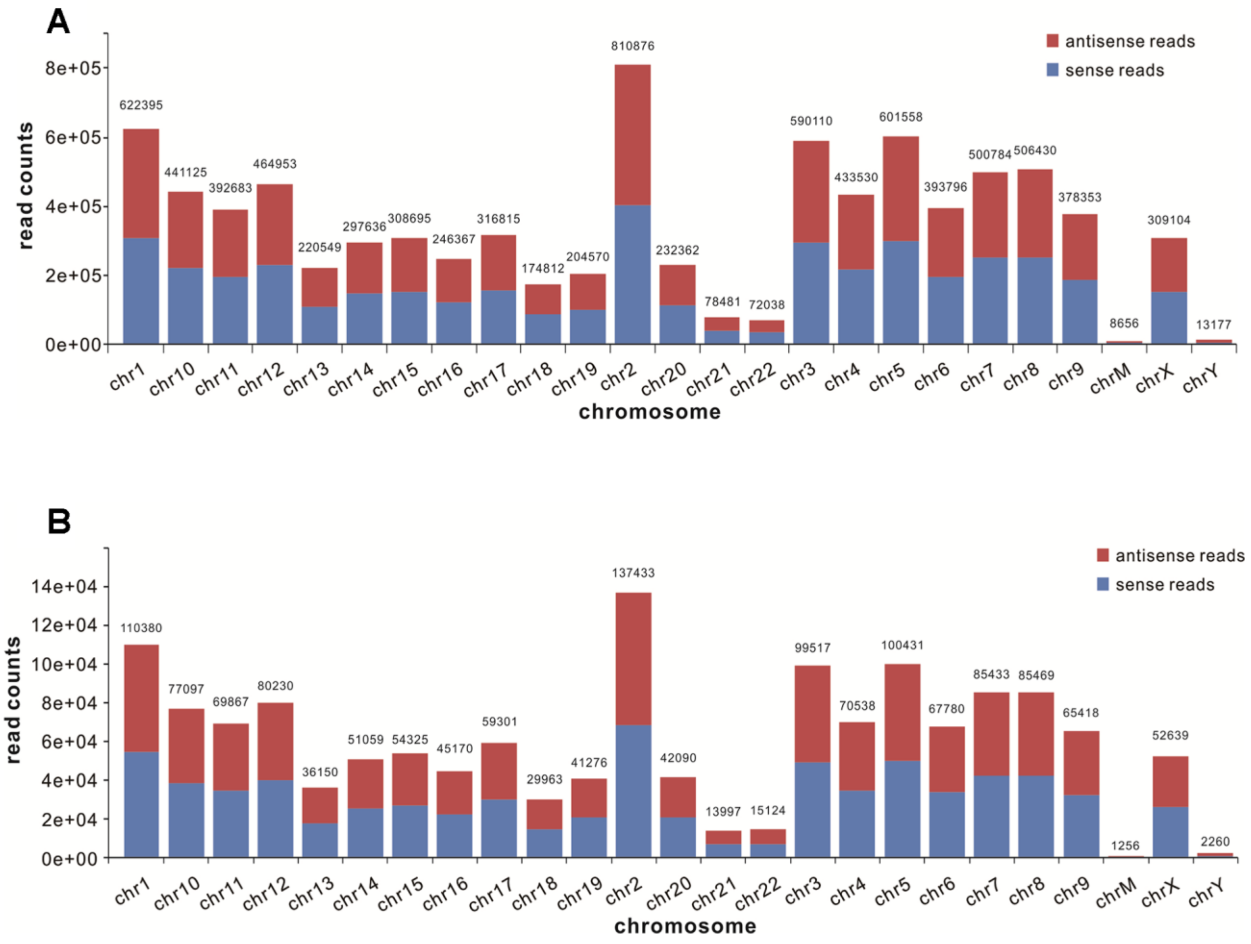

Supplementary Figure 1. Sequence alignment of ChIPed DNA A, Bar chart of the total number of aligned tags against the human genome (hg 19) for control (**A**) and input DNA (**B**).

A

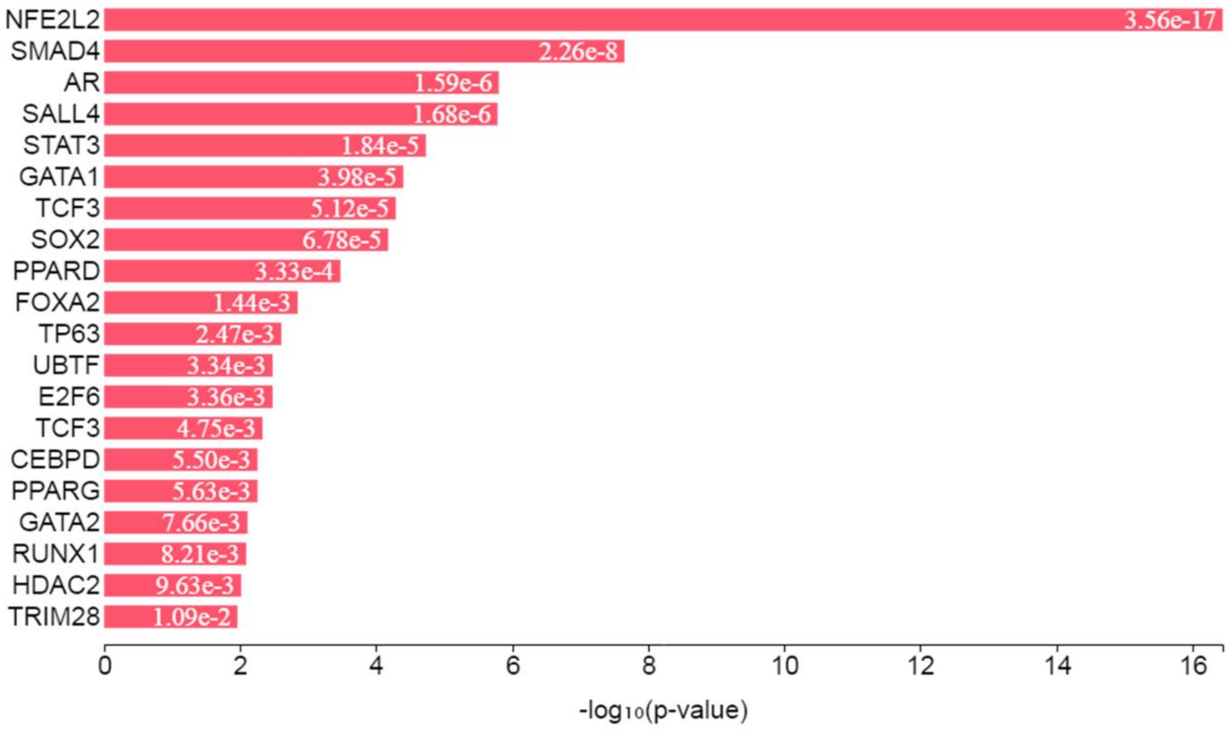
NFE2L2 3.56e-17
SMAD4 2.26e-8
AR 1.59e-6
SALL4 1.68e-6
STAT3 1.84e-5
GATA1 3.98e-5
TCF3 5.12e-5
SOX2 6.78e-5
PPARD 3.33e-4
FOXA2 1.44e-3
TP63 2.47e-3
UBTF 3.34e-3
E2F6 3.36e-3
TCF3 4.75e-3
CEBPD 5.50e-3
PPARG 5.63e-3
GATA2 7.66e-3
RUNX1 8.21e-3
HDAC2 9.63e-3
TRIM28 1.09e-2
0
2
4
6
8
10
12
14
16
-log10(p-value)

B

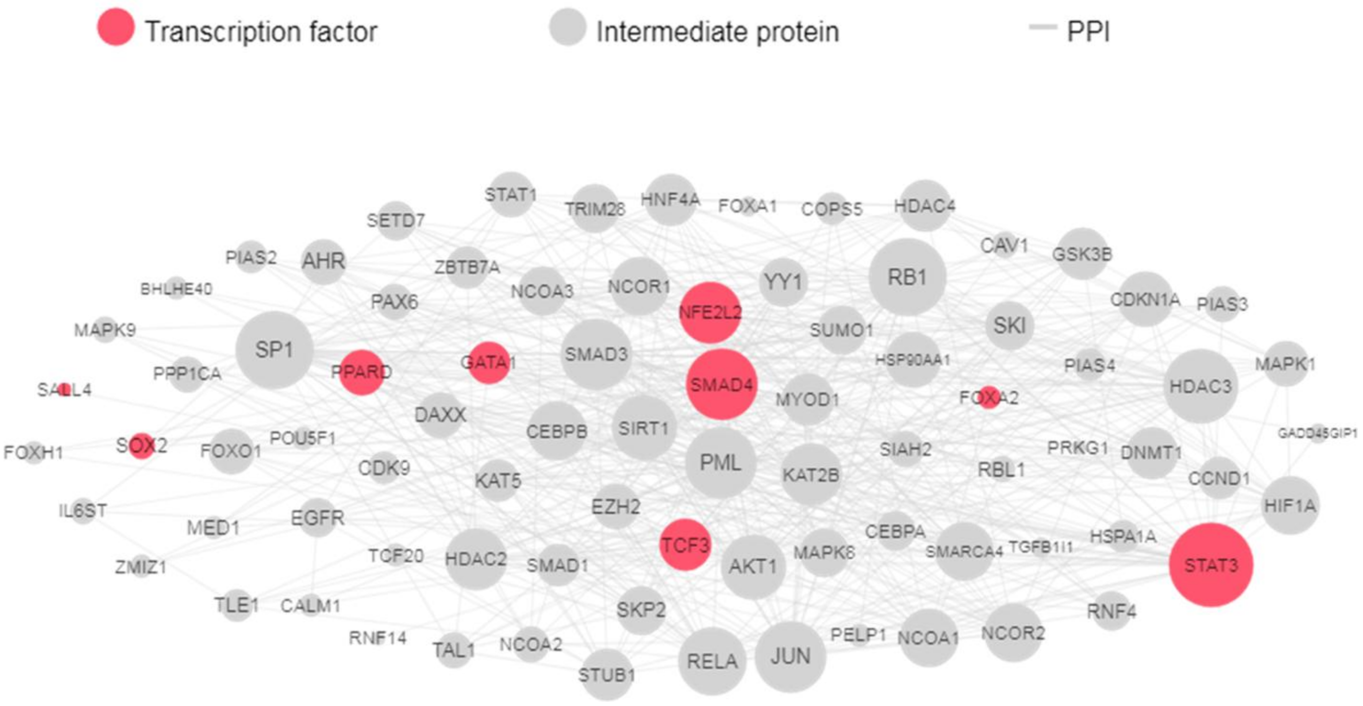
Transcription factor
Intermediate protein
PPI
STAT1
TRIM28
HNF4A
FOXA1
COPS5
HDAC4
SETD7
CAV1
GSK3B
PIAS2
AHR
ZBTB7A
RB1
BHLHE40
PAX6
NCOA3
NCOR1
YY1
CDKN1A
PIAS3
NFE2L2
MAPK9
SP1
SMAD3
SUMO1
SKI
PPARD
GATA1
HSP90AA1
PIAS4
MAPK1
PPP1CA
SMAD4
HDAC3
SALL4
MYOD1
FOXA2
DAXX
CEBPB
SIRT1
GADD45GIP1
SOX2
FOXH1
FOXO1
POU5F1
PML
SIAH2
PRKG1
DNMT1
CDK9
KAT5
KAT2B
RBL1
CCND1
IL6ST
EZH2
HIF1A
MED1
EGFR
CEBPA
TCF3
HSPA1A
MAPK8
SMARCA4
TGFB1I1
ZMIZ1
TCF20
HDAC2
SMAD1
AKT1
STAT3
TLE1
CALM1
SKP2
RNF4
RNF14
TAL1
NCOA2
PELP1
NCOA1
NCOR2
STUB1
RELA
JUN

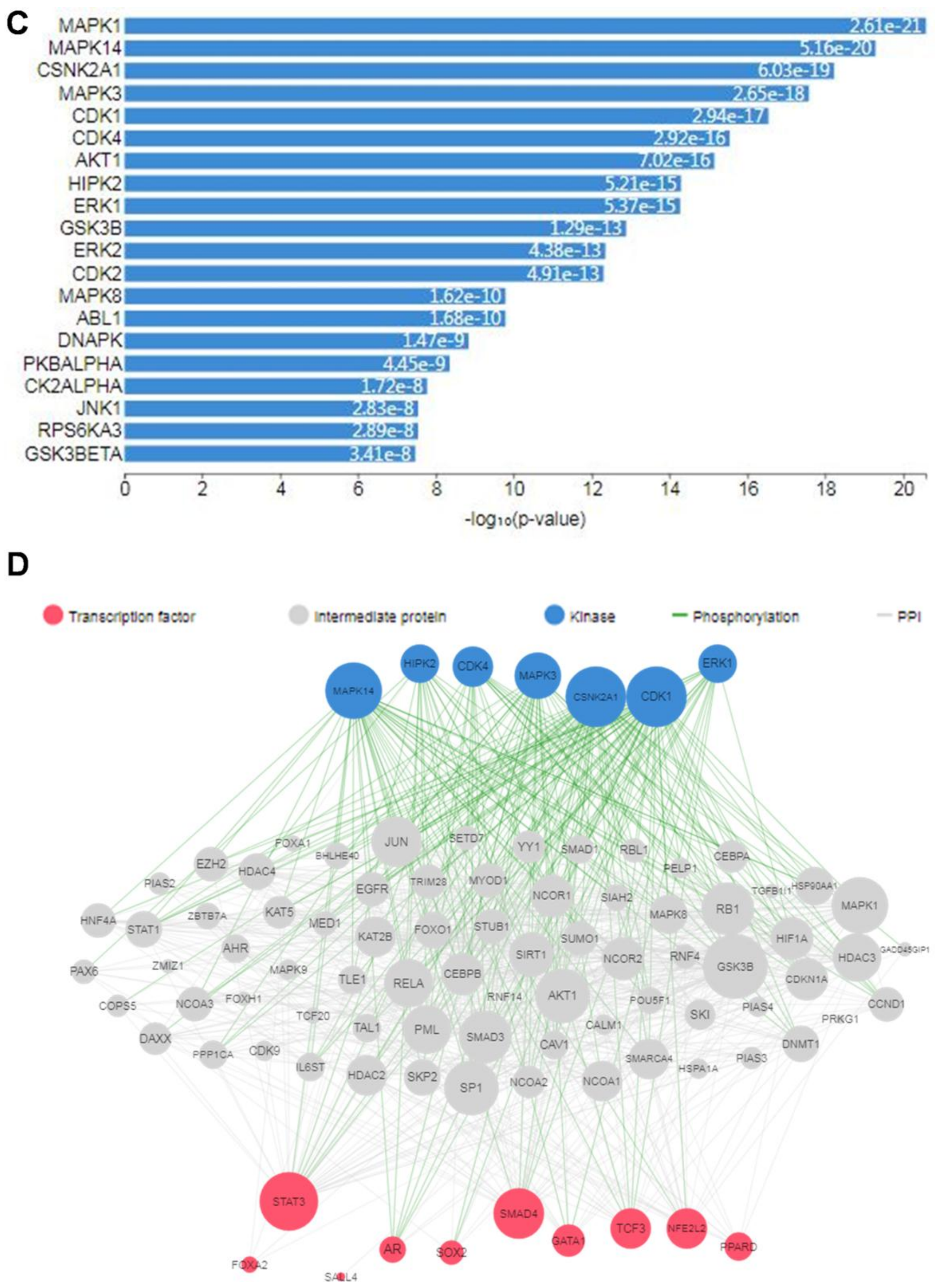

Supplementary Figure 2. eXpression2Kinases analysis of the NRF2 TFBS. (**A**). Bar graph of the transcription factor enrichment analysis results containing various transcription factors associated with the NRF2 TFBS. NRF2 ranked the top transcription factor. (**B**) Protein-protein interaction sub-network showing the association of enriched transcription factors (red) with intermediate proteins (gray). (**C**) Bar graph of the kinase enrichment analysis results for the NRF2 TFBS. (**D**) Sub-network showing the association of all kinases at the top (blue), the intermediate proteins (gray) in the middle, and the transcription factors (red) below (green lines connecting kinase nodes to intermediate proteins represent the phosphorylated substrates).

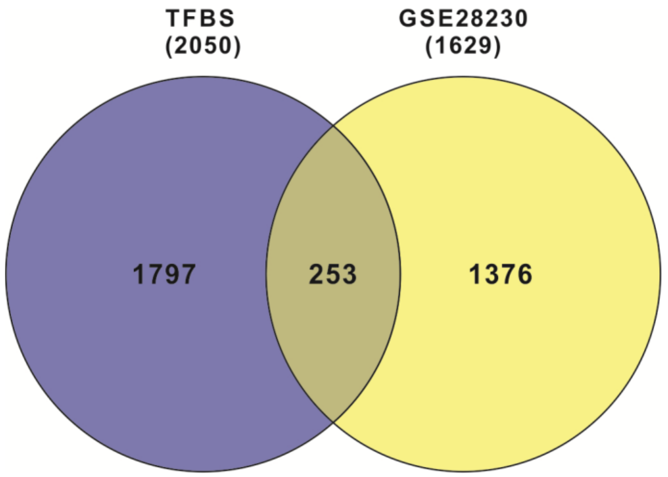

Supplementary Figure 3. Venn diagram showing the overlapping genes of the NRF2 TFBS and downregulated genes in the NRF2-knockdown microarray (FC>1.25).
