# Supplementary material for: Genome-wide global identification of NRF2 binding sites in A549 non-small cell lung cancer cells by ChIP-Seq reveals NRF2 regulation of genes involved in focal adhesion pathways
Source: Aging (Albany NY). 2019 Dec 28;11(24):12600–23. doi: 10.18632/aging.102590 (PMC6949066; doi:10.18632/aging.102590)
Supplement: Supplementary Tables [file aging-11-102590-s004..pdf]

## Supplementary Tables

Please browse Full Text version to see the data of Supplementary Tables 1 to 4.

**Supplementary Table 1. NRF2 binding regions in A549 cells identified using ChIP-Seq.**

**Supplementary Table 2. Known motifs identified by HOMER motif enrichment analysis software.**

**Supplementary Table 3. NRF2 binding regions in the promoter region.**

**Supplementary Table 4. Overlapping genes from an integrated analysis of NRF2 TFBS and downregulated genes in the NRF2-knockdown microarray (FC>1.25).**

**Supplementary Table 5. KEGG pathways from DAVID analysis of overlapping genes.**

| Term | PValue | Genes |
|---|---|---|
| hsa00860:Porphyrin and chlorophyll metabolism | 2.76E-04 | FTMT, UGT1A6, FECH, HMOX1, BLVRB, FTH1 |
| hsa00980:Metabolism of xenobiotics by cytochrome P450 | 6.79E-04 | AKR1C3, AKR1C2, UGT1A6, EPHX1, AKR1C1, MGST1, ALDH3A1 |
| hsa00030:Pentose phosphate pathway | 9.49E-04 | ALDOA, TALDO1, PGD, PFKP, TKT |
| hsa00480:Glutathione metabolism | 0.001937 | GPX2, GSR, GCLC, PGD, GCLM, MGST1 |
| hsa00051:Fructose and mannose metabolism | 0.003073 | ALDOA, TPI1, AKR1B10, AKR1B1, PFKP |
| hsa00590:Arachidonic acid metabolism | 0.018007 | AKR1C3, GPX2, TBXAS1, CYP4F3, CYP4F2 |
| hsa00010:Glycolysis / Gluconeogenesis | 0.022643 | ALDOA, TPI1, PFKP, ALDH3A2, ALDH3A1 |
| hsa00620:Pyruvate metabolism | 0.034972 | ME1, AKR1B1, ALDH3A2, MDH2 |
| hsa00053:Ascorbate and aldarate metabolism | 0.03692 | UGT1A6, UGDH, ALDH3A2 |
| hsa00040:Pentose and glucuronate interconversions | 0.041055 | UGT1A6, AKR1B1, UGDH |
| hsa00140:Steroid hormone biosynthesis | 0.049758 | AKR1C3, AKR1C2, UGT1A6, AKR1C1 |

**Supplementary Table 6. NRF2 TFBS genes clinically correlated with data from TCGA LUAD patients with altered KEAP1.**

| Gene | Log Ratio | p-Value | q-Value | Tendency |
|---|---|---|---|---|
| AKR1C2 | 6.54 | 5.01E-19 | 1.15E-15 | Over-expressed |
| GPX2 | 5.51 | 4.57E-19 | 1.15E-15 | Over-expressed |
| AKR1C1 | 5.31 | 1.50E-16 | 1.72E-13 | Over-expressed |
| ABCC2 | 3.94 | 1.57E-09 | 2.69E-07 | Over-expressed |
| AKR1C3 | 3.66 | 8.52E-17 | 1.06E-13 | Over-expressed |
| UCHL1 | 2.99 | 9.51E-11 | 2.42E-08 | Over-expressed |
| SLC7A11 | 2.72 | 5.31E-15 | 3.54E-12 | Over-expressed |
| TRIM16L | 2.72 | 1.09E-20 | 7.49E-17 | Over-expressed |
| NQO1 | 2.65 | 1.13E-21 | 1.56E-17 | Over-expressed |
| OSGIN1 | 2.39 | 5.02E-14 | 2.87E-11 | Over-expressed |
| SRXN1 | 2.37 | 3.68E-16 | 3.89E-13 | Over-expressed |
| CYP24A1 | 2.14 | 3.99E-05 | 4.71E-04 | Over-expressed |
| TXNRD1 | 2.09 | 2.45E-13 | 1.24E-10 | Over-expressed |
| PIR | 2.02 | 5.52E-20 | 2.53E-16 | Over-expressed |
| UGDH | 1.96 | 1.95E-18 | 3.82E-15 | Over-expressed |
| CES1 | 1.95 | 1.48E-04 | 1.29E-03 | Over-expressed |
| KYNU | 1.89 | 6.88E-06 | 1.25E-04 | Over-expressed |
| NEIL3 | 1.82 | 1.89E-07 | 8.79E-06 | Over-expressed |
| GCLC | 1.81 | 2.17E-08 | 1.90E-06 | Over-expressed |
| RSPO3 | 1.73 | 4.99E-04 | 3.22E-03 | Over-expressed |
| CARD14 | 1.7 | 4.32E-08 | 3.04E-06 | Over-expressed |
| PGD | 1.68 | 9.21E-18 | 1.40E-14 | Over-expressed |
| ABCB6 | 1.66 | 7.94E-16 | 7.27E-13 | Over-expressed |
| PTGR1 | 1.63 | 1.21E-13 | 6.39E-11 | Over-expressed |
| GCLM | 1.59 | 1.37E-15 | 1.10E-12 | Over-expressed |
| GSR | 1.55 | 2.24E-18 | 3.85E-15 | Over-expressed |

**Supplementary Table 7. KEGG pathways from DAVID analysis of NRF2 TFBS genes.**

| Term | PValue | Genes |
|---|---|---|
| hsa00860:Porphyrin and chlorophyll metabolism | 0.010765 | FTMT, UGT1A6, FECH, HMOX1, CPOX, BLVRB, CP, FTH1, EARS2 |
| hsa04510:Focal adhesion | 0.015366 | CAV2, TLN2, PDGFA, ITGB4, ITGB1, PTEN, SRC, CTNNB1, SHC1, PIK3R1, SPP1, PRKCA, COL4A4, ACTN4, ACTN1, MAPK10, HGF, FLNC, COL5A3, CAPN2, PPP1CB, FLNB, COL5A1, COL4A6, SPDYA, LAMA2, VEGFC, LAMA3, CCND3, MAPK8, LAMC1 |
| hsa00010:Glycolysis/ Gluconeogenesis | 0.025179 | ALDOA, GPI, TPI1, G6PC, LDHAL6B, DLD, PFKP, ADH7, GAPDH, ALDH3A2, ALDH3A1, ENO1 |
| hsa00030:Pentose phosphate pathway | 0.02736 | ALDOA, GPI, TALDO1, PGD, PFKP, TKT, LOC729020 |
| hsa05200:Pathways in cancer | 0.028938 | BID, FGFR2, PTGS2, PDGFA, EGLN3, NFKBIA, NFKB1, GLI2, ITGB1, TCF7L2, PTEN, CTNNB1, TGFA, RARA, FGF1, MYC, PIK3R1, CEBPA, COL4A4, PRKCA, AR, HSP90AA1, CTBP2, TGFBR2, SMAD3, CDK6, MAPK10, HGF, MECOM, COL4A6, FZD7, CTNNA3, LAMA2, VEGFC, CBLB, FZD10, LAMA3, HIF1A, PLCG1, IKBKG, MAPK8, LAMC1, GSTP1 |
| hsa04520:Adherens junction | 0.029617 | PTPRB, PTPRJ, PARD3, WASF3, ACTN4, TGFBR2, SMAD3, ACTN1, FER, TCF7L2, CTNNA3, SRC, CTNNB1, TJP1 |
| hsa00620:Pyruvate metabolism | 0.033025 | ME1, LDHAL6B, AKR1B1, DLD, ACYP2, ACACB, ALDH3A2, MDH2, PC |
| hsa04012:ErbB signaling pathway | 0.035574 | PRKCA, NRG3, BTC, MAPK10, SRC, CBLB, PLCG1, EREG, CAMK2D, TGFA, SHC1, MAPK8, MYC, ABL2, PIK3R1 |
| hsa00051:Fructose and mannose metabolism | 0.038929 | MTMR2, ALDOA, TPI1, AKR1B15, MPI, PFKFB3, AKR1B10, AKR1B1, PFKP |

**Supplementary Table 8. Primers used for qRT-PCR analysis.**

| | | |
|---|---|---|
| CAPN2 | Forward | 5′-GAGTTGAAGAAGCCCCCTCC-3′ |
| | Reverse | 5′-GCTAGATTCGGACGGGAGTG-3′ |
| LAMC1 | Forward | 5′-CACTGTAGTCCTGTGGGCTC-3′ |
| | Reverse | 5′-GTAAGCCAGGAGTGATGGGG-3′ |
| MAPK8 | Forward | 5′-TGAAGCAGAAGCTCCACCAC-3′ |
| | Reverse | 5′-CAGCCCTCTCCTTTAGGTGC-3′ |
| SHC1 | Forward | 5′-ACTTGGGAGCTACATTGCCTG-3′ |
| | Reverse | 5′-GCAAACAGATGCCACCTCCA-3′ |
| SPDYA | Forward | 5′-CTTCCGCAGTCCTTCCTTCAG-3′ |
| | Reverse | 5′-GATGTGTTGTGAGACACCACC-3′ |
| SPP1 | Forward | 5′-AAATACCCAGATGCTGTGGC-3′ |
| | Reverse | 5′-CCCACAGACCCTTCCAAGTAAG-3′ |
| AKR1B10 | Forward | 5′-TGTGGGCCTGGGCACTTGGA-3′ |
| | Reverse | 5′-GGTCCTCCCGCTTCACAGCC-3′ |
