# Supplementary material for: Genome-wide global identification of NRF2 binding sites in A549 non-small cell lung cancer cells by ChIP-Seq reveals NRF2 regulation of genes involved in focal adhesion pathways
Source: Aging (Albany NY). 2019 Dec 28;11(24):12600–23. doi: 10.18632/aging.102590 (PMC6949066; doi:10.18632/aging.102590)
Supplement: Supplementary Table 1 [file aging-11-102590-s005..docx]

**Supplementary Table 1. NRF2 binding regions in A549 cells identified using ChIP-Seq.**

| **PeakID** | **Chr** | **Start** | **End** | **Peak Score** | **Annotation** | **Distance to TSS** | **Gene Name** |
| --- | --- | --- | --- | --- | --- | --- | --- |
| chr5-1 | chr5 | 172197694 | 172197844 | 662.6 | exon (NM_004417, exon 1 of 4) | 434 | DUSP1 |
| chr2-1 | chr2 | 232325073 | 232325223 | 508.6 | promoter-TSS (NR_004398) | 5 | SNORD82 |
| chr16-1 | chr16 | 69760851 | 69761001 | 435 | promoter-TSS (NM_001025433) | -393 | NQO1 |
| chr5-2 | chr5 | 172196621 | 172196771 | 421.6 | exon (NM_004417, exon 3 of 4) | 1507 | DUSP1 |
| chr1-3 | chr1 | 27720670 | 27720820 | 421.6 | exon (NM_005281, exon 2 of 2) | 1593 | GPR3 |
| chr5-3 | chr5 | 172195908 | 172196058 | 414.9 | exon (NM_004417, exon 4 of 4) | 2220 | DUSP1 |
| chr12-1 | chr12 | 104680633 | 104680783 | 388.2 | 5' UTR (NM_182729, exon 1 of 15) | 248 | TXNRD1 |
| chr21-1 | chr21 | 46032420 | 46032570 | 374.8 | exon (NM_198695, exon 1 of 1) | 499 | KRTAP10-8 |
| chr22-1 | chr22 | 21800745 | 21800895 | 374.8 | exon (NM_015094, exon 3 of 3) | -20639 | TMEM191C |
| chr5-4 | chr5 | 180687068 | 180687218 | 368.1 | exon (NM_032765, exon 1 of 2) | 976 | TRIM52 |
| chr2-2 | chr2 | 234608824 | 234608974 | 354.7 | intron (NM_001072, intron 1 of 4) | 7387 | UGT1A6 |
| chr12-2 | chr12 | 56374306 | 56374456 | 348 | intron (NM_001252037, intron 1 of 4) | 6586 | RAB5B |
| chr17-1 | chr17 | 19656424 | 19656574 | 341.3 | Intergenic | -4753 | ALDH3A1 |
| chr19-1 | chr19 | 16059862 | 16060012 | 334.6 | exon (NM_001004465, exon 1 of 1) | 119 | OR10H4 |
| chr19-3 | chr19 | 23542445 | 23542595 | 327.9 | exon (NM_003430, exon 4 of 4) | 35749 | ZNF91 |
| chr6-1 | chr6 | 87607110 | 87607260 | 327.9 | Intergenic | -39839 | HTR1E |
| chr2-3 | chr2 | 178096484 | 178096634 | 314.5 | exon (NM_001145413, exon 5 of 5) | 19105 | MIR4444-1 |
| chr2-4 | chr2 | 242438417 | 242438567 | 307.9 | exon (NM_006374, exon 7 of 12) | 9542 | STK25 |
| chr17-2 | chr17 | 48739252 | 48739402 | 301.2 | intron (NM_001144070, intron 8 of 11) | 27109 | ABCC3 |
| chr5-5 | chr5 | 172197176 | 172197326 | 301.2 | exon (NM_004417, exon 2 of 4) | 952 | DUSP1 |
| chr9-1 | chr9 | 113019158 | 113019308 | 301.2 | promoter-TSS (NM_003329) | -313 | TXN |
| chr11-1 | chr11 | 76480875 | 76481025 | 301.2 | Intergenic | -12407 | TSKU |
| chr22-2 | chr22 | 20102286 | 20102436 | 294.5 | exon (NM_022727, exon 6 of 12) | 2457 | TRMT2A |
| chr3-1 | chr3 | 53304190 | 53304340 | 287.8 | Intergenic | -14135 | TKT |
| chr15-1 | chr15 | 40627184 | 40627334 | 287.8 | 3' UTR (NM_207380, exon 11 of 11) | 5909 | C15orf52 |
| chr18-1 | chr18 | 55321095 | 55321245 | 287.8 | intron (NM_005603, intron 24 of 27) | 23636 | LOC100505549 |
| chr8-1 | chr8 | 62634532 | 62634682 | 281.1 | Intergenic | 7260 | MIR4470 |
| chr19-4 | chr19 | 6375729 | 6375879 | 281.1 | promoter-TSS (NM_004158) | 56 | PSPN |
| chr10-3 | chr10 | 15254733 | 15254883 | 281.1 | 3' UTR (NM_001010924, exon 8 of 8) | -44113 | NMT2 |
| chr22-3 | chr22 | 21119076 | 21119226 | 281.1 | exon (NM_058004, exon 22 of 55) | -9232 | SERPIND1 |
| chr10-2 | chr10 | 121398165 | 121398315 | 281.1 | Intergenic | -12642 | BAG3 |
| chr17-3 | chr17 | 78013977 | 78014127 | 274.4 | exon (NM_001243342, exon 3 of 18) | 3621 | CCDC40 |
| chr2-5 | chr2 | 102968135 | 102968285 | 267.7 | exon (NM_016232, exon 11 of 11) | -10887 | IL18R1 |
| chrX-1 | chrX | 153770261 | 153770411 | 267.7 | promoter-TSS (NM_001099856) | -123 | IKBKG |
| chr21-2 | chr21 | 45959420 | 45959570 | 261 | exon (NM_198691, exon 1 of 1) | 583 | KRTAP10-1 |
| chr20-1 | chr20 | 39794263 | 39794413 | 261 | exon (NM_002660, exon 16 of 32) | 28177 | PLCG1 |
| chr2-7 | chr2 | 178098762 | 178098912 | 254.3 | exon (NM_001145413, exon 2 of 5) | 21383 | MIR4444-1 |
| chr10-4 | chr10 | 53457482 | 53457632 | 254.3 | exon (NM_015235, exon 1 of 1) | 1798 | CSTF2T |
| chr2-6 | chr2 | 29353260 | 29353410 | 254.3 | intron (NM_024692, intron 2 of 15) | 15027 | CLIP4 |
| chr16-2 | chr16 | 75680480 | 75680630 | 254.3 | intron (NM_001130089, intron 1 of 14) | 1030 | KARS |
| chr3-2 | chr3 | 14452321 | 14452471 | 247.6 | intron (NM_001134368, intron 1 of 4) | 8290 | SLC6A6 |
| chr21-3 | chr21 | 9825977 | 9826127 | 247.6 | promoter-TSS (NR_037458) | -151 | MIR3687 |
| chr4-1 | chr4 | 13602629 | 13602779 | 247.6 | exon (NM_148894, exon 10 of 26) | 26624 | BOD1L1 |
| chr7-3 | chr7 | 296783 | 296933 | 240.9 | intron (NM_020223, intron 7 of 9) | -33278 | LOC100288524 |
| chr1-7 | chr1 | 186147633 | 186147783 | 234.2 | exon (NM_031935, exon 104 of 107) | -117697 | PRG4 |
| chr15-3 | chr15 | 64791831 | 64791981 | 234.2 | exon (NM_015042, exon 1 of 9) | 287 | ZNF609 |
| chr17-4 | chr17 | 73702763 | 73702913 | 234.2 | 3' UTR (NM_013260, exon 11 of 11) | -14678 | ITGB4 |
| chr1-5 | chr1 | 35652587 | 35652737 | 234.2 | exon (NM_005066, exon 9 of 10) | 6081 | SFPQ |
| chr17-5 | chr17 | 36704775 | 36704925 | 227.5 | exon (NM_025248, exon 17 of 19) | 57333 | SRCIN1 |
| chr4-2 | chr4 | 139156764 | 139156914 | 227.5 | intron (NM_014331, intron 2 of 11) | 6664 | SLC7A11 |
| chr6-5 | chr6 | 53480770 | 53480920 | 227.5 | Intergenic | 49661 | KLHL31 |
| chr20-2 | chr20 | 22392608 | 22392758 | 227.5 | intron (NR_027089, intron 1 of 3) | 8598 | LOC284788 |
| chr10-6 | chr10 | 42379837 | 42379987 | 227.5 | Intergenic | 483581 | LOC441666 |
| chr1-9 | chr1 | 109803641 | 109803791 | 227.5 | exon (NM_001408, exon 3 of 34) | 11075 | CELSR2 |
| chr7-2 | chr7 | 2962156 | 2962306 | 227.5 | intron (NM_032415, intron 17 of 24) | -78272 | GNA12 |
| chr19-6 | chr19 | 54080479 | 54080629 | 220.9 | exon (NM_001079906, exon 6 of 6) | 21993 | ZNF331 |
| chr15-2 | chr15 | 44855189 | 44855339 | 220.9 | TTS (NM_003758) | 25998 | EIF3J |
| chr2-8 | chr2 | 178097153 | 178097303 | 220.9 | exon (NM_001145413, exon 4 of 5) | 19774 | MIR4444-1 |
| chr8-2 | chr8 | 129569030 | 129569180 | 220.9 | Intergenic | 406743 | MIR1208 |
| chr2-10 | chr2 | 196642537 | 196642687 | 220.9 | exon (NM_018897, exon 59 of 65) | 120760 | SLC39A10 |
| chr7-4 | chr7 | 56019618 | 56019768 | 214.2 | promoter-TSS (NM_015969) | 82 | MRPS17 |
| chr12-3 | chr12 | 16553292 | 16553442 | 214.2 | Intergenic | 47016 | MGST1 |
| chr1-6 | chr1 | 156186352 | 156186502 | 214.2 | intron (NM_001199664, intron 1 of 4) | 3648 | PMF1-BGLAP |
| chr2-9 | chr2 | 85548338 | 85548488 | 214.2 | 3' UTR (NM_006464, exon 4 of 4) | 7006 | TGOLN2 |
| chr3-3 | chr3 | 126391470 | 126391620 | 207.5 | TTS (NR_034158) | 10621 | NUP210P1 |
| chr11-3 | chr11 | 17409382 | 17409532 | 207.5 | exon (NM_000525, exon 1 of 1) | 749 | KCNJ11 |
| chr10-7 | chr10 | 5062547 | 5062697 | 207.5 | Intergenic | -2397 | AKR1C2 |
| chr1-8 | chr1 | 207221764 | 207221914 | 207.5 | 3' UTR (NM_018566, exon 2 of 2) | 2583 | YOD1 |
| chr17-7 | chr17 | 79881404 | 79881554 | 207.5 | promoter-TSS (NM_032711) | -35 | MAFG |
| chr4-3 | chr4 | 87744696 | 87744846 | 200.8 | 3' UTR (NM_197965, exon 6 of 6) | 25645 | SLC10A6 |
| chr18-3 | chr18 | 42645664 | 42645814 | 200.8 | 3' UTR (NM_015559, exon 6 of 6) | -95608 | MIR4319 |
| chr7-6 | chr7 | 65306834 | 65306984 | 200.8 | Intergenic | -31348 | VKORC1L1 |
| chr19-9 | chr19 | 41009923 | 41010073 | 194.1 | exon (NM_020971, exon 12 of 36) | -26397 | SPTBN4 |
| chr9-2 | chr9 | 130764116 | 130764266 | 194.1 | Intergenic | -21379 | FAM102A |
| chr21-7 | chr21 | 9825521 | 9825671 | 194.1 | promoter-TSS (NR_037421) | -236 | MIR3648 |
| chr15-4 | chr15 | 43827196 | 43827346 | 194.1 | exon (NM_001130858, exon 31 of 31) | 17465 | MAP1A |
| chr20-3 | chr20 | 36007680 | 36007830 | 194.1 | intron (NM_005417, intron 2 of 13) | 33198 | SRC |
| chr19-10 | chr19 | 56888731 | 56888881 | 194.1 | non-coding (NR_033418, exon 6 of 6) | 9316 | ZNF542 |
| chr2-11 | chr2 | 26220683 | 26220833 | 194.1 | Intergenic | -15315 | KIF3C |
| chr4-4 | chr4 | 39516895 | 39517045 | 194.1 | intron (NM_001184701, intron 1 of 10) | 12248 | UGDH |
| chr17-6 | chr17 | 73269628 | 73269778 | 194.1 | exon (NM_001126121, exon 8 of 8) | 2323 | LOC100287042 |
| chr14-1 | chr14 | 57150041 | 57150191 | 187.4 | Intergenic | 103605 | C14orf101 |
| chr17-8 | chr17 | 12862041 | 12862191 | 187.4 | exon (NM_014859, exon 16 of 21) | 59265 | ELAC2 |
| chr5-6 | chr5 | 98128788 | 98128938 | 187.4 | exon (NM_001012761, exon 5 of 5) | -20075 | FLJ35946 |
| chr19-7 | chr19 | 9362504 | 9362654 | 187.4 | exon (NM_001079935, exon 1 of 1) | 859 | OR7E24 |
| chr12-4 | chr12 | 6691699 | 6691849 | 187.4 | promoter-TSS (NR_003012) | -999 | SCARNA11 |
| chr7-8 | chr7 | 73604185 | 73604335 | 187.4 | intron (NM_031992, intron 4 of 5) | -1268 | MIR590 |
| chr19-8 | chr19 | 15852184 | 15852334 | 187.4 | promoter-TSS (NM_013938) | 56 | OR10H3 |
| chr5-8 | chr5 | 57555727 | 57555877 | 187.4 | Intergenic | 200164 | PLK2 |
| chr5-7 | chr5 | 179653670 | 179653820 | 187.4 | Intergenic | -17615 | RASGEF1C |
| chr22-4 | chr22 | 30442385 | 30442535 | 187.4 | Intergenic | -33993 | HORMAD2 |
| chr14-2 | chr14 | 26750929 | 26751079 | 187.4 | Intergenic | 315956 | NOVA1 |
| chr7-7 | chr7 | 134133530 | 134133680 | 187.4 | intron (NM_001628, intron 5 of 9) | 10283 | AKR1B1 |
| chr2-12 | chr2 | 241327129 | 241327279 | 180.7 | Intergenic | -47911 | GPC1 |
| chr1-11 | chr1 | 10475590 | 10475740 | 180.7 | intron (NM_002631, intron 8 of 12) | -14494 | APITD1-CORT |
| chr12-5 | chr12 | 104613210 | 104613360 | 180.7 | intron (NM_001093771, intron 1 of 16) | 3728 | TXNRD1 |
| chr3-4 | chr3 | 134322858 | 134323008 | 180.7 | exon (NM_178554, exon 11 of 11) | 46931 | KY |
| chr5-10 | chr5 | 175281683 | 175281833 | 174 | intron (NM_006650, intron 2 of 4) | -16743 | CPLX2 |
| chr2-13 | chr2 | 26953825 | 26953975 | 174 | 3' UTR (NM_002246, exon 2 of 2) | -33242 | C2orf18 |
| chr13-1 | chr13 | 27151129 | 27151279 | 174 | intron (NM_006646, intron 1 of 9) | 19364 | WASF3 |
| chr5-9 | chr5 | 159008931 | 159009081 | 174 | Intergenic | -115722 | LOC285627 |
| chr6-7 | chr6 | 27879572 | 27879722 | 174 | exon (NM_033057, exon 1 of 1) | 527 | OR2B2 |
| chr1-12 | chr1 | 156905409 | 156905559 | 174 | TTS (NR_030527) | 552 | MIR765 |
| chr9-3 | chr9 | 89560507 | 89560657 | 174 | 3' UTR (NM_002048, exon 1 of 1) | 1522 | GAS1 |
| chr17-9 | chr17 | 78228663 | 78228813 | 174 | Intergenic | -5922 | RNF213 |
| chr6-8 | chr6 | 129250435 | 129250585 | 174 | intron (NM_001079823, intron 1 of 63) | 46224 | LAMA2 |
| chr14-5 | chr14 | 104516099 | 104516249 | 167.3 | intron (NM_153046, intron 35 of 35) | -35849 | ASPG |
| chr10-11 | chr10 | 4983801 | 4983951 | 167.3 | Intergenic | -21578 | AKR1C1 |
| chr19-16 | chr19 | 48790054 | 48790204 | 167.3 | exon (NM_153608, exon 5 of 5) | 15475 | ZNF114 |
| chr5-11 | chr5 | 140090683 | 140090833 | 167.3 | promoter-TSS (NR_026703) | -103 | VTRNA1-1 |
| chr14-4 | chr14 | 102499778 | 102499928 | 167.3 | intron (NM_001376, intron 54 of 77) | 53659 | HSP90AA1 |
| chr1-13 | chr1 | 226005462 | 226005612 | 167.3 | intron (NM_001136018, intron 1 of 8) | -7465 | EPHX1 |
| chr7-9 | chr7 | 107886878 | 107887028 | 167.3 | intron (NM_001193583, intron 3 of 29) | -6339 | NRCAM |
| chr19-14 | chr19 | 10283763 | 10283913 | 167.3 | exon (NM_001379, exon 8 of 40) | 21917 | DNMT1 |
| chr10-10 | chr10 | 16553213 | 16553363 | 167.3 | 3' UTR (NM_030664, exon 6 of 6) | 10716 | C1QL3 |
| chrX-2 | chrX | 119005055 | 119005205 | 167.3 | promoter-TSS (NM_004541) | -604 | NDUFA1 |
| chr10-12 | chr10 | 86178903 | 86179053 | 160.6 | intron (NM_018999, intron 4 of 10) | 90568 | FAM190B |
| chr2-16 | chr2 | 219558778 | 219558928 | 160.6 | intron (NM_001243313, intron 19 of 26) | -16715 | TTLL4 |
| chr19-11 | chr19 | 12491834 | 12491984 | 160.6 | Intergenic | -15434 | ZNF442 |
| chr7-11 | chr7 | 116105912 | 116106062 | 160.6 | Intergenic | -33668 | CAV2 |
| chr18-6 | chr18 | 11609499 | 11609649 | 160.6 | Intergenic | -79440 | GNAL |
| chr1-10 | chr1 | 63825896 | 63826046 | 160.6 | Intergenic | -7290 | ALG6 |
| chr14-3 | chr14 | 24760261 | 24760411 | 160.6 | intron (NM_138452, intron 8 of 8) | 8330 | DHRS1 |
| chr9-4 | chr9 | 119329370 | 119329520 | 160.6 | intron (NM_198188, intron 4 of 8) | 62883 | LOC100128505 |
| chr17-11 | chr17 | 7483505 | 7483655 | 160.6 | exon (NM_001251, exon 2 of 6) | 774 | CD68 |
| chr2-15 | chr2 | 20491522 | 20491672 | 160.6 | intron (NM_015317, intron 8 of 19) | 35547 | PUM2 |
| chr11-4 | chr11 | 67379824 | 67379974 | 160.6 | exon (NM_007103, exon 10 of 10) | 3236 | DOC2GP |
| chr9-5 | chr9 | 108145273 | 108145423 | 160.6 | intron (NM_080546, intron 13 of 15) | -64967 | FSD1L |
| chr19-13 | chr19 | 619336 | 619486 | 160.6 | intron (NM_005035, intron 13 of 20) | 14157 | POLRMT |
| chr12-7 | chr12 | 123444625 | 123444775 | 153.9 | exon (NM_019624, exon 2 of 11) | 6356 | ABCB9 |
| chr12-6 | chr12 | 50260702 | 50260852 | 153.9 | TTS (NM_012306) | -23865 | BCDIN3D |
| chr1-14 | chr1 | 1848080 | 1848230 | 153.9 | TTS (NM_178545) | 1889 | CALML6 |
| chr20-5 | chr20 | 61512892 | 61513042 | 153.9 | exon (NM_001193369, exon 16 of 16) | -19852 | TCFL5 |
| chr8-4 | chr8 | 134420563 | 134420713 | 153.9 | Intergenic | -111091 | NDRG1 |
| chr10-15 | chr10 | 95161223 | 95161373 | 153.9 | exon (NM_013451, exon 12 of 54) | 80776 | MYOF |
| chr11-6 | chr11 | 13360102 | 13360252 | 153.9 | intron (NM_001178, intron 3 of 19) | 60852 | ARNTL |
| chr7-10 | chr7 | 106089792 | 106089942 | 153.9 | Intergenic | -164229 | NAMPT |
| chr19-19 | chr19 | 12461800 | 12461950 | 153.9 | exon (NM_030824, exon 6 of 6) | 14600 | ZNF442 |
| chr3-5 | chr3 | 14148802 | 14148952 | 153.9 | Intergenic | 17494 | CHCHD4 |
| chr2-14 | chr2 | 168101512 | 168101662 | 153.9 | exon (NM_152381, exon 9 of 11) | 57794 | XIRP2 |
| chr19-18 | chr19 | 49467126 | 49467276 | 153.9 | Intergenic | -1365 | FTL |
| chr11-5 | chr11 | 113775689 | 113775839 | 153.9 | intron (NM_006028, intron 1 of 8) | 246 | HTR3B |
| chr17-13 | chr17 | 80040896 | 80041046 | 147.2 | exon (NM_004104, exon 33 of 43) | 15135 | FASN |
| chr19-20 | chr19 | 43858180 | 43858330 | 147.2 | intron (NM_020406, intron 2 of 8) | 430 | CD177 |
| chr2-18 | chr2 | 26538453 | 26538603 | 147.2 | exon (NM_001145168, exon 6 of 13) | 3389 | GPR113 |
| chr19-17 | chr19 | 2917661 | 2917811 | 147.2 | exon (NM_173480, exon 4 of 4) | 16840 | ZNF57 |
| chr19-21 | chr19 | 34850405 | 34850555 | 147.2 | Intergenic | -5165 | GPI |
| chr17-14 | chr17 | 1620477 | 1620627 | 147.2 | promoter-TSS (NR_028505) | 735 | WDR81 |
| chr3-6 | chr3 | 120397477 | 120397627 | 147.2 | intron (NM_000187, intron 1 of 13) | 3866 | HGD |
| chr8-3 | chr8 | 128772863 | 128773013 | 147.2 | Intergenic | 24623 | MYC |
| chr17-10 | chr17 | 19281193 | 19281343 | 147.2 | promoter-TSS (NM_002749) | 227 | B9D1 |
| chr14-7 | chr14 | 103996474 | 103996624 | 147.2 | exon (NM_152307, exon 2 of 4) | 1040 | TRMT61A |
| chr10-16 | chr10 | 101535713 | 101535863 | 147.2 | Intergenic | -6675 | ABCC2 |
| chr19-22 | chr19 | 45322655 | 45322805 | 147.2 | exon (NM_001013257, exon 12 of 14) | 10414 | BCAM |
| chr9-6 | chr9 | 113682086 | 113682236 | 147.2 | intron (NM_001401, intron 3 of 3) | 118204 | LPAR1 |
| chr11-7 | chr11 | 18753956 | 18754106 | 147.2 | intron (NM_006906, intron 12 of 14) | -6254 | IGSF22 |
| chr14-6 | chr14 | 92600178 | 92600328 | 147.2 | intron (NM_017437, intron 3 of 15) | 11955 | CPSF2 |
| chr1-15 | chr1 | 1477276 | 1477426 | 147.2 | 3' UTR (NM_014188, exon 5 of 5) | -1611 | TMEM240 |
| chr8-5 | chr8 | 62632093 | 62632243 | 147.2 | Intergenic | 4821 | MIR4470 |
| chr17-12 | chr17 | 44178510 | 44178660 | 147.2 | intron (NM_001193466, intron 2 of 14).2 | 91581 | KANSL1 |
| chr3-7 | chr3 | 44777598 | 44777748 | 140.5 | 3' UTR (NM_145044, exon 3 of 3) | 6575 | ZNF501 |
| chr18-7 | chr18 | 57597243 | 57597393 | 140.5 | Intergenic | 30126 | PMAIP1 |
| chr21-8 | chr21 | 43256405 | 43256555 | 140.5 | intron (NM_022115, intron 16 of 30) | 43102 | PRDM15 |
| chr9-7 | chr9 | 96320785 | 96320935 | 140.5 | intron (NM_014612, intron 14 of 17) | -18049 | PHF2 |
| chr16-4 | chr16 | 15973384 | 15973534 | 140.5 | intron (NM_144600, intron 3 of 4) | 8988 | FOPNL |
| chr5-12 | chr5 | 90610189 | 90610339 | 140.5 | Intergenic | -65900 | ARRDC3-AS1 |
| chr16-3 | chr16 | 70929666 | 70929816 | 140.5 | intron (NM_001270974, intron 54 of 85) | -94680 | VAC14 |
| chr7-12 | chr7 | 100286107 | 100286257 | 140.5 | 5' UTR (NM_022574, exon 1 of 24) | 688 | GIGYF1 |
| chr17-15 | chr17 | 60247814 | 60247964 | 140.5 | Intergenic | 105127 | TBC1D3P2 |
| chr3-8 | chr3 | 64619173 | 64619323 | 140.5 | exon (NM_182920, exon 14 of 40) | -51298 | ADAMTS9-AS2 |
| chr2-19 | chr2 | 234465720 | 234465870 | 140.5 | intron (NM_018218, intron 3 of 30) | 8441 | USP40 |
| chr7-13 | chr7 | 537291 | 537441 | 133.9 | 3' UTR (NM_002607, exon 7 of 7) | 22115 | PDGFA |
| chr10-18 | chr10 | 15902527 | 15902677 | 133.9 | promoter-TSS (NM_024948) | -83 | FAM188A |
| chr1-17 | chr1 | 10448205 | 10448355 | 133.9 | Intergenic | -10805 | PGD |
| chr16-5 | chr16 | 33963181 | 33963331 | 133.9 | promoter-TSS (NR_038368) | -753 | LINC00273 |
| chr6-9 | chr6 | 29141430 | 29141580 | 133.9 | exon (NM_030905, exon 1 of 1).5 | 194 | OR2J2 |
| chr19-23 | chr19 | 41285846 | 41285996 | 133.9 | intron (NM_016154, intron 1 of 7) | 1797 | RAB4B-EGLN2 |
| chr9-8 | chr9 | 21591721 | 21591871 | 133.9 | Intergenic | -32099 | MIR31HG |
| chr11-9 | chr11 | 124755078 | 124755228 | 133.9 | intron (NM_019055, intron 16 of 17) | 12678 | ROBO4 |
| chr10-19 | chr10 | 86012433 | 86012583 | 133.9 | intron (NM_002921, intron 3 of 6) | 7699 | RGR |
| chr1-16 | chr1 | 27734415 | 27734565 | 133.9 | 3' UTR (NM_006990, exon 9 of 9) | 15338 | GPR3 |
| chr4-6 | chr4 | 6734431 | 6734581 | 133.9 | Intergenic | 16664 | BLOC1S4 |
| chr7-14 | chr7 | 75567340 | 75567490 | 133.9 | intron (NM_000941, intron 1 of 15) | -5686 | SNORA14A |
| chr3-9 | chr3 | 182597173 | 182597323 | 133.9 | intron (NM_014616, intron 19 of 29) | 85957 | ATP11B |
| chr8-6 | chr8 | 54857565 | 54857715 | 133.9 | intron (NM_170587, intron 3 of 5) | 64198 | RGS20 |
| chr20-6 | chr20 | 30148021 | 30148171 | 133.9 | intron (NM_178580, intron 9 of 12) | 12911 | PSIMCT-1 |
| chr11-11 | chr11 | 6789614 | 6789764 | 133.9 | exon (NM_001004490, exon 1 of 1) | 499 | OR2AG2 |
| chr10-17 | chr10 | 13230835 | 13230985 | 133.9 | exon (NM_182751, exon 10 of 20) | 27356 | MCM10 |
| chr1-19 | chr1 | 204616751 | 204616901 | 133.9 | intron (NM_201630, intron 1 of 1) | 37655 | LRRN2 |
| chr15-5 | chr15 | 77321059 | 77321209 | 127.2 | intron (NM_003978, intron 7 of 14) | 33669 | PSTPIP1 |
| chr2-21 | chr2 | 92269425 | 92269575 | 127.2 | Intergenic | 140341 | ACTR3BP2 |
| chr11-10 | chr11 | 5125677 | 5125827 | 127.2 | Intergenic | 28120 | OR52A5 |
| chr9-9 | chr9 | 114364071 | 114364221 | 127.2 | TTS (NR_034087) | -2011 | PTGR1 |
| chr20-4 | chr20 | 26190054 | 26190204 | 127.2 | promoter-TSS (NR_040095) | -260 | LOC284801 |
| chr11-8 | chr11 | 112039948 | 112040098 | 127.2 | exon (NM_031275, exon 2 of 5) | 1928 | TEX12 |
| chr12-8 | chr12 | 53689561 | 53689711 | 127.2 | exon (NM_002624, exon 2 of 6) | 401 | PFDN5 |
| chr10-24 | chr10 | 123670270 | 123670420 | 127.2 | intron (NM_001001976, intron 5 of 11) | 17201 | ATE1 |
| chr1-22 | chr1 | 202183284 | 202183434 | 127.2 | promoter-TSS (NM_001017404) | 76 | LGR6 |
| chr2-23 | chr2 | 97034821 | 97034971 | 127.2 | intron (NM_015341, intron 16 of 17) | 33412 | NCAPH |
| chr11-12 | chr11 | 33914361 | 33914511 | 127.2 | promoter-TSS (NM_005574) | -600 | LMO2 |
| chr15-7 | chr15 | 63128149 | 63128299 | 127.2 | exon (NM_015059, exon 54 of 56) | 12068 | MIR190A |
| chr17-16 | chr17 | 79826771 | 79826921 | 127.2 | exon (NM_001185077, exon 6 of 6) | 1944 | ARHGDIA |
| chr9-10 | chr9 | 137299207 | 137299357 | 127.2 | intron (NM_002957, intron 2 of 9) | 28025 | MIR4669 |
| chr22-5 | chr22 | 18032721 | 18032871 | 127.2 | 3' UTR (NM_031413, exon 18 of 18) | -10387 | SLC25A18 |
| chr7-15 | chr7 | 70255184 | 70255334 | 127.2 | exon (NM_001127231, exon 18 of 18) | -342530 | WBSCR17 |
| chr7-16 | chr7 | 92439123 | 92439273 | 127.2 | intron (NM_001259, intron 2 of 7) | 24033 | CDK6 |
| chr19-24 | chr19 | 56832714 | 56832864 | 127.2 | Intergenic | -46679 | ZNF542 |
| chr15-6 | chr15 | 44962004 | 44962154 | 127.2 | intron (NM_001145112, intron 8 of 15) | -6203 | SPG11 |
| chr1-21 | chr1 | 193155068 | 193155218 | 127.2 | intron (NM_003783, intron 1 of 1) | 600 | B3GALT2 |
| chr10-21 | chr10 | 5114039 | 5114189 | 127.2 | intron (NM_001253908, intron 1 of 8) | -22454 | AKR1C3 |
| chr17-17 | chr17 | 3101268 | 3101418 | 127.2 | exon (NM_012352, exon 1 of 1) | 530 | OR1A2 |
| chr20-7 | chr20 | 10492281 | 10492431 | 127.2 | intron (NM_001009608, intron 2 of 7) | 76405 | C20orf94 |
| chr14-9 | chr14 | 102414422 | 102414572 | 127.2 | Intergenic | -16368 | DYNC1H1 |
| chr2-20 | chr2 | 74884636 | 74884786 | 127.2 | exon (NM_004263, exon 3 of 14) | 3318 | SEMA4F |
| chr10-23 | chr10 | 94968316 | 94968466 | 120.5 | Intergenic | 134744 | CYP26A1 |
| chr3-10 | chr3 | 17680835 | 17680985 | 120.5 | intron (NM_014744, intron 1 of 21) | 60602 | TBC1D5 |
| chr4-7 | chr4 | 103650241 | 103650391 | 120.5 | intron (NM_005908, intron 1 of 16) | 31835 | MANBA |
| chr14-8 | chr14 | 25043497 | 25043647 | 120.5 | exon (NM_001911, exon 4 of 5) | 1894 | CTSG |
| chr10-26 | chr10 | 56360593 | 56360743 | 120.5 | intron (NM_001142771, intron 2 of 34) | 200383 | PCDH15 |
| chr1-24 | chr1 | 23724524 | 23724674 | 120.5 | intron (NM_003196, intron 5 of 10) | 26662 | TCEA3 |
| chr5-15 | chr5 | 179246515 | 179246665 | 120.5 | intron (NM_001142299, intron 2 of 8) | -1252 | SQSTM1 |
| chr8-9 | chr8 | 62679788 | 62679938 | 120.5 | Intergenic | 52516 | MIR4470 |
| chr8-7 | chr8 | 102388385 | 102388535 | 120.5 | Intergenic | 7339 | NACAP1 |
| chr5-17 | chr5 | 122523022 | 122523172 | 120.5 | 3' UTR (NM_001136239, exon 8 of 8) | 98256 | PRDM6 |
| chr11-13 | chr11 | 49455501 | 49455651 | 120.5 | Intergenic | -124504 | LOC440040 |
| chr12-10 | chr12 | 113590538 | 113590688 | 120.5 | exon (NM_001144872, exon 3 of 8) | 2950 | CCDC42B |
| chr15-10 | chr15 | 81614554 | 81614704 | 120.5 | intron (NM_181900, intron 3 of 5) | 1895 | STARD5 |
| chr22-6 | chr22 | 35768004 | 35768154 | 120.5 | Intergenic | -8981 | HMOX1 |
| chr16-8 | chr16 | 87872420 | 87872570 | 120.5 | intron (NM_003486, intron 5 of 9) | 30605 | SLC7A5 |
| chr5-16 | chr5 | 65020366 | 65020516 | 120.5 | intron (NM_020726, intron 1 of 12) | 2418 | NLN |
| chr1-26 | chr1 | 170338950 | 170339100 | 120.5 | Intergenic | -85676 | LOC284688 |
| chr5-13 | chr5 | 154199700 | 154199850 | 120.5 | 3' UTR (NM_032385, exon 9 of 9) | 30438 | C5orf4 |
| chr19-25 | chr19 | 53958820 | 53958970 | 120.5 | exon (NM_001008401, exon 7 of 7) | -12094 | ZNF813 |
| chr16-7 | chr16 | 81396258 | 81396408 | 120.5 | intron (NM_022041, intron 6 of 10) | -22290 | MIR4720 |
| chr11-15 | chr11 | 5058294 | 5058444 | 120.5 | Intergenic | -9387 | OR52J3 |
| chr1-18 | chr1 | 45987619 | 45987769 | 120.5 | promoter-TSS (NM_181697) | -84 | PRDX1 |
| chr22-8 | chr22 | 30592186 | 30592336 | 120.5 | Intergenic | 50579 | LIF |
| chr2-24 | chr2 | 143630997 | 143631147 | 120.5 | Intergenic | -4123 | KYNU |
| chr1-20 | chr1 | 150917404 | 150917554 | 120.5 | exon (NM_001145415, exon 9 of 22) | 18664 | SETDB1 |
| chr11-14 | chr11 | 1103572 | 1103722 | 120.5 | intron (NM_002457, intron 48 of 49) | 28772 | MUC2 |
| chr9-11 | chr9 | 125281911 | 125282061 | 120.5 | exon (NM_001004452, exon 1 of 1) | 566 | OR1J4 |
| chr5-19 | chr5 | 96478188 | 96478338 | 113.8 | exon (NM_153234, exon 1 of 6) | 257 | LIX1 |
| chr1-28 | chr1 | 167814796 | 167814946 | 113.8 | exon (NM_001167749, exon 18 of 30) | 68582 | ADCY10 |
| chr8-8 | chr8 | 30585376 | 30585526 | 113.8 | promoter-TSS (NM_001195104) | 35 | GSR |
| chr7-17 | chr7 | 28077013 | 28077163 | 113.8 | intron (NM_175061, intron 1 of 4) | -142988 | JAZF1-AS1 |
| chr9-12 | chr9 | 97699857 | 97700007 | 113.8 | intron (NM_001193329, intron 6 of 15) | 127688 | MIR2278 |
| chr15-8 | chr15 | 42042539 | 42042689 | 113.8 | exon (NM_001080541, exon 16 of 23) | -24018 | MAPKBP1 |
| chr11-17 | chr11 | 94646535 | 94646685 | 113.8 | Intergenic | 60166 | CWC15 |
| chr10-30 | chr10 | 94581811 | 94581961 | 113.8 | Intergenic | -12584 | EXOC6 |
| chr1-27 | chr1 | 201187473 | 201187623 | 113.8 | intron (NM_001164586, intron 17 of 23) | 27595 | IGFN1 |
| chr3-11 | chr3 | 53270286 | 53270436 | 113.8 | intron (NM_001064, intron 4 of 13) | 19769 | TKT |
| chr10-31 | chr10 | 105797407 | 105797557 | 113.8 | exon (NM_000494, exon 46 of 56) | 10462 | MIR936 |
| chr12-14 | chr12 | 113515431 | 113515581 | 113.8 | exon (NM_004416, exon 2 of 9) | 19844 | DTX1 |
| chr8-10 | chr8 | 104897850 | 104898000 | 113.8 | exon (NM_014677, exon 2 of 22) | 66509 | RIMS2 |
| chr12-13 | chr12 | 64265712 | 64265862 | 113.8 | intron (NM_020762, intron 1 of 21) | 27246 | SRGAP1 |
| chrX-4 | chrX | 47098614 | 47098764 | 113.8 | intron (NM_004651, intron 2 of 20) | 6375 | USP11 |
| chr11-16 | chr11 | 5067740 | 5067890 | 113.8 | promoter-TSS (NM_001001916) | 59 | OR52J3 |
| chr1-23 | chr1 | 8940177 | 8940327 | 113.8 | TTS (NR_038351) | -1101 | ENO1 |
| chr14-12 | chr14 | 55819445 | 55819595 | 113.8 | 3' UTR (NM_017943, exon 2 of 2) | 59056 | ATG14 |
| chr1-30 | chr1 | 236686849 | 236686999 | 113.8 | promoter-TSS (NM_201544) | -103 | LGALS8 |
| chr12-9 | chr12 | 6539840 | 6539990 | 113.8 | Intergenic | -14136 | CD27 |
| chr20-8 | chr20 | 33805647 | 33805797 | 113.8 | Intergenic | -8817 | MMP24 |
| chr14-13 | chr14 | 64891635 | 64891785 | 113.8 | intron (NM_005956, intron 9 of 27) | 36951 | MTHFD1 |
| chr1-25 | chr1 | 94374938 | 94375088 | 113.8 | promoter-TSS (NM_002061) | -1 | GCLM |
| chrX-5 | chrX | 61839232 | 61839382 | 113.8 | Intergenic | 731911 | SPIN4 |
| chr13-2 | chr13 | 108861302 | 108861452 | 113.8 | exon (NM_206937, exon 3 of 3) | 5753 | LIG4 |
| chr15-9 | chr15 | 52161289 | 52161439 | 113.8 | intron (NM_014547, intron 2 of 9) | 39539 | TMOD3 |
| chr16-6 | chr16 | 15979623 | 15979773 | 113.8 | intron (NM_144600, intron 1 of 4) | 2749 | FOPNL |
| chr5-20 | chr5 | 140098347 | 140098497 | 113.8 | promoter-TSS (NR_026704) | -89 | VTRNA1-2 |
| chr16-11 | chr16 | 76980897 | 76981047 | 107.1 | Intergenic | 78139 | MIR4719 |
| chr4-10 | chr4 | 6107325 | 6107475 | 107.1 | exon (NM_001099433, exon 3 of 21) | 94918 | JAKMIP1 |
| chr14-14 | chr14 | 79269879 | 79270029 | 107.1 | intron (NM_004796, intron 5 of 16) | 399861 | NRXN3 |
| chr10-35 | chr10 | 73620300 | 73620450 | 107.1 | Intergenic | -9293 | PSAP |
| chr7-19 | chr7 | 1062632 | 1062782 | 107.1 | promoter-TSS (NR_029898) | -45 | MIR339 |
| chr11-20 | chr11 | 12705508 | 12705658 | 107.1 | intron (NM_021961, intron 2 of 12) | 9614 | TEAD1 |
| chr20-10 | chr20 | 345660 | 345810 | 107.1 | Intergenic | -15573 | TRIB3 |
| chr16-9 | chr16 | 16115992 | 16116142 | 107.1 | intron (NM_004996, intron 5 of 30) | 72633 | ABCC1 |
| chr7-21 | chr7 | 134212073 | 134212223 | 107.1 | promoter-TSS (NM_020299) | -196 | AKR1B10 |
| chr15-11 | chr15 | 26860663 | 26860813 | 107.1 | intron (NM_021912, intron 4 of 8) | 13499 | GABRB3 |
| chr17-21 | chr17 | 44026475 | 44026625 | 107.1 | intron (NM_016835, intron 1 of 13).2 | -50066 | STH |
| chr22-9 | chr22 | 24122751 | 24122901 | 107.1 | exon (NM_005940, exon 4 of 8) | -6324 | SMARCB1 |
| chr18-11 | chr18 | 3652955 | 3653105 | 107.1 | intron (NM_001003809, intron 4 of 9) | 58918 | DLGAP1-AS1 |
| chr2-30 | chr2 | 201502897 | 201503047 | 107.1 | exon (NM_001159, exon 23 of 35) | 52241 | AOX1 |
| chr12-20 | chr12 | 92472080 | 92472230 | 107.1 | intron (NR_046160, intron 1 of 5) | 63334 | LOC256021 |
| chr5-23 | chr5 | 72139488 | 72139638 | 107.1 | intron (NM_002270, intron 1 of 24) | -4367 | TNPO1 |
| chr17-18 | chr17 | 19963159 | 19963309 | 107.1 | intron (NM_001243439, intron 1 of 14) | -27101 | SPECC1 |
| chr1-32 | chr1 | 21965176 | 21965326 | 107.1 | intron (NM_002885, intron 2 of 24) | 13097 | RAP1GAP |
| chr13-3 | chr13 | 52413890 | 52414040 | 107.1 | intron (NR_027047, intron 1 of 4) | 5321 | LINC00282 |
| chr16-12 | chr16 | 84766618 | 84766768 | 107.1 | intron (NM_005153, intron 1 of 13) | 33138 | USP10 |
| chr6-11 | chr6 | 12492643 | 12492793 | 107.1 | Intergenic | -100132 | RNU6-48 |
| chr8-11 | chr8 | 128866872 | 128867022 | 107.1 | Intergenic | -35927 | PVT1 |
| chr14-16 | chr14 | 69403456 | 69403606 | 107.1 | intron (NM_001130004, intron 1 of 21) | 42552 | ACTN1 |
| chr12-15 | chr12 | 125399799 | 125399949 | 107.1 | promoter-TSS (NM_021009) | -219 | MIR5188 |
| chr14-11 | chr14 | 55765739 | 55765889 | 107.1 | intron (NM_152231, intron 1 of 1) | 26942 | FBXO34 |
| chr9-14 | chr9 | 111624826 | 111624976 | 107.1 | exon (NM_006687, exon 1 of 1) | 298 | ACTL7A |
| chr10-27 | chr10 | 5121166 | 5121316 | 107.1 | intron (NM_001253908, intron 1 of 8) | -15327 | AKR1C3 |
| chr2-25 | chr2 | 11729062 | 11729212 | 107.1 | TTS (NM_148903) | 46286 | GREB1 |
| chr10-36 | chr10 | 133879393 | 133879543 | 107.1 | Intergenic | -38845 | JAKMIP3 |
| chr1-34 | chr1 | 78469074 | 78469224 | 107.1 | Intergenic | -1487 | DNAJB4 |
| chr1-31 | chr1 | 247463832 | 247463982 | 107.1 | exon (NM_032752, exon 9 of 9) | 31138 | ZNF496 |
| chr12-17 | chr12 | 16502026 | 16502176 | 107.1 | intron (NR_048547, intron 1 of 2) | 1389 | MGST1 |
| chr21-9 | chr21 | 43704678 | 43704828 | 107.1 | exon (NM_016818, exon 7 of 15) | 30953 | TFF3 |
| chr18-9 | chr18 | 3624456 | 3624606 | 107.1 | intron (NM_001003809, intron 4 of 9) | 30419 | DLGAP1-AS1 |
| chr7-20 | chr7 | 34892153 | 34892303 | 107.1 | intron (NM_207173, intron 8 of 8) | -18285 | NPSR1-AS1 |
| chr11-19 | chr11 | 14278122 | 14278272 | 107.1 | exon (NM_006108, exon 10 of 16) | 101834 | RRAS2 |
| chr5-18 | chr5 | 39756120 | 39756270 | 107.1 | Intergenic | -330860 | DAB2 |
| chr10-25 | chr10 | 22331610 | 22331760 | 107.1 | Intergenic | -39035 | DNAJC1 |
| chr8-12 | chr8 | 40230971 | 40231121 | 107.1 | Intergenic | 220059 | C8orf4 |
| chr2-26 | chr2 | 29026072 | 29026222 | 107.1 | TTS (NM_206876) | -7553 | SPDYA |
| chr10-37 | chr10 | 135054600 | 135054750 | 107.1 | 3' UTR (NM_014468, exon 3 of 3) | 3267 | VENTX |
| chr4-8 | chr4 | 763788 | 763938 | 107.1 | 3' UTR (NM_006315, exon 11 of 11) | 11773 | LOC100129917 |
| chr9-16 | chr9 | 114341045 | 114341195 | 107.1 | TTS (NM_001007169) | 20591 | PTGR1 |
| chr19-30 | chr19 | 54407834 | 54407984 | 107.1 | exon (NM_002739, exon 16 of 18) | -8082 | CACNG7 |
| chr11-18 | chr11 | 1793882 | 1794032 | 107.1 | Intergenic | -8456 | MOB2 |
| chr20-11 | chr20 | 36766693 | 36766843 | 107.1 | exon (NM_198951, exon 10 of 10) | 26932 | TGM2 |
| chr2-27 | chr2 | 75466196 | 75466346 | 107.1 | Intergenic | -39626 | TACR1 |
| chr12-12 | chr12 | 53587526 | 53587676 | 107.1 | exon (NM_000889, exon 11 of 16) | -12908 | CSAD |
| chr1-29 | chr1 | 233249239 | 233249389 | 100.4 | intron (NM_014801, intron 21 of 33) | 162944 | NTPCR |
| chr8-14 | chr8 | 55435355 | 55435505 | 100.4 | Intergenic | 64935 | SOX17 |
| chr20-14 | chr20 | 32378752 | 32378902 | 100.4 | exon (NM_032819, exon 15 of 15) | -20283 | CHMP4B |
| chr19-31 | chr19 | 4793856 | 4794006 | 100.4 | 3' UTR (NM_018708, exon 1 of 1) | 2203 | FEM1A |
| chr12-19 | chr12 | 53194715 | 53194865 | 100.4 | Intergenic | -4898 | KRT3 |
| chr10-39 | chr10 | 95226232 | 95226382 | 100.4 | intron (NM_133337, intron 1 of 52) | 15767 | MYOF |
| chr10-34 | chr10 | 49634403 | 49634553 | 100.4 | exon (NM_002750, exon 8 of 11) | 24791 | MAPK8 |
| chr17-25 | chr17 | 74621214 | 74621364 | 100.4 | 3' UTR (NM_018414, exon 9 of 9) | 18605 | ST6GALNAC1 |
| chr3-15 | chr3 | 138310668 | 138310818 | 100.4 | 5' UTR (NM_024491, exon 2 of 18) | 2386 | CEP70 |
| chr6-12 | chr6 | 163784246 | 163784396 | 100.4 | Intergenic | -16256 | DKFZp451B082 |
| chr2-28 | chr2 | 92275872 | 92276022 | 100.4 | Intergenic | 146788 | ACTR3BP2 |
| chr14-10 | chr14 | 45605497 | 45605647 | 100.4 | exon (NM_020937, exon 1 of 23) | 436 | FANCM |
| chr9-17 | chr9 | 75473253 | 75473403 | 100.4 | Intergenic | 94905 | ALDH1A1 |
| chr20-13 | chr20 | 48909219 | 48909369 | 100.4 | promoter-TSS (NR_034124) | 37 | LOC284751 |
| chr14-15 | chr14 | 21623436 | 21623586 | 100.4 | exon (NM_001004731, exon 1 of 1) | 673 | OR5AU1 |
| chr17-24 | chr17 | 64463177 | 64463327 | 100.4 | intron (NM_002737, intron 2 of 16) | 164326 | PRKCA |
| chr1-33 | chr1 | 27440331 | 27440481 | 100.4 | exon (NM_003047, exon 2 of 12) | 41215 | SLC9A1 |
| chr22-10 | chr22 | 41244111 | 41244261 | 100.4 | intron (NM_003932, intron 3 of 11) | 8501 | ST13 |
| chr3-12 | chr3 | 48730921 | 48731071 | 100.4 | non-coding (NR_027438, exon 4 of 4) | -7662 | NCKIPSD |
| chr12-18 | chr12 | 49194268 | 49194418 | 100.4 | Intergenic | -11523 | ADCY6 |
| chr7-18 | chr7 | 114659651 | 114659801 | 100.4 | 3' UTR (NM_199072, exon 5 of 5) | 97517 | MDFIC |
| chr1-36 | chr1 | 150131588 | 150131738 | 100.4 | exon (NM_016274, exon 6 of 6) | 9493 | PLEKHO1 |
| chr21-10 | chr21 | 44482828 | 44482978 | 100.4 | intron (NM_001178009, intron 10 of 17) | 13137 | CBS |
| chr5-22 | chr5 | 58357295 | 58357445 | 100.4 | intron (NM_001104631, intron 5 of 14) | -22031 | PDE4D |
| chr17-23 | chr17 | 58547159 | 58547309 | 100.4 | intron (NM_006380, intron 4 of 12) | 47369 | C17orf64 |
| chr2-36 | chr2 | 233272501 | 233272651 | 100.4 | exon (NM_031313, exon 5 of 11) | 1024 | ALPPL2 |
| chr20-12 | chr20 | 26189007 | 26189157 | 100.4 | promoter-TSS (NR_030386) | -168 | MIR663A |
| chrY-2 | chrY | 10037771 | 10037921 | 100.4 | Intergenic | 289439 | TTTY23 |
| chr16-13 | chr16 | 89392550 | 89392700 | 100.4 | intron (NM_001256182, intron 3 of 13) | 5084 | LOC100287036 |
| chr6-14 | chr6 | 119558525 | 119558675 | 100.4 | intron (NM_005907, intron 6 of 12) | -88242 | FAM184A |
| chr1-38 | chr1 | 113456366 | 113456516 | 100.4 | 3' UTR (NM_003051, exon 5 of 5) | -9531 | AKR7A2P1 |
| chr3-13 | chr3 | 52522073 | 52522223 | 100.4 | exon (NM_007184, exon 16 of 21) | -7208 | STAB1 |
| chr2-33 | chr2 | 26291563 | 26291713 | 100.4 | intron (NM_016131, intron 1 of 5) | 34909 | RAB10 |
| chr17-22 | chr17 | 77469707 | 77469857 | 100.4 | intron (NM_001082575, intron 1 of 13) | 8781 | RBFOX3 |
| chr5-21 | chr5 | 39100947 | 39101097 | 100.4 | Intergenic | -26521 | RICTOR |
| chr5-25 | chr5 | 32760269 | 32760419 | 100.4 | intron (NM_001204375, intron 3 of 7) | 48906 | NPR3 |
| chr2-32 | chr2 | 19986257 | 19986407 | 100.4 | Intergenic | -82283 | FLJ12334 |
| chr13-5 | chr13 | 50567929 | 50568079 | 100.4 | non-coding (NR_002612, exon 9 of 11) | 2633 | MIR3613 |
| chr20-9 | chr20 | 24615498 | 24615648 | 100.4 | intron (NM_024893, intron 3 of 3) | 165738 | SYNDIG1 |
| chr8-13 | chr8 | 29198998 | 29199148 | 100.4 | intron (NM_001394, intron 1 of 3) | 7249 | DUSP4 |
| chr11-26 | chr11 | 122051133 | 122051283 | 93.7 | intron (NR_024430, intron 1 of 3) | 22562 | MIR100HG |
| chr9-20 | chr9 | 131930433 | 131930583 | 93.7 | Intergenic | 10032 | IER5L |
| chr13-4 | chr13 | 45969815 | 45969965 | 93.7 | 3' UTR (NM_001010875, exon 10 of 10) | 22626 | SLC25A30 |
| chr22-11 | chr22 | 35782939 | 35783089 | 93.7 | exon (NM_002133, exon 3 of 5) | 5954 | HMOX1 |
| chr12-26 | chr12 | 58704658 | 58704808 | 93.7 | Intergenic | 369288 | XRCC6BP1 |
| chr6-16 | chr6 | 88433093 | 88433243 | 93.7 | Intergenic | -21183 | AKIRIN2 |
| chr16-15 | chr16 | 50335141 | 50335291 | 93.7 | intron (NM_001114, intron 9 of 24) | 13393 | ADCY7 |
| chr8-15 | chr8 | 21608273 | 21608423 | 93.7 | exon (NM_001165038, exon 3 of 8) | 37998 | GFRA2 |
| chr19-32 | chr19 | 5173192 | 5173342 | 93.7 | Intergenic | 167547 | PTPRS |
| chr7-22 | chr7 | 64312742 | 64312892 | 93.7 | Intergenic | -50803 | ZNF273 |
| chr1-37 | chr1 | 6530346 | 6530496 | 93.7 | intron (NM_001042664, intron 15 of 20) | -4166 | TNFRSF25 |
| chr6-15 | chr6 | 33807893 | 33808043 | 93.7 | Intergenic | -36175 | MLN |
| chr11-21 | chr11 | 67141948 | 67142098 | 93.7 | promoter-TSS (NM_001166212) | -375 | CLCF1 |
| chr15-13 | chr15 | 22332770 | 22332920 | 93.7 | intron (NR_015416, intron 5 of 10) | -35633 | OR4M2 |
| chr15-15 | chr15 | 33494118 | 33494268 | 93.7 | Intergenic | 45563 | TMCO5B |
| chr9-15 | chr9 | 88924511 | 88924661 | 93.7 | intron (NM_001185074, intron 12 of 19) | -27096 | ISCA1 |
| chr2-38 | chr2 | 102680818 | 102680968 | 93.7 | Intergenic | 65469 | IL1R2 |
| chr12-27 | chr12 | 133362767 | 133362917 | 93.7 | intron (NM_001172557, intron 15 of 15) | -24391 | ANKLE2 |
| chr7-23 | chr7 | 107852014 | 107852164 | 93.7 | intron (NM_001193583, intron 10 of 29) | 28525 | NRCAM |
| chr7-25 | chr7 | 150649927 | 150650077 | 93.7 | intron (NM_172056, intron 5 of 8) | 2915 | KCNH2 |
| chr10-32 | chr10 | 21914177 | 21914327 | 93.7 | intron (NM_001195626, intron 7 of 21) | 90678 | MLLT10 |
| chr12-23 | chr12 | 133502519 | 133502669 | 93.7 | exon (NM_183238, exon 5 of 5) | 30274 | ZNF605 |
| chr2-35 | chr2 | 120358720 | 120358870 | 93.7 | exon (NM_001271049, exon 8 of 24) | 56787 | PCDP1 |
| chr12-16 | chr12 | 7000122 | 7000272 | 93.7 | Intergenic | 6351 | DSTNP2 |
| chr15-12 | chr15 | 99440380 | 99440530 | 93.7 | intron (NM_000875, intron 4 of 20) | 108430 | PGPEP1L |
| chr6-13 | chr6 | 116355621 | 116355771 | 93.7 | intron (NM_002031, intron 1 of 7) | -4198 | TPI1P3 |
| chr5-24 | chr5 | 159743078 | 159743228 | 93.7 | Intergenic | -3580 | CCNJL |
| chr10-38 | chr10 | 89726888 | 89727038 | 93.7 | 3' UTR (NM_000314, exon 9 of 9) | 103768 | PTEN |
| chr12-24 | chr12 | 34372568 | 34372718 | 93.7 | Intergenic | 197427 | ALG10 |
| chr22-12 | chr22 | 39145720 | 39145870 | 93.7 | exon (NM_015374, exon 6 of 18) | 5672 | SUN2 |
| chr3-14 | chr3 | 129574786 | 129574936 | 93.7 | intron (NM_001128224, intron 1 of 5) | 24541 | TMCC1 |
| chr19-35 | chr19 | 12429395 | 12429545 | 87 | exon (NM_145276, exon 4 of 4) | 15064 | ZNF563 |
| chr12-25 | chr12 | 48169379 | 48169529 | 87 | intron (NM_017842, intron 1 of 2) | 2487 | SLC48A1 |
| chrX-6 | chrX | 24102776 | 24102926 | 87 | Intergenic | 29786 | EIF2S3 |
| chr15-17 | chr15 | 35044929 | 35045079 | 87 | exon (NM_020660, exon 2 of 2) | 1778 | GJD2 |
| chrX-11 | chrX | 123511346 | 123511496 | 87 | 3' UTR (NM_014253, exon 31 of 31) | 31289 | SH2D1A |
| chr15-20 | chr15 | 64975519 | 64975669 | 87 | 3' UTR (NM_015042, exon 9 of 9) | 19868 | OAZ2 |
| chr10-43 | chr10 | 299331 | 299481 | 87 | 3' UTR (NM_006624, exon 15 of 15) | 73472 | ZMYND11 |
| chr7-27 | chr7 | 143093315 | 143093465 | 87 | intron (NM_005232, intron 11 of 17) | -11516 | EPHA1-AS1 |
| chr13-6 | chr13 | 25670564 | 25670714 | 87 | exon (NM_030979, exon 1 of 1) | 363 | PABPC3 |
| chr10-54 | chr10 | 123298223 | 123298373 | 87 | intron (NM_023029, intron 3 of 15) | 55183 | FGFR2 |
| chr11-24 | chr11 | 86198209 | 86198359 | 87 | intron (NM_001014811, intron 6 of 13) | 112506 | CCDC81 |
| chr4-14 | chr4 | 178229459 | 178229609 | 87 | Intergenic | -1457 | NEIL3 |
| chr5-29 | chr5 | 139494433 | 139494583 | 87 | exon (NM_005859, exon 1 of 1) | 800 | PURA |
| chrX-7 | chrX | 30301656 | 30301806 | 87 | Intergenic | 25764 | NR0B1 |
| chr18-15 | chr18 | 19345853 | 19346003 | 87 | intron (NM_020774, intron 2 of 20) | 24383 | MIB1 |
| chr5-28 | chr5 | 72137695 | 72137845 | 87 | intron (NM_002270, intron 1 of 24) | -6160 | TNPO1 |
| chr1-42 | chr1 | 210440264 | 210440414 | 87 | Intergenic | -32873 | SERTAD4-AS1 |
| chr12-32 | chr12 | 121517499 | 121517649 | 87 | Intergenic | -40529 | OASL |
| chr2-39 | chr2 | 112456537 | 112456687 | 87 | Intergenic | 185129 | ANAPC1 |
| chr10-52 | chr10 | 114663990 | 114664140 | 87 | Intergenic | -45944 | TCF7L2 |
| chr9-19 | chr9 | 75076040 | 75076190 | 87 | Intergenic | -60602 | TMC1 |
| chr15-16 | chr15 | 39420211 | 39420361 | 87 | Intergenic | -122599 | C15orf54 |
| chr9-18 | chr9 | 71691210 | 71691360 | 87 | 3' UTR (NM_000144, exon 5 of 5) | 40806 | FXN |
| chr10-40 | chr10 | 5333723 | 5333873 | 87 | Intergenic | -73178 | UCN3 |
| chr2-50 | chr2 | 220082562 | 220082712 | 87 | intron (NM_005689, intron 1 of 18) | 1075 | ABCB6 |
| chr15-19 | chr15 | 63664651 | 63664801 | 87 | intron (NM_206925, intron 2 of 9) | 9349 | CA12 |
| chr17-27 | chr17 | 56028261 | 56028411 | 87 | Intergenic | 37279 | VEZF1 |
| chr12-22 | chr12 | 127568032 | 127568182 | 87 | Intergenic | -208871 | LOC440117 |
| chr12-31 | chr12 | 105065620 | 105065770 | 87 | intron (NM_018413, intron 2 of 2) | 80284 | MIR3922 |
| chr5-27 | chr5 | 180560732 | 180560882 | 87 | Intergenic | -8503 | OR2V1 |
| chr21-11 | chr21 | 45894231 | 45894381 | 87 | Intergenic | 18913 | LRRC3 |
| chr3-17 | chr3 | 120026832 | 120026982 | 87 | Intergenic | 41279 | LRRC58 |
| chr16-17 | chr16 | 48595431 | 48595581 | 87 | exon (NM_153029, exon 2 of 7) | 48614 | N4BP1 |
| chr19-33 | chr19 | 40415542 | 40415692 | 87 | intron (NM_003890, intron 6 of 35) | 24916 | FCGBP |
| chrX-10 | chrX | 69674998 | 69675148 | 87 | 5' UTR (NM_001166278, exon 1 of 12) | 127 | DLG3 |
| chr10-51 | chr10 | 101543866 | 101544016 | 87 | intron (NM_000392, intron 1 of 31) | 1478 | ABCC2 |
| chr14-17 | chr14 | 61570530 | 61570680 | 87 | Intergenic | 122640 | SLC38A6 |
| chr5-31 | chr5 | 133996766 | 133996916 | 87 | exon (NM_001252231, exon 2 of 13) | 12366 | SEC24A |
| chr5-26 | chr5 | 140767964 | 140768114 | 87 | exon (NM_032098, exon 1 of 1) | 587 | PCDHGB4 |
| chr15-18 | chr15 | 55933315 | 55933465 | 87 | exon (NM_173814, exon 12 of 20) | -52340 | PYGO1 |
| chr1-44 | chr1 | 21348920 | 21349070 | 87 | intron (NM_001198803, intron 6 of 14) | 88881 | EIF4G3 |
| chr9-22 | chr9 | 33619680 | 33619830 | 87 | Intergenic | -4468 | ANXA2P2 |
| chr2-40 | chr2 | 151023050 | 151023200 | 87 | Intergenic | 321084 | RND3 |
| chr12-21 | chr12 | 108089709 | 108089859 | 87 | intron (NM_007062, intron 5 of 14) | 10194 | PWP1 |
| chr1-45 | chr1 | 37947359 | 37947509 | 87 | exon (NM_025079, exon 4 of 6) | 7315 | ZC3H12A |
| chr10-41 | chr10 | 27692020 | 27692170 | 87 | intron (NM_001034842, intron 3 of 3) | 11202 | PTCHD3 |
| chr11-28 | chr11 | 125828163 | 125828313 | 87 | 3' UTR (NM_001243597, exon 20 of 20) | 53977 | DDX25 |
| chr15-22 | chr15 | 100332786 | 100332936 | 87 | non-coding (NR_003260, exon 5 of 5) | 14271 | DNM1P46 |
| chr6-19 | chr6 | 41554932 | 41555082 | 87 | intron (NM_138457, intron 6 of 16) | -11454 | MIR4641 |
| chr15-14 | chr15 | 33142612 | 33142762 | 87 | intron (NM_001103184, intron 13 of 16) | 132482 | GREM1 |
| chr8-21 | chr8 | 133358589 | 133358739 | 87 | intron (NM_004519, intron 1 of 14) | 100845 | KCNQ3 |
| chr12-33 | chr12 | 123611231 | 123611381 | 87 | Intergenic | -16331 | PITPNM2 |
| chr17-29 | chr17 | 74442663 | 74442813 | 87 | intron (NM_022066, intron 1 of 17) | 6550 | UBE2O |
| chr20-15 | chr20 | 14860197 | 14860347 | 87 | intron (NM_080676, intron 5 of 16) | 49870 | MACROD2-AS1 |
| chr10-42 | chr10 | 98129680 | 98129830 | 87 | intron (NM_012465, intron 20 of 20) | -10663 | OPALIN |
| chr8-17 | chr8 | 129567058 | 129567208 | 87 | Intergenic | 404771 | MIR1208 |
| chr19-34 | chr19 | 59056760 | 59056910 | 87 | exon (NM_005762, exon 2 of 17) | 999 | TRIM28 |
| chrY-3 | chrY | 13458373 | 13458523 | 87 | Intergenic | 1074941 | GYG2P1 |
| chr2-44 | chr2 | 61997953 | 61998103 | 87 | Intergenic | 83250 | FAM161A |
| chr1-41 | chr1 | 31643757 | 31643907 | 87 | Intergenic | 68902 | NKAIN1 |
| chr1-39 | chr1 | 234505625 | 234505775 | 87 | Intergenic | -3514 | C1orf31 |
| chr19-42 | chr19 | 40970416 | 40970566 | 80.3 | intron (NM_000713, intron 1 of 4) | 1234 | BLVRB |
| chrX-14 | chrX | 142795157 | 142795307 | 80.3 | exon (NM_001009615, exon 2 of 2) | 9284 | SPANXN2 |
| chr17-28 | chr17 | 71794583 | 71794733 | 80.3 | intron (NR_040020, intron 5 of 6) | 11697 | LOC400620 |
| chr14-19 | chr14 | 51832777 | 51832927 | 80.3 | TTS (NR_038358) | 32741 | LINC00640 |
| chr16-18 | chr16 | 89347861 | 89348011 | 80.3 | exon (NM_013275, exon 9 of 13) | -39605 | LOC100287036 |
| chr12-36 | chr12 | 74437223 | 74437373 | 80.3 | Intergenic | 249113 | LOC100507377 |
| chr11-32 | chr11 | 35601349 | 35601499 | 80.3 | Intergenic | -38311 | FJX1 |
| chr8-22 | chr8 | 2093122 | 2093272 | 80.3 | 3' UTR (NM_003970, exon 37 of 37) | 100039 | MYOM2 |
| chr15-21 | chr15 | 92080153 | 92080303 | 80.3 | Intergenic | -316710 | SLCO3A1 |
| chr3-22 | chr3 | 46307019 | 46307169 | 80.3 | exon (NM_001164680, exon 3 of 3) | 23222 | CCR3 |
| chr12-35 | chr12 | 25148689 | 25148839 | 80.3 | exon (NM_001101339, exon 3 of 4) | 1609 | C12orf77 |
| chr9-25 | chr9 | 123446819 | 123446969 | 80.3 | intron (NM_001080497, intron 1 of 5) | 29871 | MEGF9 |
| chr2-53 | chr2 | 54856328 | 54856478 | 80.3 | exon (NM_003128, exon 14 of 36) | 70872 | SPTBN1 |
| chr6-22 | chr6 | 26066342 | 26066492 | 80.3 | Intergenic | -9718 | HIST1H1C |
| chr1-40 | chr1 | 12164336 | 12164486 | 80.3 | intron (NM_001243, intron 3 of 14) | -21547 | TNFRSF8 |
| chr19-37 | chr19 | 49999385 | 49999535 | 80.3 | promoter-TSS (NM_001015) | -162 | RPS11 |
| chr19-36 | chr19 | 39179595 | 39179745 | 80.3 | intron (NM_004924, intron 1 of 20) | 41403 | ACTN4 |
| chr16-16 | chr16 | 16071046 | 16071196 | 80.3 | intron (NM_004996, intron 1 of 30) | 27687 | ABCC1 |
| chr10-44 | chr10 | 3137938 | 3138088 | 80.3 | intron (NM_001242339, intron 3 of 23) | 27194 | PFKP |
| chr2-46 | chr2 | 104243960 | 104244110 | 80.3 | Intergenic | 780755 | LOC100287010 |
| chr10-57 | chr10 | 105001198 | 105001348 | 80.3 | Intergenic | -4371 | LOC729020 |
| chr9-21 | chr9 | 5839410 | 5839560 | 80.3 | Intergenic | -6404 | ERMP1 |
| chr1-43 | chr1 | 19423882 | 19424032 | 80.3 | intron (NM_020765, intron 90 of 105) | 112789 | UBR4 |
| chr16-21 | chr16 | 73054431 | 73054581 | 80.3 | intron (NM_006885, intron 1 of 9) | 27768 | ZFHX3 |
| chr4-13 | chr4 | 88583420 | 88583570 | 80.3 | exon (NM_001079911, exon 5 of 5) | 12041 | DMP1 |
| chr11-23 | chr11 | 63334960 | 63335110 | 80.3 | Intergenic | -4180 | HRASLS2 |
| chr2-57 | chr2 | 234766469 | 234766619 | 80.3 | Intergenic | -3332 | HJURP |
| chr16-22 | chr16 | 87437817 | 87437967 | 80.3 | 3' UTR (NM_022818, exon 4 of 4) | 12091 | MAP1LC3B |
| chr3-16 | chr3 | 51680172 | 51680322 | 80.3 | intron (NM_015106, intron 17 of 21) | -24975 | TEX264 |
| chr12-38 | chr12 | 132474507 | 132474657 | 80.3 | exon (NM_015409, exon 8 of 53) | 40117 | EP400 |
| chr19-43 | chr19 | 42354421 | 42354571 | 80.3 | intron (NM_001040283, intron 7 of 8) | 5410 | DMRTC2 |
| chr12-29 | chr12 | 52674870 | 52675020 | 80.3 | Intergenic | 10354 | KRT81 |
| chr3-26 | chr3 | 152856482 | 152856632 | 80.3 | Intergenic | -23472 | RAP2B |
| chr5-30 | chr5 | 55419496 | 55419646 | 80.3 | intron (NM_024669, intron 8 of 11) | 109615 | ANKRD55 |
| chr2-56 | chr2 | 225965773 | 225965923 | 80.3 | Intergenic | -58518 | DOCK10 |
| chr18-16 | chr18 | 77513872 | 77514022 | 80.3 | 3' UTR (NM_001202504, exon 13 of 13) | 72517 | CTDP1 |
| chr11-29 | chr11 | 748528 | 748678 | 80.3 | intron (NM_006755, intron 1 of 7) | 1171 | TALDO1 |
| chr17-33 | chr17 | 41799083 | 41799233 | 80.3 | Intergenic | 36998 | SOST |
| chr7-28 | chr7 | 37941942 | 37942092 | 80.3 | Intergenic | 14508 | SFRP4 |
| chr3-23 | chr3 | 113006251 | 113006401 | 80.3 | TTS (NM_033254) | 74951 | BOC |
| chr19-38 | chr19 | 9271563 | 9271713 | 80.3 | exon (NM_020933, exon 7 of 7) | 20582 | ZNF317 |
| chr6-20 | chr6 | 4358870 | 4359020 | 80.3 | Intergenic | -223114 | ECI2 |
| chr9-26 | chr9 | 132333045 | 132333195 | 80.3 | Intergenic | 49935 | C9orf50 |
| chr1-46 | chr1 | 248684895 | 248685045 | 80.3 | promoter-TSS (NM_001013355) | 22 | OR2G6 |
| chr12-34 | chr12 | 12369378 | 12369528 | 80.3 | intron (NM_002336, intron 2 of 22) | 50358 | LRP6 |
| chr17-31 | chr17 | 19235230 | 19235380 | 80.3 | exon (NM_014964, exon 10 of 11) | 12582 | MIR1180 |
| chr8-18 | chr8 | 56663625 | 56663775 | 80.3 | exon (NM_152417, exon 5 of 6) | -22091 | TGS1 |
| chr1-49 | chr1 | 54518730 | 54518880 | 80.3 | promoter-TSS (NR_039942) | 306 | TMEM59 |
| chr5-32 | chr5 | 58814145 | 58814295 | 80.3 | intron (NM_001165899, intron 3 of 16) | 68104 | PDE4D |
| chr10-45 | chr10 | 29769376 | 29769526 | 80.3 | intron (NM_021738, intron 29 of 37) | 64575 | MIR604 |
| chr4-16 | chr4 | 129438949 | 129439099 | 80.3 | Intergenic | -229040 | PGRMC2 |
| chr3-24 | chr3 | 115523721 | 115523871 | 80.3 | 3' UTR (NM_002338, exon 7 of 7) | 181645 | GAP43 |
| chr19-44 | chr19 | 51854064 | 51854214 | 80.3 | intron (NM_001014763, intron 2 of 4) | 3957 | ETFB |
| chr12-30 | chr12 | 63118702 | 63118852 | 80.3 | intron (NM_020700, intron 5 of 9) | 121311 | MIRLET7I |
| chr2-55 | chr2 | 85555154 | 85555304 | 80.3 | 5' UTR (NM_006464, exon 1 of 4) | 190 | TGOLN2 |
| chr22-13 | chr22 | 35772977 | 35773127 | 80.3 | Intergenic | -4008 | HMOX1 |
| chr17-26 | chr17 | 41633677 | 41633827 | 80.3 | Intergenic | -9952 | ETV4 |
| chr12-37 | chr12 | 110206822 | 110206972 | 80.3 | exon (NM_032829, exon 3 of 3) | 4395 | FAM222A-AS1 |
| chr10-56 | chr10 | 49612892 | 49613042 | 80.3 | exon (NM_002750, exon 2 of 11) | 3280 | MAPK8 |
| chr7-30 | chr7 | 106018644 | 106018794 | 80.3 | Intergenic | -93081 | NAMPT |
| chr2-54 | chr2 | 64871523 | 64871673 | 80.3 | intron (NM_014755, intron 1 of 1) | 9448 | SERTAD2 |
| chr1-51 | chr1 | 167096393 | 167096543 | 80.3 | exon (NM_001080426, exon 5 of 5) | 32381 | DUSP27 |
| chr17-32 | chr17 | 35848386 | 35848536 | 80.3 | Intergenic | -1490 | DUSP14 |
| chr9-27 | chr9 | 3875334 | 3875484 | 80.3 | intron (NM_152629, intron 7 of 9) | -23237 | GLIS3-AS1 |
| chr6-18 | chr6 | 37027176 | 37027326 | 80.3 | Intergenic | 53828 | FGD2 |
| chr7-33 | chr7 | 139615947 | 139616097 | 80.3 | intron (NM_001061, intron 4 of 12) | 87070 | TBXAS1 |
| chr4-15 | chr4 | 39027526 | 39027676 | 80.3 | intron (NM_024943, intron 1 of 6) | 6440 | TMEM156 |
| chr10-50 | chr10 | 49618067 | 49618217 | 80.3 | exon (NM_002750, exon 4 of 11) | 8455 | MAPK8 |
| chr3-18 | chr3 | 149091711 | 149091861 | 80.3 | intron (NM_014220, intron 3 of 4) | 3782 | TM4SF1 |
| chr6-24 | chr6 | 101075626 | 101075776 | 80.3 | intron (NM_006828, intron 28 of 41) | -164150 | SIM1 |
| chr12-28 | chr12 | 6641758 | 6641908 | 80.3 | TTS (NM_014865) | -1752 | GAPDH |
| chr8-19 | chr8 | 107095241 | 107095391 | 80.3 | Intergenic | -187090 | OXR1 |
| chr19-41 | chr19 | 38561315 | 38561465 | 80.3 | intron (NM_015073, intron 2 of 21) | 153500 | DPF1 |
| chr9-23 | chr9 | 75598627 | 75598777 | 80.3 | Intergenic | -30469 | ALDH1A1 |
| chr10-55 | chr10 | 28229365 | 28229515 | 80.3 | intron (NM_018076, intron 13 of 19) | 58537 | ARMC4 |
| chr11-25 | chr11 | 111613981 | 111614131 | 80.3 | intron (NM_001177562, intron 11 of 13) | 23113 | PPP2R1B |
| chr15-26 | chr15 | 72529826 | 72529976 | 80.3 | Intergenic | -6174 | PKM |
| chr6-23 | chr6 | 97590884 | 97591034 | 80.3 | 3' UTR (NM_198468, exon 25 of 25) | 140093 | MMS22L |
| chr6-17 | chr6 | 26025953 | 26026103 | 80.3 | Intergenic | 1452 | HIST1H4B |
| chr13-7 | chr13 | 90053551 | 90053701 | 80.3 | Intergenic | 163041 | LINC00353 |
| chr19-45 | chr19 | 58232222 | 58232372 | 80.3 | exon (NM_024833, exon 4 of 4) | 6698 | ZNF671 |
| chrX-9 | chrX | 66834364 | 66834514 | 80.3 | intron (NM_001011645, intron 1 of 7) | 45756 | AR |
| chr18-18 | chr18 | 55254148 | 55254298 | 80.3 | promoter-TSS (NM_000140) | -254 | FECH |
| chr14-18 | chr14 | 23479106 | 23479256 | 80.3 | intron (NM_001130706, intron 1 of 7) | 179 | C14orf93 |
| chr2-52 | chr2 | 234835063 | 234835213 | 80.3 | intron (NM_024080, intron 1 of 25) | 9095 | TRPM8 |
| chr20-16 | chr20 | 34195195 | 34195345 | 80.3 | intron (NR_024377, intron 1 of 40) | 214 | FER1L4 |
| chr10-58 | chr10 | 6443574 | 6443724 | 73.6 | Intergenic | 123999 | LOC399715 |
| chr2-71 | chr2 | 209353519 | 209353669 | 73.6 | intron (NM_005048, intron 10 of 12) | 82038 | PTH2R |
| chr5-34 | chr5 | 102612060 | 102612210 | 73.6 | exon (NM_033211, exon 3 of 3) | 17693 | C5orf30 |
| chr11-38 | chr11 | 73999308 | 73999458 | 73.6 | intron (NM_182904, intron 5 of 12) | 23316 | P4HA3 |
| chr20-17 | chr20 | 37547309 | 37547459 | 73.6 | 3' UTR (NM_001172735, exon 10 of 10) | -7571 | FAM83D |
| chr3-30 | chr3 | 119013888 | 119014038 | 73.6 | intron (NM_020754, intron 1 of 11) | 743 | ARHGAP31 |
| chr3-29 | chr3 | 114343770 | 114343920 | 73.6 | promoter-TSS (NM_001164347) | -53 | ZBTB20 |
| chr8-30 | chr8 | 126559510 | 126559660 | 73.6 | Intergenic | 117022 | TRIB1 |
| chr5-37 | chr5 | 120609871 | 120610021 | 73.6 | Intergenic | -577704 | FTMT |
| chr17-38 | chr17 | 33391080 | 33391230 | 73.6 | promoter-TSS (NM_001017368) | -396 | RFFL |
| chr2-45 | chr2 | 73679663 | 73679813 | 73.6 | exon (NM_015120, exon 8 of 23) | 66852 | ALMS1 |
| chr12-39 | chr12 | 6445467 | 6445617 | 73.6 | intron (NM_001065, intron 1 of 9) | 5741 | TNFRSF1A |
| chr15-28 | chr15 | 64697731 | 64697881 | 73.6 | intron (NM_016213, intron 5 of 12) | 17803 | TRIP4 |
| chr12-40 | chr12 | 41112914 | 41113064 | 73.6 | intron (NM_001843, intron 1 of 23) | 26745 | CNTN1 |
| chr19-46 | chr19 | 2917193 | 2917343 | 73.6 | exon (NM_173480, exon 4 of 4) | 16372 | ZNF57 |
| chr7-37 | chr7 | 44119525 | 44119675 | 73.6 | intron (NM_013284, intron 2 of 10) | 2529 | POLM |
| chr20-18 | chr20 | 48646637 | 48646787 | 73.6 | Intergenic | 11454 | LINC00651 |
| chr19-51 | chr19 | 53303443 | 53303593 | 73.6 | exon (NM_006969, exon 4 of 4) | -13484 | ZNF600 |
| chr9-28 | chr9 | 131545863 | 131546013 | 73.6 | Intergenic | -3572 | TBC1D13 |
| chr11-31 | chr11 | 9385869 | 9386019 | 73.6 | Intergenic | -20225 | IPO7 |
| chr7-32 | chr7 | 134200701 | 134200851 | 73.6 | Intergenic | -11568 | AKR1B10 |
| chr15-24 | chr15 | 51169279 | 51169429 | 73.6 | Intergenic | -31515 | AP4E1 |
| chr1-47 | chr1 | 5924530 | 5924680 | 73.6 | intron (NM_015102, intron 27 of 29) | -1804 | MIR4689 |
| chr17-39 | chr17 | 33584231 | 33584381 | 73.6 | intron (NM_144975, intron 1 of 4) | 14220 | SLFN5 |
| chr8-20 | chr8 | 131012227 | 131012377 | 73.6 | intron (NR_046361, intron 1 of 13) | 8397 | MIR5194 |
| chr8-25 | chr8 | 43052761 | 43052911 | 73.6 | exon (NM_152419, exon 16 of 18) | 57244 | HGSNAT |
| chr16-24 | chr16 | 11734421 | 11734571 | 73.6 | Intergenic | -27793 | SNN |
| chr21-12 | chr21 | 30693518 | 30693668 | 73.6 | non-coding (NR_027655, exon 2 of 8) | 16033 | BACH1 |
| chr2-63 | chr2 | 39054868 | 39055018 | 73.6 | intron (NM_198963, intron 14 of 23) | 48078 | DHX57 |
| chr16-19 | chr16 | 20996475 | 20996625 | 73.6 | exon (NM_017539, exon 48 of 62) | 84475 | LYRM1 |
| chr12-46 | chr12 | 132636819 | 132636969 | 73.6 | TTS (NM_024078) | 7901 | NOC4L |
| chr3-20 | chr3 | 32932405 | 32932555 | 73.6 | exon (NM_001039111, exon 4 of 4) | -60586 | CCR4 |
| chr7-41 | chr7 | 131438566 | 131438716 | 73.6 | Intergenic | -197265 | PODXL |
| chr4-22 | chr4 | 147562278 | 147562428 | 73.6 | 3' UTR (NM_004575, exon 2 of 2) | 2308 | POU4F2 |
| chr17-30 | chr17 | 8132777 | 8132927 | 73.6 | intron (NR_046431, intron 18 of 21) | -5491 | LINC00324 |
| chr16-28 | chr16 | 57844310 | 57844460 | 73.6 | Intergenic | -7946 | KIFC3 |
| chr3-27 | chr3 | 62204329 | 62204479 | 73.6 | intron (NM_002841, intron 12 of 29) | 100218 | LOC100506994 |
| chr17-42 | chr17 | 74733517 | 74733667 | 73.6 | promoter-TSS (NM_001195427) | 9 | MFSD11 |
| chr7-31 | chr7 | 130794732 | 130794882 | 73.6 | promoter-TSS (NM_001145354) | -48 | MKLN1 |
| chr5-38 | chr5 | 141977769 | 141977919 | 73.6 | intron (NM_033136, intron 2 of 2) | 15882 | FGF1 |
| chr2-72 | chr2 | 220150178 | 220150328 | 73.6 | 3' UTR (NM_006736, exon 9 of 9) | 6213 | DNAJB2 |
| chr11-30 | chr11 | 5301927 | 5302077 | 73.6 | Intergenic | -10629 | HBE1 |
| chr15-25 | chr15 | 59903978 | 59904128 | 73.6 | promoter-TSS (NM_004751) | 71 | GCNT3 |
| chr10-53 | chr10 | 115548531 | 115548681 | 73.6 | Intergenic | -10793 | MIR4483 |
| chrX-8 | chrX | 15511337 | 15511487 | 73.6 | 5' UTR (NM_003662, exon 1 of 10) | 299 | PIR |
| chr9-32 | chr9 | 95009679 | 95009829 | 73.6 | exon (NM_013417, exon 26 of 34) | 45075 | MIR3651 |
| chr6-21 | chr6 | 4402779 | 4402929 | 73.6 | Intergenic | -267023 | ECI2 |
| chr2-64 | chr2 | 42161157 | 42161307 | 73.6 | Intergenic | 19711 | C2orf91 |
| chr7-36 | chr7 | 43612235 | 43612385 | 73.6 | Intergenic | -10382 | STK17A |
| chr15-32 | chr15 | 90174738 | 90174888 | 73.6 | exon (NM_198525, exon 15 of 19) | 23869 | KIF7 |
| chr3-28 | chr3 | 101499599 | 101499749 | 73.6 | intron (NM_001134456, intron 1 of 7) | 1388 | NXPE3 |
| chr8-29 | chr8 | 120865161 | 120865311 | 73.6 | intron (NM_024094, intron 2 of 8) | 2934 | DSCC1 |
| chr7-39 | chr7 | 129649808 | 129649958 | 73.6 | Intergenic | 41350 | ZC3HC1 |
| chr19-40 | chr19 | 22469316 | 22469466 | 73.6 | intron (NM_001242680, intron 1 of 3) | 139 | ZNF729 |
| chr7-34 | chr7 | 1553069 | 1553219 | 73.6 | Intergenic | -9126 | INTS1 |
| chr1-57 | chr1 | 246470038 | 246470188 | 73.6 | intron (NM_022743, intron 5 of 11) | 110601 | SMYD3 |
| chr17-41 | chr17 | 67570625 | 67570775 | 73.6 | Intergenic | 159862 | MAP2K6 |
| chr2-65 | chr2 | 55517005 | 55517155 | 73.6 | 3' UTR (NM_001135597, exon 33 of 33) | 7625 | PRORSD1P |
| chr10-67 | chr10 | 124624568 | 124624718 | 73.6 | intron (NM_152644, intron 1 of 3) | -14506 | LOC399815 |
| chr17-40 | chr17 | 63781385 | 63781535 | 73.6 | intron (NM_001199165, intron 21 of 26) | 41201 | CEP112 |
| chr4-20 | chr4 | 125099183 | 125099333 | 73.6 | Intergenic | 403839 | LOC285419 |
| chr17-34 | chr17 | 49010929 | 49011079 | 73.6 | Intergenic | -65665 | TOB1 |
| chr3-32 | chr3 | 151554992 | 151555142 | 73.6 | Intergenic | 23206 | AADAC |
| chr9-33 | chr9 | 137300855 | 137301005 | 73.6 | exon (NM_002957, exon 4 of 10) | 29673 | MIR4669 |
| chr11-34 | chr11 | 123325041 | 123325191 | 73.6 | Intergenic | -71412 | GRAMD1B |
| chr5-35 | chr5 | 179247436 | 179247586 | 73.6 | promoter-TSS (NM_003900) | -331 | SQSTM1 |
| chr10-62 | chr10 | 49633902 | 49634052 | 73.6 | exon (NM_002750, exon 7 of 11) | 24290 | MAPK8 |
| chr9-34 | chr9 | 137323722 | 137323872 | 73.6 | exon (NM_002957, exon 8 of 10) | 52540 | MIR4669 |
| chr1-54 | chr1 | 156627316 | 156627466 | 73.6 | intron (NM_021948, intron 10 of 13) | 15651 | BCAN |
| chr10-64 | chr10 | 96989580 | 96989730 | 73.6 | TTS (NM_207321) | 35698 | C10orf129 |
| chr5-36 | chr5 | 66178509 | 66178659 | 73.6 | intron (NM_015183, intron 1 of 27) | 53980 | MAST4 |
| chr22-14 | chr22 | 18189391 | 18189541 | 73.6 | promoter-TSS (NM_001270733) | 42 | BCL2L13 |
| chr3-25 | chr3 | 129376643 | 129376793 | 73.6 | intron (NM_001017395, intron 4 of 5) | 30857 | TMCC1 |
| chr7-35 | chr7 | 31997064 | 31997214 | 73.6 | intron (NM_001191058, intron 3 of 18) | 113327 | PDE1C |
| chr15-31 | chr15 | 78572783 | 78572933 | 73.6 | 3' UTR (NM_018602, exon 8 of 8) | 14298 | DNAJA4 |
| chr2-59 | chr2 | 17536940 | 17537090 | 73.6 | Intergenic | 162691 | RAD51AP2 |
| chr7-29 | chr7 | 50602716 | 50602866 | 73.6 | intron (NR_033845, intron 1 of 4) | 3334 | LOC100129427 |
| chr5-33 | chr5 | 75375352 | 75375502 | 73.6 | Intergenic | -3878 | SV2C |
| chr3-19 | chr3 | 25623802 | 25623952 | 73.6 | intron (NM_016152, intron 5 of 7) | 81911 | TOP2B |
| chr17-36 | chr17 | 17721266 | 17721416 | 73.6 | intron (NM_004176, intron 6 of 18) | -4096 | MIR33B |
| chr15-27 | chr15 | 93347615 | 93347765 | 73.6 | Intergenic | 8976 | ASB9P1 |
| chrX-13 | chrX | 64949211 | 64949361 | 73.6 | intron (NM_002444, intron 3 of 12) | 61775 | MSN |
| chr12-41 | chr12 | 96027038 | 96027188 | 73.6 | Intergenic | -81850 | USP44 |
| chr11-33 | chr11 | 59305352 | 59305502 | 73.6 | Intergenic | 23041 | OR4D9 |
| chr4-21 | chr4 | 140941982 | 140942132 | 73.6 | intron (NM_018717, intron 1 of 5) | 133176 | MAML3 |
| chr3-21 | chr3 | 45046993 | 45047143 | 73.6 | intron (NR_023353, intron 4 of 6) | -20691 | CLEC3B |
| chr8-27 | chr8 | 101894325 | 101894475 | 73.6 | Intergenic | 68399 | YWHAZ |
| chr4-12 | chr4 | 84469701 | 84469851 | 73.6 | intron (NM_001256421, intron 3 of 12) | 12306 | AGPAT9 |
| chr9-30 | chr9 | 12784589 | 12784739 | 73.6 | intron (NM_203403, intron 1 of 1) | 9652 | LURAP1L |
| chr17-35 | chr17 | 80645281 | 80645431 | 73.6 | intron (NM_006822, intron 1 of 5) | 11242 | RAB40B |
| chr7-26 | chr7 | 81128862 | 81129012 | 73.6 | Intergenic | 270515 | HGF |
| chr11-37 | chr11 | 67290126 | 67290276 | 73.6 | intron (NM_016366, intron 1 of 6) | 698 | CABP2 |
| chr2-48 | chr2 | 133018862 | 133019012 | 73.6 | Intergenic | -3395 | ANKRD30BL |
| chr18-20 | chr18 | 54041359 | 54041509 | 73.6 | Intergenic | -236667 | LOC100505474 |
| chr15-23 | chr15 | 44281467 | 44281617 | 73.6 | intron (NM_032892, intron 1 of 13) | -111173 | PIN4P1 |
| chr3-33 | chr3 | 168864213 | 168864363 | 73.6 | promoter-TSS (NM_001105077) | 112 | MECOM |
| chr12-45 | chr12 | 124337780 | 124337930 | 73.6 | exon (NM_207437, exon 35 of 78) | 90813 | DNAH10 |
| chr7-38 | chr7 | 72299937 | 72300087 | 73.6 | promoter-TSS (NR_001588) | 60 | SBDSP1 |
| chr9-24 | chr9 | 123165792 | 123165942 | 73.6 | intron (NM_001011649, intron 32 of 36) | -158539 | MIR147A |
| chr10-63 | chr10 | 54031107 | 54031257 | 73.6 | exon (NM_006258, exon 11 of 18) | 42706 | LOC100506939 |
| chr19-47 | chr19 | 10613406 | 10613556 | 66.9 | promoter-TSS (NM_012289) | 0 | KEAP1 |
| chr3-34 | chr3 | 178978355 | 178978505 | 66.9 | promoter-TSS (NM_171829) | -751 | KCNMB3 |
| chr19-50 | chr19 | 36003893 | 36004043 | 66.9 | exon (NM_033317, exon 1 of 16) | 592 | DMKN |
| chr4-25 | chr4 | 161240372 | 161240522 | 66.9 | Intergenic | 1051449 | RAPGEF2 |
| chr2-51 | chr2 | 227876777 | 227876927 | 66.9 | intron (NM_000092, intron 45 of 47) | 152423 | COL4A4 |
| chr19-55 | chr19 | 41196854 | 41197004 | 66.9 | promoter-TSS (NM_004756) | -373 | NUMBL |
| chr1-68 | chr1 | 172903129 | 172903279 | 66.9 | Intergenic | 116899 | TNFSF18 |
| chr7-48 | chr7 | 100743554 | 100743704 | 66.9 | Intergenic | 14843 | TRIM56 |
| chr9-29 | chr9 | 7798471 | 7798621 | 66.9 | 3' UTR (NM_033428, exon 2 of 2) | 1253 | C9orf123 |
| chr20-20 | chr20 | 870922 | 871072 | 66.9 | exon (NM_015985, exon 2 of 9) | 25963 | ANGPT4 |
| chr12-43 | chr12 | 104682455 | 104682605 | 66.9 | intron (NM_001261445, intron 1 of 14) | 2070 | TXNRD1 |
| chr12-50 | chr12 | 31649278 | 31649428 | 66.9 | intron (NM_144973, intron 1 of 20) | -93504 | DENND5B-AS1 |
| chr8-35 | chr8 | 132959246 | 132959396 | 66.9 | intron (NM_015137, intron 4 of 22) | 42965 | EFR3A |
| chr10-65 | chr10 | 100017829 | 100017979 | 66.9 | exon (NM_032211, exon 7 of 15) | 10103 | LOXL4 |
| chr11-43 | chr11 | 56949304 | 56949454 | 66.9 | exon (NM_001005210, exon 1 of 2) | 158 | LRRC55 |
| chr18-19 | chr18 | 32915051 | 32915201 | 66.9 | 3' UTR (NM_006965, exon 4 of 4) | 9300 | ZNF24 |
| chr22-17 | chr22 | 46930566 | 46930716 | 66.9 | exon (NM_014246, exon 1 of 35) | 2426 | CELSR1 |
| chr2-60 | chr2 | 27238819 | 27238969 | 66.9 | intron (NM_012326, intron 1 of 6) | -16880 | TMEM214 |
| chr17-47 | chr17 | 27031689 | 27031839 | 66.9 | exon (NM_152465, exon 2 of 4) | 7108 | PROCA1 |
| chr9-38 | chr9 | 114820752 | 114820902 | 66.9 | exon (NM_022486, exon 14 of 17) | 116729 | SUSD1 |
| chr11-36 | chr11 | 57519069 | 57519219 | 66.9 | 5' UTR (NM_001145101, exon 1 of 3) | 109 | BTBD18 |
| chr1-58 | chr1 | 22222492 | 22222642 | 66.9 | intron (NM_005529, intron 2 of 96) | 41183 | HSPG2 |
| chr16-31 | chr16 | 16082254 | 16082404 | 66.9 | intron (NM_004996, intron 1 of 30) | 38895 | ABCC1 |
| chr17-49 | chr17 | 57833549 | 57833699 | 66.9 | intron (NM_030938, intron 5 of 11) | 48761 | VMP1 |
| chr1-59 | chr1 | 24620924 | 24621074 | 66.9 | Intergenic | -24813 | GRHL3 |
| chr15-36 | chr15 | 52388011 | 52388161 | 66.9 | Intergenic | 16886 | BCL2L10 |
| chr8-26 | chr8 | 48426120 | 48426270 | 66.9 | intron (NM_001080394, intron 8 of 19) | 224531 | CEBPD |
| chr2-61 | chr2 | 37442535 | 37442685 | 66.9 | intron (NM_005760, intron 8 of 15) | 16130 | CEBPZ |
| chr6-26 | chr6 | 17907982 | 17908132 | 66.9 | intron (NM_001105568, intron 2 of 37) | 79797 | KIF13A |
| chr12-61 | chr12 | 132345162 | 132345312 | 66.9 | Intergenic | 32296 | MMP17 |
| chr2-79 | chr2 | 202903742 | 202903892 | 66.9 | TTS (NM_003507) | 4507 | FZD7 |
| chr1-71 | chr1 | 212583541 | 212583691 | 66.9 | intron (NM_018252, intron 2 of 7) | 4627 | TMEM206 |
| chr11-47 | chr11 | 73849740 | 73849890 | 66.9 | exon (NM_015531, exon 5 of 31) | 32249 | C2CD3 |
| chr3-40 | chr3 | 47422462 | 47422612 | 66.9 | promoter-TSS (NM_015466) | 46 | PTPN23 |
| chr15-29 | chr15 | 69385583 | 69385733 | 66.9 | intron (NR_026949, intron 3 of 3) | 12468 | LINC00277 |
| chr13-9 | chr13 | 42875602 | 42875752 | 66.9 | exon (NM_016248, exon 8 of 13) | 29388 | AKAP11 |
| chr19-48 | chr19 | 18490893 | 18491043 | 66.9 | Intergenic | -6000 | GDF15 |
| chr10-83 | chr10 | 114893393 | 114893543 | 66.9 | intron (NM_001146283, intron 5 of 12) | 183459 | TCF7L2 |
| chrX-15 | chrX | 106161274 | 106161424 | 66.9 | promoter-TSS (NM_001171095) | -241 | CLDN2 |
| chr22-16 | chr22 | 38695947 | 38696097 | 66.9 | exon (NM_001894, exon 6 of 11) | 17391 | CSNK1E |
| chr9-36 | chr9 | 21384836 | 21384986 | 66.9 | exon (NM_000605, exon 1 of 1) | 485 | IFNA2 |
| chr10-76 | chr10 | 49609681 | 49609831 | 66.9 | promoter-TSS (NM_002750) | 69 | MAPK8 |
| chr11-48 | chr11 | 112138568 | 112138718 | 66.9 | Intergenic | -7060 | C11orf34 |
| chr6-32 | chr6 | 116580205 | 116580355 | 66.9 | Intergenic | -5019 | TSPYL4 |
| chr10-66 | chr10 | 123900501 | 123900651 | 66.9 | intron (NM_206861, intron 4 of 19) | -22529 | TACC2 |
| chr7-53 | chr7 | 144344880 | 144345030 | 66.9 | intron (NM_001042482, intron 5 of 7) | 188191 | TPK1 |
| chr12-58 | chr12 | 109232270 | 109232420 | 66.9 | intron (NM_001161330, intron 2 of 13) | -11018 | SSH1 |
| chr17-48 | chr17 | 48354065 | 48354215 | 66.9 | intron (NM_153229, intron 1 of 4) | 2352 | TMEM92 |
| chr3-31 | chr3 | 136505694 | 136505844 | 66.9 | Intergenic | -32092 | SLC35G2 |
| chr16-30 | chr16 | 14580520 | 14580670 | 66.9 | intron (NM_001134477, intron 21 of 23) | 143533 | PARN |
| chr3-41 | chr3 | 128930830 | 128930980 | 66.9 | Intergenic | -28095 | CNBP |
| chr17-46 | chr17 | 18825530 | 18825680 | 66.9 | intron (NM_001243941, intron 7 of 9) | -28384 | SLC5A10 |
| chr8-24 | chr8 | 29316580 | 29316730 | 66.9 | Intergenic | -108388 | DUSP4 |
| chr11-44 | chr11 | 61739415 | 61739565 | 66.9 | Intergenic | -4358 | FTH1 |
| chr16-25 | chr16 | 14404511 | 14404661 | 66.9 | Intergenic | 1444 | MIR365A |
| chr9-39 | chr9 | 133556609 | 133556759 | 66.9 | exon (NM_021619, exon 5 of 5) | -12474 | EXOSC2 |
| chr20-26 | chr20 | 52740354 | 52740504 | 66.9 | Intergenic | 50087 | CYP24A1 |
| chr12-60 | chr12 | 125352030 | 125352180 | 66.9 | Intergenic | -3586 | SCARB1 |
| chr10-61 | chr10 | 49628186 | 49628336 | 66.9 | exon (NM_002750, exon 5 of 11) | 18574 | MAPK8 |
| chr8-33 | chr8 | 102884471 | 102884621 | 66.9 | intron (NM_001040628, intron 4 of 7) | -81107 | NCALD |
| chr2-75 | chr2 | 128944684 | 128944834 | 66.9 | intron (NM_020120, intron 39 of 40) | 95977 | UGGT1 |
| chr7-40 | chr7 | 129665886 | 129666036 | 66.9 | intron (NM_016478, intron 6 of 9) | 25272 | ZC3HC1 |
| chr10-79 | chr10 | 70242982 | 70243132 | 66.9 | 3' UTR (NM_152707, exon 9 of 9) | -11327 | DNA2 |
| chr5-40 | chr5 | 55737402 | 55737552 | 66.9 | Intergenic | -208291 | ANKRD55 |
| chr4-17 | chr4 | 8304313 | 8304463 | 66.9 | intron (NM_053044, intron 7 of 8) | 32899 | HTRA3 |
| chr15-33 | chr15 | 92972825 | 92972975 | 66.9 | intron (NM_006011, intron 1 of 5) | 35760 | ST8SIA2 |
| chr20-25 | chr20 | 52406262 | 52406412 | 66.9 | Intergenic | 85911 | SUMO1P1 |
| chr6-35 | chr6 | 142766453 | 142766603 | 66.9 | 3' UTR (NM_001032394, exon 25 of 25) | 143472 | GPR126 |
| chr8-32 | chr8 | 29321768 | 29321918 | 66.9 | Intergenic | -113576 | DUSP4 |
| chr6-30 | chr6 | 53412983 | 53413133 | 66.9 | Intergenic | -3131 | GCLC |
| chr3-44 | chr3 | 185677610 | 185677760 | 66.9 | promoter-TSS (NR_033752) | -73 | LOC344887 |
| chr1-52 | chr1 | 90399399 | 90399549 | 66.9 | exon (NM_018103, exon 3 of 3) | 61051 | GEMIN8P4 |
| chr22-15 | chr22 | 25130003 | 25130153 | 66.9 | exon (NM_001008496, exon 14 of 21) | -30390 | TOP1P2 |
| chr12-49 | chr12 | 27904742 | 27904892 | 66.9 | intron (NM_021821, intron 7 of 7) | 19392 | MANSC4 |
| chr4-19 | chr4 | 15151556 | 15151706 | 66.9 | Intergenic | 147333 | CPEB2 |
| chr10-75 | chr10 | 47057650 | 47057800 | 66.9 | Intergenic | -25809 | PPYR1 |
| chr7-42 | chr7 | 5602336 | 5602486 | 66.9 | Intergenic | -30025 | FSCN1 |
| chr1-63 | chr1 | 37920729 | 37920879 | 66.9 | non-coding (NR_038842, exon 3 of 3) | 19240 | LOC728431 |
| chr11-40 | chr11 | 10512236 | 10512386 | 66.9 | intron (NM_001025389, intron 6 of 14) | 17562 | MIR4485 |
| chr11-49 | chr11 | 132288041 | 132288191 | 66.9 | 3' UTR (NM_002545, exon 7 of 7) | 507404 | NTM |
| chr12-52 | chr12 | 41223548 | 41223698 | 66.9 | intron (NM_001256064, intron 1 of 15) | 1883 | CNTN1 |
| chr6-29 | chr6 | 31789945 | 31790095 | 66.9 | Intergenic | -5492 | HSPA1B |
| chr6-28 | chr6 | 29932737 | 29932887 | 66.9 | Intergenic | -10080 | HCG9 |
| chr8-23 | chr8 | 27541044 | 27541194 | 66.9 | Intergenic | -18075 | MIR3622A |
| chr15-39 | chr15 | 67399439 | 67399589 | 66.9 | intron (NM_005902, intron 1 of 8) | -18540 | SMAD3 |
| chr6-34 | chr6 | 133575751 | 133575901 | 66.9 | intron (NM_172103, intron 1 of 18) | 13331 | EYA4 |
| chr1-56 | chr1 | 227545210 | 227545360 | 66.9 | Intergenic | -39459 | CDC42BPA |
| chr22-18 | chr22 | 47082174 | 47082324 | 66.9 | 3' UTR (NM_022766, exon 13 of 13) | 51903 | CERK |
| chrX-16 | chrX | 154175995 | 154176145 | 66.9 | exon (NM_000132, exon 13 of 26) | -60337 | MIR1184-3 |
| chr16-37 | chr16 | 88366808 | 88366958 | 66.9 | Intergenic | -126996 | ZNF469 |
| chr17-45 | chr17 | 17319854 | 17320004 | 66.9 | Intergenic | -2729 | SMCR9 |
| chr3-39 | chr3 | 44960228 | 44960378 | 66.9 | 3' UTR (NM_016598, exon 8 of 8) | 44205 | TGM4 |
| chr16-26 | chr16 | 14595483 | 14595633 | 66.9 | intron (NM_001134477, intron 21 of 23) | 128570 | PARN |
| chr7-47 | chr7 | 55614617 | 55614767 | 66.9 | intron (NM_030796, intron 1 of 4) | 25508 | VOPP1 |
| chr3-36 | chr3 | 10018700 | 10018850 | 66.9 | intron (NM_018447, intron 2 of 7) | 9747 | EMC3 |
| chr11-39 | chr11 | 123813868 | 123814018 | 66.9 | exon (NM_001005187, exon 1 of 1) | 602 | OR6T1 |
| chr14-23 | chr14 | 58637897 | 58638047 | 66.9 | Intergenic | -19125 | C14orf37 |
| chr12-51 | chr12 | 31902148 | 31902298 | 66.9 | Intergenic | -20115 | AMN1 |
| chr10-59 | chr10 | 34653192 | 34653342 | 66.9 | intron (NM_001184791, intron 10 of 20) | 450986 | PARD3 |
| chr8-28 | chr8 | 108117333 | 108117483 | 66.9 | Intergenic | -334936 | ABRA |
| chr5-39 | chr5 | 39119897 | 39120047 | 66.9 | intron (NM_199335, intron 13 of 17) | -45471 | RICTOR |
| chr19-39 | chr19 | 18124379 | 18124529 | 66.9 | 3' UTR (NM_001025604, exon 8 of 8) | 5477 | ARRDC2 |
| chr2-68 | chr2 | 158272235 | 158272385 | 66.9 | exon (NM_004288, exon 8 of 8) | 28294 | CYTIP |
| chr1-55 | chr1 | 205797586 | 205797736 | 66.9 | 3' UTR (NM_152491, exon 13 of 13) | -15500 | SLC41A1 |
| chr14-26 | chr14 | 105417660 | 105417810 | 66.9 | exon (NM_138420, exon 7 of 7) | 26548 | PLD4 |
| chr7-44 | chr7 | 30348149 | 30348299 | 66.9 | intron (NM_147128, intron 1 of 4) | -18718 | MIR550B1 |
| chr2-62 | chr2 | 38443248 | 38443398 | 66.9 | Intergenic | 85076 | CYP1B1-AS1 |
| chr9-37 | chr9 | 111860568 | 111860718 | 66.9 | intron (NM_032012, intron 3 of 17) | 21582 | TMEM245 |
| chr16-23 | chr16 | 2807395 | 2807545 | 66.9 | intron (NM_016333, intron 2 of 14) | -4869 | SRRM2-AS1 |
| chr6-36 | chr6 | 157787729 | 157787879 | 66.9 | Intergenic | -14753 | ZDHHC14 |
| chr20-23 | chr20 | 49378308 | 49378458 | 66.9 | Intergenic | 30302 | PARD6B |
| chr11-46 | chr11 | 67050082 | 67050232 | 66.9 | intron (NM_001619, intron 13 of 20) | -6605 | ANKRD13D |
| chr4-24 | chr4 | 42473993 | 42474143 | 66.9 | intron (NM_006095, intron 25 of 36) | 74212 | SHISA3 |
| chr2-69 | chr2 | 161979835 | 161979985 | 66.9 | Intergenic | -13556 | TANK |
| chr10-81 | chr10 | 88683179 | 88683329 | 66.9 | exon (NM_004329, exon 12 of 13) | 34171 | MMRN2 |
| chr9-44 | chr9 | 75692556 | 75692706 | 60.2 | Intergenic | -74150 | ANXA1 |
| chr17-43 | chr17 | 1617996 | 1618146 | 60.2 | promoter-TSS (NR_029494) | -790 | MIR22 |
| chr3-50 | chr3 | 133292072 | 133292222 | 60.2 | promoter-TSS (NM_017548) | -287 | CDV3 |
| chr2-80 | chr2 | 234663540 | 234663690 | 60.2 | non-coding (NR_037694, exon 1 of 1) | 376 | LOC100286922 |
| chr17-62 | chr17 | 57906578 | 57906728 | 60.2 | intron (NM_030938, intron 10 of 11) | -11974 | MIR21 |
| chr14-29 | chr14 | 69253275 | 69253425 | 60.2 | Intergenic | 7281 | ZFP36L1 |
| chr1-61 | chr1 | 31581216 | 31581366 | 60.2 | Intergenic | -42727 | PUM1 |
| chr1-70 | chr1 | 204392028 | 204392178 | 60.2 | 3' UTR (NM_002646, exon 34 of 34) | -11159 | PPP1R15B |
| chr12-54 | chr12 | 52450983 | 52451133 | 60.2 | intron (NM_002135, intron 6 of 7) | 5872 | NR4A1 |
| chr17-50 | chr17 | 59645944 | 59646094 | 60.2 | Intergenic | 22544 | NACA2 |
| chr2-81 | chr2 | 26578356 | 26578506 | 60.2 | intron (NM_033505, intron 1 of 9) | -8746 | GPR113 |
| chr2-94 | chr2 | 220115329 | 220115479 | 60.2 | TTS (NR_026909) | -2561 | TUBA4B |
| chr7-57 | chr7 | 83773892 | 83774042 | 60.2 | intron (NM_006080, intron 1 of 16) | 50250 | SEMA3A |
| chr17-54 | chr17 | 7798388 | 7798538 | 60.2 | exon (NM_001005271, exon 9 of 40) | 6294 | CHD3 |
| chr15-30 | chr15 | 76075557 | 76075707 | 60.2 | Intergenic | -20976 | MIR4313 |
| chr8-43 | chr8 | 128227652 | 128227802 | 60.2 | Intergenic | -200130 | POU5F1B |
| chr1-77 | chr1 | 110546574 | 110546724 | 60.2 | promoter-TSS (NM_001242676) | 79 | AHCYL1 |
| chr6-31 | chr6 | 56755482 | 56755632 | 60.2 | Intergenic | -64216 | BEND6 |
| chr4-28 | chr4 | 100368323 | 100368473 | 60.2 | Intergenic | -11731 | ADH7 |
| chr1-53 | chr1 | 94429307 | 94429457 | 60.2 | Intergenic | -54370 | GCLM |
| chr11-50 | chr11 | 134048653 | 134048803 | 60.2 | exon (NM_015261, exon 21 of 35) | 45698 | NCAPD3 |
| chr5-44 | chr5 | 173959640 | 173959790 | 60.2 | Intergenic | -191860 | MSX2 |
| chr9-35 | chr9 | 20345969 | 20346119 | 60.2 | 3' UTR (NM_004529, exon 11 of 11) | 65192 | MIR4473 |
| chr6-37 | chr6 | 11144065 | 11144215 | 60.2 | Intergenic | -32069 | ERVFRD-1 |
| chr3-37 | chr3 | 15311122 | 15311272 | 60.2 | intron (NM_001018009, intron 4 of 8) | 15506 | LOC100505696 |
| chrX-18 | chrX | 46127902 | 46128052 | 60.2 | Intergenic | -178647 | ZNF673 |
| chr1-81 | chr1 | 197148175 | 197148325 | 60.2 | intron (NM_194314, intron 5 of 9) | 21422 | ZBTB41 |
| chr5-48 | chr5 | 58653303 | 58653453 | 60.2 | promoter-TSS (NM_001197219) | -651 | PDE4D |
| chr3-38 | chr3 | 41309628 | 41309778 | 60.2 | intron (NM_017886, intron 35 of 36) | 68761 | CTNNB1 |
| chr1-87 | chr1 | 243663192 | 243663342 | 60.2 | 3' UTR (NM_006642, exon 18 of 18) | 153789 | MIR4677 |
| chr12-59 | chr12 | 113275408 | 113275558 | 60.2 | intron (NM_001143854, intron 4 of 21) | 45934 | RPH3A |
| chr7-52 | chr7 | 129483913 | 129484063 | 60.2 | intron (NM_182697, intron 3 of 4) | -69134 | MIR183 |
| chr1-64 | chr1 | 53791760 | 53791910 | 60.2 | intron (NM_001018054, intron 2 of 17) | 1986 | LRP8 |
| chr20-22 | chr20 | 25218681 | 25218831 | 60.2 | Intergenic | -9950 | PYGB |
| chr10-80 | chr10 | 80016042 | 80016192 | 60.2 | Intergenic | 7735 | LOC100132987 |
| chr10-69 | chr10 | 27454253 | 27454403 | 60.2 | exon (NM_001172304, exon 6 of 11) | 10575 | MASTL |
| chr5-41 | chr5 | 73639259 | 73639409 | 60.2 | Intergenic | 297915 | ENC1 |
| chr8-39 | chr8 | 41791568 | 41791718 | 60.2 | exon (NM_001099413, exon 18 of 18) | -37363 | ANK1 |
| chr11-53 | chr11 | 66636283 | 66636433 | 60.2 | exon (NM_001040716, exon 10 of 23) | 11482 | LRFN4 |
| chr16-39 | chr16 | 19514070 | 19514220 | 60.2 | 3' UTR (NM_016641, exon 6 of 6) | 19305 | GDE1 |
| chr14-22 | chr14 | 37445841 | 37445991 | 60.2 | intron (NM_030631, intron 1 of 9) | -24320 | MIR4503 |
| chr9-41 | chr9 | 21688292 | 21688442 | 60.2 | Intergenic | -114268 | MTAP |
| chr3-43 | chr3 | 171853651 | 171853801 | 60.2 | intron (NM_022763, intron 3 of 25) | 95382 | FNDC3B |
| chr1-67 | chr1 | 114515609 | 114515759 | 60.2 | TTS (NM_152696) | -6346 | OLFML3 |
| chr15-42 | chr15 | 36720504 | 36720654 | 60.2 | Intergenic | -151233 | C15orf41 |
| chr15-35 | chr15 | 43733169 | 43733319 | 60.2 | intron (NM_005657, intron 15 of 27) | 52110 | TP53BP1 |
| chr3-51 | chr3 | 152627436 | 152627586 | 60.2 | Intergenic | 74775 | P2RY1 |
| chr19-60 | chr19 | 10501940 | 10502090 | 60.2 | 3' UTR (NM_007065, exon 8 of 8) | -10767 | TYK2 |
| chr17-63 | chr17 | 59118068 | 59118218 | 60.2 | intron (NM_001099432, intron 19 of 24) | -359114 | TBX2 |
| chr6-25 | chr6 | 10519240 | 10519390 | 60.2 | Intergenic | -2253 | GCNT2 |
| chr2-93 | chr2 | 208497562 | 208497712 | 60.2 | Intergenic | -7664 | METTL21A |
| chr6-38 | chr6 | 32805685 | 32805835 | 60.2 | exon (NM_018833, exon 2 of 12) | 787 | TAP2 |
| chr16-42 | chr16 | 47010626 | 47010776 | 60.2 | Intergenic | -3076 | DNAJA2 |
| chr17-55 | chr17 | 18625205 | 18625355 | 60.2 | promoter-TSS (NM_001037330) | -122 | TRIM16L |
| chr19-64 | chr19 | 16045726 | 16045876 | 60.2 | promoter-TSS (NM_021187) | -125 | CYP4F11 |
| chr8-42 | chr8 | 122736825 | 122736975 | 60.2 | Intergenic | -83270 | HAS2 |
| chr5-43 | chr5 | 166721962 | 166722112 | 60.2 | intron (NM_001122679, intron 1 of 28) | 10194 | TENM2 |
| chr2-66 | chr2 | 81424218 | 81424368 | 60.2 | Intergenic | -892806 | LRRTM1 |
| chr4-29 | chr4 | 122301442 | 122301592 | 60.2 | exon (NM_198179, exon 1 of 6) | 664 | QRFPR |
| chr15-41 | chr15 | 33166030 | 33166180 | 60.2 | intron (NM_001103184, intron 12 of 16) | 155900 | GREM1 |
| chr3-53 | chr3 | 195424830 | 195424980 | 60.2 | Intergenic | -1367 | MIR570 |
| chr20-21 | chr20 | 25117940 | 25118090 | 60.2 | Intergenic | 10614 | LOC284798 |
| chr3-48 | chr3 | 105833834 | 105833984 | 60.2 | Intergenic | -246022 | CBLB |
| chr15-46 | chr15 | 71530083 | 71530233 | 60.2 | intron (NM_024817, intron 3 of 16) | 96370 | THSD4 |
| chr4-26 | chr4 | 26949207 | 26949357 | 60.2 | intron (NM_001169117, intron 2 of 12) | 86969 | STIM2 |
| chr19-59 | chr19 | 10102439 | 10102589 | 60.2 | exon (NM_015719, exon 23 of 67) | 18633 | COL5A3 |
| chr13-11 | chr13 | 46730551 | 46730701 | 60.2 | exon (NM_002298, exon 5 of 16) | 25833 | LCP1 |
| chr8-46 | chr8 | 144536862 | 144537012 | 60.2 | intron (NM_015117, intron 9 of 11) | -24335 | MAFA |
| chr9-42 | chr9 | 34458486 | 34458636 | 60.2 | promoter-TSS (NM_001184945) | 7 | FAM219A |
| chr14-21 | chr14 | 57092180 | 57092330 | 60.2 | intron (NM_017799, intron 12 of 15) | 45744 | C14orf101 |
| chr19-54 | chr19 | 40433172 | 40433322 | 60.2 | exon (NM_003890, exon 2 of 36) | 7286 | FCGBP |
| chr10-84 | chr10 | 114910693 | 114910843 | 60.2 | exon (NM_001146284, exon 8 of 13) | 200759 | TCF7L2 |
| chr1-74 | chr1 | 33190871 | 33191021 | 60.2 | Intergenic | -16566 | KIAA1522 |
| chr10-87 | chr10 | 34869683 | 34869833 | 60.2 | intron (NM_001184791, intron 2 of 20) | 234495 | PARD3 |
| chr10-97 | chr10 | 124067203 | 124067353 | 60.2 | intron (NM_144587, intron 10 of 15) | 36457 | BTBD16 |
| chr5-47 | chr5 | 54650518 | 54650668 | 60.2 | intron (NM_015360, intron 14 of 26) | 47017 | SKIV2L2 |
| chr17-51 | chr17 | 73120464 | 73120614 | 60.2 | intron (NM_024585, intron 2 of 2) | 7351 | NT5C |
| chr15-34 | chr15 | 35083494 | 35083644 | 60.2 | intron (NM_005159, intron 5 of 6) | 4358 | ACTC1 |
| chr17-52 | chr17 | 78441762 | 78441912 | 60.2 | 3' UTR (NM_002522, exon 5 of 5) | 8567 | NPTX1 |
| chr10-86 | chr10 | 33294283 | 33294433 | 60.2 | Intergenic | -47065 | ITGB1 |
| chr10-96 | chr10 | 116582655 | 116582805 | 60.2 | intron (NM_001135051, intron 1 of 16) | 1227 | FAM160B1 |
| chr10-77 | chr10 | 60616387 | 60616537 | 60.2 | Intergenic | 141687 | FAM133CP |
| chr1-79 | chr1 | 162355065 | 162355215 | 60.2 | 3' UTR (NM_001085375, exon 2 of 2) | 3620 | C1orf226 |
| chr19-61 | chr19 | 12371122 | 12371272 | 60.2 | Intergenic | 34517 | ZNF44 |
| chr14-24 | chr14 | 102722227 | 102722377 | 60.2 | intron (NM_014226, intron 4 of 11) | 49229 | MOK |
| chr12-65 | chr12 | 95599502 | 95599652 | 60.2 | intron (NM_018351, intron 2 of 20) | 11663 | FGD6 |
| chr2-89 | chr2 | 166621410 | 166621560 | 60.2 | exon (NM_004482, exon 3 of 11) | 29318 | GALNT3 |
| chr10-70 | chr10 | 27536955 | 27537105 | 60.2 | non-coding (NR_003525, exon 1 of 1) | 4205 | LRRC37A6P |
| chr5-42 | chr5 | 108229896 | 108230046 | 60.2 | intron (NM_005246, intron 9 of 19) | 146448 | FER |
| chr11-52 | chr11 | 59211139 | 59211289 | 60.2 | exon (NM_001004728, exon 1 of 1) | 572 | OR5A1 |
| chr16-43 | chr16 | 48157118 | 48157268 | 60.2 | intron (NM_033226, intron 10 of 28) | 23488 | ABCC12 |
| chr17-59 | chr17 | 41081103 | 41081253 | 60.2 | Intergenic | 28364 | G6PC |
| chr15-43 | chr15 | 45444524 | 45444674 | 60.2 | exon (NM_175940, exon 25 of 34) | 22407 | DUOX1 |
| chr9-46 | chr9 | 96248676 | 96248826 | 60.2 | intron (NM_014612, intron 3 of 17) | -32877 | FAM120AOS |
| chr1-84 | chr1 | 225696361 | 225696511 | 60.2 | intron (NM_001008493, intron 10 of 14) | -79879 | LBR |
| chr12-42 | chr12 | 98795958 | 98796108 | 60.2 | Intergenic | 54890 | SLC9A7P1 |
| chr15-48 | chr15 | 99663989 | 99664139 | 60.2 | intron (NM_015286, intron 2 of 4) | 18778 | SYNM |
| chr4-23 | chr4 | 38051257 | 38051407 | 60.2 | exon (NM_015173, exon 11 of 20) | -60470 | TBC1D1 |
| chr2-96 | chr2 | 228518352 | 228518502 | 60.2 | Intergenic | -20391 | C2orf83 |
| chr16-33 | chr16 | 25059946 | 25060096 | 60.2 | Intergenic | 16959 | LOC554206 |
| chr7-54 | chr7 | 4778493 | 4778643 | 60.2 | intron (NM_001037165, intron 1 of 8) | -36694 | AP5Z1 |
| chr7-55 | chr7 | 46017900 | 46018050 | 60.2 | Intergenic | -57104 | IGFBP3 |
| chr2-87 | chr2 | 148586822 | 148586972 | 60.2 | Intergenic | -15673 | ACVR2A |
| chr12-57 | chr12 | 79477087 | 79477237 | 60.2 | intron (NM_001135805, intron 4 of 11) | 37729 | SYT1 |
| chr11-55 | chr11 | 92788130 | 92788280 | 60.2 | Intergenic | 85416 | MTNR1B |
| chr19-71 | chr19 | 51437047 | 51437197 | 60.2 | Intergenic | 19222 | KLK5 |
| chr19-53 | chr19 | 39894201 | 39894351 | 60.2 | Intergenic | -3211 | ZFP36 |
| chr10-82 | chr10 | 94404342 | 94404492 | 60.2 | intron (NM_004523, intron 17 of 21) | -45264 | HHEX |
| chr8-34 | chr8 | 129189203 | 129189353 | 60.2 | Intergenic | 26916 | MIR1208 |
| chr12-55 | chr12 | 53386932 | 53387082 | 60.2 | Intergenic | -13055 | EIF4B |
| chr10-94 | chr10 | 86320318 | 86320468 | 60.2 | Intergenic | 231983 | FAM190B |
| chr11-45 | chr11 | 65186616 | 65186766 | 60.2 | Intergenic | -3578 | NEAT1 |
| chr3-49 | chr3 | 129405972 | 129406122 | 60.2 | intron (NM_001017395, intron 3 of 5) | 1528 | TMCC1 |
| chr1-80 | chr1 | 179030067 | 179030217 | 60.2 | intron (NM_014864, intron 4 of 7) | -20970 | TOR3A |
| chr1-85 | chr1 | 233504373 | 233504523 | 60.2 | intron (NM_032435, intron 5 of 9) | 40934 | KIAA1804 |
| chr18-21 | chr18 | 71918993 | 71919143 | 60.2 | Intergenic | 40183 | CYB5A |
| chr13-8 | chr13 | 29327744 | 29327894 | 60.2 | Intergenic | -34669 | SLC46A3 |
| chr20-24 | chr20 | 50303668 | 50303818 | 60.2 | intron (NM_006045, intron 9 of 27) | 81165 | ATP9A |
| chr2-76 | chr2 | 157287659 | 157287809 | 60.2 | Intergenic | -4231 | GPD2 |
| chr1-66 | chr1 | 113867737 | 113867887 | 60.2 | Intergenic | -65663 | MAGI3 |
| chr17-60 | chr17 | 45688106 | 45688256 | 60.2 | intron (NM_006310, intron 17 of 22) | -39094 | KPNB1 |
| chr2-70 | chr2 | 174090553 | 174090703 | 60.2 | 3' UTR (NM_133646, exon 12 of 12) | 56136 | MLK7-AS1 |
| chr12-64 | chr12 | 57081997 | 57082147 | 60.2 | promoter-TSS (NM_006601) | 6 | PTGES3 |
| chr12-62 | chr12 | 19668202 | 19668352 | 60.2 | intron (NM_001267043, intron 7 of 8) | 74762 | AEBP2 |
| chr4-18 | chr4 | 13990157 | 13990307 | 60.2 | Intergenic | -123360 | LOC152742 |
| chr10-93 | chr10 | 84324658 | 84324808 | 60.2 | intron (NM_001010848, intron 2 of 8) | 687290 | NRG3 |
| chr17-53 | chr17 | 1619867 | 1620017 | 60.2 | promoter-TSS (NR_028505) | 125 | WDR81 |
| chr16-35 | chr16 | 71606736 | 71606886 | 60.2 | intron (NM_000353, intron 4 of 11) | 4187 | TAT |
| chr8-31 | chr8 | 23213686 | 23213836 | 60.2 | intron (NM_002318, intron 3 of 13) | 20040 | LOC100507156 |
| chr17-56 | chr17 | 19550800 | 19550950 | 60.2 | Intergenic | -1189 | ALDH3A2 |
| chr2-73 | chr2 | 55338981 | 55339131 | 60.2 | Intergenic | -61322 | RTN4 |
| chr16-32 | chr16 | 24855940 | 24856090 | 60.2 | Intergenic | -1169 | SLC5A11 |
| chr6-39 | chr6 | 109609614 | 109609764 | 60.2 | Intergenic | -5817 | CCDC162P |
| chr3-46 | chr3 | 30269590 | 30269740 | 60.2 | Intergenic | -378329 | TGFBR2 |
| chr11-58 | chr11 | 112137918 | 112138068 | 60.2 | Intergenic | -6410 | C11orf34 |
| chr10-85 | chr10 | 7998602 | 7998752 | 60.2 | intron (NM_031923, intron 2 of 6) | 96770 | FLJ45983 |
| chr15-40 | chr15 | 33145224 | 33145374 | 60.2 | intron (NM_001103184, intron 13 of 16) | 135094 | GREM1 |
| chr8-36 | chr8 | 143645582 | 143645732 | 60.2 | Intergenic | 50176 | ARC |
| chr9-47 | chr9 | 108059261 | 108059411 | 60.2 | intron (NM_080546, intron 1 of 15) | 52407 | SLC44A1 |
| chr7-43 | chr7 | 29690355 | 29690505 | 60.2 | non-coding (NR_024278, exon 2 of 2) | 30014 | MIR550A3 |
| chr3-45 | chr3 | 9912723 | 9912873 | 60.2 | intron (NM_001199551, intron 3 of 6) | 7698 | CIDEC |
| chr14-30 | chr14 | 90854907 | 90855057 | 60.2 | Intergenic | -8345 | CALM1 |
| chr21-13 | chr21 | 45970785 | 45970935 | 60.2 | exon (NM_198693, exon 1 of 1) | 528 | KRTAP10-2 |
| chr15-38 | chr15 | 60762194 | 60762344 | 60.2 | intron (NM_024611, intron 3 of 15) | 9075 | NARG2 |
| chr2-58 | chr2 | 10425781 | 10425931 | 60.2 | Intergenic | -17174 | HPCAL1 |
| chr12-56 | chr12 | 56994265 | 56994415 | 60.2 | intron (NM_013449, intron 23 of 28) | 35823 | BAZ2A |
| chr11-56 | chr11 | 100590661 | 100590811 | 60.2 | intron (NM_152432, intron 1 of 23) | 32329 | ARHGAP42 |
| chr15-37 | chr15 | 56189425 | 56189575 | 60.2 | intron (NM_006154, intron 5 of 28) | 19829 | NEDD4 |
| chr16-44 | chr16 | 56846908 | 56847058 | 60.2 | intron (NM_014669, intron 5 of 21) | 29570 | NUP93 |
| chr2-43 | chr2 | 12164057 | 12164207 | 60.2 | Intergenic | -187020 | MIR4262 |
| chr12-67 | chr12 | 123237201 | 123237351 | 60.2 | promoter-TSS (NM_003677) | -95 | DENR |
| chr10-68 | chr10 | 10818333 | 10818483 | 60.2 | Intergenic | 18469 | SFTA1P |
| chr8-40 | chr8 | 91081365 | 91081515 | 60.2 | exon (NM_004929, exon 4 of 11) | 13667 | CALB1 |
| chr7-50 | chr7 | 105289358 | 105289508 | 60.2 | intron (NM_138495, intron 2 of 9) | 30176 | ATXN7L1 |
| chr1-65 | chr1 | 90372274 | 90372424 | 60.2 | intron (NM_001134479, intron 2 of 2) | 84869 | LRRC8D |
| chr2-82 | chr2 | 69135720 | 69135870 | 60.2 | Intergenic | -37146 | BMP10 |
| chr7-51 | chr7 | 108060996 | 108061146 | 60.2 | intron (NM_001193583, intron 1 of 29) | 35770 | NRCAM |
| chr16-46 | chr16 | 67916098 | 67916248 | 60.2 | exon (NM_014329, exon 24 of 29) | -2608 | NRN1L |
| chr2-78 | chr2 | 183924391 | 183924541 | 60.2 | Intergenic | -18821 | DUSP19 |
| chr8-45 | chr8 | 128579392 | 128579542 | 60.2 | Intergenic | -85083 | LOC727677 |
| chr17-44 | chr17 | 4843502 | 4843652 | 60.2 | promoter-TSS (NM_001165418) | -53 | RNF167 |
| chr3-42 | chr3 | 149687117 | 149687267 | 60.2 | intron (NM_002628, intron 1 of 2) | 1549 | PFN2 |
| chr1-86 | chr1 | 240476324 | 240476474 | 60.2 | intron (NM_020066, intron 8 of 17) | 221214 | FMN2 |
| chr6-27 | chr6 | 32937929 | 32938079 | 60.2 | promoter-TSS (NM_001113182).3 | -661 | BRD2 |
| chrX-24 | chrX | 114425256 | 114425406 | 53.5 | exon (NM_001145346, exon 1 of 1) | 1368 | RBMXL3 |
| chr16-56 | chr16 | 90062328 | 90062478 | 53.5 | non-coding (NR_003227, exon 10 of 10) | 14126 | DBNDD1 |
| chr10-92 | chr10 | 50154713 | 50154863 | 53.5 | intron (NM_020945, intron 50 of 61) | -32508 | LRRC18 |
| chr14-33 | chr14 | 51208290 | 51208440 | 53.5 | exon (NM_016350, exon 24 of 29) | -73294 | SAV1 |
| chr15-45 | chr15 | 63712768 | 63712918 | 53.5 | Intergenic | -38768 | CA12 |
| chr10-101 | chr10 | 31418279 | 31418429 | 53.5 | Intergenic | -97488 | ZNF438 |
| chr11-63 | chr11 | 65249204 | 65249354 | 53.5 | Intergenic | -15954 | MALAT1 |
| chr9-51 | chr9 | 35923908 | 35924058 | 53.5 | Intergenic | 14503 | LOC158376 |
| chr17-76 | chr17 | 55447608 | 55447758 | 53.5 | intron (NM_170721, intron 4 of 9) | 113309 | MSI2 |
| chr2-114 | chr2 | 113998501 | 113998651 | 53.5 | TTS (NR_047570) | 4730 | LOC654433 |
| chr16-47 | chr16 | 163585 | 163735 | 53.5 | intron (NM_001039476, intron 2 of 10) | 25037 | NPRL3 |
| chr2-119 | chr2 | 228056211 | 228056361 | 53.5 | intron (NM_000091, intron 1 of 51) | 27005 | COL4A3 |
| chr9-54 | chr9 | 84677152 | 84677302 | 53.5 | Intergenic | 73540 | FAM75D1 |
| chrX-23 | chrX | 109331217 | 109331367 | 53.5 | intron (NM_017698, intron 2 of 5) | 5946 | MIR3978 |
| chr2-115 | chr2 | 141709570 | 141709720 | 53.5 | intron (NM_018557, intron 18 of 90) | 1179625 | LRP1B |
| chr11-57 | chr11 | 103496210 | 103496360 | 53.5 | Intergenic | -224349 | MIR4693 |
| chr17-67 | chr17 | 27966409 | 27966559 | 53.5 | intron (NM_033389, intron 13 of 14) | -18043 | CORO6 |
| chr13-10 | chr13 | 45097302 | 45097452 | 53.5 | intron (NM_183422, intron 1 of 2) | -48940 | TSC22D1 |
| chr10-98 | chr10 | 5498904 | 5499054 | 53.5 | non-coding (NR_073040, exon 10 of 10) | 10465 | NET1 |
| chr17-73 | chr17 | 43449009 | 43449159 | 53.5 | Intergenic | 53928 | ARHGAP27 |
| chr16-45 | chr16 | 58768258 | 58768408 | 53.5 | promoter-TSS (NM_002080) | -87 | GOT2 |
| chr2-101 | chr2 | 26635239 | 26635389 | 53.5 | intron (NM_145038, intron 1 of 16) | 10530 | CCDC164 |
| chr2-98 | chr2 | 235271805 | 235271955 | 53.5 | Intergenic | 133813 | ARL4C |
| chr6-44 | chr6 | 16299988 | 16300138 | 53.5 | 3' UTR (NM_001128164, exon 8 of 8) | 61252 | GMPR |
| chrX-20 | chrX | 108297671 | 108297821 | 53.5 | Intergenic | -318139 | IRS4 |
| chr5-53 | chr5 | 72969021 | 72969171 | 53.5 | intron (NM_001177693, intron 1 of 35) | 47113 | ARHGEF28 |
| chr3-47 | chr3 | 47387208 | 47387358 | 53.5 | 3' UTR (NM_025010, exon 10 of 10) | -35208 | PTPN23 |
| chr20-32 | chr20 | 33899344 | 33899494 | 53.5 | intron (NM_199487, intron 7 of 8) | -19194 | FAM83C |
| chr16-55 | chr16 | 86613572 | 86613722 | 53.5 | 3' UTR (NM_005250, exon 1 of 1) | 1532 | FOXL1 |
| chr2-107 | chr2 | 71004658 | 71004808 | 53.5 | intron (NM_001004311, intron 4 of 4) | -9358 | ADD2 |
| chrY-8 | chrY | 10029579 | 10029729 | 53.5 | Intergenic | 281247 | TTTY23 |
| chr4-33 | chr4 | 103361083 | 103361233 | 53.5 | Intergenic | -61328 | NFKB1 |
| chr17-66 | chr17 | 14204765 | 14204915 | 53.5 | exon (NM_006041, exon 1 of 2) | 334 | HS3ST3B1 |
| chr8-55 | chr8 | 62789408 | 62789558 | 53.5 | Intergenic | 162136 | MIR4470 |
| chr1-73 | chr1 | 23524919 | 23525069 | 53.5 | Intergenic | -3772 | HTR1D |
| chr12-63 | chr12 | 28188585 | 28188735 | 53.5 | Intergenic | -63744 | PTHLH |
| chr16-38 | chr16 | 1429039 | 1429189 | 53.5 | intron (NM_001193388, intron 10 of 14) | 570 | UNKL |
| chr11-67 | chr11 | 69150894 | 69151044 | 53.5 | Intergenic | 89347 | MYEOV |
| chr12-47 | chr12 | 516021 | 516171 | 53.5 | intron (NM_001130146, intron 2 of 11) | 5355 | CCDC77 |
| chr5-54 | chr5 | 106805353 | 106805503 | 53.5 | intron (NM_001962, intron 1 of 4) | 201168 | EFNA5 |
| chr7-58 | chr7 | 114459758 | 114459908 | 53.5 | Intergenic | -102376 | MDFIC |
| chr8-58 | chr8 | 129077920 | 129078070 | 53.5 | intron (NR_003367, intron 4 of 7) | 16597 | MIR1207 |
| chr9-50 | chr9 | 33942768 | 33942918 | 53.5 | intron (NM_018449, intron 15 of 28) | 8557 | SNORD121B |
| chr17-58 | chr17 | 37617492 | 37617642 | 53.5 | promoter-TSS (NM_015083) | -172 | CDK12 |
| chr16-53 | chr16 | 81439470 | 81439620 | 53.5 | Intergenic | 20922 | MIR4720 |
| chr1-69 | chr1 | 200609174 | 200609324 | 53.5 | Intergenic | -19387 | KIF14 |
| chr1-94 | chr1 | 156756244 | 156756394 | 53.5 | intron (NM_005973, intron 2 of 6) | 19045 | PRCC |
| chr17-70 | chr17 | 39519630 | 39519780 | 53.5 | TTS (NM_002279) | 6347 | KRT33B |
| chr6-40 | chr6 | 127423437 | 127423587 | 53.5 | Intergenic | -16536 | RSPO3 |
| chr7-66 | chr7 | 91630657 | 91630807 | 53.5 | exon (NM_005751, exon 8 of 50) | 60543 | AKAP9 |
| chr21-16 | chr21 | 35134114 | 35134264 | 53.5 | intron (NM_001001132, intron 8 of 29) | 119405 | ITSN1 |
| chr1-101 | chr1 | 241805102 | 241805252 | 53.5 | Intergenic | -1476 | OPN3 |
| chr2-105 | chr2 | 63001877 | 63002027 | 53.5 | intron (NM_001142616, intron 5 of 22) | 67920 | EHBP1 |
| chr1-78 | chr1 | 158735934 | 158736084 | 53.5 | exon (NM_001005185, exon 1 of 1) | 463 | OR6N1 |
| chr1-83 | chr1 | 223888653 | 223888803 | 53.5 | promoter-TSS (NM_001146068) | -567 | CAPN2 |
| chr3-58 | chr3 | 120412347 | 120412497 | 53.5 | intron (NM_173825, intron 6 of 7) | -11004 | HGD |
| chr12-81 | chr12 | 132970778 | 132970928 | 53.5 | Intergenic | -64948 | GALNT9 |
| chr11-62 | chr11 | 20486145 | 20486295 | 53.5 | intron (NM_001145167, intron 12 of 14) | 77144 | PRMT3 |
| chr20-35 | chr20 | 52238149 | 52238299 | 53.5 | Intergenic | -38588 | ZNF217 |
| chr12-66 | chr12 | 96196924 | 96197074 | 53.5 | Intergenic | -12463 | NTN4 |
| chr17-64 | chr17 | 78155114 | 78155264 | 53.5 | intron (NR_047566, intron 2 of 21) | 2882 | CARD14 |
| chr19-76 | chr19 | 15751454 | 15751604 | 53.5 | promoter-TSS (NM_000896) | -178 | CYP4F3 |
| chr19-58 | chr19 | 9225891 | 9226041 | 53.5 | exon (NM_001005192, exon 1 of 1) | 473 | OR7G1 |
| chr2-88 | chr2 | 153361997 | 153362147 | 53.5 | intron (NM_052905, intron 1 of 25) | 170321 | FMNL2 |
| chr10-107 | chr10 | 76677388 | 76677538 | 53.5 | intron (NM_012330, intron 3 of 17) | 91292 | KAT6B |
| chr19-82 | chr19 | 39569346 | 39569496 | 53.5 | Intergenic | -5524 | PAPL |
| chr9-45 | chr9 | 93959646 | 93959796 | 53.5 | Intergenic | -122307 | LOC100129316 |
| chr10-100 | chr10 | 6321550 | 6321700 | 53.5 | intron (NR_040079, intron 1 of 6) | 1975 | LOC399715 |
| chr20-29 | chr20 | 20140197 | 20140347 | 53.5 | intron (NM_001167816, intron 9 of 12) | 102982 | C20orf26 |
| chr11-51 | chr11 | 30389593 | 30389743 | 53.5 | Intergenic | 45019 | ARL14EP |
| chr12-77 | chr12 | 88526335 | 88526485 | 53.5 | intron (NM_025114, intron 6 of 53) | 9583 | CEP290 |
| chr19-85 | chr19 | 49377356 | 49377506 | 53.5 | exon (NM_014330, exon 2 of 3) | 1782 | PPP1R15A |
| chr15-51 | chr15 | 72072734 | 72072884 | 53.5 | 3' UTR (NM_024817, exon 17 of 17) | -30085 | NR2E3 |
| chr7-62 | chr7 | 23312874 | 23313024 | 53.5 | intron (NM_001005340, intron 9 of 10) | -25991 | MALSU1 |
| chr2-116 | chr2 | 170751951 | 170752101 | 53.5 | intron (NM_172070, intron 7 of 38) | 68008 | UBR3 |
| chr14-32 | chr14 | 35519897 | 35520047 | 53.5 | intron (NM_173607, intron 1 of 4) | 4362 | FAM177A1 |
| chr1-90 | chr1 | 62212536 | 62212686 | 53.5 | intron (NM_176877, intron 1 of 42) | 4462 | INADL |
| chr6-43 | chr6 | 3105982 | 3106132 | 53.5 | exon (NM_003804, exon 8 of 10) | -12869 | BPHL |
| chr20-31 | chr20 | 22994186 | 22994336 | 53.5 | Intergenic | -21796 | SSTR4 |
| chr6-45 | chr6 | 17703677 | 17703827 | 53.5 | intron (NM_005124, intron 1 of 21) | 3066 | NUP153 |
| chr16-48 | chr16 | 14623035 | 14623185 | 53.5 | intron (NM_001134477, intron 21 of 23) | 101018 | PARN |
| chr1-82 | chr1 | 200333339 | 200333489 | 53.5 | intron (NR_040064, intron 4 of 4) | 9506 | C1orf98 |
| chr2-100 | chr2 | 25739735 | 25739885 | 53.5 | intron (NM_001256303, intron 9 of 19) | 133281 | DTNB |
| chr10-112 | chr10 | 124013379 | 124013529 | 53.5 | intron (NM_206862, intron 22 of 22) | -17367 | BTBD16 |
| chr7-45 | chr7 | 32551305 | 32551455 | 53.5 | intron (NM_015060, intron 1 of 15) | 16204 | AVL9 |
| chr8-51 | chr8 | 37605024 | 37605174 | 53.5 | non-coding (NR_003671, exon 1 of 1) | 465 | LOC728024 |
| chr17-72 | chr17 | 41797444 | 41797594 | 53.5 | Intergenic | 38637 | SOST |
| chr11-59 | chr11 | 6477277 | 6477427 | 53.5 | exon (NM_006458, exon 8 of 13) | -15098 | HPX |
| chr1-93 | chr1 | 153113221 | 153113371 | 53.5 | intron (NR_003062, intron 1 of 1) | 673 | SPRR2C |
| chr8-38 | chr8 | 32127218 | 32127368 | 53.5 | intron (NM_013962, intron 1 of 4) | -170969 | NRG1-IT3 |
| chr5-52 | chr5 | 59492431 | 59492581 | 53.5 | intron (NM_001165899, intron 1 of 16) | -291034 | PART1 |
| chr18-31 | chr18 | 68040383 | 68040533 | 53.5 | Intergenic | 84321 | SOCS6 |
| chr1-72 | chr1 | 22192080 | 22192230 | 53.5 | intron (NM_005529, intron 34 of 96) | 53397 | LDLRAD2 |
| chr2-92 | chr2 | 192082410 | 192082560 | 53.5 | Intergenic | -27622 | MYO1B |
| chr18-30 | chr18 | 50929115 | 50929265 | 53.5 | exon (NM_005215, exon 19 of 29) | 819592 | SNORA37 |
| chr9-58 | chr9 | 97629562 | 97629712 | 53.5 | intron (NM_032823, intron 5 of 14) | 57393 | MIR2278 |
| chr12-73 | chr12 | 55329214 | 55329364 | 53.5 | Intergenic | 38227 | TESPA1 |
| chr8-48 | chr8 | 24858141 | 24858291 | 53.5 | Intergenic | -44085 | NEFL |
| chr16-36 | chr16 | 72882661 | 72882811 | 53.5 | intron (NM_006885, intron 4 of 9) | 199538 | ZFHX3 |
| chr15-50 | chr15 | 27012337 | 27012487 | 53.5 | intron (NM_021912, intron 3 of 8) | 5811 | GABRB3 |
| chr12-74 | chr12 | 56144124 | 56144274 | 53.5 | 3' UTR (NM_005811, exon 3 of 3) | 7135 | GDF11 |
| chr1-100 | chr1 | 225616507 | 225616657 | 53.5 | promoter-TSS (NM_002296) | -25 | LBR |
| chr9-56 | chr9 | 90493942 | 90494092 | 53.5 | Intergenic | -3755 | FAM75E1 |
| chr8-37 | chr8 | 30580419 | 30580569 | 53.5 | intron (NM_000637, intron 1 of 12) | 4992 | GSR |
| chr13-14 | chr13 | 98828929 | 98829079 | 53.5 | exon (NM_178861, exon 1 of 2) | 517 | RNF113B |
| chr19-81 | chr19 | 36831764 | 36831914 | 53.5 | exon (NM_020917, exon 5 of 5) | -9219 | LINC00665 |
| chr2-91 | chr2 | 190413868 | 190414018 | 53.5 | Intergenic | 31594 | SLC40A1 |
| chr20-19 | chr20 | 634148 | 634298 | 53.5 | promoter-TSS (NM_080725) | -333 | SRXN1 |
| chr7-65 | chr7 | 77039642 | 77039792 | 53.5 | intron (NM_017439, intron 1 of 30) | 6000 | PION |
| chr16-54 | chr16 | 83986689 | 83986839 | 53.5 | promoter-TSS (NM_182981) | -63 | OSGIN1 |
| chr3-59 | chr3 | 170224417 | 170224567 | 53.5 | intron (NM_020949, intron 2 of 7) | 79371 | SLC7A14 |
| chr5-46 | chr5 | 43453899 | 43454049 | 53.5 | exon (NM_022483, exon 2 of 3) | 30018 | C5orf28 |
| chr17-65 | chr17 | 1437177 | 1437327 | 53.5 | intron (NM_006224, intron 10 of 11) | 17039 | PITPNA-AS1 |
| chr2-104 | chr2 | 54841227 | 54841377 | 53.5 | intron (NM_178313, intron 3 of 30) | 55771 | SPTBN1 |
| chr8-56 | chr8 | 104457853 | 104458003 | 53.5 | Intergenic | -30460 | SLC25A32 |
| chr17-82 | chr17 | 79887364 | 79887514 | 53.5 | non-coding (NR_015454, exon 2 of 2) | 1734 | MAFG-AS1 |
| chr14-38 | chr14 | 102428986 | 102429136 | 53.5 | Intergenic | -1804 | DYNC1H1 |
| chr19-83 | chr19 | 41172363 | 41172513 | 53.5 | 3' UTR (NM_004756, exon 10 of 10) | 24118 | NUMBL |
| chr12-70 | chr12 | 47549718 | 47549868 | 53.5 | Intergenic | -31802 | MIR4698 |
| chr2-95 | chr2 | 220252411 | 220252561 | 53.5 | exon (NM_012100, exon 1 of 15) | 176 | DNPEP |
| chr11-69 | chr11 | 124109107 | 124109257 | 53.5 | Intergenic | -11241 | OR8G1 |
| chr8-41 | chr8 | 95254618 | 95254768 | 53.5 | Intergenic | 19854 | GEM |
| chr18-25 | chr18 | 56456468 | 56456618 | 53.5 | Intergenic | -73518 | ZNF532 |
| chr14-27 | chr14 | 35843647 | 35843797 | 53.5 | Intergenic | 30238 | NFKBIA |
| chr6-48 | chr6 | 47779344 | 47779494 | 53.5 | non-coding (NR_033806, exon 6 of 7) | 24615 | OPN5 |
| chr19-69 | chr19 | 41426991 | 41427141 | 53.5 | Intergenic | -3104 | CYP2B7P1 |
| chr1-96 | chr1 | 182991561 | 182991711 | 53.5 | promoter-TSS (NM_002293) | -959 | LAMC1 |
| chr19-80 | chr19 | 30181931 | 30182081 | 53.5 | Intergenic | 23957 | C19orf12 |
| chr10-111 | chr10 | 119042770 | 119042920 | 53.5 | exon (NM_173791, exon 5 of 5) | 42261 | SLC18A2 |
| chrY-9 | chrY | 10031379 | 10031529 | 53.5 | Intergenic | 283047 | TTTY23 |
| chr15-47 | chr15 | 97300547 | 97300697 | 53.5 | Intergenic | -26057 | SPATA8 |
| chr3-54 | chr3 | 12647816 | 12647966 | 53.5 | intron (NM_002880, intron 5 of 16) | 49297 | MKRN2 |
| chr8-59 | chr8 | 131515502 | 131515652 | 53.5 | Intergenic | -59671 | ASAP1 |
| chr15-54 | chr15 | 99865042 | 99865192 | 53.5 | intron (NM_144598, intron 5 of 9) | 73465 | LRRC28 |
| chr2-83 | chr2 | 69309928 | 69310078 | 53.5 | intron (NM_053034, intron 8 of 14) | -20811 | MIR3126 |
| chr13-13 | chr13 | 33051227 | 33051377 | 53.5 | intron (NM_033111, intron 6 of 9) | 21327 | MINOS1P1 |
| chr17-77 | chr17 | 57920582 | 57920732 | 53.5 | Intergenic | 2030 | MIR21 |
| chr8-44 | chr8 | 128234955 | 128235105 | 53.5 | Intergenic | -192827 | POU5F1B |
| chr19-78 | chr19 | 22545458 | 22545608 | 53.5 | Intergenic | 59615 | ZNF98 |
| chr12-72 | chr12 | 50474978 | 50475128 | 53.5 | exon (NM_001095, exon 11 of 12) | -3930 | SMARCD1 |
| chr12-79 | chr12 | 98793467 | 98793617 | 53.5 | Intergenic | 57381 | SLC9A7P1 |
| chr6-47 | chr6 | 29393783 | 29393933 | 53.5 | 3' UTR (NM_013937, exon 1 of 1).6 | 1651 | OR11A1 |
| chr9-43 | chr9 | 38039310 | 38039460 | 53.5 | intron (NM_003028, intron 1 of 5) | 29825 | SHB |
| chr15-53 | chr15 | 75415820 | 75415970 | 53.5 | Intergenic | -78326 | C15orf39 |
| chr7-60 | chr7 | 158595312 | 158595462 | 53.5 | intron (NM_020728, intron 1 of 21) | 26932 | ESYT2 |
| chr13-15 | chr13 | 102280110 | 102280260 | 53.5 | intron (NM_004791, intron 7 of 10) | 175219 | ITGBL1 |
| chr7-49 | chr7 | 105043018 | 105043168 | 53.5 | Intergenic | -13752 | SRPK2 |
| chr19-62 | chr19 | 12780867 | 12781017 | 53.5 | promoter-TSS (NM_016145) | 425 | WDR83 |
| chr1-92 | chr1 | 94127758 | 94127908 | 53.5 | intron (NM_001261408, intron 4 of 13) | 19093 | BCAR3 |
| chr5-51 | chr5 | 20276631 | 20276781 | 53.5 | Intergenic | -288353 | CDH18 |
| chr1-76 | chr1 | 55119301 | 55119451 | 53.5 | non-coding (NR_037639, exon 3 of 33) | 11949 | HEATR8-TTC4 |
| chr8-57 | chr8 | 124642697 | 124642847 | 53.5 | Intergenic | 22418 | KLHL38 |
| chr11-64 | chr11 | 66717615 | 66717765 | 53.5 | intron (NM_000920, intron 2 of 21) | 8157 | PC |
| chr7-59 | chr7 | 135262440 | 135262590 | 53.5 | intron (NM_015135, intron 5 of 42) | 19853 | NUP205 |
| chr1-60 | chr1 | 27189964 | 27190114 | 53.5 | exon (NM_006142, exon 1 of 1) | 406 | SFN |
| chr15-49 | chr15 | 23045235 | 23045385 | 53.5 | 3' UTR (NM_144599, exon 5 of 5) | -10883 | NIPA2 |
| chr12-68 | chr12 | 130647499 | 130647649 | 53.5 | exon (NM_007197, exon 1 of 1) | 570 | FZD10 |
| chr1-89 | chr1 | 26564122 | 26564272 | 53.5 | intron (NM_022778, intron 1 of 13) | 3504 | CEP85 |
| chr5-50 | chr5 | 10683785 | 10683935 | 53.5 | intron (NM_004394, intron 2 of 3) | 77527 | DAP |
| chr17-61 | chr17 | 53167713 | 53167863 | 53.5 | intron (NM_178509, intron 16 of 17) | 121662 | STXBP4 |
| chr14-37 | chr14 | 81104962 | 81105112 | 53.5 | intron (NM_152446, intron 18 of 23) | 300847 | CEP128 |
| chr12-78 | chr12 | 88641120 | 88641270 | 53.5 | Intergenic | 105122 | TMTC3 |
| chr1-97 | chr1 | 183510201 | 183510351 | 53.5 | intron (NM_001174061, intron 12 of 21) | 49463 | NCF2 |
| chr12-75 | chr12 | 57377488 | 57377638 | 53.5 | Intergenic | -10792 | GPR182 |
| chr9-40 | chr9 | 21678066 | 21678216 | 53.5 | Intergenic | -118444 | MIR31HG |
| chr2-90 | chr2 | 179972841 | 179972991 | 53.5 | 3' UTR (NM_178123, exon 18 of 18) | -58130 | CCDC141 |
| chrX-17 | chrX | 26211919 | 26212069 | 53.5 | exon (NM_173523, exon 2 of 2) | 1437 | MAGEB6 |
| chr11-65 | chr11 | 66935702 | 66935852 | 53.5 | intron (NM_012308, intron 2 of 20) | 49037 | KDM2A |
| chr5-45 | chr5 | 16897372 | 16897522 | 53.5 | intron (NM_012334, intron 1 of 40) | 38938 | MYO10 |
| chr4-32 | chr4 | 14183067 | 14183217 | 53.5 | Intergenic | 69550 | LOC152742 |
| chr12-80 | chr12 | 116930790 | 116930940 | 53.5 | Intergenic | -40362 | LINC00173 |
| chr15-44 | chr15 | 48795961 | 48796111 | 53.5 | exon (NM_000138, exon 17 of 66) | 141949 | FBN1 |
| chr5-55 | chr5 | 121356395 | 121356545 | 53.5 | exon (NM_152546, exon 6 of 8) | 56448 | LOX |
| chr6-41 | chr6 | 132785150 | 132785300 | 53.5 | intron (NM_003569, intron 8 of 9) | 49112 | STX7 |
| chr13-16 | chr13 | 114290393 | 114290543 | 53.5 | intron (NM_007111, intron 9 of 11) | 22045 | ATP4B |
| chr3-55 | chr3 | 16344112 | 16344262 | 53.5 | exon (NM_138381, exon 8 of 9) | 37520 | OXNAD1 |
| chr3-56 | chr3 | 36749216 | 36749366 | 53.5 | Intergenic | 32061 | DCLK3 |
| chr20-36 | chr20 | 60877717 | 60877867 | 53.5 | promoter-TSS (NM_007002) | -235 | ADRM1 |
| chr17-78 | chr17 | 63060877 | 63061027 | 53.5 | Intergenic | -8032 | GNA13 |
| chrX-25 | chrX | 129398625 | 129398775 | 53.5 | intron (NM_017666, intron 1 of 18) | 4222 | ZNF280C |
| chr6-50 | chr6 | 56560595 | 56560745 | 53.5 | intron (NR_046940, intron 1 of 5) | -52976 | DST |
| chr1-91 | chr1 | 90127062 | 90127212 | 46.8 | intron (NM_032270, intron 1 of 2) | 28493 | LRRC8C |
| chr14-40 | chr14 | 30112686 | 30112836 | 46.8 | intron (NM_002742, intron 4 of 17) | 61847 | MIR548AI |
| chr12-87 | chr12 | 50963783 | 50963933 | 46.8 | intron (NM_173602, intron 1 of 37) | 65090 | DIP2B |
| chr8-54 | chr8 | 56814254 | 56814404 | 46.8 | intron (NM_002350, intron 1 of 12) | 21943 | LYN |
| chr17-92 | chr17 | 39802157 | 39802307 | 46.8 | Intergenic | -5781 | KRT42P |
| chr9-60 | chr9 | 137293595 | 137293745 | 46.8 | exon (NM_002957, exon 2 of 10) | 22413 | MIR4669 |
| chr5-72 | chr5 | 158521949 | 158522099 | 46.8 | intron (NM_024007, intron 4 of 15) | 4764 | EBF1 |
| chr15-70 | chr15 | 89495874 | 89496024 | 46.8 | Intergenic | -39286 | MFGE8 |
| chr19-98 | chr19 | 44570816 | 44570966 | 46.8 | exon (NM_013361, exon 5 of 5) | -5406 | ZNF284 |
| chr20-41 | chr20 | 6145780 | 6145930 | 46.8 | Intergenic | -41664 | FERMT1 |
| chr15-68 | chr15 | 71347950 | 71348100 | 46.8 | Intergenic | 59814 | CT62 |
| chr16-50 | chr16 | 51591012 | 51591162 | 46.8 | Intergenic | -405904 | SALL1 |
| chr3-75 | chr3 | 149299570 | 149299720 | 46.8 | intron (NM_001168280, intron 2 of 6) | 76167 | WWTR1 |
| chr16-59 | chr16 | 19938124 | 19938274 | 46.8 | Intergenic | -42048 | GPRC5B |
| chr11-70 | chr11 | 607697 | 607847 | 46.8 | exon (NM_020901, exon 14 of 18) | 7956 | IRF7 |
| chr1-109 | chr1 | 43288925 | 43289075 | 46.8 | intron (NM_001017922, intron 1 of 11) | -2249 | ERMAP |
| chr6-61 | chr6 | 75794762 | 75794912 | 46.8 | 3' UTR (NM_080645, exon 51 of 51) | 120786 | COL12A1 |
| chr18-23 | chr18 | 11857214 | 11857364 | 46.8 | promoter-TSS (NM_001261444) | -148 | GNAL |
| chr1-121 | chr1 | 111890395 | 111890545 | 46.8 | intron (NM_181643, intron 3 of 5) | 1275 | PIFO |
| chr12-97 | chr12 | 122238876 | 122239026 | 46.8 | intron (NR_002809, intron 4 of 6) | 2439 | LOC338799 |
| chr19-86 | chr19 | 53669146 | 53669296 | 46.8 | exon (NM_024733, exon 4 of 4) | -6899 | ZNF347 |
| chr20-27 | chr20 | 3293759 | 3293909 | 46.8 | intron (NM_001009984, intron 20 of 36) | -73947 | SLC4A11 |
| chr2-136 | chr2 | 110316172 | 110316322 | 46.8 | intron (NR_047585, intron 6 of 8) | 55536 | 10-Sep |
| chr20-37 | chr20 | 60883860 | 60884010 | 46.8 | TTS (NM_007002) | 5908 | ADRM1 |
| chr12-84 | chr12 | 45609526 | 45609676 | 46.8 | promoter-TSS (NM_001142679) | -169 | ANO6 |
| chr4-50 | chr4 | 140521332 | 140521482 | 46.8 | Intergenic | -43830 | SETD7 |
| chr7-70 | chr7 | 29024284 | 29024434 | 46.8 | intron (NR_038965, intron 1 of 4) | 4776 | LOC100506497 |
| chr2-142 | chr2 | 203830577 | 203830727 | 46.8 | intron (NM_024744, intron 9 of 16) | -48950 | NBEAL1 |
| chr10-123 | chr10 | 74397244 | 74397394 | 46.8 | Intergenic | -11370 | MICU1 |
| chr10-108 | chr10 | 89465142 | 89465292 | 46.8 | intron (NM_001015880, intron 1 of 12) | 45741 | PAPSS2 |
| chr8-70 | chr8 | 92330442 | 92330592 | 46.8 | exon (NM_052832, exon 5 of 19) | 69001 | SLC26A7 |
| chr15-56 | chr15 | 34540470 | 34540620 | 46.8 | intron (NM_001042497, intron 11 of 23) | 23300 | EMC4 |
| chr18-32 | chr18 | 3385037 | 3385187 | 46.8 | Intergenic | -26960 | TGIF1 |
| chr15-64 | chr15 | 59568985 | 59569135 | 46.8 | intron (NM_004998, intron 1 of 27) | 70045 | LDHAL6B |
| chr7-75 | chr7 | 76018415 | 76018565 | 46.8 | TTS (NM_080744) | -8351 | ZP3 |
| chr19-94 | chr19 | 16013864 | 16014014 | 46.8 | Intergenic | -5055 | CYP4F2 |
| chr7-74 | chr7 | 76002636 | 76002786 | 46.8 | Intergenic | -14369 | YWHAG |
| chr1-125 | chr1 | 161018073 | 161018223 | 46.8 | exon (NM_001025598, exon 12 of 12) | -2391 | USF1 |
| chr6-46 | chr6 | 24659312 | 24659462 | 46.8 | intron (NM_016614, intron 2 of 6) | 7728 | TDP2 |
| chr8-50 | chr8 | 37470234 | 37470384 | 46.8 | Intergenic | -82992 | ZNF703 |
| chr11-74 | chr11 | 28031063 | 28031213 | 46.8 | Intergenic | -47224 | MIR610 |
| chr6-53 | chr6 | 29430162 | 29430312 | 46.8 | exon (NM_030883, exon 3 of 3).3 | 4007 | OR2H1 |
| chr12-76 | chr12 | 65190064 | 65190214 | 46.8 | Intergenic | -28213 | TBC1D30 |
| chr7-81 | chr7 | 130569835 | 130569985 | 46.8 | intron (NR_034120, intron 2 of 3) | -7612 | MIR29B1 |
| chr2-145 | chr2 | 228280413 | 228280563 | 46.8 | Intergenic | -36466 | TM4SF20 |
| chr6-49 | chr6 | 53659960 | 53660110 | 46.8 | 5' UTR (NM_018214, exon 1 of 14) | 257 | LRRC1 |
| chr17-80 | chr17 | 75211781 | 75211931 | 46.8 | 3' UTR (NM_001143999, exon 17 of 17) | 30561 | SEC14L1 |
| chr11-73 | chr11 | 18600800 | 18600950 | 46.8 | intron (NM_001040697, intron 1 of 11) | 190 | UEVLD |
| chr14-48 | chr14 | 77422978 | 77423128 | 46.8 | Intergenic | 71989 | IRF2BPL |
| chr9-59 | chr9 | 131698778 | 131698928 | 46.8 | intron (NM_001100877, intron 5 of 9) | -11124 | NUP188 |
| chr4-49 | chr4 | 139163557 | 139163707 | 46.8 | promoter-TSS (NM_014331) | -129 | SLC7A11 |
| chr14-36 | chr14 | 76002179 | 76002329 | 46.8 | intron (NM_006399, intron 2 of 2) | 13470 | BATF |
| chr12-88 | chr12 | 52167344 | 52167494 | 46.8 | intron (NM_014191, intron 19 of 26) | 58282 | FIGNL2 |
| chr1-106 | chr1 | 16107303 | 16107453 | 46.8 | intron (NM_001024216, intron 4 of 4) | 15919 | FBLIM1 |
| chr11-78 | chr11 | 47968599 | 47968749 | 46.8 | Intergenic | -33436 | PTPRJ |
| chr1-133 | chr1 | 227231541 | 227231691 | 46.8 | intron (NM_003607, intron 22 of 35) | 103678 | ADCK3 |
| chr10-113 | chr10 | 5326524 | 5326674 | 46.8 | Intergenic | -80377 | UCN3 |
| chr17-102 | chr17 | 78201560 | 78201710 | 46.8 | exon (NM_001166347, exon 7 of 18) | 7435 | SLC26A11 |
| chr6-64 | chr6 | 114056480 | 114056630 | 46.8 | Intergenic | -121972 | MARCKS |
| chr8-74 | chr8 | 108508183 | 108508333 | 46.8 | intron (NM_001146, intron 1 of 8) | 1996 | ANGPT1 |
| chr1-130 | chr1 | 207081327 | 207081477 | 46.8 | intron (NM_005449, intron 7 of 7) | 10614 | IL24 |
| chr5-57 | chr5 | 5340632 | 5340782 | 46.8 | Intergenic | -82079 | KIAA0947 |
| chr7-72 | chr7 | 39504725 | 39504875 | 46.8 | TTS (NM_001166018) | -58855 | POU6F2-AS1 |
| chr10-122 | chr10 | 71206507 | 71206657 | 46.8 | Intergenic | -4644 | TSPAN15 |
| chr1-119 | chr1 | 95407779 | 95407929 | 46.8 | intron (NR_033998, intron 2 of 3) | 14270 | LOC729970 |
| chr6-60 | chr6 | 74290141 | 74290291 | 46.8 | Intergenic | -59461 | EEF1A1 |
| chr19-97 | chr19 | 44039303 | 44039453 | 46.8 | exon (NM_174945, exon 4 of 4) | 2038 | ZNF575 |
| chr1-116 | chr1 | 78005572 | 78005722 | 46.8 | intron (NM_174858, intron 13 of 13) | 142696 | ZZZ3 |
| chr17-89 | chr17 | 33390700 | 33390850 | 46.8 | promoter-TSS (NM_001017368) | -16 | RFFL |
| chr14-51 | chr14 | 94394612 | 94394762 | 46.8 | exon (NM_138344, exon 3 of 3) | 956 | FAM181A |
| chr18-37 | chr18 | 32527719 | 32527869 | 46.8 | Intergenic | -29098 | MAPRE2 |
| chrX-27 | chrX | 22154497 | 22154647 | 46.8 | intron (NM_000444, intron 12 of 21) | 36528 | PHEX-AS1 |
| chr3-60 | chr3 | 189110821 | 189110971 | 46.8 | Intergenic | -152513 | TPRG1-AS2 |
| chr16-58 | chr16 | 9141820 | 9141970 | 46.8 | Intergenic | -43642 | C16orf72 |
| chr9-49 | chr9 | 33320506 | 33320656 | 46.8 | intron (NM_147133, intron 9 of 20) | 30163 | NFX1 |
| chr8-65 | chr8 | 32962636 | 32962786 | 46.8 | Intergenic | 367953 | FUT10 |
| chr9-57 | chr9 | 93094577 | 93094727 | 46.8 | Intergenic | 250376 | LOC340515 |
| chr9-72 | chr9 | 103493946 | 103494096 | 46.8 | Intergenic | 153685 | MURC |
| chr10-118 | chr10 | 32871940 | 32872090 | 46.8 | 5' UTR (NM_024688, exon 2 of 23) | 15364 | C10orf68 |
| chr2-124 | chr2 | 27929150 | 27929300 | 46.8 | Intergenic | -42776 | SUPT7L |
| chr4-47 | chr4 | 95882697 | 95882847 | 46.8 | intron (NM_001203, intron 2 of 12) | -34611 | BMPR1B |
| chr9-69 | chr9 | 75799506 | 75799656 | 46.8 | Intergenic | 32800 | ANXA1 |
| chr6-55 | chr6 | 34212365 | 34212515 | 46.8 | intron (NM_145902, intron 4 of 4) | 4464 | C6orf1 |
| chr5-68 | chr5 | 144967315 | 144967465 | 46.8 | Intergenic | 247477 | PRELID2 |
| chr2-106 | chr2 | 70752353 | 70752503 | 46.8 | intron (NM_003236, intron 1 of 5) | 28719 | TGFA |
| chr4-48 | chr4 | 103560637 | 103560787 | 46.8 | intron (NM_005908, intron 14 of 16) | 121439 | MANBA |
| chr2-130 | chr2 | 72393066 | 72393216 | 46.8 | Intergenic | -18178 | CYP26B1 |
| chr7-61 | chr7 | 21698571 | 21698721 | 46.8 | exon (NM_003777, exon 30 of 83) | 115813 | DNAH11 |
| chr11-75 | chr11 | 34066672 | 34066822 | 46.8 | Intergenic | -6483 | CAPRIN1 |
| chr9-67 | chr9 | 75172588 | 75172738 | 46.8 | intron (NM_138691, intron 1 of 23) | 35946 | TMC1 |
| chr10-128 | chr10 | 125456415 | 125456565 | 46.8 | 3' UTR (NM_153442, exon 3 of 3) | 30619 | GPR26 |
| chr1-104 | chr1 | 8100777 | 8100927 | 46.8 | Intergenic | -14459 | ERRFI1 |
| chr11-88 | chr11 | 86454580 | 86454730 | 46.8 | Intergenic | -56836 | PRSS23 |
| chr9-75 | chr9 | 123605173 | 123605323 | 46.8 | promoter-TSS (NM_001270427) | 51 | PSMD5 |
| chr11-84 | chr11 | 69235421 | 69235571 | 46.8 | Intergenic | 173874 | MYEOV |
| chr19-90 | chr19 | 3434346 | 3434496 | 46.8 | intron (NM_205843, intron 5 of 10) | 46119 | C19orf77 |
| chr12-85 | chr12 | 47639503 | 47639653 | 46.8 | Intergenic | -29352 | PCED1B-AS1 |
| chr20-45 | chr20 | 46018955 | 46019105 | 46.8 | Intergenic | -33556 | ZMYND8 |
| chr18-26 | chr18 | 3603546 | 3603696 | 46.8 | intron (NM_001003809, intron 4 of 9) | 9509 | DLGAP1-AS1 |
| chr7-78 | chr7 | 84154391 | 84154541 | 46.8 | Intergenic | -330249 | SEMA3A |
| chr1-88 | chr1 | 21048023 | 21048173 | 46.8 | 3' UTR (NM_001103160, exon 9 of 9) | -3781 | KIF17 |
| chr2-121 | chr2 | 234652218 | 234652368 | 46.8 | exon (NM_001001394, exon 1 of 1) | 368 | DNAJB3 |
| chr1-111 | chr1 | 51253729 | 51253879 | 46.8 | exon (NM_007051, exon 4 of 19) | 172132 | FAF1 |
| chr17-69 | chr17 | 37642014 | 37642164 | 46.8 | intron (NM_016507, intron 2 of 13) | 24350 | CDK12 |
| chrX-29 | chrX | 66764679 | 66764829 | 46.8 | 5' UTR (NM_000044, exon 1 of 8) | 880 | AR |
| chr14-35 | chr14 | 71165113 | 71165263 | 46.8 | Intergenic | 56684 | TTC9 |
| chr10-114 | chr10 | 6281281 | 6281431 | 46.8 | Intergenic | 36516 | PFKFB3 |
| chr1-124 | chr1 | 160783125 | 160783275 | 46.8 | intron (NM_001261456, intron 2 of 9) | 17336 | LY9 |
| chr12-94 | chr12 | 94018835 | 94018985 | 46.8 | Intergenic | -52241 | CRADD |
| chr9-62 | chr9 | 6681622 | 6681772 | 46.8 | Intergenic | -36005 | GLDC |
| chr19-84 | chr19 | 49375450 | 49375600 | 46.8 | promoter-TSS (NM_014330) | -124 | PPP1R15A |
| chr4-39 | chr4 | 52719383 | 52719533 | 46.8 | intron (NM_001040402, intron 1 of 10) | 10182 | DCUN1D4 |
| chr17-81 | chr17 | 75283136 | 75283286 | 46.8 | promoter-TSS (NM_001113492) | -762 | 9-Sep |
| chr14-42 | chr14 | 54317786 | 54317936 | 46.8 | Intergenic | 97341 | MIR5580 |
| chr3-74 | chr3 | 149253190 | 149253340 | 46.8 | intron (NM_001168280, intron 4 of 6) | 60897 | TM4SF4 |
| chr15-55 | chr15 | 34529575 | 34529725 | 46.8 | exon (NM_001042494, exon 22 of 26) | 12405 | EMC4 |
| chr12-90 | chr12 | 63179851 | 63180001 | 46.8 | intron (NM_020700, intron 4 of 9) | 148739 | PPM1H |
| chr15-71 | chr15 | 102312729 | 102312879 | 46.8 | Intergenic | -33119 | OR4F6 |
| chr19-93 | chr19 | 12090539 | 12090689 | 46.8 | 3' UTR (NM_001012753, exon 4 of 4) | 14745 | ZNF763 |
| chr18-29 | chr18 | 24057904 | 24058054 | 46.8 | intron (NM_001258221, intron 2 of 4) | 70521 | KCTD1 |
| chr9-68 | chr9 | 75688659 | 75688809 | 46.8 | Intergenic | -78047 | ANXA1 |
| chr16-66 | chr16 | 71526095 | 71526245 | 46.8 | Intergenic | -2916 | ZNF19 |
| chr19-88 | chr19 | 55053571 | 55053721 | 46.8 | intron (NR_026716, intron 6 of 8) | 9737 | KIR3DX1 |
| chr18-36 | chr18 | 30092650 | 30092800 | 46.8 | non-coding (NR_003558, exon 1 of 1) | 1099 | WBP11P1 |
| chr12-71 | chr12 | 49351215 | 49351365 | 46.8 | promoter-TSS (NM_001659) | -38 | ARF3 |
| chr10-125 | chr10 | 112185734 | 112185884 | 46.8 | Intergenic | -71816 | DUSP5 |
| chr17-96 | chr17 | 51220427 | 51220577 | 46.8 | Intergenic | 157622 | C17orf112 |
| chr20-39 | chr20 | 4554888 | 4555038 | 46.8 | Intergenic | -111834 | PRNP |
| chr12-82 | chr12 | 13145495 | 13145645 | 46.8 | intron (NM_015987, intron 1 of 3) | 7673 | HEBP1 |
| chr15-61 | chr15 | 45929892 | 45930042 | 46.8 | intron (NM_021199, intron 1 of 9) | 3029 | SQRDL |
| chr20-34 | chr20 | 42832945 | 42833095 | 46.8 | intron (NM_016470, intron 2 of 3) | 6526 | C20orf111 |
| chr2-128 | chr2 | 69244347 | 69244497 | 46.8 | intron (NM_053034, intron 1 of 14) | 4146 | ANTXR1 |
| chr19-95 | chr19 | 32864823 | 32864973 | 46.8 | intron (NM_014910, intron 4 of 5) | 28384 | ZNF507 |
| chr1-131 | chr1 | 209572261 | 209572411 | 46.8 | Intergenic | -29832 | MIR205HG |
| chr5-56 | chr5 | 169761155 | 169761305 | 46.8 | non-coding (NR_026945, exon 3 of 3) | 2795 | LOC257358 |
| chr17-75 | chr17 | 53045992 | 53046142 | 46.8 | promoter-TSS (NM_001162861) | -3 | COX11 |
| chr4-43 | chr4 | 88896098 | 88896248 | 46.8 | promoter-TSS (NM_000582) | -629 | SPP1 |
| chr14-52 | chr14 | 102452592 | 102452742 | 46.8 | exon (NM_001376, exon 8 of 78) | 21802 | DYNC1H1 |
| chr8-72 | chr8 | 102038580 | 102038730 | 46.8 | Intergenic | -25627 | FLJ42969 |
| chr2-113 | chr2 | 106731852 | 106732002 | 46.8 | intron (NR_045607, intron 8 of 13) | 23410 | UXS1 |
| chr14-49 | chr14 | 77494104 | 77494254 | 46.8 | 5' UTR (NM_024496, exon 1 of 1) | 863 | IRF2BPL |
| chr7-77 | chr7 | 83097694 | 83097844 | 46.8 | intron (NM_012431, intron 3 of 16) | 172978 | SEMA3E |
| chr13-20 | chr13 | 74761796 | 74761946 | 46.8 | Intergenic | -53805 | KLF12 |
| chr2-140 | chr2 | 192815299 | 192815449 | 46.8 | intron (NM_016192, intron 9 of 9) | -103368 | SDPR |
| chr6-56 | chr6 | 36060482 | 36060632 | 46.8 | intron (NM_001315, intron 8 of 11) | -37704 | MAPK13 |
| chr9-53 | chr9 | 84150525 | 84150675 | 46.8 | Intergenic | 152996 | TLE1 |
| chr9-73 | chr9 | 108266307 | 108266457 | 46.8 | intron (NM_001145313, intron 7 of 13) | -54029 | FKTN |
| chr3-71 | chr3 | 145884368 | 145884518 | 46.8 | Intergenic | -5161 | PLOD2 |
| chr1-127 | chr1 | 186685458 | 186685608 | 46.8 | Intergenic | -35974 | PTGS2 |
| chr3-73 | chr3 | 149104850 | 149105000 | 46.8 | Intergenic | -9357 | TM4SF1 |
| chr1-117 | chr1 | 84569151 | 84569301 | 46.8 | intron (NM_207578, intron 1 of 8) | 25481 | PRKACB |
| chr3-78 | chr3 | 177527960 | 177528110 | 46.8 | Intergenic | 368326 | LINC00578 |
| chr11-76 | chr11 | 34448988 | 34449138 | 46.8 | Intergenic | -11409 | CAT |
| chr2-138 | chr2 | 127822443 | 127822593 | 46.8 | intron (NM_139349, intron 6 of 14) | 42385 | BIN1 |
| chr10-106 | chr10 | 70091669 | 70091819 | 46.8 | promoter-TSS (NM_012207) | -24 | HNRNPH3 |
| chr12-86 | chr12 | 47665235 | 47665385 | 46.8 | Intergenic | -55084 | PCED1B-AS1 |
| chr20-43 | chr20 | 33721629 | 33721779 | 46.8 | intron (NR_026728, intron 5 of 9) | 13457 | EDEM2 |
| chr19-73 | chr19 | 7671440 | 7671590 | 46.8 | intron (NM_020902, intron 4 of 16) | 10727 | CAMSAP3 |
| chr12-95 | chr12 | 96757968 | 96758118 | 46.8 | intron (NM_002595, intron 1 of 16) | 36323 | CDK17 |
| chr2-122 | chr2 | 2567475 | 2567625 | 46.8 | Intergenic | -232505 | MYT1L |
| chr2-108 | chr2 | 73291090 | 73291240 | 46.8 | intron (NM_144579, intron 1 of 13) | 7800 | SFXN5 |
| chr2-125 | chr2 | 28499100 | 28499250 | 46.8 | intron (NM_001261840, intron 10 of 11) | 34151 | LOC100505716 |
| chr6-66 | chr6 | 138002223 | 138002373 | 46.8 | Intergenic | -186027 | TNFAIP3 |
| chr8-76 | chr8 | 134306612 | 134306762 | 46.8 | intron (NM_001258433, intron 1 of 14) | 2860 | NDRG1 |
| chr18-34 | chr18 | 21300004 | 21300154 | 46.8 | intron (NM_198129, intron 2 of 74) | 30517 | LAMA3 |
| chr5-75 | chr5 | 173396509 | 173396659 | 46.8 | Intergenic | -19578 | C5orf47 |
| chr17-100 | chr17 | 62631919 | 62632069 | 46.8 | intron (NM_022739, intron 1 of 18) | 26392 | SMURF2 |
| chr8-52 | chr8 | 38385739 | 38385889 | 46.8 | intron (NM_207412, intron 1 of 2) | 366 | C8orf86 |
| chr8-62 | chr8 | 18818759 | 18818909 | 46.8 | intron (NM_015310, intron 1 of 15) | 52362 | PSD3 |
| chr3-77 | chr3 | 158892623 | 158892773 | 46.8 | intron (NM_001042706, intron 1 of 3) | -98338 | SCHIP1 |
| chr1-108 | chr1 | 27175831 | 27175981 | 46.8 | intron (NM_032283, intron 3 of 7) | -13727 | SFN |
| chr11-83 | chr11 | 67147185 | 67147335 | 46.8 | intron (NR_024469, intron 2 of 3) | -5612 | CLCF1 |
| chr11-61 | chr11 | 15991065 | 15991215 | 46.8 | 3' UTR (NM_001145819, exon 16 of 16) | 433273 | SOX6 |
| chr17-94 | chr17 | 47269049 | 47269199 | 46.8 | Intergenic | 17619 | GNGT2 |
| chr1-128 | chr1 | 196047933 | 196048083 | 46.8 | Intergenic | 503603 | MIR4735 |
| chr2-131 | chr2 | 74902884 | 74903034 | 46.8 | exon (NM_004263, exon 12 of 14) | 21566 | SEMA4F |
| chr8-73 | chr8 | 107072677 | 107072827 | 46.8 | Intergenic | -209654 | OXR1 |
| chr16-60 | chr16 | 30411980 | 30412130 | 46.8 | TTS (NM_152652) | 5315 | ZNF48 |
| chr3-69 | chr3 | 121838394 | 121838544 | 46.8 | 3' UTR (NM_176892, exon 6 of 6) | 41773 | CD86 |
| chr1-112 | chr1 | 62330087 | 62330237 | 46.8 | exon (NM_176877, exon 20 of 43) | 122013 | INADL |
| chr4-30 | chr4 | 8230425 | 8230575 | 46.8 | intron (NM_018986, intron 12 of 17) | 29440 | SH3TC1 |
| chr10-127 | chr10 | 120718729 | 120718879 | 46.8 | Intergenic | -70424 | NANOS1 |
| chr16-52 | chr16 | 73104514 | 73104664 | 46.8 | Intergenic | -12055 | ZFHX3 |
| chr20-30 | chr20 | 22370963 | 22371113 | 46.8 | Intergenic | 30243 | LOC284788 |
| chr4-31 | chr4 | 8305364 | 8305514 | 46.8 | intron (NM_053044, intron 7 of 8) | 33950 | HTRA3 |
| chr15-66 | chr15 | 60666927 | 60667077 | 46.8 | intron (NM_001002857, intron 4 of 13) | 23183 | ANXA2 |
| chr11-90 | chr11 | 95658282 | 95658432 | 46.8 | promoter-TSS (NM_201278) | -986 | MTMR2 |
| chr2-129 | chr2 | 70315323 | 70315473 | 46.8 | exon (NM_006196, exon 1 of 1) | 813 | PCBP1 |
| chr14-34 | chr14 | 62129267 | 62129417 | 46.8 | Intergenic | -32777 | HIF1A |
| chr8-71 | chr8 | 101724795 | 101724945 | 46.8 | intron (NM_002568, intron 6 of 14) | 9445 | PABPC1 |
| chr17-95 | chr17 | 48650104 | 48650254 | 46.8 | exon (NM_001256333, exon 6 of 37) | -10710 | CACNA1G-AS1 |
| chr15-58 | chr15 | 40212651 | 40212801 | 46.8 | 5' UTR (NM_007223, exon 1 of 3) | 367 | GPR176 |
| chr14-50 | chr14 | 89467025 | 89467175 | 46.8 | Intergenic | 176122 | TTC8 |
| chr17-97 | chr17 | 57696913 | 57697063 | 46.8 | promoter-TSS (NM_004859) | -62 | CLTC |
| chr2-141 | chr2 | 201992532 | 201992682 | 46.8 | intron (NM_001127183, intron 1 of 9) | -5081 | CFLAR |
| chr6-57 | chr6 | 41929510 | 41929660 | 46.8 | intron (NM_001136017, intron 1 of 4) | -20033 | CCND3 |
| chr16-65 | chr16 | 69970274 | 69970424 | 46.8 | exon (NM_001270453, exon 16 of 21) | 3365 | MIR140 |
| chr1-126 | chr1 | 172876519 | 172876669 | 46.8 | Intergenic | 143509 | TNFSF18 |
| chr17-68 | chr17 | 35486188 | 35486338 | 46.8 | intron (NM_198837, intron 45 of 53) | 95221 | MIR2909 |
| chr10-109 | chr10 | 101544978 | 101545128 | 46.8 | intron (NM_000392, intron 2 of 31) | 2590 | ABCC2 |
| chr6-62 | chr6 | 84140835 | 84140985 | 46.8 | promoter-TSS (NM_002395) | 28 | ME1 |
| chr2-126 | chr2 | 46976270 | 46976420 | 46.8 | intron (NM_144949, intron 1 of 1) | 49996 | SOCS5 |
| chr9-55 | chr9 | 90328383 | 90328533 | 46.8 | Intergenic | -12516 | CTSL1 |
| chr2-132 | chr2 | 85664827 | 85664977 | 46.8 | TTS (NM_198482) | 2984 | SH2D6 |
| chr15-69 | chr15 | 77327616 | 77327766 | 46.8 | intron (NM_003978, intron 12 of 14) | 35879 | TSPAN3 |
| chr19-99 | chr19 | 45535984 | 45536134 | 46.8 | intron (NM_006509, intron 8 of 10) | -6239 | CLASRP |
| chr17-74 | chr17 | 47411304 | 47411454 | 46.8 | intron (NM_014897, intron 1 of 5) | 28097 | ZNF652 |
| chr8-78 | chr8 | 144911512 | 144911662 | 46.8 | promoter-TSS (NM_001136033) | -31 | PUF60 |
| chr7-56 | chr7 | 68171255 | 68171405 | 46.8 | Intergenic | -892575 | AUTS2 |
| chr8-49 | chr8 | 27474659 | 27474809 | 46.8 | Intergenic | -2406 | CLU |
| chr8-67 | chr8 | 40062597 | 40062747 | 46.8 | Intergenic | 51685 | C8orf4 |
| chr2-118 | chr2 | 225956213 | 225956363 | 46.8 | Intergenic | -48958 | DOCK10 |
| chr21-18 | chr21 | 44841139 | 44841289 | 46.8 | exon (NM_173354, exon 6 of 14) | 5788 | SIK1 |
| chr7-79 | chr7 | 99841198 | 99841348 | 46.8 | intron (NM_178831, intron 1 of 4) | 24402 | PVRIG |
| chr7-68 | chr7 | 27497894 | 27498044 | 46.8 | Intergenic | 204651 | HIBADH |
| chr1-95 | chr1 | 167517712 | 167517862 | 46.8 | intron (NM_003851, intron 1 of 3) | 5269 | CREG1 |
| chr19-79 | chr19 | 30179290 | 30179440 | 46.8 | Intergenic | 23038 | PLEKHF1 |
| chr19-74 | chr19 | 13949923 | 13950073 | 46.8 | Intergenic | -2525 | MIR23A |
| chr2-123 | chr2 | 24014071 | 24014221 | 46.8 | intron (NM_017552, intron 19 of 27) | 135790 | ATAD2B |
| chr15-57 | chr15 | 34787567 | 34787717 | 46.8 | Intergenic | 32930 | MIR1233-1 |
| chr2-117 | chr2 | 214116409 | 214116559 | 46.8 | Intergenic | 32445 | LOC100130451 |
| chr8-75 | chr8 | 110476919 | 110477069 | 46.8 | exon (NM_177531, exon 49 of 78) | -74935 | EBAG9 |
| chr17-101 | chr17 | 77907706 | 77907856 | 46.8 | Intergenic | -94568 | CBX4 |
| chr10-110 | chr10 | 105576327 | 105576477 | 46.8 | intron (NM_014631, intron 1 of 13) | 38762 | SH3PXD2A |
| chr18-33 | chr18 | 20562369 | 20562519 | 46.8 | intron (NM_203292, intron 7 of 17) | 48605 | RBBP8 |
| chr18-27 | chr18 | 3624044 | 3624194 | 46.8 | intron (NM_001003809, intron 4 of 9) | 30007 | DLGAP1-AS1 |
| chr11-68 | chr11 | 75150928 | 75151078 | 46.8 | exon (NM_030792, exon 15 of 17) | -9329 | KLHL35 |
| chr12-92 | chr12 | 87106252 | 87106402 | 46.8 | intron (NM_013244, intron 1 of 6) | 126354 | MGAT4C |
| chr3-76 | chr3 | 156499468 | 156499618 | 46.8 | intron (NR_038387, intron 1 of 2) | 30267 | PA2G4P4 |
| chr13-22 | chr13 | 91570896 | 91571046 | 46.8 | intron (NR_027039, intron 1 of 5) | 7880 | LINC00410 |
| chr12-96 | chr12 | 98991694 | 98991844 | 46.8 | exon (NM_005888, exon 4 of 8) | -1644 | SNORA53 |
| chr2-144 | chr2 | 210360895 | 210361045 | 46.8 | intron (NM_001039538, intron 1 of 14) | 72199 | MAP2 |
| chr20-38 | chr20 | 3894735 | 3894885 | 46.8 | intron (NM_024960, intron 4 of 6) | -3331 | MIR103A2 |
| chr10-124 | chr10 | 79587296 | 79587446 | 46.8 | intron (NM_004747, intron 13 of 31) | 98977 | DLG5 |
| chr6-52 | chr6 | 10759655 | 10759805 | 46.8 | Intergenic | 11735 | TMEM14B |
| chr22-21 | chr22 | 35789451 | 35789601 | 46.8 | exon (NM_002133, exon 5 of 5) | -6590 | MCM5 |
| chr15-52 | chr15 | 72668764 | 72668914 | 46.8 | promoter-TSS (NM_000520) | -319 | HEXA |
| chr9-65 | chr9 | 35869475 | 35869625 | 46.8 | TTS (NM_001004487) | 848 | OR13J1 |
| chr7-71 | chr7 | 32919121 | 32919271 | 46.8 | exon (NM_015483, exon 2 of 4) | 12272 | KBTBD2 |
| chr8-61 | chr8 | 1703868 | 1704018 | 46.8 | Intergenic | -7927 | CLN8 |
| chr7-76 | chr7 | 80571529 | 80571679 | 46.8 | Intergenic | -22937 | SEMA3C |
| chr5-73 | chr5 | 169283698 | 169283848 | 46.8 | intron (NM_004946, intron 27 of 51) | 123971 | FAM196B |
| chr11-82 | chr11 | 65666832 | 65666982 | 46.8 | intron (NM_005438, intron 1 of 3) | 1090 | FOSL1 |
| chr9-70 | chr9 | 85550064 | 85550214 | 46.8 | Intergenic | 127904 | RASEF |
| chr11-72 | chr11 | 9484792 | 9484942 | 46.8 | intron (NM_003442, intron 1 of 15) | 2355 | ZNF143 |
| chr2-99 | chr2 | 7320847 | 7320997 | 46.8 | Intergenic | -240470 | LOC100506274 |
| chr14-47 | chr14 | 77385246 | 77385396 | 46.8 | Intergenic | 92596 | C14orf166B |
| chr2-102 | chr2 | 26699996 | 26700146 | 46.8 | exon (NM_194248, exon 21 of 47) | 839 | OTOF |
| chr3-105 | chr3 | 153007262 | 153007412 | 40.2 | Intergenic | 127308 | RAP2B |
| chr1-129 | chr1 | 206904640 | 206904790 | 40.2 | intron (NM_004759, intron 7 of 9) | 41124 | IL10 |
| chr7-107 | chr7 | 130877852 | 130878002 | 40.2 | intron (NM_001145354, intron 2 of 18) | 83072 | MKLN1 |
| chr1-150 | chr1 | 85724994 | 85725144 | 40.2 | non-coding (NR_024113, exon 1 of 4) | 286 | C1orf52 |
| chr8-64 | chr8 | 29994868 | 29995018 | 40.2 | exon (NM_001128208, exon 4 of 4) | 7257 | MBOAT4 |
| chr1-157 | chr1 | 145652454 | 145652604 | 40.2 | intron (NM_014455, intron 3 of 8) | 41493 | RNF115 |
| chrX-35 | chrX | 46193198 | 46193348 | 40.2 | Intergenic | -113351 | ZNF673 |
| chr17-116 | chr17 | 39616323 | 39616473 | 40.2 | exon (NM_002278, exon 7 of 7) | 7240 | KRT32 |
| chr16-86 | chr16 | 72992672 | 72992822 | 40.2 | exon (NM_006885, exon 2 of 10) | 89527 | ZFHX3 |
| chr9-71 | chr9 | 98220423 | 98220573 | 40.2 | exon (NM_000264, exon 18 of 24) | -5393 | LOC100507346 |
| chr15-86 | chr15 | 71704682 | 71704832 | 40.2 | intron (NM_024817, intron 6 of 16) | 270969 | THSD4 |
| chr1-170 | chr1 | 180260330 | 180260480 | 40.2 | intron (NM_032360, intron 7 of 7) | -16589 | LOC100527964 |
| chr20-54 | chr20 | 34755774 | 34755924 | 40.2 | intron (NM_012156, intron 1 of 21) | 13187 | EPB41L1 |
| chr4-57 | chr4 | 37636521 | 37636671 | 40.2 | exon (NM_001085400, exon 5 of 7) | 50741 | C4orf19 |
| chr4-72 | chr4 | 177800638 | 177800788 | 40.2 | Intergenic | -86818 | VEGFC |
| chr7-98 | chr7 | 87000084 | 87000234 | 40.2 | intron (NM_001143935, intron 8 of 18) | 25208 | CROT |
| chr2-153 | chr2 | 26235408 | 26235558 | 40.2 | Intergenic | -21246 | RAB10 |
| chr10-144 | chr10 | 50819840 | 50819990 | 40.2 | exon (NM_003055, exon 1 of 1) | -1239 | CHAT |
| chr7-85 | chr7 | 3961993 | 3962143 | 40.2 | intron (NM_152744, intron 5 of 44) | -207249 | SDK1 |
| chr12-117 | chr12 | 89968888 | 89969038 | 40.2 | Intergenic | -48924 | POC1B |
| chr1-115 | chr1 | 77788060 | 77788210 | 40.2 | intron (NM_174858, intron 5 of 13) | 39848 | AK5 |
| chr19-114 | chr19 | 39184330 | 39184480 | 40.2 | intron (NM_004924, intron 1 of 20) | 46138 | ACTN4 |
| chr18-43 | chr18 | 11947386 | 11947536 | 40.2 | Intergenic | -33966 | IMPA2 |
| chr11-98 | chr11 | 20385554 | 20385704 | 40.2 | promoter-TSS (NM_001098523) | -58 | HTATIP2 |
| chr5-67 | chr5 | 143578121 | 143578271 | 40.2 | intron (NM_020768, intron 2 of 3) | 27759 | KCTD16 |
| chr8-94 | chr8 | 126283715 | 126283865 | 40.2 | intron (NM_173685, intron 5 of 7) | -158773 | TRIB1 |
| chr17-98 | chr17 | 57930642 | 57930792 | 40.2 | Intergenic | 12090 | MIR21 |
| chr10-119 | chr10 | 33527034 | 33527184 | 40.2 | intron (NM_001244972, intron 6 of 16) | 96724 | NRP1 |
| chr15-74 | chr15 | 35585891 | 35586041 | 40.2 | Intergenic | 56439 | ANP32AP1 |
| chr6-90 | chr6 | 125305909 | 125306059 | 40.2 | intron (NM_152553, intron 1 of 8) | 1470 | RNF217 |
| chr2-149 | chr2 | 11237544 | 11237694 | 40.2 | Intergenic | 34683 | FLJ33534 |
| chr9-86 | chr9 | 35492253 | 35492403 | 40.2 | intron (NR_052015, intron 1 of 11) | 2321 | RUSC2 |
| chr17-106 | chr17 | 19250301 | 19250451 | 40.2 | intron (NM_001243473, intron 5 of 5) | -2489 | MIR1180 |
| chr5-95 | chr5 | 172209793 | 172209943 | 40.2 | Intergenic | -11665 | DUSP1 |
| chr1-135 | chr1 | 233248881 | 233249031 | 40.2 | intron (NM_014801, intron 21 of 33) | 162586 | NTPCR |
| chr6-68 | chr6 | 3002161 | 3002311 | 40.2 | intron (NM_000904, intron 1 of 6) | 2169 | NQO2 |
| chr19-75 | chr19 | 14268746 | 14268896 | 40.2 | exon (NM_014921, exon 13 of 23) | 20857 | LOC100507373 |
| chr15-63 | chr15 | 59191707 | 59191857 | 40.2 | exon (NM_001013843, exon 7 of 21) | 34070 | SLTM |
| chr11-97 | chr11 | 18350812 | 18350962 | 40.2 | intron (NM_001142307, intron 2 of 15) | 7071 | GTF2H1 |
| chr14-54 | chr14 | 23067892 | 23068042 | 40.2 | intron (NM_022060, intron 1 of 6) | 820 | ABHD4 |
| chr1-118 | chr1 | 93416912 | 93417062 | 40.2 | intron (NM_001252270, intron 1 of 3) | 10092 | FAM69A |
| chr1-165 | chr1 | 165620331 | 165620481 | 40.2 | intron (NM_004528, intron 3 of 5) | 20296 | MGST3 |
| chr9-96 | chr9 | 112942123 | 112942273 | 40.2 | Intergenic | 28215 | C9orf152 |
| chr10-150 | chr10 | 95196762 | 95196912 | 40.2 | intron (NM_133337, intron 3 of 52) | 45237 | MYOF |
| chr2-187 | chr2 | 235889988 | 235890138 | 40.2 | intron (NM_014521, intron 1 of 5) | 29435 | SH3BP4 |
| chr1-105 | chr1 | 8257874 | 8258024 | 40.2 | Intergenic | -126441 | SLC45A1 |
| chr11-89 | chr11 | 91129529 | 91129679 | 40.2 | Intergenic | -840579 | MIR4490 |
| chr11-85 | chr11 | 72518393 | 72518543 | 40.2 | Intergenic | -6983 | ATG16L2 |
| chr20-55 | chr20 | 44572936 | 44573086 | 40.2 | intron (NM_022104, intron 10 of 16) | 9694 | PCIF1 |
| chrX-43 | chrX | 107279769 | 107279919 | 40.2 | Intergenic | -8356 | VSIG1 |
| chr17-124 | chr17 | 66202237 | 66202387 | 40.2 | Intergenic | 7511 | LOC440461 |
| chr4-69 | chr4 | 112433371 | 112433521 | 40.2 | Intergenic | -633107 | C4orf32 |
| chr20-46 | chr20 | 60508194 | 60508344 | 40.2 | intron (NM_001794, intron 14 of 15) | 20449 | MIR1257 |
| chr16-69 | chr16 | 9171253 | 9171403 | 40.2 | Intergenic | -14209 | C16orf72 |
| chr8-93 | chr8 | 122755221 | 122755371 | 40.2 | Intergenic | -101666 | HAS2 |
| chr3-81 | chr3 | 12857991 | 12858141 | 40.2 | exon (NM_001162499, exon 10 of 15) | 19895 | CAND2 |
| chr7-112 | chr7 | 151384750 | 151384900 | 40.2 | intron (NM_016203, intron 3 of 15) | -55481 | PRKAG2 |
| chr5-89 | chr5 | 127009243 | 127009393 | 40.2 | Intergenic | 20611 | CTXN3 |
| chr10-156 | chr10 | 127836785 | 127836935 | 40.2 | intron (NM_021641, intron 4 of 18) | 240267 | ADAM12 |
| chr5-58 | chr5 | 29795495 | 29795645 | 40.2 | Intergenic | 868593 | LSP1P3 |
| chr10-148 | chr10 | 77524451 | 77524601 | 40.2 | Intergenic | -17993 | C10orf11 |
| chr20-50 | chr20 | 14194775 | 14194925 | 40.2 | intron (NM_080676, intron 3 of 16) | 123463 | FLRT3 |
| chr1-141 | chr1 | 9360279 | 9360429 | 40.2 | intron (NM_025106, intron 1 of 2) | 7413 | SPSB1 |
| chr7-64 | chr7 | 76009470 | 76009620 | 40.2 | Intergenic | -17296 | ZP3 |
| chr9-64 | chr9 | 8553565 | 8553715 | 40.2 | intron (NM_130391, intron 3 of 30) | 180306 | PTPRD |
| chr20-61 | chr20 | 58225607 | 58225757 | 40.2 | intron (NM_080672, intron 1 of 12) | -22338 | LOC100506384 |
| chr2-161 | chr2 | 72356419 | 72356569 | 40.2 | 3' UTR (NM_019885, exon 6 of 6) | 18469 | CYP26B1 |
| chr16-73 | chr16 | 19130319 | 19130469 | 40.2 | non-coding (NR_028028, exon 2 of 2) | 5140 | ITPRIPL2 |
| chr7-88 | chr7 | 31448027 | 31448177 | 40.2 | Intergenic | -67564 | NEUROD6 |
| chr12-125 | chr12 | 124773690 | 124773840 | 40.2 | promoter-TSS (NM_181709) | 55 | FAM101A |
| chr9-74 | chr9 | 123391438 | 123391588 | 40.2 | intron (NM_001080497, intron 2 of 5) | -49076 | CDK5RAP2 |
| chr12-93 | chr12 | 90150652 | 90150802 | 40.2 | Intergenic | 47995 | LOC338758 |
| chr1-161 | chr1 | 154452317 | 154452467 | 40.2 | 3' UTR (NM_001010846, exon 6 of 6) | 22134 | SHE |
| chr8-98 | chr8 | 133023073 | 133023223 | 40.2 | 3' UTR (NM_015137, exon 23 of 23) | 48479 | OC90 |
| chr7-106 | chr7 | 130870297 | 130870447 | 40.2 | intron (NM_001145354, intron 2 of 18) | 75517 | MKLN1 |
| chrX-30 | chrX | 16844421 | 16844571 | 40.2 | intron (NM_018360, intron 3 of 9) | 39941 | TXLNG |
| chr4-53 | chr4 | 6857801 | 6857951 | 40.2 | intron (NM_014743, intron 4 of 9) | -53295 | TBC1D14 |
| chr13-25 | chr13 | 30496658 | 30496808 | 40.2 | Intergenic | -13935 | LINC00544 |
| chr12-111 | chr12 | 48170595 | 48170745 | 40.2 | intron (NM_017842, intron 1 of 2) | 3703 | SLC48A1 |
| chr1-179 | chr1 | 235606168 | 235606318 | 40.2 | intron (NM_001079515, intron 15 of 16) | 61538 | B3GALNT2 |
| chr3-63 | chr3 | 30343649 | 30343799 | 40.2 | Intergenic | -304270 | TGFBR2 |
| chr12-104 | chr12 | 29534158 | 29534308 | 40.2 | promoter-TSS (NM_016570) | -90 | ERGIC2 |
| chr22-20 | chr22 | 21062189 | 21062339 | 40.2 | 3' UTR (NM_058004, exon 55 of 55) | 6862 | TMEM191A |
| chr14-67 | chr14 | 93012697 | 93012847 | 40.2 | intron (NM_024832, intron 1 of 9) | 32647 | RIN3 |
| chr3-91 | chr3 | 52640743 | 52640893 | 40.2 | intron (NM_018313, intron 17 of 29) | 70197 | SMIM4 |
| chr2-120 | chr2 | 228368321 | 228368471 | 40.2 | intron (NM_004504, intron 2 of 12) | 31508 | AGFG1 |
| chr19-109 | chr19 | 16189809 | 16189959 | 40.2 | intron (NM_003290, intron 1 of 7) | 2749 | TPM4 |
| chr14-60 | chr14 | 56666004 | 56666154 | 40.2 | intron (NM_021255, intron 2 of 5) | 80986 | PELI2 |
| chr9-63 | chr9 | 7320246 | 7320396 | 40.2 | Intergenic | 479478 | C9orf123 |
| chr18-39 | chr18 | 3596720 | 3596870 | 40.2 | non-coding (NR_024101, exon 3 of 3) | 2683 | DLGAP1-AS1 |
| chr1-166 | chr1 | 167793764 | 167793914 | 40.2 | intron (NM_018417, intron 27 of 32) | 89614 | ADCY10 |
| chr8-85 | chr8 | 48873426 | 48873576 | 40.2 | promoter-TSS (NM_001081640) | 738 | MCM4 |
| chr2-158 | chr2 | 55844951 | 55845101 | 40.2 | promoter-TSS (NM_001122964) | -230 | SMEK2 |
| chr4-51 | chr4 | 106407 | 106557 | 40.2 | intron (NM_001039127, intron 3 of 3) | 53205 | ZNF718 |
| chr5-63 | chr5 | 100426990 | 100427140 | 40.2 | Intergenic | -188078 | ST8SIA4 |
| chr5-86 | chr5 | 95177689 | 95177839 | 40.2 | Intergenic | -10172 | C5orf27 |
| chr2-146 | chr2 | 235588827 | 235588977 | 40.2 | Intergenic | -183209 | ARL4C |
| chr1-173 | chr1 | 207247712 | 207247862 | 40.2 | 3' UTR (NM_006212, exon 15 of 15) | -14425 | C4BPB |
| chr14-69 | chr14 | 104094574 | 104094724 | 40.2 | promoter-TSS (NM_182923) | -876 | KLC1 |
| chr14-41 | chr14 | 38060645 | 38060795 | 40.2 | exon (NM_004496, exon 2 of 2) | 3605 | FOXA1 |
| chr19-100 | chr19 | 50176810 | 50176960 | 40.2 | intron (NM_138639, intron 6 of 6) | -3524 | PRMT1 |
| chr15-80 | chr15 | 55095737 | 55095887 | 40.2 | Intergenic | 393419 | RSL24D1 |
| chr11-71 | chr11 | 1892391 | 1892541 | 40.2 | 5' UTR (NM_001013254, exon 1 of 11) | 312 | LSP1 |
| chr5-78 | chr5 | 57389945 | 57390095 | 40.2 | Intergenic | 365946 | PLK2 |
| chr18-47 | chr18 | 57584878 | 57585028 | 40.2 | Intergenic | 17761 | PMAIP1 |
| chr4-74 | chr4 | 188967967 | 188968117 | 40.2 | Intergenic | 51117 | ZFP42 |
| chr16-87 | chr16 | 81018195 | 81018345 | 40.2 | intron (NM_020188, intron 2 of 3) | -21833 | CENPN |
| chr2-180 | chr2 | 161418590 | 161418740 | 40.2 | Intergenic | -68347 | RBMS1 |
| chr12-105 | chr12 | 31885958 | 31886108 | 40.2 | Intergenic | -3925 | AMN1 |
| chr6-70 | chr6 | 12345307 | 12345457 | 40.2 | Intergenic | 47204 | RNU6-48 |
| chr6-91 | chr6 | 132275344 | 132275494 | 40.2 | Intergenic | -2901 | CTGF |
| chr4-44 | chr4 | 90169595 | 90169745 | 40.2 | exon (NM_198281, exon 2 of 2) | 59491 | GPRIN3 |
| chr22-24 | chr22 | 46116991 | 46117141 | 40.2 | intron (NM_013236, intron 6 of 11) | -39338 | MIR4762 |
| chr11-96 | chr11 | 13356040 | 13356190 | 40.2 | intron (NM_001178, intron 3 of 19) | 56790 | ARNTL |
| chr17-85 | chr17 | 15546366 | 15546516 | 40.2 | intron (NM_006470, intron 4 of 8) | -23423 | CDRT1 |
| chr15-92 | chr15 | 85498574 | 85498724 | 40.2 | Intergenic | -25095 | PDE8A |
| chr16-85 | chr16 | 70995849 | 70995999 | 40.2 | exon (NM_001270974, exon 38 of 86) | -160863 | VAC14 |
| chr3-85 | chr3 | 36155937 | 36156087 | 40.2 | Intergenic | -266085 | STAC |
| chr8-97 | chr8 | 130722834 | 130722984 | 40.2 | Intergenic | 76225 | GSDMC |
| chr17-112 | chr17 | 33412030 | 33412180 | 40.2 | intron (NR_037713, intron 1 of 6) | 4243 | RFFL |
| chr19-104 | chr19 | 5831472 | 5831622 | 40.2 | exon (NM_000150, exon 3 of 3) | 7729 | NRTN |
| chr7-108 | chr7 | 132468837 | 132468987 | 40.2 | TTS (NM_017812) | 135359 | FLJ40288 |
| chr17-99 | chr17 | 61352765 | 61352915 | 40.2 | intron (NM_025185, intron 7 of 24) | 165367 | CYB561 |
| chr12-116 | chr12 | 88693494 | 88693644 | 40.2 | Intergenic | 157496 | TMTC3 |
| chr19-115 | chr19 | 39936090 | 39936240 | 40.2 | promoter-TSS (NM_001130824) | -21 | SUPT5H |
| chr17-126 | chr17 | 67316459 | 67316609 | 40.2 | intron (NM_172232, intron 1 of 38) | -4760 | ABCA5 |
| chr1-180 | chr1 | 242389134 | 242389284 | 40.2 | intron (NM_152666, intron 5 of 10) | 223575 | PLD5 |
| chr2-137 | chr2 | 110863779 | 110863929 | 40.2 | intron (NM_005434, intron 1 of 3) | 10289 | MALL |
| chr20-44 | chr20 | 34330151 | 34330301 | 40.2 | promoter-TSS (NR_040724) | 32 | RBM39 |
| chr6-77 | chr6 | 32948220 | 32948370 | 40.2 | intron (NR_037625, intron 13 of 13) | 7434 | BRD2 |
| chr7-110 | chr7 | 149191920 | 149192070 | 40.2 | intron (NM_152557, intron 1 of 6) | 2903 | ZNF746 |
| chr4-34 | chr4 | 1641597 | 1641747 | 40.2 | TTS (NM_001174070) | 44046 | FAM53A |
| chr2-184 | chr2 | 219140065 | 219140215 | 40.2 | 3' UTR (NM_022152, exon 12 of 12) | 5025 | PNKD |
| chr7-111 | chr7 | 150938142 | 150938292 | 40.2 | intron (NM_001003802, intron 9 of 13) | 2710 | MIR671 |
| chr10-149 | chr10 | 86070317 | 86070467 | 40.2 | Intergenic | -18018 | FAM190B |
| chr6-65 | chr6 | 125523387 | 125523537 | 40.2 | intron (NM_001003395, intron 1 of 6) | 48042 | TPD52L1 |
| chr14-65 | chr14 | 89337885 | 89338035 | 40.2 | exon (NM_198310, exon 10 of 13) | 46982 | TTC8 |
| chr10-136 | chr10 | 31531897 | 31532047 | 40.2 | Intergenic | 76052 | ZEB1-AS1 |
| chr6-54 | chr6 | 30796461 | 30796611 | 40.2 | Intergenic | -55325 | DDR1 |
| chr2-172 | chr2 | 121697311 | 121697461 | 40.2 | intron (NM_005270, intron 2 of 12) | 142519 | GLI2 |
| chr2-175 | chr2 | 128266219 | 128266369 | 40.2 | intron (NM_017969, intron 2 of 13) | 17793 | IWS1 |
| chr5-76 | chr5 | 10504383 | 10504533 | 40.2 | Intergenic | -59977 | ANKRD33B |
| chr7-67 | chr7 | 134278717 | 134278867 | 40.2 | Intergenic | 44943 | AKR1B15 |
| chr13-29 | chr13 | 67231413 | 67231563 | 40.2 | intron (NM_020403, intron 2 of 3) | -167813 | PCDH9-AS2 |
| chr5-79 | chr5 | 59082410 | 59082560 | 40.2 | intron (NM_001165899, intron 3 of 16) | -18047 | PDE4D |
| chr15-62 | chr15 | 52876874 | 52877024 | 40.2 | exon (NM_019600, exon 12 of 13) | -15736 | ARPP19 |
| chr3-89 | chr3 | 49131481 | 49131631 | 40.2 | promoter-TSS (NM_017730) | -52 | QRICH1 |
| chr14-61 | chr14 | 56806806 | 56806956 | 40.2 | Intergenic | 221788 | PELI2 |
| chr11-113 | chr11 | 85376282 | 85376432 | 40.2 | promoter-TSS (NM_001039618) | -175 | CREBZF |
| chr14-55 | chr14 | 31911039 | 31911189 | 40.2 | Intergenic | 15566 | DTD2 |
| chr10-155 | chr10 | 121417904 | 121418054 | 40.2 | intron (NM_004281, intron 1 of 3) | 7097 | BAG3 |
| chr9-97 | chr9 | 114821798 | 114821948 | 40.2 | intron (NM_022486, intron 13 of 16) | 115683 | SUSD1 |
| chr5-91 | chr5 | 137336000 | 137336150 | 40.2 | intron (NM_001101800, intron 7 of 21) | 32727 | FAM13B |
| chr17-127 | chr17 | 67470283 | 67470433 | 40.2 | intron (NM_002758, intron 1 of 11) | 59520 | MAP2K6 |
| chr1-153 | chr1 | 90440624 | 90440774 | 40.2 | Intergenic | 19826 | GEMIN8P4 |
| chr20-58 | chr20 | 48782751 | 48782901 | 40.2 | Intergenic | -12491 | TMEM189-UBE2V1 |
| chr4-54 | chr4 | 14728834 | 14728984 | 40.2 | Intergenic | 274760 | LOC441009 |
| chr3-80 | chr3 | 12235813 | 12235963 | 40.2 | Intergenic | -35037 | TIMP4 |
| chr6-89 | chr6 | 121730782 | 121730932 | 40.2 | Intergenic | -25888 | GJA1 |
| chr9-82 | chr9 | 26940094 | 26940244 | 40.2 | intron (NM_001031689, intron 1 of 13) | -6868 | IFT74 |
| chr12-109 | chr12 | 47470289 | 47470439 | 40.2 | TTS (NM_001143668) | 3370 | AMIGO2 |
| chr14-39 | chr14 | 24502353 | 24502503 | 40.2 | Intergenic | -3282 | DHRS4L1 |
| chr19-111 | chr19 | 33023271 | 33023421 | 40.2 | Intergenic | -48748 | PDCD5 |
| chr13-18 | chr13 | 67508188 | 67508338 | 40.2 | intron (NM_203487, intron 2 of 4) | -43258 | PCDH9-AS3 |
| chr2-157 | chr2 | 55265783 | 55265933 | 40.2 | intron (NM_207520, intron 1 of 7) | 10468 | RTN4 |
| chr16-72 | chr16 | 17922019 | 17922169 | 40.2 | Intergenic | -357356 | XYLT1 |
| chr12-110 | chr12 | 47479650 | 47479800 | 40.2 | Intergenic | -5991 | AMIGO2 |
| chr7-103 | chr7 | 115872073 | 115872223 | 40.2 | intron (NM_152829, intron 1 of 6) | 9143 | TES |
| chr7-82 | chr7 | 139614110 | 139614260 | 40.2 | intron (NM_001061, intron 4 of 12) | 85233 | TBXAS1 |
| chr22-28 | chr22 | 43391312 | 43391462 | 40.2 | intron (NM_001184970, intron 1 of 10) | 19797 | PACSIN2 |
| chr9-89 | chr9 | 71397493 | 71397643 | 40.2 | 3' UTR (NM_138333, exon 1 of 1) | 2604 | FAM122A |
| chr1-178 | chr1 | 227619634 | 227619784 | 40.2 | Intergenic | -113883 | CDC42BPA |
| chr8-92 | chr8 | 105598107 | 105598257 | 40.2 | intron (NM_013437, intron 1 of 6) | 3070 | LRP12 |
| chr6-74 | chr6 | 17876275 | 17876425 | 40.2 | intron (NM_001105568, intron 3 of 37) | 111504 | KIF13A |
| chr19-118 | chr19 | 45250739 | 45250889 | 40.2 | Intergenic | -1164 | BCL3 |
| chr5-69 | chr5 | 149185497 | 149185647 | 40.2 | intron (NM_001172699, intron 1 of 10) | 34069 | PPARGC1B |
| chr19-116 | chr19 | 41892353 | 41892503 | 40.2 | 3' UTR (NM_020158, exon 6 of 6) | 9259 | TMEM91 |
| chr4-64 | chr4 | 75240878 | 75241028 | 40.2 | intron (NM_001432, intron 1 of 4) | 10093 | EREG |
| chr19-108 | chr19 | 15636107 | 15636257 | 40.2 | exon (NM_173483, exon 3 of 14) | 16846 | CYP4F22 |
| chr17-104 | chr17 | 17124810 | 17124960 | 40.2 | exon (NM_144606, exon 8 of 8) | -15239 | PLD6 |
| chr5-60 | chr5 | 42950813 | 42950963 | 40.2 | Intergenic | 68025 | LOC648987 |
| chr14-59 | chr14 | 56060419 | 56060569 | 40.2 | intron (NM_001079521, intron 1 of 43) | 13569 | KTN1 |
| chr1-137 | chr1 | 248789806 | 248789956 | 40.2 | exon (NM_001001964, exon 1 of 1) | 548 | OR2T11 |
| chr10-117 | chr10 | 32229860 | 32230010 | 40.2 | Intergenic | -12131 | ARHGAP12 |
| chr5-84 | chr5 | 88039367 | 88039517 | 40.2 | intron (NM_002397, intron 6 of 10) | -58822 | LINC00461 |
| chr9-80 | chr9 | 6196388 | 6196538 | 40.2 | Intergenic | -19323 | IL33 |
| chr13-19 | chr13 | 67695151 | 67695301 | 40.2 | intron (NM_203487, intron 2 of 4) | 109242 | PCDH9 |
| chr7-87 | chr7 | 24772890 | 24773040 | 40.2 | intron (NM_001127454, intron 2 of 8) | 24118 | DFNA5 |
| chr10-152 | chr10 | 101295879 | 101296029 | 40.2 | 3' UTR (NM_145285, exon 2 of 2) | 3264 | NKX2-3 |
| chr2-188 | chr2 | 237621046 | 237621196 | 40.2 | Intergenic | 142741 | CXCR7 |
| chr6-80 | chr6 | 43225684 | 43225834 | 40.2 | intron (NM_032538, intron 10 of 14) | 14537 | TTBK1 |
| chr1-146 | chr1 | 46420295 | 46420445 | 40.2 | intron (NM_015112, intron 4 of 28) | 151085 | MAST2 |
| chr8-86 | chr8 | 59666195 | 59666345 | 40.2 | Intergenic | -93866 | NSMAF |
| chr3-66 | chr3 | 56835753 | 56835903 | 40.2 | 5' UTR (NM_019555, exon 1 of 10) | 167 | ARHGEF3 |
| chr6-88 | chr6 | 118721882 | 118722032 | 40.2 | Intergenic | -100579 | BRD7P3 |
| chr9-92 | chr9 | 91389972 | 91390122 | 40.2 | Intergenic | -29227 | MIR4289 |
| chr14-62 | chr14 | 68699298 | 68699448 | 40.2 | intron (NM_133510, intron 7 of 10) | 412877 | RAD51B |
| chr17-121 | chr17 | 57784786 | 57784936 | 40.2 | promoter-TSS (NM_016077) | -2 | VMP1 |
| chr9-99 | chr9 | 136218703 | 136218853 | 40.2 | TTS (NM_000972) | 1077 | SNORD36C |
| chr1-172 | chr1 | 199777246 | 199777396 | 40.2 | Intergenic | -219449 | NR5A2 |
| chrX-26 | chrX | 13795345 | 13795495 | 40.2 | intron (NM_001001996, intron 6 of 7) | 39894 | GPM6B |
| chr7-109 | chr7 | 134844971 | 134845121 | 40.2 | intron (NM_018295, intron 1 of 1) | 9180 | C7orf49 |
| chr6-78 | chr6 | 34625301 | 34625451 | 40.2 | intron (NM_024294, intron 1 of 4) | 39249 | C6orf106 |
| chr2-181 | chr2 | 170623558 | 170623708 | 40.2 | Intergenic | -31689 | SSB |
| chr5-87 | chr5 | 106620985 | 106621135 | 40.2 | Intergenic | 385536 | EFNA5 |
| chr5-70 | chr5 | 153747672 | 153747822 | 40.2 | intron (NM_198321, intron 4 of 11) | 77635 | FLJ38109 |
| chr3-65 | chr3 | 33132336 | 33132486 | 40.2 | 3' UTR (NM_001136238, exon 2 of 2) | 5882 | TMPPE |
| chr6-83 | chr6 | 57039079 | 57039229 | 40.2 | intron (NM_004282, intron 1 of 2) | 2050 | BAG2 |
| chr15-83 | chr15 | 67291638 | 67291788 | 40.2 | Intergenic | -66482 | SMAD3 |
| chr2-163 | chr2 | 89035549 | 89035699 | 40.2 | intron (NM_144563, intron 6 of 8) | -29795 | ANKRD36BP2 |
| chr1-155 | chr1 | 110881549 | 110881699 | 40.2 | promoter-TSS (NM_001201545) | 169 | LOC440600 |
| chr8-79 | chr8 | 19484494 | 19484644 | 40.2 | intron (NR_024040, intron 1 of 9) | -24513 | CSGALNACT1 |
| chr8-96 | chr8 | 129295389 | 129295539 | 40.2 | Intergenic | 133102 | MIR1208 |
| chr3-109 | chr3 | 196844124 | 196844274 | 40.2 | intron (NM_001204386, intron 12 of 24) | 66803 | DLG1 |
| chr3-84 | chr3 | 29725283 | 29725433 | 40.2 | intron (NM_001003793, intron 4 of 14) | 402555 | RBMS3 |
| chr2-173 | chr2 | 122288724 | 122288874 | 40.2 | TTS (NR_023343) | 343 | RNU4ATAC |
| chr17-110 | chr17 | 31149672 | 31149822 | 40.2 | intron (NM_015194, intron 1 of 21) | 54155 | MYO1D |
| chr14-53 | chr14 | 103800244 | 103800394 | 40.2 | promoter-TSS (NM_001969) | -20 | EIF5 |
| chr9-76 | chr9 | 125589546 | 125589696 | 40.2 | intron (NM_005388, intron 1 of 3) | 1314 | PDCL |
| chr1-168 | chr1 | 171506350 | 171506500 | 40.2 | exon (NM_015172, exon 15 of 34) | 51759 | PRRC2C |
| chr1-163 | chr1 | 156631237 | 156631387 | 40.2 | Intergenic | 15877 | NES |
| chr14-56 | chr14 | 39530843 | 39530993 | 40.2 | intron (NM_006364, intron 13 of 19) | 41519 | SEC23A |
| chr12-91 | chr12 | 80014781 | 80014931 | 40.2 | Intergenic | 69934 | PAWR |
| chr8-95 | chr8 | 128981775 | 128981925 | 40.2 | intron (NR_003367, intron 2 of 7) | 8971 | MIR1205 |
| chr2-143 | chr2 | 206599235 | 206599385 | 40.2 | intron (NM_201264, intron 7 of 8) | 52086 | NRP2 |
| chr4-55 | chr4 | 16258247 | 16258397 | 40.2 | non-coding (NR_027697, exon 3 of 3) | 30036 | FLJ39653 |
| chr11-108 | chr11 | 66300146 | 66300296 | 40.2 | 3' UTR (NM_024649, exon 17 of 17) | 13450 | ZDHHC24 |
| chr19-119 | chr19 | 46519561 | 46519711 | 40.2 | exon (NM_001267723, exon 9 of 14) | -2554 | MIR769 |
| chr9-84 | chr9 | 34163564 | 34163714 | 40.2 | Intergenic | -15364 | UBAP1 |
| chr20-48 | chr20 | 5046662 | 5046812 | 40.2 | Intergenic | 46996 | TMEM230 |
| chr8-83 | chr8 | 38856936 | 38857086 | 40.2 | intron (NR_027638, intron 1 of 20) | 2506 | ADAM9 |
| chr15-72 | chr15 | 30107679 | 30107829 | 40.2 | intron (NM_175610, intron 1 of 26) | 6952 | TJP1 |
| chr15-93 | chr15 | 85901257 | 85901407 | 40.2 | Intergenic | -22515 | AKAP13 |
| chr6-73 | chr6 | 16687766 | 16687916 | 40.2 | intron (NM_000332, intron 3 of 8) | 73880 | ATXN1 |
| chrX-39 | chrX | 53835178 | 53835328 | 40.2 | Intergenic | -121556 | HUWE1 |
| chr2-185 | chr2 | 223907570 | 223907720 | 40.2 | Intergenic | -9217 | KCNE4 |
| chr19-124 | chr19 | 53857405 | 53857555 | 40.2 | 3' UTR (NM_138374, exon 4 of 4) | -11488 | ZNF525 |
| chr6-92 | chr6 | 133318644 | 133318794 | 40.2 | Intergenic | -90500 | LINC00326 |
| chr7-104 | chr7 | 128484830 | 128484980 | 40.2 | exon (NM_001458, exon 21 of 48) | 14422 | FLNC |
| chr16-82 | chr16 | 48337179 | 48337329 | 40.2 | intron (NM_031490, intron 11 of 14) | -52342 | LOC100507577 |
| chr1-98 | chr1 | 189576442 | 189576592 | 40.2 | Intergenic | 870242 | FAM5C |
| chrX-33 | chrX | 30846888 | 30847038 | 40.2 | 3' UTR (NM_152787, exon 11 of 11) | 60548 | TAB3 |
| chr18-48 | chr18 | 57721019 | 57721169 | 40.2 | Intergenic | 153902 | PMAIP1 |
| chr19-110 | chr19 | 31981377 | 31981527 | 40.2 | Intergenic | -101735 | THEG5 |
| chr5-83 | chr5 | 82937380 | 82937530 | 40.2 | exon (NM_001884, exon 5 of 5) | 79441 | HAPLN1 |
| chr20-59 | chr20 | 55167321 | 55167471 | 40.2 | Intergenic | -36962 | TFAP2C |
| chr1-176 | chr1 | 212662079 | 212662229 | 40.2 | Intergenic | 55925 | NENF |
| chr19-96 | chr19 | 34717118 | 34717268 | 40.2 | intron (NM_015578, intron 9 of 9) | -28263 | KIAA0355 |
| chr5-80 | chr5 | 65278559 | 65278709 | 40.2 | intron (NM_001253701, intron 1 of 24) | -37233 | LOC100303749 |
| chr9-94 | chr9 | 97708161 | 97708311 | 40.2 | intron (NM_001193329, intron 6 of 15) | 135992 | MIR2278 |
| chr5-65 | chr5 | 118660082 | 118660232 | 40.2 | intron (NM_001077654, intron 1 of 1) | -31439 | TNFAIP8 |
| chr13-28 | chr13 | 52733900 | 52734050 | 40.2 | promoter-TSS (NM_001146099) | 21 | NEK3 |
| chr10-146 | chr10 | 71149998 | 71150148 | 40.2 | intron (NM_000188, intron 14 of 17) | 26601 | TACR2 |
| chr19-112 | chr19 | 33771562 | 33771712 | 40.2 | Intergenic | 21793 | CEBPA |
| chr2-151 | chr2 | 23930848 | 23930998 | 40.2 | 3' UTR (NM_052920, exon 14 of 14) | 219013 | ATAD2B |
| chr7-101 | chr7 | 100681535 | 100681685 | 40.2 | exon (NM_001040105, exon 3 of 13) | 18246 | MUC17 |
| chr14-66 | chr14 | 91496275 | 91496425 | 40.2 | intron (NM_004755, intron 1 of 16) | 30643 | RPS6KA5 |
| chrX-41 | chrX | 64888581 | 64888731 | 40.2 | intron (NM_002444, intron 1 of 12) | 1145 | MSN |
| chr12-114 | chr12 | 79758142 | 79758292 | 40.2 | intron (NM_001135805, intron 10 of 11) | -54820 | MIR1252 |
| chr5-85 | chr5 | 95109546 | 95109696 | 40.2 | intron (NM_014899, intron 8 of 11) | 42771 | RHOBTB3 |
| chr10-116 | chr10 | 30101167 | 30101317 | 40.2 | Intergenic | -76512 | SVIL |
| chr18-42 | chr18 | 9825672 | 9825822 | 40.2 | intron (NM_006868, intron 5 of 6) | -59976 | TXNDC2 |
| chr6-87 | chr6 | 116575008 | 116575158 | 40.2 | exon (NM_021648, exon 1 of 1) | 178 | TSPYL4 |
| chr11-106 | chr11 | 62160708 | 62160858 | 40.2 | 3' UTR (NM_025080, exon 7 of 7) | -25724 | SCGB1A1 |
| chr5-92 | chr5 | 143154989 | 143155139 | 40.2 | Intergenic | -36662 | HMHB1 |
| chr1-147 | chr1 | 52344740 | 52344890 | 40.2 | promoter-TSS (NM_001101662) | -206 | NRD1 |
| chr3-87 | chr3 | 48627862 | 48628012 | 40.2 | exon (NM_000094, exon 14 of 118) | 4656 | COL7A1 |
| chr7-69 | chr7 | 28338536 | 28338686 | 40.2 | promoter-TSS (NM_182899) | -329 | CREB5 |
| chr17-90 | chr17 | 33416926 | 33417076 | 40.2 | promoter-TSS (NR_037713) | -653 | RFFL |
| chr2-168 | chr2 | 101519428 | 101519578 | 40.2 | intron (NM_002518, intron 1 of 20) | 82890 | NPAS2 |
| chr1-110 | chr1 | 44249456 | 44249606 | 40.2 | intron (NM_001270465, intron 2 of 7) | 76327 | ST3GAL3 |
| chr16-67 | chr16 | 86341340 | 86341490 | 40.2 | Intergenic | 21378 | LOC146513 |
| chr2-150 | chr2 | 15870667 | 15870817 | 40.2 | Intergenic | 138997 | DDX1 |
| chr19-87 | chr19 | 54523597 | 54523747 | 40.2 | Intergenic | 28130 | CACNG6 |
| chr3-102 | chr3 | 142371824 | 142371974 | 40.2 | intron (NM_002670, intron 1 of 15) | -3840 | PLS1 |
| chr8-99 | chr8 | 142367451 | 142367601 | 40.2 | exon (NM_005293, exon 2 of 2) | 9839 | GPR20 |
| chr19-117 | chr19 | 42734029 | 42734179 | 40.2 | TTS (NM_019884) | 9612 | ZNF526 |
| chr16-76 | chr16 | 24850156 | 24850306 | 40.2 | Intergenic | -6953 | SLC5A11 |
| chr15-77 | chr15 | 44817499 | 44817649 | 40.2 | 3' UTR (NM_016396, exon 13 of 13) | 11524 | LOC645212 |
| chr22-26 | chr22 | 35779069 | 35779219 | 40.2 | exon (NM_002133, exon 2 of 5) | 2084 | HMOX1 |
| chr7-97 | chr7 | 80108131 | 80108281 | 40.2 | exon (NM_001102386, exon 4 of 8) | 33036 | GNAT3 |
| chr19-106 | chr19 | 12384435 | 12384585 | 40.2 | exon (NM_016264, exon 4 of 4) | 21204 | ZNF44 |
| chr15-89 | chr15 | 76612508 | 76612658 | 40.2 | Intergenic | -8773 | ETFA |
| chr2-148 | chr2 | 677406 | 677556 | 40.2 | promoter-TSS (NM_152834) | -42 | TMEM18 |
| chr3-64 | chr3 | 30789561 | 30789711 | 40.2 | intron (NM_207359, intron 14 of 14) | 141642 | TGFBR2 |
| chr5-61 | chr5 | 58358974 | 58359124 | 40.2 | intron (NM_001104631, intron 5 of 14) | -23710 | PDE4D |
| chr1-142 | chr1 | 16219814 | 16219964 | 40.2 | intron (NM_015001, intron 3 of 14) | -45247 | FLJ37453 |
| chr3-70 | chr3 | 131262726 | 131262876 | 40.2 | intron (NM_130808, intron 14 of 15) | -40941 | MRPL3 |
| chr16-51 | chr16 | 71715689 | 71715839 | 40.2 | exon (NM_015020, exon 5 of 18) | 16840 | SNORA70D |
| chr3-72 | chr3 | 148766534 | 148766684 | 40.2 | intron (NM_139048, intron 16 of 25) | -37510 | HLTF-AS1 |
| chr1-114 | chr1 | 71538144 | 71538294 | 40.2 | exon (NM_005455, exon 5 of 11) | -4820 | MIR186 |
| chr6-67 | chr6 | 158461411 | 158461561 | 40.2 | intron (NM_001178088, intron 3 of 25) | 23406 | SYNJ2 |
| chr3-108 | chr3 | 191480178 | 191480328 | 40.2 | Intergenic | 301301 | PYDC2 |
| chr1-140 | chr1 | 8319060 | 8319210 | 40.2 | Intergenic | -65255 | SLC45A1 |
| chr21-19 | chr21 | 46021192 | 46021342 | 40.2 | exon (NM_198689, exon 2 of 2) | 770 | KRTAP10-7 |
| chr20-40 | chr20 | 5061035 | 5061185 | 40.2 | Intergenic | 32623 | TMEM230 |
| chr20-53 | chr20 | 23603340 | 23603490 | 40.2 | Intergenic | 15159 | CST3 |
| chr21-21 | chr21 | 16816698 | 16816848 | 40.2 | Intergenic | -285723 | USP25 |
| chr17-120 | chr17 | 47658808 | 47658958 | 40.2 | 3' UTR (NM_007225, exon 2 of 2) | 5585 | NXPH3 |
| chr16-57 | chr16 | 1696054 | 1696204 | 40.2 | intron (NM_020825, intron 5 of 19) | 31488 | CRAMP1L |
| chr10-143 | chr10 | 48524506 | 48524656 | 40.2 | Intergenic | -85443 | GDF10 |
| chr7-92 | chr7 | 43732314 | 43732464 | 40.2 | intron (NR_047701, intron 1 of 7) | 36751 | COA1 |
| chr1-154 | chr1 | 110334896 | 110335046 | 40.2 | Intergenic | -28327 | EPS8L3 |
| chr5-66 | chr5 | 122545320 | 122545470 | 40.2 | Intergenic | 120554 | PRDM6 |
| chr4-42 | chr4 | 75607995 | 75608145 | 40.2 | Intergenic | 111812 | BTC |
| chr14-43 | chr14 | 62026758 | 62026908 | 40.2 | Intergenic | -10425 | FLJ22447 |
| chr17-115 | chr17 | 39368402 | 39368552 | 40.2 | Intergenic | -14423 | KRTAP9-2 |
| chr6-72 | chr6 | 16623367 | 16623517 | 40.2 | intron (NM_000332, intron 4 of 8) | 138279 | ATXN1 |
| chr16-84 | chr16 | 68025888 | 68026038 | 40.2 | intron (NM_022355, intron 4 of 10) | 7458 | DPEP2 |
| chr12-83 | chr12 | 31886744 | 31886894 | 40.2 | Intergenic | -4711 | AMN1 |
| chr9-77 | chr9 | 135145658 | 135145808 | 40.2 | intron (NM_015046, intron 24 of 25) | 84639 | SETX |
| chr11-94 | chr11 | 3971639 | 3971789 | 40.2 | intron (NM_003156, intron 1 of 11) | 94422 | MIR4687 |
| chrX-32 | chrX | 24164070 | 24164220 | 40.2 | TTS (NR_046657) | -3617 | ZFX |
| chrX-38 | chrX | 48897431 | 48897581 | 40.2 | intron (NM_006521, intron 2 of 9) | 3484 | TFE3 |
| chr2-162 | chr2 | 73456445 | 73456595 | 40.2 | intron (NM_032319, intron 3 of 4) | 3836 | PRADC1 |
| chr7-102 | chr7 | 105765940 | 105766090 | 40.2 | Intergenic | -12922 | SYPL1 |
| chrX-42 | chrX | 69478981 | 69479131 | 40.2 | exon (NM_002565, exon 1 of 1) | 598 | P2RY4 |
| chr8-82 | chr8 | 37823524 | 37823674 | 40.2 | exon (NM_000025, exon 1 of 2) | 585 | ADRB3 |
| chr7-84 | chr7 | 3801344 | 3801494 | 40.2 | intron (NM_152744, intron 4 of 44) | -367898 | SDK1 |
| chr11-100 | chr11 | 32728627 | 32728777 | 40.2 | intron (NM_001008391, intron 3 of 17) | 87485 | CCDC73 |
| chr4-46 | chr4 | 95563486 | 95563636 | 40.2 | intron (NM_001256426, intron 13 of 16) | -115567 | BMPR1B |
| chr2-139 | chr2 | 191708730 | 191708880 | 40.2 | Intergenic | -36742 | GLS |
| chr19-101 | chr19 | 50180143 | 50180293 | 40.2 | promoter-TSS (NM_001536) | -191 | PRMT1 |
| chr11-109 | chr11 | 66738372 | 66738522 | 40.2 | Intergenic | -4307 | C11orf86 |
| chr17-84 | chr17 | 14641596 | 14641746 | 40.2 | Intergenic | -292621 | CDRT7 |
| chr5-59 | chr5 | 31323178 | 31323328 | 40.2 | exon (NM_004932, exon 12 of 12) | 129491 | CDH6 |
| chr10-126 | chr10 | 115086019 | 115086169 | 40.2 | Intergenic | -224496 | HABP2 |
| chr6-75 | chr6 | 24822364 | 24822514 | 40.2 | intron (NM_014722, intron 20 of 22) | 46798 | GMNN |
| chr12-103 | chr12 | 26385033 | 26385183 | 40.2 | 3' UTR (NM_005086, exon 3 of 3) | 36602 | SSPN |
| chr17-111 | chr17 | 31440027 | 31440177 | 40.2 | intron (NM_001094, intron 1 of 9) | 121220 | SPACA3 |
| chr11-105 | chr11 | 55211912 | 55212062 | 40.2 | Intergenic | 76627 | OR4A15 |
| chr1-171 | chr1 | 183897329 | 183897479 | 40.2 | 3' UTR (NM_015149, exon 19 of 19) | 109459 | GLT25D2 |
| chr7-105 | chr7 | 128486266 | 128486416 | 40.2 | intron (NM_001127487, intron 22 of 46) | 15858 | FLNC |
| chr1-103 | chr1 | 3229765 | 3229915 | 40.2 | intron (NM_199454, intron 3 of 16) | -141307 | ARHGEF16 |
| chr11-86 | chr11 | 85010417 | 85010567 | 40.2 | intron (NM_001142699, intron 3 of 27) | 327822 | DLG2 |
| chr16-83 | chr16 | 56948725 | 56948875 | 40.2 | 3' UTR (NM_000339, exon 26 of 26) | -16948 | HERPUD1 |
| chr10-151 | chr10 | 99357688 | 99357838 | 40.2 | intron (NM_001134670, intron 1 of 2) | 2788 | MIR5692C2 |
| chr9-95 | chr9 | 103191651 | 103191801 | 40.2 | promoter-TSS (NM_001198806) | -70 | MSANTD3 |
| chr1-160 | chr1 | 152140449 | 152140599 | 40.2 | Intergenic | -8820 | RPTN |
| chr5-93 | chr5 | 162806641 | 162806791 | 40.2 | Intergenic | -57861 | CCNG1 |
| chr15-84 | chr15 | 69652093 | 69652243 | 40.2 | intron (NM_001104554, intron 2 of 8) | 45426 | PAQR5 |
| chr1-151 | chr1 | 89236072 | 89236222 | 40.2 | exon (NM_006256, exon 4 of 22) | 86225 | PKN2 |
| chr5-74 | chr5 | 171384627 | 171384777 | 40.2 | exon (NM_012300, exon 2 of 13) | 49175 | FBXW11 |
| chr5-62 | chr5 | 59788874 | 59789024 | 40.2 | intron (NR_024617, intron 1 of 3) | -5024 | PDE4D |
| chr11-115 | chr11 | 102435008 | 102435158 | 40.2 | Intergenic | -33605 | MMP7 |
| chr3-68 | chr3 | 105833081 | 105833231 | 40.2 | Intergenic | -245269 | CBLB |
| chr6-82 | chr6 | 48794693 | 48794843 | 40.2 | Intergenic | 636273 | MUT |
| chr15-94 | chr15 | 99526722 | 99526872 | 40.2 | intron (NM_001167902, intron 2 of 4) | 22088 | PGPEP1L |
| chr8-63 | chr8 | 23780257 | 23780407 | 40.2 | Intergenic | -68012 | STC1 |
| chr10-198 | chr10 | 120891025 | 120891175 | 33.5 | intron (NM_207009, intron 7 of 8) | 27471 | FAM45B |
| chr5-131 | chr5 | 142872474 | 142872624 | 33.5 | Intergenic | -57472 | NR3C1 |
| chr7-136 | chr7 | 75969996 | 75970146 | 33.5 | intron (NM_012479, intron 1 of 1) | 18271 | YWHAG |
| chr14-89 | chr14 | 93784375 | 93784525 | 33.5 | intron (NM_001002860, intron 1 of 10) | 14935 | BTBD7 |
| chr16-100 | chr16 | 27579481 | 27579631 | 33.5 | intron (NM_015202, intron 1 of 27) | 18088 | KIAA0556 |
| chr10-172 | chr10 | 29024120 | 29024270 | 33.5 | Intergenic | 57771 | BAMBI |
| chr17-128 | chr17 | 72987898 | 72988048 | 33.5 | intron (NM_014603, intron 1 of 4) | 4246 | CDR2L |
| chr12-135 | chr12 | 17031820 | 17031970 | 33.5 | Intergenic | -109786 | SKP1P2 |
| chr4-100 | chr4 | 85200898 | 85201048 | 33.5 | Intergenic | 218414 | NKX6-1 |
| chrX-48 | chrX | 18433604 | 18433754 | 33.5 | Intergenic | -10046 | CDKL5 |
| chr8-127 | chr8 | 117264219 | 117264369 | 33.5 | intron (NR_046215, intron 4 of 13) | 73003 | LINC00536 |
| chrX-47 | chrX | 13416361 | 13416511 | 33.5 | Intergenic | 63076 | LOC100133123 |
| chr5-120 | chr5 | 67700958 | 67701108 | 33.5 | Intergenic | 112637 | PIK3R1 |
| chr1-206 | chr1 | 111459599 | 111459749 | 33.5 | Intergenic | 43952 | CD53 |
| chr3-112 | chr3 | 25831349 | 25831499 | 33.5 | promoter-TSS (NM_001145391) | 106 | NGLY1 |
| chr9-129 | chr9 | 134917854 | 134918004 | 33.5 | intron (NM_004269, intron 2 of 7) | 37345 | MED27 |
| chr8-135 | chr8 | 132869135 | 132869285 | 33.5 | Intergenic | -47146 | EFR3A |
| chr12-149 | chr12 | 89556755 | 89556905 | 33.5 | Intergenic | -143361 | LOC728084 |
| chr4-99 | chr4 | 84482530 | 84482680 | 33.5 | intron (NM_001256421, intron 3 of 12) | 25135 | AGPAT9 |
| chr5-127 | chr5 | 140235485 | 140235635 | 33.5 | promoter-TSS (NM_031859) | -74 | PCDHA10 |
| chr9-126 | chr9 | 132524924 | 132525074 | 33.5 | Intergenic | -9655 | PTGES |
| chr1-228 | chr1 | 179556811 | 179556961 | 33.5 | Intergenic | -3862 | TDRD5 |
| chr12-158 | chr12 | 106520384 | 106520534 | 33.5 | intron (NM_014840, intron 1 of 6) | 13352 | NUAK1 |
| chr1-138 | chr1 | 1333519 | 1333669 | 33.5 | intron (NM_001039577, intron 3 of 5) | 1124 | CCNL2 |
| chr17-147 | chr17 | 25901003 | 25901153 | 33.5 | intron (NM_014238, intron 3 of 20) | -57096 | LGALS9 |
| chr12-159 | chr12 | 107742801 | 107742951 | 33.5 | intron (NM_001018072, intron 1 of 16) | 30679 | BTBD11 |
| chr10-132 | chr10 | 5051510 | 5051660 | 33.5 | intron (NM_001354, intron 1 of 10) | -5379 | AKR1C2 |
| chr5-115 | chr5 | 58858829 | 58858979 | 33.5 | intron (NM_001165899, intron 3 of 16) | 23420 | PDE4D |
| chr20-83 | chr20 | 52199539 | 52199689 | 33.5 | promoter-TSS (NM_006526) | 22 | ZNF217 |
| chr1-159 | chr1 | 147737088 | 147737238 | 33.5 | Intergenic | 69515 | MIR5087 |
| chr2-171 | chr2 | 120517186 | 120517336 | 33.5 | promoter-TSS (NM_002830) | 54 | PTPN4 |
| chr15-125 | chr15 | 91522826 | 91522976 | 33.5 | intron (NM_001267580, intron 6 of 12) | 13302 | LOC100507118 |
| chr7-127 | chr7 | 47384267 | 47384417 | 33.5 | intron (NM_022748, intron 20 of 30) | 237400 | TNS3 |
| chr11-123 | chr11 | 33655012 | 33655162 | 33.5 | intron (NM_012194, intron 15 of 19) | 67199 | C11orf91 |
| chr15-105 | chr15 | 62939716 | 62939866 | 33.5 | intron (NM_015059, intron 1 of 55) | 281 | TLN2 |
| chr7-120 | chr7 | 21909550 | 21909700 | 33.5 | intron (NM_003777, intron 74 of 82) | 75917 | CDCA7L |
| chr17-161 | chr17 | 45303975 | 45304125 | 33.5 | Intergenic | 17336 | MYL4 |
| chr14-68 | chr14 | 93725344 | 93725494 | 33.5 | intron (NM_001002860, intron 5 of 10) | -51960 | C14orf142 |
| chr2-194 | chr2 | 25032578 | 25032728 | 33.5 | intron (NM_024322, intron 3 of 7) | 16322 | CENPO |
| chr11-134 | chr11 | 59774010 | 59774160 | 33.5 | Intergenic | -33663 | PLAC1L |
| chr1-242 | chr1 | 244102675 | 244102825 | 33.5 | intron (NR_033883, intron 1 of 11) | 22046 | LOC339529 |
| chr2-256 | chr2 | 235112037 | 235112187 | 33.5 | Intergenic | 152766 | SPP2 |
| chr4-110 | chr4 | 151158445 | 151158595 | 33.5 | intron (NR_036614, intron 10 of 16) | 159094 | DCLK2 |
| chr4-75 | chr4 | 779116 | 779266 | 33.5 | 3' UTR (NM_006651, exon 4 of 4) | -3555 | LOC100129917 |
| chr9-78 | chr9 | 4155286 | 4155436 | 33.5 | intron (NM_001042413, intron 2 of 10) | -3178 | GLIS3 |
| chr3-146 | chr3 | 149257962 | 149258112 | 33.5 | intron (NM_001168280, intron 4 of 6) | 65669 | TM4SF4 |
| chr19-143 | chr19 | 34116292 | 34116442 | 33.5 | intron (NM_001127896, intron 1 of 3) | 3494 | CHST8 |
| chr17-150 | chr17 | 28732357 | 28732507 | 33.5 | intron (NM_001199775, intron 2 of 20) | 24936 | CPD |
| chr1-175 | chr1 | 210771195 | 210771345 | 33.5 | intron (NM_018194, intron 10 of 11) | 268630 | HHAT |
| chr1-182 | chr1 | 8933777 | 8933927 | 33.5 | intron (NM_001428, intron 2 of 11) | -2217 | ENO1 |
| chr17-176 | chr17 | 79767133 | 79767283 | 33.5 | intron (NM_000160, intron 2 of 13) | 5198 | GCGR |
| chr19-165 | chr19 | 54511696 | 54511846 | 33.5 | intron (NM_145814, intron 3 of 3) | 16229 | CACNG6 |
| chr1-195 | chr1 | 43407485 | 43407635 | 33.5 | intron (NM_006516, intron 2 of 9) | -17160 | SLC2A1-AS1 |
| chr17-139 | chr17 | 15182546 | 15182696 | 33.5 | Intergenic | -13977 | PMP22 |
| chr2-169 | chr2 | 101815852 | 101816002 | 33.5 | Intergenic | -48081 | TBC1D8 |
| chr8-114 | chr8 | 63300106 | 63300256 | 33.5 | intron (NM_173688, intron 1 of 5) | 138680 | NKAIN3 |
| chr4-114 | chr4 | 169059434 | 169059584 | 33.5 | intron (NM_007193, intron 2 of 11) | 45821 | ANXA10 |
| chr8-113 | chr8 | 59754673 | 59754823 | 33.5 | intron (NM_014729, intron 4 of 8) | -182344 | NSMAF |
| chr1-181 | chr1 | 6661157 | 6661307 | 33.5 | intron (NM_014851, intron 1 of 3) | 1697 | KLHL21 |
| chr19-122 | chr19 | 50507231 | 50507381 | 33.5 | intron (NM_001025778, intron 4 of 13) | 21499 | VRK3 |
| chr20-78 | chr20 | 40272301 | 40272451 | 33.5 | Intergenic | -25243 | CHD6 |
| chr12-139 | chr12 | 49454496 | 49454646 | 33.5 | Intergenic | -5464 | MLL2 |
| chr3-121 | chr3 | 46713231 | 46713381 | 33.5 | intron (NR_033815, intron 24 of 25) | 5593 | ALS2CL |
| chr12-160 | chr12 | 108406972 | 108407122 | 33.5 | Intergenic | -109499 | LOC728739 |
| chr12-153 | chr12 | 95625086 | 95625236 | 33.5 | intron (NR_038241, intron 1 of 12) | 13639 | VEZT |
| chr6-71 | chr6 | 14777704 | 14777854 | 33.5 | Intergenic | -468427 | JARID2 |
| chr3-145 | chr3 | 148942996 | 148943146 | 33.5 | Intergenic | -3239 | CP |
| chr2-186 | chr2 | 234335417 | 234335567 | 33.5 | intron (NM_152879, intron 3 of 29) | 38692 | DGKD |
| chr9-118 | chr9 | 110856625 | 110856775 | 33.5 | Intergenic | -604653 | KLF4 |
| chr9-116 | chr9 | 98729666 | 98729816 | 33.5 | 3' UTR (NM_001010895, exon 14 of 14) | 54296 | LINC00092 |
| chr3-116 | chr3 | 33444266 | 33444416 | 33.5 | exon (NM_001128160, exon 8 of 15) | 37529 | UBP1 |
| chr1-148 | chr1 | 74507158 | 74507308 | 33.5 | exon (NM_001105659, exon 7 of 8) | 156638 | LRRIQ3 |
| chr10-199 | chr10 | 121201339 | 121201489 | 33.5 | intron (NM_005308, intron 10 of 15) | 63930 | MIR4681 |
| chr10-182 | chr10 | 62822780 | 62822930 | 33.5 | Intergenic | -61657 | RHOBTB1 |
| chr12-144 | chr12 | 58950151 | 58950301 | 33.5 | Intergenic | 363101 | LRIG3 |
| chr10-137 | chr10 | 37515556 | 37515706 | 33.5 | intron (NM_052997, intron 34 of 35) | 100846 | ANKRD30A |
| chr22-37 | chr22 | 44368658 | 44368808 | 33.5 | intron (NM_015380, intron 5 of 14) | 17472 | SAMM50 |
| chr19-129 | chr19 | 6530855 | 6531005 | 33.5 | promoter-TSS (NM_003811) | -80 | TNFSF9 |
| chrX-57 | chrX | 73251592 | 73251742 | 33.5 | non-coding (NR_028379, exon 6 of 7) | 87508 | JPX |
| chr7-143 | chr7 | 129505017 | 129505167 | 33.5 | intron (NM_003344, intron 3 of 6) | 87691 | UBE2H |
| chr3-152 | chr3 | 187990327 | 187990477 | 33.5 | intron (NM_005578, intron 2 of 10) | 47209 | LPP |
| chr2-207 | chr2 | 54342938 | 54343088 | 33.5 | intron (NM_138448, intron 1 of 3) | 603 | ACYP2 |
| chr3-137 | chr3 | 118910759 | 118910909 | 33.5 | intron (NM_006952, intron 5 of 7) | 18409 | UPK1B |
| chrX-60 | chrX | 100662848 | 100662998 | 33.5 | promoter-TSS (NM_000169) | 78 | GLA |
| chr5-143 | chr5 | 175310806 | 175310956 | 33.5 | 3' UTR (NM_006650, exon 5 of 5) | 12380 | CPLX2 |
| chr18-46 | chr18 | 55593121 | 55593271 | 33.5 | Intergenic | -118414 | NEDD4L |
| chr14-90 | chr14 | 105144198 | 105144348 | 33.5 | promoter-TSS (NR_039860) | -187 | MIR4710 |
| chr2-249 | chr2 | 224403029 | 224403179 | 33.5 | Intergenic | 64113 | SCG2 |
| chr20-57 | chr20 | 48293376 | 48293526 | 33.5 | intron (NM_004776, intron 1 of 8) | 36970 | B4GALT5 |
| chr12-138 | chr12 | 47615348 | 47615498 | 33.5 | intron (NM_138371, intron 1 of 1) | -5197 | PCED1B-AS1 |
| chr1-216 | chr1 | 151058725 | 151058875 | 33.5 | intron (NM_144618, intron 1 of 8) | 15720 | GABPB2 |
| chr17-142 | chr17 | 17699484 | 17699634 | 33.5 | exon (NM_030665, exon 3 of 6) | -16716 | SMCR5 |
| chr8-100 | chr8 | 8313407 | 8313557 | 33.5 | Intergenic | -74225 | SGK223 |
| chr10-169 | chr10 | 25138478 | 25138628 | 33.5 | 3' UTR (NM_020200, exon 9 of 9) | 102980 | PRTFDC1 |
| chr13-39 | chr13 | 101045420 | 101045570 | 33.5 | intron (NM_000282, intron 19 of 23) | 87774 | PCCA-AS1 |
| chr11-138 | chr11 | 66659762 | 66659912 | 33.5 | intron (NM_000920, intron 2 of 21) | 15503 | PC |
| chr2-253 | chr2 | 228316186 | 228316336 | 33.5 | Intergenic | -20587 | MIR5703 |
| chr7-125 | chr7 | 36566731 | 36566881 | 33.5 | intron (NM_001637, intron 19 of 20) | 72920 | AOAH-IT1 |
| chr15-78 | chr15 | 52385704 | 52385854 | 33.5 | Intergenic | 19193 | BCL2L10 |
| chr20-82 | chr20 | 48232219 | 48232369 | 33.5 | Intergenic | -47587 | PTGIS |
| chr1-143 | chr1 | 24127053 | 24127203 | 33.5 | promoter-TSS (NM_000403) | -94 | GALE |
| chr3-83 | chr3 | 29647568 | 29647718 | 33.5 | intron (NM_001003793, intron 4 of 14) | 324840 | RBMS3 |
| chr15-95 | chr15 | 32044705 | 32044855 | 33.5 | Intergenic | -97238 | OTUD7A |
| chr10-181 | chr10 | 53592804 | 53592954 | 33.5 | intron (NM_001098512, intron 4 of 17) | -133524 | CSTF2T |
| chr11-151 | chr11 | 110228541 | 110228691 | 33.5 | Intergenic | -61179 | RDX |
| chr11-141 | chr11 | 70303426 | 70303576 | 33.5 | Intergenic | 58889 | CTTN |
| chr2-174 | chr2 | 128171487 | 128171637 | 33.5 | Intergenic | -4434 | PROC |
| chr12-118 | chr12 | 94533915 | 94534065 | 33.5 | Intergenic | -8509 | PLXNC1 |
| chr2-240 | chr2 | 191724720 | 191724870 | 33.5 | Intergenic | -20752 | GLS |
| chr14-64 | chr14 | 81687717 | 81687867 | 33.5 | promoter-TSS (NM_015859) | -217 | GTF2A1 |
| chr22-40 | chr22 | 50279895 | 50280045 | 33.5 | exon (NM_014838, exon 2 of 2) | 32136 | ALG12 |
| chr14-86 | chr14 | 90240048 | 90240198 | 33.5 | Intergenic | -154629 | FOXN3 |
| chr5-108 | chr5 | 55652076 | 55652226 | 33.5 | Intergenic | -122965 | ANKRD55 |
| chr2-246 | chr2 | 219132345 | 219132495 | 33.5 | intron (NM_001087, intron 2 of 10) | 2473 | AAMP |
| chr11-112 | chr11 | 84275636 | 84275786 | 33.5 | intron (NM_001142699, intron 6 of 27) | -247329 | DLG2 |
| chr7-137 | chr7 | 97989954 | 97990104 | 33.5 | intron (NM_018842, intron 2 of 13) | 40398 | BAIAP2L1 |
| chr19-157 | chr19 | 45242534 | 45242684 | 33.5 | Intergenic | -9369 | BCL3 |
| chr1-200 | chr1 | 61607252 | 61607402 | 33.5 | intron (NM_001134673, intron 2 of 10) | 59347 | NFIA |
| chr7-132 | chr7 | 68190113 | 68190263 | 33.5 | Intergenic | -873717 | AUTS2 |
| chr11-120 | chr11 | 10004824 | 10004974 | 33.5 | intron (NM_030962, intron 13 of 39) | -223819 | LOC440028 |
| chr10-186 | chr10 | 66585645 | 66585795 | 33.5 | non-coding (NR_001446, exon 1 of 1) | 435 | ANXA2P3 |
| chr11-101 | chr11 | 46951696 | 46951846 | 33.5 | Intergenic | -6480 | C11orf49 |
| chr1-187 | chr1 | 23076942 | 23077092 | 33.5 | intron (NM_017449, intron 1 of 15) | 31007 | MIR4684 |
| chr12-127 | chr12 | 928084 | 928234 | 33.5 | intron (NM_014823, intron 2 of 25) | 66070 | WNK1 |
| chr10-159 | chr10 | 4720259 | 4720409 | 33.5 | promoter-TSS (NR_024475) | -72 | LOC100216001 |
| chr15-85 | chr15 | 69803507 | 69803657 | 33.5 | Intergenic | -50477 | LOC145837 |
| chr3-127 | chr3 | 78873067 | 78873217 | 33.5 | intron (NM_133631, intron 2 of 28) | 195467 | ROBO1 |
| chr8-110 | chr8 | 54680859 | 54681009 | 33.5 | intron (NM_015941, intron 11 of 13) | 74668 | ATP6V1H |
| chr8-126 | chr8 | 110490517 | 110490667 | 33.5 | intron (NM_177531, intron 53 of 77) | -61337 | EBAG9 |
| chr22-29 | chr22 | 18264606 | 18264756 | 33.5 | Intergenic | -7250 | BID |
| chr3-161 | chr3 | 196758327 | 196758477 | 33.5 | Intergenic | -1715 | MFI2 |
| chr5-96 | chr5 | 1471046 | 1471196 | 33.5 | intron (NM_024830, intron 11 of 13) | -25578 | SLC6A3 |
| chr2-197 | chr2 | 29032681 | 29032831 | 33.5 | promoter-TSS (NM_001142634) | -944 | SPDYA |
| chr21-31 | chr21 | 33836440 | 33836590 | 33.5 | intron (NM_058187, intron 3 of 7) | 51763 | FAM176C |
| chr5-112 | chr5 | 58236286 | 58236436 | 33.5 | Intergenic | 59398 | PDE4D |
| chr4-87 | chr4 | 39975138 | 39975288 | 33.5 | intron (NM_001100399, intron 2 of 32) | 4363 | PDS5A |
| chrX-53 | chrX | 44429876 | 44430026 | 33.5 | Intergenic | -27730 | FUNDC1 |
| chr14-80 | chr14 | 54656511 | 54656661 | 33.5 | Intergenic | -207087 | CDKN3 |
| chr2-210 | chr2 | 63041364 | 63041514 | 33.5 | intron (NM_001142616, intron 5 of 22) | 107407 | EHBP1 |
| chr3-93 | chr3 | 98533993 | 98534143 | 33.5 | intron (NM_080927, intron 9 of 15) | 82520 | ST3GAL6 |
| chr12-167 | chr12 | 120557477 | 120557627 | 33.5 | Intergenic | -2909 | RAB35 |
| chr16-120 | chr16 | 83841524 | 83841674 | 33.5 | promoter-TSS (NM_001537) | 91 | HSBP1 |
| chr3-141 | chr3 | 142038132 | 142038282 | 33.5 | intron (NM_019001, intron 37 of 41) | -93758 | GK5 |
| chr10-115 | chr10 | 21581954 | 21582104 | 33.5 | Intergenic | 118798 | LOC100128511 |
| chr15-100 | chr15 | 51837903 | 51838053 | 33.5 | intron (NM_001174116, intron 7 of 42) | 76989 | DMXL2 |
| chr1-191 | chr1 | 31440965 | 31441115 | 33.5 | promoter-TSS (NR_003066) | 44 | SNORD85 |
| chr2-200 | chr2 | 42149425 | 42149575 | 33.5 | Intergenic | 31443 | C2orf91 |
| chr9-121 | chr9 | 116840717 | 116840867 | 33.5 | promoter-TSS (NM_001633) | -40 | AMBP |
| chr3-132 | chr3 | 100629662 | 100629812 | 33.5 | intron (NM_015429, intron 2 of 34) | 82597 | ABI3BP |
| chr15-126 | chr15 | 92257378 | 92257528 | 33.5 | Intergenic | -139485 | SLCO3A1 |
| chr5-124 | chr5 | 90279122 | 90279272 | 33.5 | intron (NM_032119, intron 84 of 89) | -396967 | ARRDC3-AS1 |
| chr6-94 | chr6 | 144663360 | 144663510 | 33.5 | intron (NM_007124, intron 1 of 73) | 50562 | UTRN |
| chr3-160 | chr3 | 196066737 | 196066887 | 33.5 | Intergenic | -1521 | TM4SF19 |
| chr11-117 | chr11 | 122727386 | 122727536 | 33.5 | intron (NM_019604, intron 5 of 9) | 18206 | CRTAM |
| chr5-121 | chr5 | 74147893 | 74148043 | 33.5 | intron (NM_015566, intron 1 of 12) | 14244 | FAM169A |
| chr1-225 | chr1 | 172173535 | 172173685 | 33.5 | intron (NM_001136127, intron 14 of 19) | -59635 | DNM3OS |
| chr7-121 | chr7 | 24517332 | 24517482 | 33.5 | Intergenic | -95678 | MPP6 |
| chr4-113 | chr4 | 160948043 | 160948193 | 33.5 | Intergenic | 759120 | RAPGEF2 |
| chr11-122 | chr11 | 31014140 | 31014290 | 33.5 | promoter-TSS (NM_020869) | 18 | DCDC5 |
| chr6-84 | chr6 | 84577372 | 84577522 | 33.5 | intron (NM_016230, intron 2 of 15) | 8077 | CYB5R4 |
| chr12-156 | chr12 | 104671239 | 104671389 | 33.5 | intron (NM_001093771, intron 3 of 16) | -9146 | TXNRD1 |
| chr10-145 | chr10 | 69426291 | 69426441 | 33.5 | promoter-TSS (NM_001127384) | -950 | CTNNA3 |
| chr2-195 | chr2 | 25194781 | 25194931 | 33.5 | promoter-TSS (NR_034113) | 107 | DNAJC27 |
| chr7-128 | chr7 | 47823654 | 47823804 | 33.5 | intron (NM_138295, intron 56 of 56) | -11160 | C7orf69 |
| chr19-128 | chr19 | 4841052 | 4841202 | 33.5 | intron (NM_001164194, intron 7 of 7) | -9373 | TICAM1 |
| chr15-87 | chr15 | 72044570 | 72044720 | 33.5 | intron (NM_024817, intron 13 of 16) | -58249 | NR2E3 |
| chr8-118 | chr8 | 74332812 | 74332962 | 33.5 | 3' UTR (NM_001164380, exon 15 of 15) | 578 | STAU2-AS1 |
| chr1-139 | chr1 | 8065320 | 8065470 | 33.5 | Intergenic | 20998 | ERRFI1 |
| chr2-257 | chr2 | 236080208 | 236080358 | 33.5 | Intergenic | 219655 | SH3BP4 |
| chr3-104 | chr3 | 152094244 | 152094394 | 33.5 | intron (NM_021038, intron 2 of 9) | -35540 | TMEM14E |
| chr2-260 | chr2 | 242775684 | 242775834 | 33.5 | Intergenic | 23729 | NEU4 |
| chr13-32 | chr13 | 30096503 | 30096653 | 33.5 | exon (NM_003045, exon 8 of 13) | -34691 | MTUS2-AS1 |
| chr20-51 | chr20 | 14528227 | 14528377 | 33.5 | intron (NM_080676, intron 4 of 16) | -209989 | FLRT3 |
| chr1-193 | chr1 | 33794391 | 33794541 | 33.5 | intron (NM_004427, intron 6 of 6) | 3627 | MIR3605 |
| chr10-173 | chr10 | 30846977 | 30847127 | 33.5 | Intergenic | 71595 | LYZL2 |
| chr1-231 | chr1 | 202032327 | 202032477 | 33.5 | Intergenic | 52712 | ELF3 |
| chr1-203 | chr1 | 89981704 | 89981854 | 33.5 | Intergenic | -8618 | LRRC8B |
| chr17-132 | chr17 | 2587892 | 2588042 | 33.5 | 3' UTR (NM_000430, exon 11 of 11) | 26960 | KIAA0664 |
| chr3-155 | chr3 | 189749963 | 189750113 | 33.5 | intron (NM_001134418, intron 1 of 14) | 88870 | LEPREL1 |
| chr18-55 | chr18 | 48277890 | 48278040 | 33.5 | Intergenic | 68469 | MRO |
| chr10-192 | chr10 | 90961223 | 90961373 | 33.5 | Intergenic | 5773 | CH25H |
| chr9-87 | chr9 | 37472488 | 37472638 | 33.5 | Intergenic | -7156 | ZBTB5 |
| chr5-142 | chr5 | 174623435 | 174623585 | 33.5 | Intergenic | -200776 | FLJ16171 |
| chr3-142 | chr3 | 142695575 | 142695725 | 33.5 | Intergenic | -13472 | PAQR9 |
| chr10-163 | chr10 | 11726905 | 11727055 | 33.5 | Intergenic | -57376 | ECHDC3 |
| chr8-87 | chr8 | 67228174 | 67228324 | 33.5 | Intergenic | 112963 | LOC100505676 |
| chr15-99 | chr15 | 50528155 | 50528305 | 33.5 | exon (NM_001159629, exon 9 of 9) | 29932 | HDC |
| chr10-160 | chr10 | 4999208 | 4999358 | 33.5 | Intergenic | -6171 | AKR1C1 |
| chr12-145 | chr12 | 71065837 | 71065987 | 33.5 | intron (NM_001207016, intron 7 of 10) | -34692 | PTPRB |
| chr2-239 | chr2 | 179107099 | 179107249 | 33.5 | intron (NM_001201481, intron 1 of 23) | 47966 | OSBPL6 |
| chr6-115 | chr6 | 125378830 | 125378980 | 33.5 | intron (NM_152553, intron 4 of 8) | 74391 | RNF217 |
| chr9-117 | chr9 | 108563523 | 108563673 | 33.5 | Intergenic | 106773 | TMEM38B |
| chr3-67 | chr3 | 97732665 | 97732815 | 33.5 | intron (NR_047685, intron 4 of 9) | 21408 | GABRR3 |
| chrX-56 | chrX | 70473966 | 70474116 | 33.5 | promoter-TSS (NM_201599) | -3 | ZMYM3 |
| chr5-139 | chr5 | 169578242 | 169578392 | 33.5 | Intergenic | 45400 | FOXI1 |
| chr2-250 | chr2 | 224428281 | 224428431 | 33.5 | Intergenic | 38861 | SCG2 |
| chr1-149 | chr1 | 77813492 | 77813642 | 33.5 | intron (NM_174858, intron 6 of 13) | 65280 | AK5 |
| chr19-142 | chr19 | 33784300 | 33784450 | 33.5 | Intergenic | 9055 | CEBPA |
| chr5-144 | chr5 | 176719083 | 176719233 | 33.5 | exon (NM_022455, exon 22 of 23) | 11586 | RAB24 |
| chr2-226 | chr2 | 102711595 | 102711745 | 33.5 | Intergenic | -58732 | IL1R1 |
| chr6-85 | chr6 | 97346444 | 97346594 | 33.5 | promoter-TSS (NM_014165) | -752 | NDUFAF4 |
| chr8-120 | chr8 | 86873982 | 86874132 | 33.5 | Intergenic | -33886 | REXO1L2P |
| chr5-119 | chr5 | 67640333 | 67640483 | 33.5 | Intergenic | 52012 | PIK3R1 |
| chr17-153 | chr17 | 39176802 | 39176952 | 33.5 | Intergenic | 6577 | KRTAP1-5 |
| chr8-141 | chr8 | 143743778 | 143743928 | 33.5 | 3' UTR (NM_003724, exon 3 of 3) | 7548 | JRK |
| chr4-83 | chr4 | 25863239 | 25863389 | 33.5 | intron (NM_015187, intron 1 of 23) | 1296 | SEL1L3 |
| chr8-115 | chr8 | 71085070 | 71085220 | 33.5 | intron (NM_006540, intron 5 of 22) | -101583 | PRDM14 |
| chr20-84 | chr20 | 52369765 | 52369915 | 33.5 | Intergenic | 122408 | SUMO1P1 |
| chr20-77 | chr20 | 35389674 | 35389824 | 33.5 | intron (NM_001145316, intron 6 of 10) | 12405 | DSN1 |
| chr4-103 | chr4 | 91766980 | 91767130 | 33.5 | intron (NM_001145065, intron 8 of 10) | 610873 | FAM190A |
| chr19-164 | chr19 | 53454086 | 53454236 | 33.5 | exon (NM_001202456, exon 4 of 4) | -8314 | ZNF321P |
| chr3-96 | chr3 | 119122135 | 119122285 | 33.5 | intron (NM_020754, intron 10 of 11) | 60261 | TMEM39A |
| chr17-108 | chr17 | 27684939 | 27685089 | 33.5 | Intergenic | -32666 | MIR4523 |
| chr10-189 | chr10 | 74554127 | 74554277 | 33.5 | intron (NM_001270680, intron 1 of 7) | 73415 | MIR4676 |
| chr8-80 | chr8 | 19523047 | 19523197 | 33.5 | intron (NR_024040, intron 1 of 9) | 16972 | CSGALNACT1 |
| chr18-44 | chr18 | 19248142 | 19248292 | 33.5 | intron (NM_138340, intron 4 of 8) | -15254 | MIR320C1 |
| chr16-96 | chr16 | 23550062 | 23550212 | 33.5 | intron (NR_003501, intron 3 of 9) | 18559 | EARS2 |
| chr19-134 | chr19 | 19222987 | 19223137 | 33.5 | 3' UTR (NM_178526, exon 8 of 8) | 26248 | TMEM161A |
| chr3-97 | chr3 | 121370677 | 121370827 | 33.5 | intron (NM_005335, intron 3 of 13) | 9039 | HCLS1 |
| chr19-156 | chr19 | 45189339 | 45189489 | 33.5 | Intergenic | -13007 | CEACAM16 |
| chr4-86 | chr4 | 38858454 | 38858604 | 33.5 | promoter-TSS (NM_006068) | -91 | TLR6 |
| chr8-136 | chr8 | 134387855 | 134388005 | 33.5 | Intergenic | -78383 | NDRG1 |
| chr6-86 | chr6 | 105970671 | 105970821 | 33.5 | Intergenic | -119747 | PREP |
| chr12-101 | chr12 | 7071325 | 7071475 | 33.5 | TTS (NM_002831) | -1462 | MIR200C |
| chr15-82 | chr15 | 63659661 | 63659811 | 33.5 | intron (NM_206925, intron 2 of 9) | 14339 | CA12 |
| chr22-41 | chr22 | 50364543 | 50364693 | 33.5 | Intergenic | 10475 | PIM3 |
| chr7-89 | chr7 | 32708853 | 32709003 | 33.5 | intron (NR_036680, intron 5 of 16) | 49852 | DPY19L1P1 |
| chr3-125 | chr3 | 58038143 | 58038293 | 33.5 | intron (NM_001164317, intron 1 of 46) | 44091 | FLNB |
| chr7-131 | chr7 | 65705388 | 65705538 | 33.5 | exon (NM_003596, exon 2 of 6) | 35204 | TPST1 |
| chr1-196 | chr1 | 45196516 | 45196666 | 33.5 | Intergenic | -8899 | KIF2C |
| chr5-82 | chr5 | 75919208 | 75919358 | 33.5 | promoter-TSS (NM_001256566) | -24 | F2RL2 |
| chr10-166 | chr10 | 21464203 | 21464353 | 33.5 | TTS (NR_046283) | 1047 | LOC100128511 |
| chr20-62 | chr20 | 957325 | 957475 | 33.5 | intron (NM_001040007, intron 1 of 3) | 25507 | RSPO4 |
| chr9-91 | chr9 | 75733904 | 75734054 | 33.5 | Intergenic | -32802 | ANXA1 |
| chr3-113 | chr3 | 27236669 | 27236819 | 33.5 | Intergenic | 174168 | NEK10 |
| chr20-56 | chr20 | 46020950 | 46021100 | 33.5 | Intergenic | -35551 | ZMYND8 |
| chr1-162 | chr1 | 154943229 | 154943379 | 33.5 | promoter-TSS (NM_183001) | -81 | SHC1 |
| chr7-118 | chr7 | 14381244 | 14381394 | 33.5 | intron (NM_004080, intron 21 of 24) | -350269 | ETV1 |
| chr11-128 | chr11 | 35184844 | 35184994 | 33.5 | intron (NM_001001389, intron 1 of 16) | 24502 | CD44 |
| chr5-97 | chr5 | 10525401 | 10525551 | 33.5 | Intergenic | -38959 | ANKRD33B |
| chr3-151 | chr3 | 186395903 | 186396053 | 33.5 | TTS (NM_000412) | 12180 | HRG |
| chr14-45 | chr14 | 65409507 | 65409657 | 33.5 | promoter-TSS (NR_046321) | 41 | GPX2 |
| chr3-110 | chr3 | 5391090 | 5391240 | 33.5 | Intergenic | -99225 | MIR4790 |
| chr2-206 | chr2 | 48573404 | 48573554 | 33.5 | exon (NM_002158, exon 3 of 7) | 31684 | FOXN2 |
| chr3-82 | chr3 | 15672632 | 15672782 | 33.5 | intron (NM_000060, intron 1 of 3) | 29452 | BTD |
| chr2-230 | chr2 | 120566421 | 120566571 | 33.5 | intron (NM_002830, intron 1 of 26) | 49289 | PTPN4 |
| chr17-152 | chr17 | 38471011 | 38471161 | 33.5 | intron (NM_000964, intron 1 of 8) | -3387 | RARA |
| chr3-131 | chr3 | 100349588 | 100349738 | 33.5 | intron (NM_032787, intron 3 of 15) | 21230 | GPR128 |
| chr3-136 | chr3 | 114217671 | 114217821 | 33.5 | intron (NM_001164346, intron 2 of 5) | -115257 | ZBTB20 |
| chr1-177 | chr1 | 219262416 | 219262566 | 33.5 | intron (NR_038845, intron 3 of 4) | 84639 | LOC643723 |
| chr18-40 | chr18 | 3618567 | 3618717 | 33.5 | intron (NM_001003809, intron 4 of 9) | 24530 | DLGAP1-AS1 |
| chr3-92 | chr3 | 66604725 | 66604875 | 33.5 | Intergenic | -53955 | LRIG1 |
| chr12-140 | chr12 | 51009983 | 51010133 | 33.5 | intron (NM_173602, intron 1 of 37) | 111290 | DIP2B |
| chr5-138 | chr5 | 166310154 | 166310304 | 33.5 | Intergenic | -401614 | TENM2 |
| chr10-200 | chr10 | 126920210 | 126920360 | 33.5 | Intergenic | -70661 | CTBP2 |
| chr9-113 | chr9 | 91258958 | 91259108 | 33.5 | Intergenic | 8042 | LOC286238 |
| chrX-36 | chrX | 46838896 | 46839046 | 33.5 | intron (NM_014735, intron 1 of 10) | 67103 | PHF16 |
| chrX-52 | chrX | 39921921 | 39922071 | 33.5 | intron (NM_001123385, intron 9 of 14) | 34723 | BCOR |
| chr6-104 | chr6 | 49508918 | 49509068 | 33.5 | Intergenic | -9120 | C6orf141 |
| chr2-211 | chr2 | 66998833 | 66998983 | 33.5 | Intergenic | 336376 | MEIS1 |
| chr12-166 | chr12 | 118454437 | 118454587 | 33.5 | promoter-TSS (NM_007370) | 6 | RFC5 |
| chr17-135 | chr17 | 8079699 | 8079849 | 33.5 | promoter-TSS (NM_183065) | -60 | TMEM107 |
| chr4-95 | chr4 | 57943911 | 57944061 | 33.5 | intron (NM_001553, intron 1 of 4) | -31942 | LOC255130 |
| chr22-35 | chr22 | 40723279 | 40723429 | 33.5 | 3' UTR (NM_015088, exon 21 of 21) | -19150 | ADSL |
| chr15-81 | chr15 | 57738164 | 57738314 | 33.5 | intron (NM_001252335, intron 5 of 19) | 69536 | CGNL1 |
| chr10-196 | chr10 | 118729698 | 118729848 | 33.5 | intron (NM_001258298, intron 1 of 15) | 35315 | KIAA1598 |
| chr6-119 | chr6 | 152665216 | 152665366 | 33.5 | exon (NM_033071, exon 73 of 146) | 292695 | SYNE1 |
| chr1-229 | chr1 | 182764867 | 182765017 | 33.5 | intron (NM_001200052, intron 3 of 11) | 6358 | NPL |
| chr17-170 | chr17 | 71394114 | 71394264 | 33.5 | exon (NM_001144952, exon 24 of 45) | -86046 | CDC42EP4 |
| chr3-126 | chr3 | 78368071 | 78368221 | 33.5 | Intergenic | 700463 | ROBO1 |
| chr1-238 | chr1 | 218672140 | 218672290 | 33.5 | Intergenic | -153195 | LOC728463 |
| chr15-103 | chr15 | 59226162 | 59226312 | 33.5 | promoter-TSS (NM_001013843) | -385 | SLTM |
| chr19-159 | chr19 | 47289757 | 47289907 | 33.5 | intron (NM_005628, intron 1 of 7) | 713 | SLC1A5 |
| chr20-66 | chr20 | 4139548 | 4139698 | 33.5 | intron (NM_001270691, intron 1 of 7) | 10197 | SMOX |
| chr1-241 | chr1 | 242253269 | 242253419 | 33.5 | exon (NM_001195812, exon 9 of 9) | -90959 | MAP1LC3C |
| chr8-109 | chr8 | 50968919 | 50969069 | 33.5 | intron (NM_018967, intron 1 of 18) | 144397 | SNTG1 |
| chr2-160 | chr2 | 70914179 | 70914329 | 33.5 | intron (NM_001185054, intron 9 of 15) | 80593 | ADD2 |
| chr12-134 | chr12 | 16704003 | 16704153 | 33.5 | non-coding (NR_045014, exon 4 of 4) | 54235 | LMO3 |
| chr1-185 | chr1 | 18995435 | 18995585 | 33.5 | intron (NM_001135254, intron 4 of 8) | 38010 | PAX7 |
| chr21-30 | chr21 | 27778136 | 27778286 | 33.5 | Intergenic | 167370 | CYYR1 |
| chr10-185 | chr10 | 65183389 | 65183539 | 33.5 | intron (NM_032776, intron 1 of 25) | -41525 | LOC84989 |
| chr16-74 | chr16 | 19302816 | 19302966 | 33.5 | intron (NM_001256720, intron 1 of 4) | 5786 | CLEC19A |
| chr15-116 | chr15 | 72632111 | 72632261 | 33.5 | Intergenic | -19661 | CELF6 |
| chr8-103 | chr8 | 19262614 | 19262764 | 33.5 | non-coding (NR_024040, exon 10 of 10) | 91202 | SH2D4A |
| chr1-184 | chr1 | 16472160 | 16472310 | 33.5 | intron (NM_004431, intron 3 of 16) | 10347 | EPHA2 |
| chr1-237 | chr1 | 216850725 | 216850875 | 33.5 | exon (NM_001438, exon 2 of 7) | 46014 | ESRRG |
| chr7-149 | chr7 | 151202630 | 151202780 | 33.5 | intron (NM_005614, intron 1 of 7) | 14305 | RHEB |
| chr4-82 | chr4 | 14380907 | 14381057 | 33.5 | Intergenic | 267390 | LOC152742 |
| chr9-102 | chr9 | 2718981 | 2719131 | 33.5 | exon (NM_133497, exon 1 of 2) | 1530 | KCNV2 |
| chr20-74 | chr20 | 24727117 | 24727267 | 33.5 | Intergenic | -202674 | CST7 |
| chr16-95 | chr16 | 22309568 | 22309718 | 33.5 | intron (NM_001258035, intron 1 of 19) | 947 | POLR3E |
| chr10-184 | chr10 | 65176259 | 65176409 | 33.5 | intron (NM_032776, intron 1 of 25) | -43526 | MIR1296 |
| chr1-211 | chr1 | 147191237 | 147191387 | 33.5 | Intergenic | 41402 | GJA5 |
| chr8-140 | chr8 | 142464975 | 142465125 | 33.5 | intron (NM_207414, intron 19 of 29) | 33043 | PTP4A3 |
| chr16-109 | chr16 | 57481250 | 57481400 | 33.5 | promoter-TSS (NM_020313) | -12 | COQ9 |
| chr20-52 | chr20 | 22980536 | 22980686 | 33.5 | Intergenic | -35446 | SSTR4 |
| chr7-114 | chr7 | 4158354 | 4158504 | 33.5 | intron (NM_152744, intron 25 of 44) | -10888 | SDK1 |
| chr1-167 | chr1 | 170513976 | 170514126 | 33.5 | 3' UTR (NM_001146039, exon 4 of 4) | 12788 | GORAB |
| chr7-142 | chr7 | 116772913 | 116773063 | 33.5 | intron (NM_018412, intron 6 of 14) | 12626 | ST7-AS2 |
| chr6-111 | chr6 | 102483336 | 102483486 | 33.5 | exon (NM_021956, exon 14 of 16) | 636550 | GRIK2 |
| chrX-51 | chrX | 29680880 | 29681030 | 33.5 | intron (NM_014271, intron 5 of 10) | -552720 | MAGEB2 |
| chr17-172 | chr17 | 74656053 | 74656203 | 33.5 | Intergenic | -16234 | ST6GALNAC1 |
| chr16-77 | chr16 | 30076731 | 30076881 | 33.5 | promoter-TSS (NM_184041) | -188 | ALDOA |
| chr12-115 | chr12 | 87530220 | 87530370 | 33.5 | Intergenic | -297614 | MGAT4C |
| chr12-151 | chr12 | 93251965 | 93252115 | 33.5 | intron (NM_003566, intron 3 of 28) | 71067 | EEA1 |
| chr2-147 | chr2 | 69461 | 69611 | 33.5 | Intergenic | -22948 | FAM110C |
| chr19-147 | chr19 | 37210442 | 37210592 | 33.5 | exon (NM_152603, exon 4 of 4) | 30215 | ZNF567 |
| chr3-118 | chr3 | 34098469 | 34098619 | 33.5 | Intergenic | 258481 | PDCD6IP |
| chr12-113 | chr12 | 78333741 | 78333891 | 33.5 | intron (NM_014903, intron 1 of 38) | 108747 | NAV3 |
| chr4-102 | chr4 | 87884967 | 87885117 | 33.5 | intron (NM_001166693, intron 2 of 20) | 28888 | AFF1 |
| chr2-212 | chr2 | 70520807 | 70520957 | 33.5 | promoter-TSS (NM_003096) | -13 | SNRPG |
| chr2-215 | chr2 | 77328750 | 77328900 | 33.5 | intron (NM_001134745, intron 3 of 3) | 420677 | LRRTM4 |
| chr12-162 | chr12 | 109227609 | 109227759 | 33.5 | intron (NM_001161330, intron 2 of 13) | -6357 | SSH1 |
| chr1-199 | chr1 | 49344175 | 49344325 | 33.5 | intron (NM_032785, intron 5 of 13) | -101703 | BEND5 |
| chr19-127 | chr19 | 2434200 | 2434350 | 33.5 | intron (NM_032737, intron 7 of 11) | -6400 | TIMM13 |
| chr19-120 | chr19 | 46571966 | 46572116 | 33.5 | Intergenic | -27767 | IGFL4 |
| chr10-135 | chr10 | 21653877 | 21654027 | 33.5 | Intergenic | 131618 | MIR1915 |
| chr12-132 | chr12 | 10169565 | 10169715 | 33.5 | 3' UTR (NM_205852, exon 5 of 5) | 6409 | CLEC12B |
| chr3-95 | chr3 | 111286483 | 111286633 | 33.5 | intron (NM_005816, intron 3 of 13) | 25632 | CD96 |
| chr1-230 | chr1 | 182836243 | 182836393 | 33.5 | intron (NM_001357, intron 14 of 27) | 27879 | DHX9 |
| chr21-17 | chr21 | 30558686 | 30558836 | 33.5 | Intergenic | -7054 | LINC00189 |
| chr2-242 | chr2 | 206892718 | 206892868 | 33.5 | intron (NM_017759, intron 6 of 10) | 58113 | INO80D |
| chr11-144 | chr11 | 77705667 | 77705817 | 33.5 | promoter-TSS (NM_033547) | -25 | INTS4 |
| chr5-129 | chr5 | 140615284 | 140615434 | 33.5 | non-coding (NR_001281, exon 1 of 1) | 1421 | PCDHB18 |
| chr5-134 | chr5 | 148463732 | 148463882 | 33.5 | Intergenic | -21070 | SH3TC2 |
| chr8-121 | chr8 | 87569206 | 87569356 | 33.5 | intron (NM_003909, intron 16 of 16) | 42625 | CPNE3 |
| chr14-77 | chr14 | 53301380 | 53301530 | 33.5 | Intergenic | -43069 | GNPNAT1 |
| chr17-168 | chr17 | 70130725 | 70130875 | 33.5 | Intergenic | 13639 | SOX9 |
| chr7-138 | chr7 | 99679340 | 99679490 | 33.5 | promoter-TSS (NM_032924) | -44 | ZNF3 |
| chr19-155 | chr19 | 42873040 | 42873190 | 33.5 | exon (NM_001410, exon 36 of 41) | 21329 | CNFN |
| chr15-128 | chr15 | 99343677 | 99343827 | 33.5 | intron (NM_000875, intron 2 of 20) | 16097 | MIR4714 |
| chr7-123 | chr7 | 30325659 | 30325809 | 33.5 | intron (NM_147128, intron 1 of 4) | 1811 | ZNRF2 |
| chr1-223 | chr1 | 167789319 | 167789469 | 33.5 | intron (NM_018417, intron 30 of 32) | 94059 | ADCY10 |
| chrX-62 | chrX | 107639733 | 107639883 | 33.5 | intron (NM_001847, intron 2 of 44) | 41850 | COL4A6 |
| chr4-80 | chr4 | 6883263 | 6883413 | 33.5 | 3' UTR (NM_001100590, exon 9 of 9) | -27833 | TBC1D14 |
| chr18-41 | chr18 | 9017232 | 9017382 | 33.5 | Intergenic | -85321 | NDUFV2 |
| chr7-91 | chr7 | 43601668 | 43601818 | 33.5 | 3' UTR (NM_015052, exon 30 of 30) | -20949 | STK17A |
| chr17-86 | chr17 | 20837718 | 20837868 | 33.5 | Intergenic | -38340 | CCDC144NL |
| chr4-112 | chr4 | 159918037 | 159918187 | 33.5 | intron (NM_152543, intron 1 of 4) | 38221 | C4orf45 |
| chr15-88 | chr15 | 75182340 | 75182490 | 33.5 | promoter-TSS (NM_002435) | 5 | MPI |
| chr8-104 | chr8 | 27730158 | 27730308 | 33.5 | intron (NM_173833, intron 8 of 8) | 13400 | MIR4287 |
| chr15-112 | chr15 | 70289037 | 70289187 | 33.5 | Intergenic | 82695 | MIR629 |
| chr2-191 | chr2 | 11884289 | 11884439 | 33.5 | intron (NM_001261427, intron 2 of 20) | -2358 | LPIN1 |
| chr1-226 | chr1 | 179187201 | 179187351 | 33.5 | intron (NM_007314, intron 1 of 11) | 11543 | ABL2 |
| chr15-111 | chr15 | 69807905 | 69808055 | 33.5 | Intergenic | -46079 | LOC145837 |
| chr3-148 | chr3 | 154900473 | 154900623 | 33.5 | 3' UTR (NM_007289, exon 23 of 23) | 102469 | MME |
| chr17-178 | chr17 | 79892261 | 79892411 | 33.5 | exon (NM_006907, exon 6 of 7) | 2632 | PYCR1 |
| chr16-116 | chr16 | 72867021 | 72867171 | 33.5 | intron (NM_006885, intron 4 of 9) | 215178 | ZFHX3 |
| chr7-83 | chr7 | 2996415 | 2996565 | 33.5 | intron (NM_032415, intron 2 of 24) | 87019 | CARD11 |
| chr3-159 | chr3 | 196006358 | 196006508 | 33.5 | intron (NM_005017, intron 2 of 9) | 8151 | PCYT1A |
| chr5-118 | chr5 | 63921607 | 63921757 | 33.5 | Intergenic | -64453 | FAM159B |
| chr2-229 | chr2 | 115017019 | 115017169 | 33.5 | Intergenic | -182805 | DPP10 |
| chr14-72 | chr14 | 23504360 | 23504510 | 33.5 | promoter-TSS (NM_002797) | -6 | PSMB5 |
| chr3-114 | chr3 | 27719066 | 27719216 | 33.5 | Intergenic | 44644 | EOMES |
| chr12-137 | chr12 | 45628293 | 45628443 | 33.5 | intron (NM_001025356, intron 1 of 19) | -18579 | PLEKHA8P1 |
| chr19-150 | chr19 | 39834932 | 39835082 | 33.5 | intron (NM_018028, intron 1 of 15) | 1899 | SAMD4B |
| chr8-128 | chr8 | 119001783 | 119001933 | 33.5 | intron (NM_000127, intron 1 of 10) | 122200 | EXT1 |
| chr3-130 | chr3 | 98377116 | 98377266 | 33.5 | Intergenic | -64736 | CPOX |
| chr11-126 | chr11 | 34361455 | 34361605 | 33.5 | intron (NM_145804, intron 1 of 16) | 18025 | ABTB2 |
| chr7-139 | chr7 | 107262304 | 107262454 | 33.5 | non-coding (NR_027830, exon 7 of 7) | -38701 | SLC26A4 |
| chr2-198 | chr2 | 32037396 | 32037546 | 33.5 | Intergenic | 198227 | MEMO1 |
| chr5-94 | chr5 | 171533573 | 171533723 | 33.5 | exon (NM_005990, exon 6 of 19) | 81698 | STK10 |
| chr13-23 | chr13 | 21750693 | 21750843 | 33.5 | promoter-TSS (NM_001166017) | -27 | SKA3 |
| chr5-125 | chr5 | 100416279 | 100416429 | 33.5 | Intergenic | -177367 | ST8SIA4 |
| chr19-103 | chr19 | 4374703 | 4374853 | 33.5 | intron (NM_001199944, intron 1 of 9) | 25637 | SH3GL1 |
| chr19-162 | chr19 | 51667615 | 51667765 | 33.5 | Intergenic | -2895 | SIGLEC17P |
| chr10-162 | chr10 | 7299329 | 7299479 | 33.5 | intron (NM_001018039, intron 7 of 20) | 151899 | SFMBT2 |
| chr12-123 | chr12 | 107790858 | 107791008 | 33.5 | intron (NM_001018072, intron 1 of 16) | 78736 | BTBD11 |
| chr9-106 | chr9 | 8485702 | 8485852 | 33.5 | exon (NM_002839, exon 28 of 46) | 248169 | PTPRD |
| chr16-99 | chr16 | 27351395 | 27351545 | 33.5 | intron (NM_000418, intron 2 of 10) | 26240 | IL4R |
| chr9-122 | chr9 | 121371747 | 121371897 | 33.5 | Intergenic | 759917 | DBC1 |
| chr8-137 | chr8 | 141474303 | 141474453 | 33.5 | Intergenic | -5700 | TRAPPC9 |
| chr9-83 | chr9 | 32946287 | 32946437 | 33.5 | Intergenic | 55212 | APTX |
| chr20-70 | chr20 | 19672855 | 19673005 | 33.5 | intron (NM_020689, intron 12 of 16) | -194235 | RIN2 |
| chr7-153 | chr7 | 157283294 | 157283444 | 33.5 | Intergenic | 83745 | MIR153-2 |
| chr12-131 | chr12 | 6974965 | 6975115 | 33.5 | intron (NM_001098536, intron 19 of 19) | -1544 | TPI1 |
| chr1-234 | chr1 | 209953757 | 209953907 | 33.5 | exon (NM_025228, exon 15 of 17) | 4058 | C1orf74 |
| chr2-156 | chr2 | 45252170 | 45252320 | 33.5 | Intergenic | -15703 | SIX2 |
| chr15-106 | chr15 | 65303702 | 65303852 | 33.5 | intron (NM_139242, intron 6 of 8) | 18200 | MTFMT |
| chr22-22 | chr22 | 37771220 | 37771370 | 33.5 | exon (NM_052906, exon 3 of 3) | 52210 | ELFN2 |
| chr10-131 | chr10 | 3510530 | 3510680 | 33.5 | Intergenic | -295572 | PITRM1 |
| chr14-82 | chr14 | 61448788 | 61448938 | 33.5 | intron (NR_033344, intron 1 of 16) | 898 | SLC38A6 |
| chr20-85 | chr20 | 56982607 | 56982757 | 33.5 | intron (NR_036633, intron 1 of 3) | 18507 | VAPB |
| chr21-23 | chr21 | 30547249 | 30547399 | 33.5 | 3' UTR (NM_020152, exon 9 of 9) | -18491 | LINC00189 |
| chr8-89 | chr8 | 72503500 | 72503650 | 33.5 | Intergenic | -229108 | EYA1 |
| chr2-258 | chr2 | 241535838 | 241535988 | 33.5 | exon (NM_023083, exon 8 of 12) | -8912 | GPR35 |
| chr2-234 | chr2 | 134192956 | 134193106 | 33.5 | intron (NM_207363, intron 3 of 19) | 133000 | NCKAP5 |
| chr15-102 | chr15 | 52629541 | 52629691 | 33.5 | intron (NM_000259, intron 32 of 40) | -41621 | MYO5C |
| chr2-255 | chr2 | 235103059 | 235103209 | 33.5 | Intergenic | 143788 | SPP2 |
| chr21-28 | chr21 | 24762562 | 24762712 | 33.5 | Intergenic | -5481 | D21S2088E |
| chr8-116 | chr8 | 72743619 | 72743769 | 33.5 | Intergenic | -11664 | LOC100132891 |
| chr17-165 | chr17 | 55613318 | 55613468 | 33.5 | intron (NM_170721, intron 6 of 9) | 279019 | MSI2 |
| chr11-148 | chr11 | 86473057 | 86473207 | 33.5 | Intergenic | -38359 | PRSS23 |
| chr7-134 | chr7 | 75677257 | 75677407 | 33.5 | promoter-TSS (NM_016086) | -11 | STYXL1 |
| chr5-103 | chr5 | 34787269 | 34787419 | 33.5 | intron (NM_015577, intron 3 of 17) | -51925 | TTC23L |
| chr1-198 | chr1 | 46806487 | 46806637 | 33.5 | promoter-TSS (NM_001256128) | -288 | NSUN4 |
| chr17-163 | chr17 | 46975390 | 46975540 | 33.5 | Intergenic | 5317 | ATP5G1 |
| chr3-139 | chr3 | 127779254 | 127779404 | 33.5 | intron (NM_013336, intron 6 of 11) | 8117 | SEC61A1 |
| chr15-76 | chr15 | 44086039 | 44086189 | 33.5 | promoter-TSS (NR_031695) | -157 | MIR1282 |
| chr2-209 | chr2 | 62729066 | 62729216 | 33.5 | intron (NM_198276, intron 3 of 3) | 4463 | TMEM17 |
| chr16-90 | chr16 | 12737694 | 12737844 | 33.5 | Intergenic | -76409 | MIR4718 |
| chr1-145 | chr1 | 45671700 | 45671850 | 33.5 | exon (NM_020883, exon 1 of 14) | 475 | ZSWIM5 |
| chr3-111 | chr3 | 14964360 | 14964510 | 33.5 | intron (NM_152536, intron 15 of 19) | 24577 | FGD5-AS1 |
| chr18-53 | chr18 | 32559108 | 32559258 | 33.5 | intron (NM_001143827, intron 1 of 7) | 975 | MAPRE2 |
| chr16-88 | chr16 | 1652269 | 1652419 | 33.5 | intron (NM_014714, intron 4 of 30) | 9765 | IFT140 |
| chr12-165 | chr12 | 113726352 | 113726502 | 33.5 | intron (NM_001143819, intron 21 of 28) | 46498 | SLC24A6 |
| chr1-218 | chr1 | 152883189 | 152883339 | 33.5 | exon (NM_005547, exon 2 of 2) | 2225 | IVL |
| chr20-63 | chr20 | 1550180 | 1550330 | 33.5 | intron (NM_001083910, intron 2 of 3) | -11912 | SIRPD |
| chr2-244 | chr2 | 212008918 | 212009068 | 33.5 | Intergenic | 526698 | CPS1-IT1 |
| chr14-73 | chr14 | 34420817 | 34420967 | 33.5 | promoter-TSS (NM_022073) | -608 | EGLN3 |
| chr1-202 | chr1 | 78148364 | 78148514 | 33.5 | promoter-TSS (NM_015534) | -96 | ZZZ3 |
| chr7-96 | chr7 | 75701534 | 75701684 | 33.5 | Intergenic | 24216 | MDH2 |
| chr8-124 | chr8 | 107023678 | 107023828 | 33.5 | Intergenic | -258653 | OXR1 |
| chr17-105 | chr17 | 18023187 | 18023337 | 33.5 | exon (NM_016239, exon 2 of 65) | 11242 | MYO15A |
| chr3-124 | chr3 | 57969740 | 57969890 | 33.5 | Intergenic | -24312 | FLNB |
| chr6-101 | chr6 | 44355174 | 44355324 | 33.5 | promoter-TSS (NM_001253) | -2 | CDC5L |
| chr7-116 | chr7 | 4672751 | 4672901 | 33.5 | Intergenic | -49104 | FOXK1 |
| chr20-80 | chr20 | 44566157 | 44566307 | 33.5 | intron (NM_022104, intron 2 of 16) | 2915 | PCIF1 |
| chr12-148 | chr12 | 81172048 | 81172198 | 33.5 | Intergenic | 54285 | MIR617 |
| chr19-126 | chr19 | 1099581 | 1099731 | 33.5 | Intergenic | -4265 | POLR2E |
| chr12-152 | chr12 | 95576704 | 95576854 | 33.5 | intron (NM_018351, intron 2 of 20) | 34461 | FGD6 |
| chr21-26 | chr21 | 17121803 | 17121953 | 33.5 | intron (NM_013396, intron 1 of 23) | 19382 | USP25 |
| chr10-190 | chr10 | 81081027 | 81081177 | 33.5 | Intergenic | -26118 | PPIF |
| chr16-111 | chr16 | 67275296 | 67275446 | 33.5 | intron (NM_013241, intron 1 of 21) | 6054 | FHOD1 |
| chr9-130 | chr9 | 137484412 | 137484562 | 33.5 | Intergenic | -49165 | COL5A1 |
| chr4-56 | chr4 | 28178648 | 28178798 | 33.5 | Intergenic | -642481 | MIR4275 |
| chr15-60 | chr15 | 44101938 | 44102088 | 33.5 | exon (NM_005926, exon 7 of 9) | 9394 | HYPK |
| chrX-58 | chrX | 77192842 | 77192992 | 33.5 | intron (NM_000052, intron 1 of 22) | 26723 | ATP7A |
| chr17-162 | chr17 | 46080064 | 46080214 | 33.5 | Intergenic | 31710 | CDK5RAP3 |
| chr5-100 | chr5 | 32387658 | 32387808 | 33.5 | exon (NM_016107, exon 14 of 20) | 57111 | ZFR |
| chr2-213 | chr2 | 72500719 | 72500869 | 33.5 | intron (NM_015189, intron 20 of 21) | -125831 | CYP26B1 |
| chr6-102 | chr6 | 44393840 | 44393990 | 33.5 | exon (NM_001253, exon 12 of 16) | -9463 | MIR4642 |
| chr3-94 | chr3 | 100784920 | 100785070 | 33.5 | Intergenic | -72661 | ABI3BP |
| chr14-57 | chr14 | 39846082 | 39846232 | 33.5 | Intergenic | 55547 | FBXO33 |
| chr6-117 | chr6 | 146134510 | 146134660 | 33.5 | intron (NM_032145, intron 1 of 2) | 1336 | FBXO30 |
| chr9-93 | chr9 | 96229363 | 96229513 | 33.5 | intron (NM_014612, intron 1 of 17) | -13564 | FAM120AOS |
| chr1-164 | chr1 | 159849899 | 159850049 | 33.5 | intron (NM_012337, intron 8 of 11) | -17527 | VSIG8 |
| chr20-79 | chr20 | 42839435 | 42839585 | 33.5 | promoter-TSS (NM_016470) | 36 | C20orf111 |
| chr1-212 | chr1 | 150048285 | 150048435 | 33.5 | exon (NM_007259, exon 4 of 15) | 9018 | VPS45 |
| chr4-85 | chr4 | 33495892 | 33496042 | 33.5 | Intergenic | 2750012 | ARAP2 |
| chr1-183 | chr1 | 11346361 | 11346511 | 33.5 | 3' UTR (NM_013319, exon 2 of 2) | 13181 | UBIAD1 |
| chr11-125 | chr11 | 34265359 | 34265509 | 33.5 | intron (NM_145804, intron 1 of 16) | 114121 | ABTB2 |
| chr6-112 | chr6 | 106049452 | 106049602 | 33.5 | Intergenic | -198528 | PREP |
| chr8-117 | chr8 | 74253391 | 74253541 | 33.5 | Intergenic | 46629 | RDH10 |
| chr17-151 | chr17 | 36499307 | 36499457 | 33.5 | exon (NM_001004334, exon 1 of 11) | 311 | GPR179 |
| chr20-73 | chr20 | 24015617 | 24015767 | 33.5 | Intergenic | -46276 | GGTLC1 |
| chr13-26 | chr13 | 35441807 | 35441957 | 33.5 | Intergenic | -74542 | NBEA |
| chr15-118 | chr15 | 77097567 | 77097717 | 33.5 | intron (NM_020843, intron 4 of 30) | 56643 | SCAPER |
| chr4-101 | chr4 | 87014028 | 87014178 | 33.5 | intron (NM_138981, intron 4 of 8) | 14703 | MAPK10 |
| chr4-73 | chr4 | 187075620 | 187075770 | 33.5 | exon (NM_001006655, exon 5 of 13) | 5368 | FAM149A |
| chr3-150 | chr3 | 177077744 | 177077894 | 33.5 | Intergenic | -81890 | LINC00578 |
| chr7-150 | chr7 | 152841382 | 152841532 | 33.5 | Intergenic | 384623 | ACTR3B |
| chr12-172 | chr12 | 133263874 | 133264024 | 33.5 | promoter-TSS (NM_018663) | -4 | POLE |
| chrX-50 | chrX | 24828158 | 24828308 | 33.5 | intron (NM_016937, intron 27 of 36) | 65675 | SCARNA23 |
| chr8-102 | chr8 | 13061293 | 13061443 | 33.5 | intron (NM_182643, intron 5 of 17) | -70559 | DLC1 |
| chr6-79 | chr6 | 41934815 | 41934965 | 33.5 | intron (NM_001136017, intron 1 of 4) | -25338 | CCND3 |
| chr5-137 | chr5 | 152936645 | 152936795 | 33.5 | intron (NM_001258020, intron 3 of 16) | 64988 | GRIA1 |
| chr8-90 | chr8 | 90909548 | 90909698 | 33.5 | Intergenic | -4473 | OSGIN2 |
| chr1-207 | chr1 | 111490524 | 111490674 | 33.5 | exon (NM_018372, exon 4 of 4) | 15967 | LRIF1 |
| chrX-46 | chrX | 9320754 | 9320904 | 33.5 | Intergenic | -110506 | TBL1X |
| chr3-162 | chr3 | 197445887 | 197446037 | 33.5 | intron (NM_001145642, intron 1 of 20) | 17811 | KIAA0226 |
| chr7-141 | chr7 | 108291072 | 108291222 | 33.5 | Intergenic | 80958 | DNAJB9 |
| chr11-136 | chr11 | 65260126 | 65260276 | 33.5 | Intergenic | -5032 | MALAT1 |
| chr9-81 | chr9 | 12812790 | 12812940 | 33.5 | intron (NM_203403, intron 1 of 1) | 37853 | LURAP1L |
| chr3-123 | chr3 | 56972656 | 56972806 | 33.5 | intron (NM_001128615, intron 3 of 12) | -1337 | ARHGEF3-AS1 |
| chr15-129 | chr15 | 99417170 | 99417320 | 33.5 | intron (NM_000875, intron 2 of 20) | 89590 | MIR4714 |
| chr4-111 | chr4 | 154478012 | 154478162 | 33.5 | intron (NM_001131007, intron 5 of 34) | 90589 | KIAA0922 |
| chr3-157 | chr3 | 193807455 | 193807605 | 33.5 | Intergenic | -46401 | HES1 |
| chr15-122 | chr15 | 84433329 | 84433479 | 33.5 | intron (NM_207517, intron 3 of 29) | 110566 | ADAMTSL3 |
| chr10-183 | chr10 | 64529443 | 64529593 | 33.5 | Intergenic | -34998 | ADO |
| chr12-154 | chr12 | 98029509 | 98029659 | 33.5 | Intergenic | 71994 | MIR135A2 |
| chr11-135 | chr11 | 61404216 | 61404366 | 33.5 | non-coding (NR_002775, exon 5 of 5) | 21783 | RPLP0P2 |
| chr2-170 | chr2 | 113551880 | 113552030 | 33.5 | Intergenic | -8984 | IL1A |
| chr21-22 | chr21 | 18864527 | 18864677 | 33.5 | Intergenic | -20622 | CXADR |
| chr2-204 | chr2 | 47420415 | 47420565 | 33.5 | Intergenic | -16750 | CALM2 |
| chr2-189 | chr2 | 240202072 | 240202222 | 33.5 | intron (NM_006037, intron 2 of 26) | -25010 | MIR4269 |
| chr1-224 | chr1 | 168362231 | 168362381 | 33.5 | Intergenic | 17544 | MIR557 |
| chr10-175 | chr10 | 33666310 | 33666460 | 33.5 | Intergenic | -42552 | NRP1 |
| chr11-142 | chr11 | 72145637 | 72145787 | 33.5 | promoter-TSS (NM_001258392) | 12 | CLPB |
| chr14-78 | chr14 | 53745928 | 53746078 | 33.5 | Intergenic | -125957 | DDHD1 |
| chr12-100 | chr12 | 754789 | 754939 | 33.5 | intron (NM_016533, intron 1 of 3) | 17891 | NINJ2 |
| chr13-38 | chr13 | 73664661 | 73664811 | 33.5 | Intergenic | 31594 | KLF5 |
| chr2-154 | chr2 | 27944240 | 27944390 | 33.5 | Intergenic | -50269 | MRPL33 |
| chr17-129 | chr17 | 75230035 | 75230185 | 33.5 | Intergenic | -47382 | 9-Sep |
| chr15-97 | chr15 | 35219178 | 35219328 | 33.5 | exon (NM_014691, exon 13 of 35) | 42742 | AQR |
| chr10-194 | chr10 | 112424855 | 112425005 | 33.5 | intron (NM_001134363, intron 1 of 13) | 20775 | RBM20 |
| chr18-49 | chr18 | 68004519 | 68004669 | 33.5 | Intergenic | 48457 | SOCS6 |
| chr5-77 | chr5 | 37668912 | 37669062 | 33.5 | intron (NM_018034, intron 10 of 17) | 166606 | GDNF |
| chr4-88 | chr4 | 41259341 | 41259491 | 33.5 | intron (NM_004181, intron 2 of 8) | 518 | UCHL1 |
| chr15-90 | chr15 | 79165506 | 79165656 | 33.5 | intron (NM_001265604, intron 1 of 11) | 250 | MORF4L1 |
| chr8-130 | chr8 | 125642132 | 125642282 | 33.5 | intron (NM_014751, intron 3 of 13) | 90864 | NDUFB9 |
| chr3-144 | chr3 | 148661373 | 148661523 | 33.5 | Intergenic | -47747 | GYG1 |
| chr11-111 | chr11 | 77313494 | 77313644 | 33.5 | intron (NM_173039, intron 1 of 2) | 12889 | AQP11 |
| chr5-106 | chr5 | 54736392 | 54736542 | 33.5 | intron (NM_176895, intron 4 of 5) | 68287 | MIR5687 |
| chr7-93 | chr7 | 47826212 | 47826362 | 33.5 | intron (NM_138295, intron 56 of 56) | -8602 | C7orf69 |
| chr9-85 | chr9 | 35071916 | 35072066 | 33.5 | intron (NM_007126, intron 1 of 16) | 748 | VCP |
| chr1-201 | chr1 | 63009431 | 63009581 | 33.5 | intron (NM_033407, intron 22 of 48) | -53681 | ANGPTL3 |
| chr12-122 | chr12 | 104020272 | 104020422 | 33.5 | intron (NM_017564, intron 5 of 68) | 39278 | STAB2 |
| chr6-93 | chr6 | 143730508 | 143730658 | 33.5 | Intergenic | 41258 | ADAT2 |
| chr15-73 | chr15 | 33159621 | 33159771 | 33.5 | intron (NM_001103184, intron 12 of 16) | 149491 | GREM1 |
| chr2-203 | chr2 | 45844351 | 45844501 | 33.5 | Intergenic | -5993 | SRBD1 |
| chr12-119 | chr12 | 96894945 | 96895095 | 33.5 | Intergenic | -100654 | CDK17 |
| chr2-155 | chr2 | 28807193 | 28807343 | 33.5 | intron (NM_153021, intron 25 of 57) | 88330 | PLB1 |
| chr9-107 | chr9 | 21071122 | 21071272 | 33.5 | Intergenic | 6746 | IFNB1 |
| chr12-130 | chr12 | 4606414 | 4606564 | 33.5 | intron (NM_020374, intron 11 of 13) | 41148 | C12orf4 |
| chr8-81 | chr8 | 24014000 | 24014150 | 33.5 | Intergenic | -137505 | ADAM28 |
| chr8-131 | chr8 | 128680727 | 128680877 | 33.5 | Intergenic | -67513 | MYC |
| chr20-86 | chr20 | 61863725 | 61863875 | 33.5 | Intergenic | -3476 | BIRC7 |
| chr9-109 | chr9 | 34158457 | 34158607 | 33.5 | Intergenic | -20471 | UBAP1 |
| chr3-140 | chr3 | 134047102 | 134047252 | 33.5 | Intergenic | 46229 | AMOTL2 |
| chr8-138 | chr8 | 141819690 | 141819840 | 33.5 | intron (NM_005607, intron 10 of 31) | -174119 | EIF2C2 |
| chr22-38 | chr22 | 45945176 | 45945326 | 33.5 | intron (NM_006485, intron 13 of 14) | 46532 | FBLN1 |
| chr5-109 | chr5 | 55853177 | 55853327 | 33.5 | Intergenic | -257648 | MAP3K1 |
| chr19-123 | chr19 | 52089705 | 52089855 | 33.5 | intron (NM_007147, intron 4 of 4) | 7853 | FLJ30403 |
| chr1-169 | chr1 | 172325849 | 172325999 | 33.5 | intron (NM_015569, intron 17 of 20) | -63904 | C1orf105 |
| chr16-117 | chr16 | 80061092 | 80061242 | 33.5 | Intergenic | -426545 | MAF |
| chr14-83 | chr14 | 65078875 | 65079025 | 33.5 | Intergenic | 62330 | PPP1R36 |
| chr10-187 | chr10 | 70782410 | 70782560 | 33.5 | Intergenic | 34008 | KIAA1279 |
| chr11-110 | chr11 | 72983155 | 72983305 | 33.5 | promoter-TSS (NM_004154) | -17 | P2RY6 |
| chr1-190 | chr1 | 29386211 | 29386361 | 33.5 | intron (NM_001166005, intron 14 of 20) | 62727 | TMEM200B |
| chr7-154 | chr7 | 158465822 | 158465972 | 33.5 | intron (NM_017760, intron 12 of 27) | 31623 | NCAPG2 |
| chr5-117 | chr5 | 60705415 | 60705565 | 33.5 | intron (NM_020928, intron 1 of 13) | 77390 | ZSWIM6 |
| chr7-119 | chr7 | 17448662 | 17448812 | 33.5 | Intergenic | 110461 | AHR |
| chr11-139 | chr11 | 67351166 | 67351316 | 33.5 | 5' UTR (NM_000852, exon 1 of 7) | 175 | GSTP1 |
| chr17-138 | chr17 | 13498131 | 13498281 | 33.5 | intron (NM_006042, intron 1 of 1) | 7038 | HS3ST3A1 |
| chr13-37 | chr13 | 64009927 | 64010077 | 33.5 | Intergenic | -301566 | OR7E156P |
| chr17-137 | chr17 | 12530630 | 12530780 | 33.5 | intron (NR_034144, intron 2 of 2) | -38502 | MYOCD |
| chr3-158 | chr3 | 194210441 | 194210591 | 33.5 | Intergenic | 2647 | FLJ34208 |
| chr16-70 | chr16 | 10719943 | 10720093 | 33.5 | Intergenic | -45479 | EMP2 |
| chr4-66 | chr4 | 83273718 | 83273868 | 33.5 | TTS (NM_031369) | 21356 | HNRNPD |
| chr7-145 | chr7 | 130602635 | 130602785 | 33.5 | Intergenic | -4641 | LOC646329 |
| chr9-124 | chr9 | 130341378 | 130341528 | 33.5 | promoter-TSS (NM_001035534) | -185 | FAM129B |
| chr5-114 | chr5 | 58844751 | 58844901 | 33.5 | intron (NM_001165899, intron 3 of 16) | 37498 | PDE4D |
| chr16-107 | chr16 | 55870322 | 55870472 | 33.5 | Intergenic | -3322 | CES1 |
| chr20-64 | chr20 | 3824235 | 3824385 | 33.5 | Intergenic | -3136 | MAVS |
| chr4-84 | chr4 | 27405895 | 27406045 | 33.5 | Intergenic | 543657 | STIM2 |
| chr15-65 | chr15 | 59642477 | 59642627 | 33.5 | intron (NM_004998, intron 1 of 27) | 22519 | MYO1E |
| chr1-243 | chr1 | 244505061 | 244505211 | 33.5 | Intergenic | -10801 | C1orf100 |
| chr4-115 | chr4 | 184909368 | 184909518 | 33.5 | intron (NM_020225, intron 1 of 3) | 82934 | STOX2 |
| chr15-130 | chr15 | 100781883 | 100782033 | 33.5 | intron (NM_139057, intron 7 of 21) | 100225 | ADAMTS17 |
| chr13-33 | chr13 | 31736564 | 31736714 | 33.5 | promoter-TSS (NM_006644) | -522 | HSPH1 |
| chr8-101 | chr8 | 11339374 | 11339524 | 33.5 | Intergenic | -12072 | BLK |
| chr17-155 | chr17 | 40612126 | 40612276 | 33.5 | intron (NM_001130020, intron 1 of 20) | 1339 | ATP6V0A1 |
| chr10-147 | chr10 | 73930948 | 73931098 | 33.5 | intron (NR_045564, intron 5 of 9) | -44735 | ANAPC16 |
| chr8-122 | chr8 | 95831666 | 95831816 | 33.5 | Intergenic | -3793 | INTS8 |
| chr7-115 | chr7 | 4671429 | 4671579 | 33.5 | Intergenic | -50426 | FOXK1 |
| chr20-65 | chr20 | 4116307 | 4116457 | 33.5 | Intergenic | -13044 | SMOX |
| chr2-182 | chr2 | 174049149 | 174049299 | 33.5 | intron (NM_016653, intron 4 of 19) | 97540 | MLK7-AS1 |
| chr18-45 | chr18 | 28495456 | 28495606 | 33.5 | Intergenic | 127250 | DSC3 |
| chrX-31 | chrX | 23797290 | 23797440 | 33.5 | Intergenic | -3910 | SAT1 |
| chr17-125 | chr17 | 66756038 | 66756188 | 33.5 | Intergenic | -159018 | FAM20A |
| chr1-215 | chr1 | 151032732 | 151032882 | 33.5 | promoter-TSS (NM_020239) | 656 | MLLT11 |
| chr5-111 | chr5 | 58186948 | 58187098 | 33.5 | Intergenic | 108736 | PDE4D |
| chr3-138 | chr3 | 122288758 | 122288908 | 33.5 | exon (NM_138287, exon 3 of 5) | -5310 | PARP9 |
| chr18-54 | chr18 | 41438477 | 41438627 | 33.5 | Intergenic | -580937 | SYT4 |
| chr2-208 | chr2 | 58254306 | 58254456 | 33.5 | Intergenic | -19396 | VRK2 |
| chr3-119 | chr3 | 43914586 | 43914736 | 33.5 | Intergenic | 182286 | ABHD5 |
| chr5-99 | chr5 | 17375631 | 17375781 | 33.5 | Intergenic | 11713 | LOC401177 |
| chr3-153 | chr3 | 188302001 | 188302151 | 33.5 | intron (NM_005578, intron 5 of 10) | 358883 | LPP |
| chr16-93 | chr16 | 15744223 | 15744373 | 33.5 | intron (NM_017668, intron 1 of 8) | 215 | NDE1 |
| chr6-114 | chr6 | 123851171 | 123851321 | 33.5 | intron (NM_001256022, intron 5 of 5) | 106992 | TRDN |
| chr5-81 | chr5 | 68342282 | 68342432 | 33.5 | Intergenic | -47419 | SLC30A5 |
| chr10-165 | chr10 | 15194972 | 15195122 | 33.5 | intron (NM_004808, intron 1 of 11) | 2299 | LOC100192204 |
| chr11-146 | chr11 | 83170188 | 83170338 | 26.8 | 3' UTR (NM_001142699, exon 28 of 28) | -172886 | CCDC90B |
| chr2-235 | chr2 | 158067114 | 158067264 | 26.8 | Intergenic | -47151 | GALNT5 |
| chr15-123 | chr15 | 88424038 | 88424188 | 26.8 | intron (NM_001243101, intron 16 of 17) | 303953 | LINC00052 |
| chr16-112 | chr16 | 68330232 | 68330382 | 26.8 | exon (NM_003983, exon 9 of 11) | 14561 | SLC7A6OS |
| chr16-118 | chr16 | 81751600 | 81751750 | 26.8 | Intergenic | 52716 | LOC100129617 |
| chrX-64 | chrX | 144712958 | 144713108 | 26.8 | Intergenic | -186314 | SLITRK2 |
| chr9-104 | chr9 | 4226967 | 4227117 | 26.8 | intron (NM_001042413, intron 2 of 10) | 72993 | GLIS3 |
| chr16-97 | chr16 | 23608675 | 23608825 | 26.8 | Intergenic | -1111 | NDUFAB1 |
| chr11-124 | chr11 | 34104380 | 34104530 | 26.8 | intron (NM_203364, intron 8 of 17) | -22656 | NAT10 |
| chr10-161 | chr10 | 5190476 | 5190626 | 26.8 | Intergenic | 36599 | AKR1CL1 |
| chr15-98 | chr15 | 43249122 | 43249272 | 26.8 | intron (NM_174916, intron 44 of 46) | -36190 | TTBK2 |
| chr8-123 | chr8 | 97283641 | 97283791 | 26.8 | intron (NM_014754, intron 1 of 12) | 9549 | PTDSS1 |
| chr5-136 | chr5 | 148825580 | 148825730 | 26.8 | Intergenic | 15446 | MIR145 |
| chr11-129 | chr11 | 35326148 | 35326298 | 26.8 | intron (NM_004171, intron 5 of 10) | 114882 | SLC1A2 |
| chr15-117 | chr15 | 75315770 | 75315920 | 26.8 | promoter-TSS (NM_021823) | -82 | PPCDC |
| chr10-193 | chr10 | 94706774 | 94706924 | 26.8 | intron (NM_019053, intron 13 of 21) | 98624 | EXOC6 |
| chr1-221 | chr1 | 156252613 | 156252763 | 26.8 | promoter-TSS (NR_026678) | -16 | TMEM79 |
| chr12-146 | chr12 | 72435677 | 72435827 | 26.8 | Intergenic | 103126 | TPH2 |
| chr13-36 | chr13 | 50433655 | 50433805 | 26.8 | Intergenic | -30815 | CTAGE10P |
| chr3-107 | chr3 | 188133112 | 188133262 | 26.8 | intron (NM_005578, intron 3 of 10) | 189994 | LPP |
| chr1-236 | chr1 | 214952033 | 214952183 | 26.8 | Intergenic | 175576 | CENPF |
| chr17-154 | chr17 | 40118609 | 40118759 | 26.8 | promoter-TSS (NM_033133) | -75 | CNP |
| chr7-140 | chr7 | 107531553 | 107531703 | 26.8 | promoter-TSS (NM_000108) | 42 | DLD |
| chr19-160 | chr19 | 47593432 | 47593582 | 26.8 | intron (NM_015168, intron 4 of 14) | 23502 | ZC3H4 |
| chr9-112 | chr9 | 84746050 | 84746200 | 26.8 | Intergenic | 142438 | FAM75D1 |
| chr9-125 | chr9 | 130829143 | 130829293 | 26.8 | exon (NM_197956, exon 1 of 2) | 381 | NAIF1 |
| chr4-94 | chr4 | 57182162 | 57182312 | 26.8 | exon (NM_020722, exon 8 of 11) | 71401 | AASDH |
| chr3-129 | chr3 | 81792612 | 81792762 | 26.8 | intron (NM_000158, intron 1 of 15) | 18263 | GBE1 |
| chr4-81 | chr4 | 7807372 | 7807522 | 26.8 | intron (NM_198595, intron 9 of 16) | 51630 | AFAP1-AS1 |
| chrX-49 | chrX | 18721089 | 18721239 | 26.8 | intron (NM_152226, intron 3 of 17) | 12119 | PPEF1 |
| chr12-163 | chr12 | 109641465 | 109641615 | 26.8 | intron (NM_001093, intron 18 of 51) | 64338 | ACACB |
| chr5-128 | chr5 | 140383948 | 140384098 | 26.8 | intron (NM_018901, intron 3 of 3) | 37671 | PCDHAC2 |
| chr14-76 | chr14 | 52897280 | 52897430 | 26.8 | TTS (NM_020784) | 116339 | PTGER2 |
| chr5-104 | chr5 | 39543665 | 39543815 | 26.8 | Intergenic | -118405 | DAB2 |
| chr9-105 | chr9 | 5811235 | 5811385 | 26.8 | exon (NM_024896, exon 7 of 15) | 21771 | ERMP1 |
| chr17-169 | chr17 | 70385452 | 70385602 | 26.8 | Intergenic | 203416 | LINC00673 |
| chr6-107 | chr6 | 88498936 | 88499086 | 26.8 | Intergenic | -87026 | AKIRIN2 |
| chrX-63 | chrX | 142760999 | 142761149 | 26.8 | Intergenic | -37148 | SLITRK4 |
| chr8-105 | chr8 | 33239080 | 33239230 | 26.8 | intron (NM_032664, intron 4 of 4) | 91509 | FUT10 |
| chr19-121 | chr19 | 48857910 | 48858060 | 26.8 | intron (NM_018273, intron 3 of 7) | 9201 | TMEM143 |
| chr17-160 | chr17 | 44364393 | 44364543 | 26.8 | Intergenic | -8029 | LRRC37A |
| chr15-113 | chr15 | 70911388 | 70911538 | 26.8 | Intergenic | 83157 | UACA |
| chr3-147 | chr3 | 151614434 | 151614584 | 26.8 | Intergenic | 23078 | SUCNR1 |
| chr16-121 | chr16 | 83869710 | 83869860 | 26.8 | Intergenic | 28277 | HSBP1 |
| chr11-127 | chr11 | 35031076 | 35031226 | 26.8 | Intergenic | 67767 | MIR1343 |
| chr12-133 | chr12 | 14944160 | 14944310 | 26.8 | intron (NM_016312, intron 8 of 11) | 12166 | WBP11 |
| chr16-123 | chr16 | 87894256 | 87894406 | 26.8 | intron (NM_003486, intron 1 of 9) | 8769 | SLC7A5 |
| chr1-222 | chr1 | 165806079 | 165806229 | 26.8 | intron (NM_012474, intron 1 of 6) | 9422 | UCK2 |
| chr13-35 | chr13 | 44542754 | 44542904 | 26.8 | Intergenic | -53642 | LINC00284 |
| chr5-102 | chr5 | 34676052 | 34676202 | 26.8 | intron (NM_001145520, intron 1 of 17) | -8485 | RAI14 |
| chr1-208 | chr1 | 113952499 | 113952649 | 26.8 | intron (NM_152900, intron 1 of 20) | 19099 | MAGI3 |
| chr16-91 | chr16 | 13011681 | 13011831 | 26.8 | intron (NM_001145204, intron 2 of 4) | 16279 | SHISA9 |
| chr2-201 | chr2 | 42648081 | 42648231 | 26.8 | Intergenic | -59800 | COX7A2L |
| chr4-79 | chr4 | 5823139 | 5823289 | 26.8 | 3' UTR (NM_001014809, exon 14 of 14) | 67101 | CRMP1 |
| chr14-88 | chr14 | 93763213 | 93763363 | 26.8 | intron (NM_001002860, intron 1 of 10) | 36097 | BTBD7 |
| chr11-149 | chr11 | 100660348 | 100660498 | 26.8 | intron (NM_152432, intron 2 of 23) | 102016 | ARHGAP42 |
| chr11-150 | chr11 | 110182709 | 110182859 | 26.8 | Intergenic | -15347 | RDX |
| chr2-225 | chr2 | 98487143 | 98487293 | 26.8 | intron (NM_015348, intron 4 of 40) | 125136 | TMEM131 |
| chr15-127 | chr15 | 95790304 | 95790454 | 26.8 | Intergenic | 79950 | LOC400456 |
| chr4-107 | chr4 | 117520027 | 117520177 | 26.8 | Intergenic | 299221 | MIR1973 |
| chr19-139 | chr19 | 32201591 | 32201741 | 26.8 | Intergenic | 118479 | THEG5 |
| chr17-174 | chr17 | 76677122 | 76677272 | 26.8 | intron (NM_004762, intron 10 of 12) | 101179 | CYTH1 |
| chr2-251 | chr2 | 225133157 | 225133307 | 26.8 | Intergenic | 133479 | FAM124B |
| chr4-96 | chr4 | 78267018 | 78267168 | 26.8 | Intergenic | -165814 | CXCL13 |
| chr2-205 | chr2 | 47750831 | 47750981 | 26.8 | intron (NM_022055, intron 1 of 1) | 46564 | KCNK12 |
| chr4-89 | chr4 | 43589833 | 43589983 | 26.8 | Intergenic | 694625 | GRXCR1 |
| chr3-128 | chr3 | 81792232 | 81792382 | 26.8 | intron (NM_000158, intron 1 of 15) | 18643 | GBE1 |
| chr3-115 | chr3 | 31330049 | 31330199 | 26.8 | Intergenic | -244367 | STT3B |
| chr7-122 | chr7 | 28862463 | 28862613 | 26.8 | 3' UTR (NM_001011666, exon 7 of 7) | 135491 | TRIL |
| chr11-137 | chr11 | 65880724 | 65880874 | 26.8 | intron (NM_018026, intron 1 of 23) | 42975 | PACS1 |
| chr5-107 | chr5 | 55299007 | 55299157 | 26.8 | Intergenic | -8261 | IL6ST |
| chr7-129 | chr7 | 47970872 | 47971022 | 26.8 | intron (NM_138295, intron 5 of 56) | 17124 | PKD1L1 |
| chr15-109 | chr15 | 66572433 | 66572583 | 26.8 | Intergenic | -13125 | DIS3L |
| chr14-84 | chr14 | 68745381 | 68745531 | 26.8 | intron (NM_133510, intron 7 of 10) | 458960 | RAD51B |
| chr8-112 | chr8 | 59017286 | 59017436 | 26.8 | intron (NM_147189, intron 4 of 4) | 110248 | FAM110B |
| chr7-146 | chr7 | 130916550 | 130916700 | 26.8 | intron (NM_001145354, intron 2 of 18) | -95970 | MKLN1 |
| chr19-141 | chr19 | 33610009 | 33610159 | 26.8 | intron (NM_018025, intron 17 of 19) | -12914 | WDR88 |
| chr16-108 | chr16 | 57238497 | 57238647 | 26.8 | exon (NM_133368, exon 2 of 15) | 18331 | RSPRY1 |
| chr2-202 | chr2 | 45186222 | 45186372 | 26.8 | Intergenic | 17260 | SIX3 |
| chr19-158 | chr19 | 46313084 | 46313234 | 26.8 | intron (NM_030785, intron 2 of 5) | 5446 | RSPH6A |
| chr1-244 | chr1 | 244559668 | 244559818 | 26.8 | Intergenic | 43806 | C1orf100 |
| chr4-77 | chr4 | 5038142 | 5038292 | 26.8 | Intergenic | -15310 | STK32B |
| chr1-197 | chr1 | 45732135 | 45732285 | 26.8 | Intergenic | -37372 | LOC400752 |
| chr14-87 | chr14 | 91066142 | 91066292 | 26.8 | intron (NM_001010854, intron 17 of 19) | 144643 | LINC00642 |
| chr1-227 | chr1 | 179274669 | 179274819 | 26.8 | intron (NM_001252511, intron 2 of 14) | 11895 | SOAT1 |
| chr1-220 | chr1 | 155533005 | 155533155 | 26.8 | promoter-TSS (NM_018489) | -756 | ASH1L |
| chr10-167 | chr10 | 21575015 | 21575165 | 26.8 | Intergenic | 111859 | LOC100128511 |
| chr6-113 | chr6 | 116376795 | 116376945 | 26.8 | intron (NM_002031, intron 1 of 7) | 5051 | FRK |
| chr3-154 | chr3 | 189072018 | 189072168 | 26.8 | Intergenic | -113710 | TPRG1-AS2 |
| chr5-113 | chr5 | 58351510 | 58351660 | 26.8 | intron (NM_001104631, intron 5 of 14) | -16246 | PDE4D |
| chr12-157 | chr12 | 105352267 | 105352417 | 26.8 | Intergenic | -27756 | C12orf45 |
| chr10-195 | chr10 | 115613444 | 115613594 | 26.8 | promoter-TSS (NM_198514) | 340 | DCLRE1A |
| chr5-101 | chr5 | 33850317 | 33850467 | 26.8 | intron (NM_030955, intron 2 of 23) | 41732 | ADAMTS12 |
| chr9-103 | chr9 | 3457271 | 3457421 | 26.8 | intron (NM_002919, intron 2 of 15) | 68637 | RFX3 |
| chr2-236 | chr2 | 158336582 | 158336732 | 26.8 | Intergenic | -36053 | CYTIP |
| chr9-115 | chr9 | 98637789 | 98637939 | 26.8 | promoter-TSS (NM_001010895) | -36 | ERCC6L2 |
| chr20-69 | chr20 | 17949453 | 17949603 | 26.8 | promoter-TSS (NM_014426) | -38 | SNX5 |
| chr2-243 | chr2 | 211749350 | 211749500 | 26.8 | Intergenic | 267130 | CPS1-IT1 |
| chr7-133 | chr7 | 73120800 | 73120950 | 26.8 | intron (NM_004603, intron 3 of 9) | -4772 | MIR4284 |
| chr19-166 | chr19 | 57874696 | 57874846 | 26.8 | promoter-TSS (NR_002166) | -108 | TRAPPC2P1 |
| chr17-173 | chr17 | 75407249 | 75407399 | 26.8 | intron (NM_001113494, intron 2 of 10) | -14188 | MIR4316 |
| chr5-132 | chr5 | 146086069 | 146086219 | 26.8 | intron (NM_181676, intron 1 of 8) | 172015 | PPP2R2B |
| chr16-94 | chr16 | 19316280 | 19316430 | 26.8 | intron (NM_001256720, intron 3 of 4) | 19250 | CLEC19A |
| chr7-124 | chr7 | 36247355 | 36247505 | 26.8 | intron (NM_030636, intron 2 of 7) | 54594 | EEPD1 |
| chr17-167 | chr17 | 67009532 | 67009682 | 26.8 | intron (NM_080283, intron 22 of 38) | 47529 | ABCA9 |
| chr6-96 | chr6 | 13395652 | 13395802 | 26.8 | intron (NM_018988, intron 1 of 1) | 12642 | GFOD1 |
| chr17-175 | chr17 | 78121030 | 78121180 | 26.8 | promoter-TSS (NM_014740) | -123 | EIF4A3 |
| chr14-71 | chr14 | 20687323 | 20687473 | 26.8 | Intergenic | -4471 | OR11H6 |
| chr5-110 | chr5 | 57702982 | 57703132 | 26.8 | Intergenic | 52909 | PLK2 |
| chr2-199 | chr2 | 33009640 | 33009790 | 26.8 | intron (NM_001193509, intron 15 of 19) | -40795 | LINC00486 |
| chr5-126 | chr5 | 121239140 | 121239290 | 26.8 | Intergenic | 51565 | FTMT |
| chr17-133 | chr17 | 5436040 | 5436190 | 26.8 | intron (NM_033007, intron 10 of 14) | -31796 | LOC728392 |
| chr15-114 | chr15 | 71737663 | 71737813 | 26.8 | intron (NM_024817, intron 6 of 16) | 303950 | THSD4 |
| chr6-106 | chr6 | 54358036 | 54358186 | 26.8 | Intergenic | 184908 | TINAG |
| chr12-136 | chr12 | 32443431 | 32443581 | 26.8 | intron (NM_001003398, intron 2 of 8) | 183321 | BICD1 |
| chr2-248 | chr2 | 223891554 | 223891704 | 26.8 | Intergenic | -25233 | KCNE4 |
| chr1-233 | chr1 | 203923677 | 203923827 | 26.8 | Intergenic | 86640 | LINC00303 |
| chr19-131 | chr19 | 13280675 | 13280825 | 26.8 | Intergenic | 19468 | IER2 |
| chr1-188 | chr1 | 24106642 | 24106792 | 26.8 | intron (NM_020362, intron 3 of 5) | 1841 | PITHD1 |
| chr4-106 | chr4 | 114758676 | 114758826 | 26.8 | Intergenic | -75668 | CAMK2D |
| chr15-101 | chr15 | 52501151 | 52501301 | 26.8 | intron (NM_018728, intron 35 of 40) | -17661 | GNB5 |
| chr17-179 | chr17 | 80399994 | 80400144 | 26.8 | TTS (NR_036516) | 7533 | C17orf62 |
| chr4-97 | chr4 | 79567259 | 79567409 | 26.8 | non-coding (NR_038305, exon 1 of 4) | 186 | LOC100505702 |
| chr3-149 | chr3 | 156530375 | 156530525 | 26.8 | promoter-TSS (NR_003284) | -640 | PA2G4P4 |
| chr10-168 | chr10 | 23342169 | 23342319 | 26.8 | Intergenic | -42183 | MSRB2 |
| chr2-252 | chr2 | 228253443 | 228253593 | 26.8 | Intergenic | -9496 | TM4SF20 |
| chr15-108 | chr15 | 66088982 | 66089132 | 26.8 | Intergenic | -4426 | DENND4A |
| chr9-111 | chr9 | 84144894 | 84145044 | 26.8 | Intergenic | 158627 | TLE1 |
| chr12-161 | chr12 | 109116901 | 109117051 | 26.8 | intron (NM_014325, intron 1 of 10) | 8319 | CORO1C |
| chr15-121 | chr15 | 81322003 | 81322153 | 26.8 | Intergenic | 28783 | MESDC1 |
| chr14-91 | chr14 | 105398121 | 105398271 | 26.8 | exon (NM_138790, exon 8 of 11) | 7009 | PLD4 |
| chr2-245 | chr2 | 218882990 | 218883140 | 26.8 | Intergenic | -16592 | RUFY4 |
| chr12-128 | chr12 | 1609432 | 1609582 | 26.8 | promoter-TSS (NR_028415) | -150 | LOC100292680 |
| chr17-130 | chr17 | 597624 | 597774 | 26.8 | intron (NM_001128159, intron 4 of 21) | 20397 | VPS53 |
| chr11-121 | chr11 | 13484766 | 13484916 | 26.8 | promoter-TSS (NM_032320) | -3 | BTBD10 |
| chr6-109 | chr6 | 89745286 | 89745436 | 26.8 | Intergenic | -45068 | PNRC1 |
| chr10-171 | chr10 | 29008726 | 29008876 | 26.8 | Intergenic | 42377 | BAMBI |
| chr2-196 | chr2 | 28990313 | 28990463 | 26.8 | intron (NM_002709, intron 1 of 7) | 15762 | PPP1CB |
| chr3-133 | chr3 | 102517652 | 102517802 | 26.8 | Intergenic | 363868 | ZPLD1 |
| chr21-27 | chr21 | 20287949 | 20288099 | 26.8 | Intergenic | -512054 | TMPRSS15 |
|  |  |  |  |  |  |  |  |
