# Supplementary material for: Genome-wide global identification of NRF2 binding sites in A549 non-small cell lung cancer cells by ChIP-Seq reveals NRF2 regulation of genes involved in focal adhesion pathways
Source: Aging (Albany NY). 2019 Dec 28;11(24):12600–23. doi: 10.18632/aging.102590 (PMC6949066; doi:10.18632/aging.102590)
Supplement: Supplementary Table 2 [file aging-11-102590-s003..docx]

**Supplementary Table 2. Known motifs identified by HOMER motif enrichment analysis software.**

| **Motif Name** | **Consensus** | **P-value** |
| --- | --- | --- |
| Bach1(bZIP)/K562-Bach1-ChIP-Seq(GSE31477)/Homer | AWWNTGCTGAGTCAT | 1e-1063 |
| Nrf2(bZIP)/Lymphoblast-Nrf2-ChIP-Seq(GSE37589)/Homer | HTGCTGAGTCAT | 1e-1058 |
| NF-E2(bZIP)/K562-NFE2-ChIP-Seq/Homer | GATGACTCAGCA | 1e-1022 |
| Jun-AP1(bZIP)/K562-cJun-ChIP-Seq/Homer | GATGASTCATCN | 1e-621 |
| MafK(bZIP)/C2C12-MafK-ChIP-Seq(GSE36030)/Homer | GCTGASTCAGCA | 1e-540 |
| AP-1(bZIP)/ThioMac-PU.1-ChIP-Seq/Homer | VTGACTCATC | 1e-535 |
| HIF1b(HLH)/O785-HIF1b-ChIP-Seq(GSE34871)/Homer | VTGASTCABH | 1e-522 |
| MafA(bZIP)/Islet-MafA-ChIP-Seq(GSE30298)/Homer | TGCTGACTCA | 1e-327 |
| MafF(bZIP)/HepG2-MafF-ChIP-Seq(GSE31477)/Homer | HWWGTCAGCAWWTTT | 1.00E-112 |
| GFX(?)/Promoter/Homer | ATTCTCGCGAGA | 1.00E-23 |
| ZBTB33/GM12878-ZBTB33-ChIP-Seq/Homer | GGVTCTCGCGAGAAC | 1.00E-19 |
| ETS1(ETS)/Jurkat-ETS1-ChIP-Seq/Homer | ACAGGAAGTG | 1.00E-10 |
| PAX5-shortForm(Paired/Homeobox)/GM12878-PAX5-ChIP-Seq/Homer | GTCACGCTCSCTGM | 1.00E-09 |
| ETV1(ETS)/GIST48-ETV1-ChIP-Seq/Homer | AACCGGAAGT | 1.00E-08 |
| CEBP(bZIP)/CEBPb-ChIP-Seq/Homer | ATTGCGCAAC | 1.00E-08 |
| NFAT:AP1/Jurkat-NFATC1-ChIP-Seq/Homer | SARTGGAAAAWRTGAGTCAB | 1.00E-08 |
| HEB?/mES-Nanog-ChIP-Seq/Homer | CACAGCAGGGGG | 1.00E-08 |
| Nanog(Homeobox)/mES-Nanog-ChIP-Seq/Homer | RGCCATTAAC | 1.00E-08 |
| Fli1(ETS)/CD8-FLI-ChIP-Seq(GSE20898)/Homer | NRYTTCCGGH | 1.00E-08 |
| FOXA1(Forkhead)/LNCAP-FOXA1-ChIP-Seq/Homer | WAAGTAAACA | 1.00E-08 |
| GABPA(ETS)/Jurkat-GABPa-ChIP-Seq/Homer | RACCGGAAGT | 1.00E-07 |
| ERG(ETS)/VCaP-ERG-ChIP-Seq/Homer | ACAGGAAGTG | 1.00E-07 |
| ERE(NR/IR3)/MCF7-ERa-ChIP-Seq/Homer | VAGGTCACNSTGACC | 1.00E-07 |
| EWS:ERG-fusion(ETS)/CADO_ES1-EWS:ERG-ChIP-Seq/Homer | ATTTCCTGTN | 1.00E-07 |
| FOXA1(Forkhead)/MCF7-FOXA1-ChIP-Seq/Homer | WAAGTAAACA | 1.00E-06 |
| SPDEF(ETS)/VCaP-SPDEF-ChIP-Seq/Homer | ASWTCCTGBT | 1.00E-06 |
| SCL/HPC7-Scl-ChIP-Seq/Homer | AVCAGCTG | 1.00E-06 |
| ELF1(ETS)/Jurkat-ELF1-ChIP-Seq/Homer | AVCCGGAAGT | 1.00E-06 |
| Sox3(HMG)/NPC-Sox3-ChIP-Seq(GSE33059)/Homer | CCWTTGTY | 1.00E-06 |
| ETS(ETS)/Promoter/Homer | AACCGGAAGT | 1.00E-06 |
| NF1-halfsite(CTF)/LNCaP-NF1-ChIP-Seq/Homer | YTGCCAAG | 1.00E-05 |
| Elk1(ETS)/Hela-Elk1-ChIP-Seq(GSE31477)/Homer | HACTTCCGGY | 1.00E-05 |
| Elk4(ETS)/Hela-Elk4-ChIP-Seq(GSE31477)/Homer | NRYTTCCGGY | 1.00E-05 |
| Tlx?/NPC-H3K4me1-ChIP-Seq/Homer | CTGGCAGSCTGCCA | 1.00E-04 |
| EWS:FLI1-fusion(ETS)/SK_N_MC-EWS:FLI1-ChIP-Seq/Homer | VACAGGAAAT | 1.00E-04 |
| CEBP:AP1/ThioMac-CEBPb-ChIP-Seq/Homer | DRTGTTGCAA | 1.00E-04 |
| Foxo1(Forkhead)/RAW-Foxo1-ChIP-Seq/Homer | CTGTTTAC | 1.00E-04 |
| NF1(CTF)/LNCAP-NF1-ChIP-Seq/Homer | CYTGGCABNSTGCCAR | 1.00E-04 |
| TEAD4(TEA)/Tropoblast-Tead4-ChIP-Seq(GSE37350)/Homer | CCWGGAATGY | 1.00E-04 |
| TEAD(TEA)/Fibroblast-PU.1-ChIP-Seq/Homer | YCWGGAATGY | 1.00E-04 |
| NFkB-p65(RHD)/GM12787-p65-ChIP-Seq/Homer | WGGGGATTTCCC | 1.00E-04 |
| FOXP1(Forkhead)/H9-FOXP1-ChIP-Seq(GSE31006)/Homer | NYYTGTTTACHN | 1.00E-03 |
| Stat3+il23(Stat)/CD4-Stat3-ChIP-Seq/Homer | SVYTTCCNGGAARB | 1.00E-03 |
| Sox6(HMG)/Myotubes-Sox6-ChIP-Seq(GSE32627)/Homer | CCATTGTTNY | 1.00E-03 |
| RUNX1(Runt)/Jurkat-RUNX1-ChIP-Seq/Homer | AAACCACARM | 1.00E-03 |
| GATA3(Zf)/iTreg-Gata3-ChIP-Seq(GSE20898)/Homer | AGATAASR | 1.00E-03 |
| Hnf1(Homeobox)/Liver-Foxa2-Chip-Seq/Homer | GGTTAAWCATTAA | 1.00E-03 |
| NF1:FOXA1/LNCAP-FOXA1-ChIP-Seq/Homer | WNTGTTTRYTTTGGCA | 1.00E-03 |
| Atf1(bZIP)/K562-ATF1-ChIP-Seq(GSE31477)/Homer | GATGACGTCA | 1.00E-03 |
| BMYB(HTH)/Hela-BMYB-ChIPSeq(GSE27030)/Homer | NHAACBGYYV | 1.00E-03 |
| Ets1-distal(ETS)/CD4+-PolII-ChIP-Seq/Homer | MACAGGAAGT | 1.00E-03 |
| RUNX(Runt)/HPC7-Runx1-ChIP-Seq/Homer | SAAACCACAG | 1.00E-02 |
| Gata2(Zf)/K562-GATA2-ChIP-Seq/Homer | BBCTTATCTS | 1.00E-02 |
| Foxa2(Forkhead)/Liver-Foxa2-ChIP-Seq/Homer | CYTGTTTACWYW | 1.00E-02 |
| E2F4(E2F)/K562-E2F4-ChIP-Seq(GSE31477)/Homer | GGCGGGAAAH | 1.00E-02 |
| RUNX2(Runt)/PCa-RUNX2-ChIP-Seq(GSE33889)/Homer | NWAACCACADNN | 1.00E-02 |
| PAX3:FKHR-fusion(Paired/Homeobox)/Rh4-PAX3:FKHR-ChIP-Seq/Homer | ACCRTGACTAATTNN | 1.00E-02 |
| GATA-DR8(Zf)/iTreg-Gata3-ChIP-Seq(GSE20898)/Homer | AGATSTNDNNDSAGATAASN | 1.00E-02 |
| BORIS(Zf)/K562-CTCFL-ChIP-Seq/Homer | CNNBRGCGCCCCCTGSTGGC | 1.00E-02 |
| EKLF(Zf)/Erythrocyte-Klf1-ChIP-Seq(GSE20478)/Homer | NWGGGTGTGGCY | 1.00E-02 |
| Klf4(Zf)/mES-Klf4-ChIP-Seq/Homer | GCCACACCCA | 1.00E-02 |
| Bcl6(Zf)/Liver-Bcl6-ChIP-Seq(GSE31578)/Homer | NNNCTTTCCAGGAAA | 1.00E-02 |
| c-Jun-CRE(bZIP)/K562-cJun-ChIP-Seq/Homer | ATGACGTCATCY | 1.00E-02 |
| Gata4(Zf)/Heart-Gata4-ChIP-Seq(GSE35151)/Homer | NBWGATAAGR | 1.00E-02 |
| Gata1(Zf)/K562-GATA1-ChIP-Seq/Homer | SAGATAAGRV | 1.00E-02 |
| PU.1(ETS)/ThioMac-PU.1-ChIP-Seq/Homer | AGAGGAAGTG | 1.00E-02 |
| Reverb(NR/DR2)/BLRP(RAW)-Reverba-ChIP-Seq/Homer | GTRGGTCASTGGGTCA | 1.00E-02 |
| EGR(Zf)/K562-EGR1-ChIP-Seq/Homer | TCCGCCCACGCA | 1.00E-02 |
| Sox2(HMG)/mES-Sox2-ChIP-Seq/Homer | BCCATTGTTC | 1.00E-02 |
| p63(p53)/Keratinocyte-p63-ChIP-Seq/Homer | NNDRCATGYCYNRRCATGYH | 1.00E-02 |
| STAT4(Stat)/CD4-Stat4-ChIP-Seq/Homer | NYTTCCWGGAAR | 1.00E-02 |
| Rfx1(HTH)/NPC-Rfx1-ChIP-Seq/Homer | KGTTGCCATGGCAA | 1.00E-02 |
| AP2gamma(AP2)/MCF7-TFAP2c-ChIP-Seq/Homer | HHTGSCCTSAGGSCA | 1.00E-02 |
| RUNX-AML(Runt)/CD4+-PolII-ChIP-Seq/Homer | GCTGTGGTTW | 1.00E-01 |
| ETS:RUNX/Jurkat-RUNX1-ChIP-Seq/Homer | RCAGGATGTGGT | 1.00E-01 |
| CTCF(Zf)/CD4+-CTCF-ChIP-Seq/Homer | AYAGTGCCMYCTRGTGGCCA | 1.00E-01 |
| MYB(HTH)/ERMYB-Myb-ChIPSeq(GSE22095)/Homer | GGCVGTTR | 1.00E-01 |
| Sp1(Zf)/Promoter/Homer | GGCCCCGCCCCC | 1.00E-01 |
| ISRE(IRF)/ThioMac-LPS-exp/HOMER | AGTTTCASTTTC | 1.00E-01 |
| Usf2(HLH)/C2C12-Usf2-ChIP-Seq(GSE36030)/Homer | GTCACGTGGT | 1.00E-01 |
| Unknown/Homeobox/Limb-p300-ChIP-Seq/Homer | SSCMATWAAA | 1.00E-01 |
| PAX5(Paired/Homeobox)/GM12878-PAX5-ChIP-Seq/Homer | GCAGCCAAGCRTGACH | 1.00E-01 |
| JunD(bZIP)/K562-JunD-ChIP-Seq/Homer | ATGACGTCATCN | 1.00E-01 |
| Foxh1(Forkhead)/hESC-FOXH1-ChIP-Seq(GSE29422)/Homer | NNTGTGGATTSS | 1.00E-01 |
| FXR(NR/IR1)/Liver-FXR-ChIP-Seq/Homer | AGGTCANTGACCTB | 1.00E-01 |
| STAT6(Stat)/CD4-Stat6-ChIP-Seq/Homer | ABTTCYYRRGAA | 1.00E-01 |
| Hoxc9/Ainv15-Hoxc9-ChIP-Seq/Homer | GGCCATAAATCA | 1.00E-01 |
| ZNF711(Zf)/SH-SY5Y-ZNF711-ChIP-Seq/Homer | AGGCCTAG | 1.00E-01 |
| Cdx2(Homeobox)/mES-Cdx2-ChIP-Seq/Homer | GYMATAAAAH | 1.00E-01 |
| Pdx1(Homeobox)/Islet-Pdx1-ChIP-Seq/Homer | YCATYAATCA | 1.00E-01 |
| Esrrb(NR)/mES-Esrrb-ChIP-Seq/Homer | KTGACCTTGA | 1.00E-01 |
| USF1(HLH)/GM12878-Usf1-ChIP-Seq/Homer | SGTCACGTGR | 1.00E-01 |
| CRX(Homeobox)/Retina-Crx-ChIP-Seq/Homer | GCTAATCC | 1.00E-01 |
| HOXA9/HSC-Hoxa9-ChIP-Seq(GSE33509)/Homer | GGCCATAAATCA | 1.00E-01 |
| ZFX(Zf)/mES-Zfx-ChIP-Seq/Homer | AGGCCTRG | 1.00E-01 |
| PR(NR)/T47D-PR-ChIP-Seq(GSE31130)/Homer | VAGRACAKNCTGTBC | 1.00E-01 |
| YY1(Zf)/Promoter/Homer | CAAGATGGCGGC | 1.00E-01 |
| NFkB-p65-Rel(RHD)/LPS-exp/Homer | GGAAATTCCC | 1.00E-01 |
| VDR(NR/DR3)/GM10855-VDR+vitD-ChIP-Seq/Homer | ARAGGTCANWGAGTTCANNN | 1.00E-01 |
| GFY(?)/Promoter/Homer | ACTACAATTCCC | 1.00E-01 |
| CArG(MADS)/PUER-Srf-ChIP-Seq/Homer | CCATATATGGNM | 1.00E-01 |
| E-box(HLH)/Promoter/Homer | SSGGTCACGTGA | 1.00E-01 |
| ATF3(bZIP)/K562-ATF3-ChIP-Seq/Homer | SGGTCACGTGAC | 1.00E-01 |
| PU.1-IRF/Bcell-PU.1-ChIP-Seq/Homer | MGGAAGTGAAAC | 1.00E-01 |
| Stat3(Stat)/mES-Stat3-ChIP-Seq/Homer | CTTCCGGGAA | 1.00E-01 |
| STAT6/Macrophage-Stat6-ChIP-Seq/Homer | TTCCKNAGAA | 1.00E-01 |
| EBF1(EBF)/Near-E2A-ChIP-Seq/Homer | GTCCCCWGGGGA | 1.00E-01 |
| E2A(HLH)/proBcell-E2A-ChIP-Seq/Homer | DNRCAGCTGY | 1.00E-01 |
| Srebp1a(HLH)/HepG2-Srebp1a-ChIP-Seq/Homer | RTCACSCCAY | 1.00E-01 |
| AR-halfsite(NR)/LNCaP-AR-ChIP-Seq/Homer | CCAGGAACAG | 1.00E-01 |
| Nr5a2(NR)/Pancreas-LRH1-ChIP-Seq(GSE34295)/Homer | BTCAAGGTCA | 1.00E+00 |
| AP-2alpha(AP2)/Hela-AP2alpha-ChIP-Seq/Homer | ATGCCCTGAGGC | 1.00E+00 |
| Mouse_Recombination_Hotspot/Testis-DMC1-ChIP-Seq/Homer | ACTYKNATTCGTGNTACTTC | 1.00E+00 |
| Tcf12(HLH)/GM12878-Tcf12-ChIP-Seq/Homer | VCAGCTGYTG | 1.00E+00 |
| E2F(E2F)/Cell-Cycle-Exp/Homer | TTSGCGCGAAAA | 1.00E+00 |
| PRDM1/BMI1(Zf)/Hela-PRDM1-ChIP-Seq(GSE31477)/Homer | ACTTTCACTTTC | 1.00E+00 |
| E2F1(E2F)/Hela-E2F1-ChIP-Seq/Hoemr | CWGGCGGGAA | 1.00E+00 |
| Nur77(NR)/K562-NR4A1-ChIP-Seq(GSE31363)/Homer | TGACCTTTNCNT | 1.00E+00 |
| FOXA1:AR/LNCAP-AR-ChIP-Seq/Homer | AGTAAACAAAAAAGAACAND | 1.00E+00 |
| Olig2(bHLH)/Neuron-Olig2-ChIP-Seq(GSE30882)/Homer | RCCATMTGTT | 1.00E+00 |
| Rfx5(HTH)/GM12878-Rfx5-ChIP-Seq(GSE31477)/Homer | SCCTAGCAACAG | 1.00E+00 |
| Pbx3(Homeobox)/GM12878-PBX3-ChIP-Seq/Homer | SCTGTCAMTCAN | 1.00E+00 |
| X-box(HTH)/NPC-H3K4me1-ChIP-Seq/Homer | GGTTGCCATGGCAA | 1.00E+00 |
| Six1(Homeobox)/Myoblast-Six1-ChIP-Chip(GSE20150)/Homer | GKVTCADRTTWC | 1.00E+00 |
| CTCF-SatelliteElement/CD4+-CTCF-ChIP-Seq/Homer | TGCAGTTCCMVNWRTGGCCA | 1.00E+00 |
| RFX(HTH)/K562-RFX3-ChIP-Seq/Homer | CGGTTGCCATGGCAAC | 1.00E+00 |
| Hoxb4/ES-Hoxb4-ChIP-Seq(GSE34014)/Homer | TGATTRATGGCY | 1.00E+00 |
| Erra(NR)/HepG2-Erra-ChIP-Seq/Homer | CAAAGGTCAG | 1.00E+00 |
| Nr5a2(NR)/mES-Nr5a2-ChIP-Seq/Homer | BTCAAGGTCA | 1.00E+00 |
| NFAT(RHD)/Jurkat-NFATC1-ChIP-Seq/Homer | ATTTTCCATT | 1.00E+00 |
| GFY-Staf/Promoters/Homer | RACTACAATTCCCAGAAKGC | 1.00E+00 |
| TATA-Box(TBP)/Promoter/Homer | CCTTTTAWAGSC | 1.00E+00 |
| Myf5(bHLH)/GM-Myf5-ChIP-Seq(GSE24852)/Homer | BAACAGCTGT | 1.00E+00 |
| Egr2/Thymocytes-Egr2-ChIP-Seq(GSE34254)/Homer | NGCGTGGGCGGR | 1.00E+00 |
| NFkB-p50,p52(RHD)/p50-ChIP-Chip/Homer | GGGGGAATCCCC | 1.00E+00 |
| HIF2a(HLH)/O785-HIF2a-ChIP-Seq(GSE34871)/Homer | GCACGTACCC | 1.00E+00 |
| Maz(Zf)/HepG2-Maz-ChIP-Seq(GSE31477)/Homer | GGGGGGGG | 1.00E+00 |
| p53(p53)/Saos-p53-ChIP-Seq/Homer | RRCATGYCYRGRCATGYYYN | 1.00E+00 |
| EBF(EBF)/proBcell-EBF-ChIP-Seq/Homer | DGTCCCYRGGGA | 1.00E+00 |
| CRE(bZIP)/Promoter/Homer | CSGTGACGTCAC | 1.00E+00 |
| E2F6(E2F)/Hela-E2F6-ChIP-Seq(GSE31477)/Homer | GGCGGGAARN | 1.00E+00 |
| Tbox:Smad/ESCd5-Smad2_3-ChIP-Seq(GSE29422)/Homer | AGGTGHCAGACA | 1.00E+00 |
| Mef2c(MADS)/GM12878-Mef2c-ChIP-Seq(GSE32465)/Homer | DCYAAAAATAGM | 1.00E+00 |
| GATA-IR4(Zf)/iTreg-Gata3-ChIP-Seq(GSE20898)/Homer | NAGATWNBNATCTNN | 1.00E+00 |
| GATA-DR4(Zf)/iTreg-Gata3-ChIP-Seq(GSE20898)/Homer | AGATGKDGAGATAAG | 1.00E+00 |
| HRE(HSF)/HepG2-HSF1-ChIP-Seq/Homer | BSTTCTRGAABVTTCYAGAA | 1.00E+00 |
| Gfi1b(Zf)/HPC7-Gfi1b-ChIP-Seq/Homer | MAATCACTGC | 1.00E+00 |
| Tbx20(T-box)/Heart-Tbx20-ChIP-Seq(GSE29636)/Homer | GGTGYTGACAGS | 1.00E+00 |
| Pax7-long(Paired/Homeobox)/Myoblast-Pax7-ChIP-Seq(GSE25064)/Homer | TAATCHGATTAC | 1.00E+00 |
| Oct2(POU/Homeobox)/Bcell-Oct2-ChIP-Seq/Homer | ATATGCAAAT | 1.00E+00 |
| T1ISRE(IRF)/Ifnb-Exp/Homer | ACTTTCGTTTCT | 1.00E+00 |
| NRF1(NRF)/MCF7-NRF1-ChIP-Seq/Homer | CTGCGCATGCGC | 1.00E+00 |
| Oct4(POU/Homeobox)/mES-Oct4-ChIP-Seq/Homer | ATTTGCATAW | 1.00E+00 |
| MyoG(HLH)/C2C12-MyoG-ChIP-Seq(GSE36024)/Homer | AACAGCTG | 1.00E+00 |
| Srebp2(HLH)/HepG2-Srebp2-ChIP-Seq/Homer | CGGTCACSCCAC | 1.00E+00 |
| Atoh1(bHLH)/Cerebellum-Atoh1-ChIP-Seq/Homer | VNRVCAGCTGGY | 1.00E+00 |
| PBX1(Homeobox)/MCF7-PBX1-ChIP-Seq(GSE28007)/Homer | GSCTGTCACTCA | 1.00E+00 |
| GATA:SCL/Ter119-SCL-ChIP-Seq/Homer | CRGCTGBNGNSNNSAGATAA | 1.00E+00 |
| c-Myc(HLH)/LNCAP-cMyc-ChIP-Seq/Homer | VCCACGTG | 1.00E+00 |
| Pax8(Paired/Homeobox)/Rat-Pax8-ChIP-Seq/Homer | GTCATGCHTGRCTGS | 1.00E+00 |
| HIF-1a(HLH)/MCF7-HIF1a-ChIP-Seq/Homer | TACGTGCV | 1.00E+00 |
| OCT4-SOX2-TCF-NANOG((POU/Homeobox/HMG)/mES-ChIP-Seq/Homer | ATTTGCATAACAATG | 1.00E+00 |
| Mef2a(MADS)/HL1-Mef2a.biotin-ChIP-Seq/Homer/ | CYAAAAATAG | 1.00E+00 |
| Nkx3.1(Homeobox)/LNCaP-Nkx3.1-ChIP-Seq(GSE28264)/Homer | AAGCACTTAA | 1.00E+00 |
| NeuroD1(bHLH)/Islet-NeuroD1-ChIP-Seq(GSE30298)/Homer | GCCATCTGTT | 1.00E+00 |
| CHR/Cell-Cycle-Exp/Homer | SRGTTTCAAA | 1.00E+00 |
| MyoD(HLH)/Myotube-MyoD-ChIP-Seq/Homer | RRCAGCTGYTSY | 1.00E+00 |
| EBNA1(EBV virus)/Raji-EBNA1-ChIP-Seq(GSE30709)/Homer | GGYAGCAYDTGCTDCCCNNN | 1.00E+00 |
| STAT1(Stat)/HelaS3-STAT1-ChIP-Seq/Homer | NATTTCCNGGAAAT | 1.00E+00 |
| bHLHE40(HLH)/HepG2-BHLHE40-ChIP-Seq/Homer | SGKCACGTGM | 1.00E+00 |
| Tcfcp2l1(CP2)/mES-Tcfcp2l1-ChIP-Seq/Homer | NRAACCRGTTYRAACCRGYT | 1.00E+00 |
| Max(HLH)/K562-Max-ChIP-Seq/Homer | RCCACGTGGYYN | 1.00E+00 |
| PPARE(NR/DR1)/3T3L1-Pparg-ChIP-Seq/Homer | TGACCTTTGCCCCA | 1.00E+00 |
| Pax7-longest(Paired/Homeobox)/Myoblast-Pax7-ChIP-Seq(GSE25064)/Homer | NTAATTDGCYAATTANNWWD | 1.00E+00 |
| n-Myc(HLH)/mES-nMyc-ChIP-Seq/Homer | VRCCACGTGG | 1.00E+00 |
| AARE(HLH)/mES-cMyc-ChIP-Seq/Homer | GATTGCATCA | 1.00E+00 |
| STAT5(Stat)/mCD4+-Stat5a\|b-ChIP-Seq/Homer | RTTTCTNAGAAA | 1.00E+00 |
| GATA-IR3(Zf)/iTreg-Gata3-ChIP-Seq(GSE20898)/Homer | NNNNNBAGATAWYATCTVHN | 1.00E+00 |
| Lhx3(Homeobox)/Forebrain-p300-ChIP-Seq/Homer | CTAATTAGCH | 1.00E+00 |
| HNF4a(NR/DR1)/HepG2-HNF4a-ChIP-Seq/Homer | CARRGKBCAAAGTYCA | 1.00E+00 |
| p53(p53)/mES-cMyc-ChIP-Seq/Homer | ACATGCCCGGGCAT | 1.00E+00 |
| GRE/RAW264.7-GRE-ChIP-Seq/Homer | VAGRACAKWCTGTYC | 1.00E+00 |
| RXR(NR/DR1)/3T3L1-RXR-ChIP-Seq/Homer | TAGGGCAAAGGTCA | 1.00E+00 |
| TR4(NR/DR1)/Hela-TR4-ChIP-Seq/Homer | GAGGTCAAAGGTCA | 1.00E+00 |
| Smad3(MAD)/NPC-Smad3-ChIP-Seq(GSE36673)/Homer | TWGTCTGV | 1.00E+00 |
| Znf263(Zf)/K562-Znf263-ChIP-Seq/Homer | CVGTSCTCCC | 1.00E+00 |
| E2F7(E2F)/Hela-E2F7-ChIP-Seq(GSE32673)/Homer | VDTTTCCCGCCA | 1.00E+00 |
| RARg(NR)/ES-RARg-ChIP-Seq(GSE30538)/Homer | AGGTCAAGGTCA | 1.00E+00 |
| ETS:E-box/HPC7-Scl-ChIP-Seq/Homer | AGGAARCAGCTG | 1.00E+00 |
| GRE(NR/IR3)/A549-GR-ChIP-Seq/Homer | NRGVACABNVTGTYCY | 1.00E+00 |
| TCFL2(HMG)/K562-TCF7L2-ChIP-Seq(GSE29196)/Homer | ACWTCAAAGG | 1.00E+00 |
| Smad2(MAD)/ES-SMAD2-ChIP-Seq(GSE29422)/Homer | CTGTCTGG | 1.00E+00 |
| NRF1/Promoter/Homer | STGCGCATGCGC | 1.00E+00 |
| Nkx2.5(Homeobox)/HL1-Nkx2.5.biotin-ChIP-Seq/Homer | RRSCACTYAA | 1.00E+00 |
| Smad4(MAD)/ESC-SMAD4-ChIP-Seq(GSE29422)/Homer | VBSYGTCTGG | 1.00E+00 |
| NFY(CCAAT)/Promoter/Homer | RGCCAATSRG | 1.00E+00 |
| Pax7(Paired/Homeobox)/Myoblast-Pax7-ChIP-Seq(GSE25064)/Homer | TAATCAATTA | 1.00E+00 |
| E2A-nearPU.1(HLH)/Bcell-PU.1-ChIP-Seq/Homer | NVCACCTGBN | 1.00E+00 |
| c-Myc/mES-cMyc-ChIP-Seq/Homer | VVCCACGTGG | 1.00E+00 |
| PRDM9(Zf)/Testis-DMC1-ChIP-Seq(GSE35498)/Homer | ADGGYAGYAGCATCT | 1.00E+00 |
| LXRE(NR/DR4)/BLRP(RAW)-LXRb-ChIP-Seq/Homer | RGGTTACTANAGGTCA | 1.00E+00 |
| Tbx5(T-box)/HL1-Tbx5.biotin-ChIP-Seq/Homer | AGGTGTCA | 1.00E+00 |
| Tbet(T-box)/CD8-Tbet-ChIP-Seq(GSE33802)/Homer | AGGTGTGAAM | 1.00E+00 |
| GLI3(Zf)/GLI3-ChIP-Chip/Homer | CGTGGGTGGTCC | 1.00E+00 |
| IRF4(IRF)/GM12878-IRF4-ChIP-Seq/Homer | ACTGAAACCA | 1.00E+00 |
| ARE(NR)/LNCAP-AR-ChIP-Seq/Homer | RGRACASNSTGTYCYB | 1.00E+00 |
| REST-NRSF(Zf)/Jurkat-NRSF-ChIP-Seq/Homer | GGMGCTGTCCATGGTGCTGA | 1.00E+00 |
| Eomes(T-box)/H9-Eomes-ChIP-Seq/Homer | AGGTGTTAAN | 1.00E+00 |
| Tcf4(HMG)/Hct116-Tcf4-ChIP-Seq/Homer | ASATCAAAGGVA | 1.00E+00 |
| Tcf3(HMG)/mES-Tcf3-ChIP-Seq/Homer | ASWTCAAAGG | 1.00E+00 |
| ZNF143\|STAF(Zf)/CUTLL-ZNF143-ChIP-Seq(GSE29600)/Homer | ATTTCCCAGVAKSCY | 1.00E+00 |
| PRDM14(Zf)/H1-PRDM14-ChIP-Seq/Homer | RGGTCTCTAACY | 1.00E+00 |
| HOXA2(Homeobox)/mES-Hoxa2-ChIP-Seq/Homer | GYCATCMATCAT | 1.00E+00 |
| HNF6(Homeobox)/Liver-Hnf6-ChIP-Seq(ERP000394) | NTATYGATCH | 1.00E+00 |
| p53(p53)/p53-ChIP-Chip/Homer | NRGACATGTCYRGACATGTC | 1.00E+00 |
