# Supplementary material for: Genome-wide global identification of NRF2 binding sites in A549 non-small cell lung cancer cells by ChIP-Seq reveals NRF2 regulation of genes involved in focal adhesion pathways
Source: Aging (Albany NY). 2019 Dec 28;11(24):12600–23. doi: 10.18632/aging.102590 (PMC6949066; doi:10.18632/aging.102590)
Supplement: Supplementary Table 3 [file aging-11-102590-s002..docx]

**Supplementary Table 3. NRF2 binding regions in the promoter region.**

| **PeakID** | **Chr** | **Start** | **End** | **Peak Score** | **Annotation** | **Distance to TSS** | **Gene Name** |
| --- | --- | --- | --- | --- | --- | --- | --- |
| chr12-4 | chr12 | 6691699 | 6691849 | 187.4 | promoter-TSS (NR_003012) | -999 | SCARNA11 |
| chr11-90 | chr11 | 95658282 | 95658432 | 46.8 | promoter-TSS (NM_201278) | -986 | MTMR2 |
| chr1-96 | chr1 | 182991561 | 182991711 | 53.5 | promoter-TSS (NM_002293) | -959 | LAMC1 |
| chr10-145 | chr10 | 69426291 | 69426441 | 33.5 | promoter-TSS (NM_001127384) | -950 | CTNNA3 |
| chr2-197 | chr2 | 29032681 | 29032831 | 33.5 | promoter-TSS (NM_001142634) | -944 | SPDYA |
| chr14-69 | chr14 | 104094574 | 104094724 | 40.2 | promoter-TSS (NM_182923) | -876 | KLC1 |
| chr17-43 | chr17 | 1617996 | 1618146 | 60.2 | promoter-TSS (NR_029494) | -790 | MIR22 |
| chr17-81 | chr17 | 75283136 | 75283286 | 46.8 | promoter-TSS (NM_001113492) | -762 | 9-Sep |
| chr1-220 | chr1 | 155533005 | 155533155 | 26.8 | promoter-TSS (NM_018489) | -756 | ASH1L |
| chr16-5 | chr16 | 33963181 | 33963331 | 133.9 | promoter-TSS (NR_038368) | -753 | LINC00273 |
| chr6-85 | chr6 | 97346444 | 97346594 | 33.5 | promoter-TSS (NM_014165) | -752 | NDUFAF4 |
| chr3-34 | chr3 | 178978355 | 178978505 | 66.9 | promoter-TSS (NM_171829) | -751 | KCNMB3 |
| chr6-27 | chr6 | 32937929 | 32938079 | 60.2 | promoter-TSS (NM_001113182).3 | -661 | BRD2 |
| chr17-90 | chr17 | 33416926 | 33417076 | 40.2 | promoter-TSS (NR_037713) | -653 | RFFL |
| chr5-48 | chr5 | 58653303 | 58653453 | 60.2 | promoter-TSS (NM_001197219) | -651 | PDE4D |
| chr3-149 | chr3 | 156530375 | 156530525 | 26.8 | promoter-TSS (NR_003284) | -640 | PA2G4P4 |
| chr4-43 | chr4 | 88896098 | 88896248 | 46.8 | promoter-TSS (NM_000582) | -629 | SPP1 |
| chr14-73 | chr14 | 34420817 | 34420967 | 33.5 | promoter-TSS (NM_022073) | -608 | EGLN3 |
| chrX-2 | chrX | 119005055 | 119005205 | 167.3 | promoter-TSS (NM_004541) | -604 | NDUFA1 |
| chr11-12 | chr11 | 33914361 | 33914511 | 127.2 | promoter-TSS (NM_005574) | -600 | LMO2 |
| chr1-83 | chr1 | 223888653 | 223888803 | 53.5 | promoter-TSS (NM_001146068) | -567 | CAPN2 |
| chr13-33 | chr13 | 31736564 | 31736714 | 33.5 | promoter-TSS (NM_006644) | -522 | HSPH1 |
| chr17-38 | chr17 | 33391080 | 33391230 | 73.6 | promoter-TSS (NM_001017368) | -396 | RFFL |
| chr16-1 | chr16 | 69760851 | 69761001 | 435 | promoter-TSS (NM_001025433) | -393 | NQO1 |
| chr15-103 | chr15 | 59226162 | 59226312 | 33.5 | promoter-TSS (NM_001013843) | -385 | SLTM |
| chr11-21 | chr11 | 67141948 | 67142098 | 93.7 | promoter-TSS (NM_001166212) | -375 | CLCF1 |
| chr19-55 | chr19 | 41196854 | 41197004 | 66.9 | promoter-TSS (NM_004756) | -373 | NUMBL |
| chr20-19 | chr20 | 634148 | 634298 | 53.5 | promoter-TSS (NM_080725) | -333 | SRXN1 |
| chr5-35 | chr5 | 179247436 | 179247586 | 73.6 | promoter-TSS (NM_003900) | -331 | SQSTM1 |
| chr7-69 | chr7 | 28338536 | 28338686 | 40.2 | promoter-TSS (NM_182899) | -329 | CREB5 |
| chr15-52 | chr15 | 72668764 | 72668914 | 46.8 | promoter-TSS (NM_000520) | -319 | HEXA |
| chr9-1 | chr9 | 113019158 | 113019308 | 301.2 | promoter-TSS (NM_003329) | -313 | TXN |
| chr1-198 | chr1 | 46806487 | 46806637 | 33.5 | promoter-TSS (NM_001256128) | -288 | NSUN4 |
| chr3-50 | chr3 | 133292072 | 133292222 | 60.2 | promoter-TSS (NM_017548) | -287 | CDV3 |
| chr20-4 | chr20 | 26190054 | 26190204 | 127.2 | promoter-TSS (NR_040095) | -260 | LOC284801 |
| chr18-18 | chr18 | 55254148 | 55254298 | 80.3 | promoter-TSS (NM_000140) | -254 | FECH |
| chrX-15 | chrX | 106161274 | 106161424 | 66.9 | promoter-TSS (NM_001171095) | -241 | CLDN2 |
| chr21-7 | chr21 | 9825521 | 9825671 | 194.1 | promoter-TSS (NR_037421) | -236 | MIR3648 |
| chr20-36 | chr20 | 60877717 | 60877867 | 53.5 | promoter-TSS (NM_007002) | -235 | ADRM1 |
| chr2-158 | chr2 | 55844951 | 55845101 | 40.2 | promoter-TSS (NM_001122964) | -230 | SMEK2 |
| chr12-15 | chr12 | 125399799 | 125399949 | 107.1 | promoter-TSS (NM_021009) | -219 | MIR5188 |
| chr14-64 | chr14 | 81687717 | 81687867 | 33.5 | promoter-TSS (NM_015859) | -217 | GTF2A1 |
| chr1-147 | chr1 | 52344740 | 52344890 | 40.2 | promoter-TSS (NM_001101662) | -206 | NRD1 |
| chr7-21 | chr7 | 134212073 | 134212223 | 107.1 | promoter-TSS (NM_020299) | -196 | AKR1B10 |
| chr19-101 | chr19 | 50180143 | 50180293 | 40.2 | promoter-TSS (NM_001536) | -191 | PRMT1 |
| chr16-77 | chr16 | 30076731 | 30076881 | 33.5 | promoter-TSS (NM_184041) | -188 | ALDOA |
| chr14-90 | chr14 | 105144198 | 105144348 | 33.5 | promoter-TSS (NR_039860) | -187 | MIR4710 |
| chr9-124 | chr9 | 130341378 | 130341528 | 33.5 | promoter-TSS (NM_001035534) | -185 | FAM129B |
| chr19-76 | chr19 | 15751454 | 15751604 | 53.5 | promoter-TSS (NM_000896) | -178 | CYP4F3 |
| chr11-113 | chr11 | 85376282 | 85376432 | 40.2 | promoter-TSS (NM_001039618) | -175 | CREBZF |
| chr17-58 | chr17 | 37617492 | 37617642 | 53.5 | promoter-TSS (NM_015083) | -172 | CDK12 |
| chr12-84 | chr12 | 45609526 | 45609676 | 46.8 | promoter-TSS (NM_001142679) | -169 | ANO6 |
| chr20-12 | chr20 | 26189007 | 26189157 | 100.4 | promoter-TSS (NR_030386) | -168 | MIR663A |
| chr19-37 | chr19 | 49999385 | 49999535 | 80.3 | promoter-TSS (NM_001015) | -162 | RPS11 |
| chr15-76 | chr15 | 44086039 | 44086189 | 33.5 | promoter-TSS (NR_031695) | -157 | MIR1282 |
| chr21-3 | chr21 | 9825977 | 9826127 | 247.6 | promoter-TSS (NR_037458) | -151 | MIR3687 |
| chr12-128 | chr12 | 1609432 | 1609582 | 26.8 | promoter-TSS (NR_028415) | -150 | LOC100292680 |
| chr18-23 | chr18 | 11857214 | 11857364 | 46.8 | promoter-TSS (NM_001261444) | -148 | GNAL |
| chr4-49 | chr4 | 139163557 | 139163707 | 46.8 | promoter-TSS (NM_014331) | -129 | SLC7A11 |
| chr19-64 | chr19 | 16045726 | 16045876 | 60.2 | promoter-TSS (NM_021187) | -125 | CYP4F11 |
| chr19-84 | chr19 | 49375450 | 49375600 | 46.8 | promoter-TSS (NM_014330) | -124 | PPP1R15A |
| chrX-1 | chrX | 153770261 | 153770411 | 267.7 | promoter-TSS (NM_001099856) | -123 | IKBKG |
| chr17-175 | chr17 | 78121030 | 78121180 | 26.8 | promoter-TSS (NM_014740) | -123 | EIF4A3 |
| chr17-55 | chr17 | 18625205 | 18625355 | 60.2 | promoter-TSS (NM_001037330) | -122 | TRIM16L |
| chr19-166 | chr19 | 57874696 | 57874846 | 26.8 | promoter-TSS (NR_002166) | -108 | TRAPPC2P1 |
| chr5-11 | chr5 | 140090683 | 140090833 | 167.3 | promoter-TSS (NR_026703) | -103 | VTRNA1-1 |
| chr1-30 | chr1 | 236686849 | 236686999 | 113.8 | promoter-TSS (NM_201544) | -103 | LGALS8 |
| chr1-202 | chr1 | 78148364 | 78148514 | 33.5 | promoter-TSS (NM_015534) | -96 | ZZZ3 |
| chr12-67 | chr12 | 123237201 | 123237351 | 60.2 | promoter-TSS (NM_003677) | -95 | DENR |
| chr1-143 | chr1 | 24127053 | 24127203 | 33.5 | promoter-TSS (NM_000403) | -94 | GALE |
| chr4-86 | chr4 | 38858454 | 38858604 | 33.5 | promoter-TSS (NM_006068) | -91 | TLR6 |
| chr12-104 | chr12 | 29534158 | 29534308 | 40.2 | promoter-TSS (NM_016570) | -90 | ERGIC2 |
| chr5-20 | chr5 | 140098347 | 140098497 | 113.8 | promoter-TSS (NR_026704) | -89 | VTRNA1-2 |
| chr16-45 | chr16 | 58768258 | 58768408 | 53.5 | promoter-TSS (NM_002080) | -87 | GOT2 |
| chr1-18 | chr1 | 45987619 | 45987769 | 120.5 | promoter-TSS (NM_181697) | -84 | PRDX1 |
| chr10-18 | chr10 | 15902527 | 15902677 | 133.9 | promoter-TSS (NM_024948) | -83 | FAM188A |
| chr15-117 | chr15 | 75315770 | 75315920 | 26.8 | promoter-TSS (NM_021823) | -82 | PPCDC |
| chr1-162 | chr1 | 154943229 | 154943379 | 33.5 | promoter-TSS (NM_183001) | -81 | SHC1 |
| chr19-129 | chr19 | 6530855 | 6531005 | 33.5 | promoter-TSS (NM_003811) | -80 | TNFSF9 |
| chr17-154 | chr17 | 40118609 | 40118759 | 26.8 | promoter-TSS (NM_033133) | -75 | CNP |
| chr5-127 | chr5 | 140235485 | 140235635 | 33.5 | promoter-TSS (NM_031859) | -74 | PCDHA10 |
| chr3-44 | chr3 | 185677610 | 185677760 | 66.9 | promoter-TSS (NR_033752) | -73 | LOC344887 |
| chr10-159 | chr10 | 4720259 | 4720409 | 33.5 | promoter-TSS (NR_024475) | -72 | LOC100216001 |
| chr9-95 | chr9 | 103191651 | 103191801 | 40.2 | promoter-TSS (NM_001198806) | -70 | MSANTD3 |
| chr16-54 | chr16 | 83986689 | 83986839 | 53.5 | promoter-TSS (NM_182981) | -63 | OSGIN1 |
| chr17-97 | chr17 | 57696913 | 57697063 | 46.8 | promoter-TSS (NM_004859) | -62 | CLTC |
| chr17-135 | chr17 | 8079699 | 8079849 | 33.5 | promoter-TSS (NM_183065) | -60 | TMEM107 |
| chr11-98 | chr11 | 20385554 | 20385704 | 40.2 | promoter-TSS (NM_001098523) | -58 | HTATIP2 |
| chr3-29 | chr3 | 114343770 | 114343920 | 73.6 | promoter-TSS (NM_001164347) | -53 | ZBTB20 |
| chr17-44 | chr17 | 4843502 | 4843652 | 60.2 | promoter-TSS (NM_001165418) | -53 | RNF167 |
| chr3-89 | chr3 | 49131481 | 49131631 | 40.2 | promoter-TSS (NM_017730) | -52 | QRICH1 |
| chr7-31 | chr7 | 130794732 | 130794882 | 73.6 | promoter-TSS (NM_001145354) | -48 | MKLN1 |
| chr7-19 | chr7 | 1062632 | 1062782 | 107.1 | promoter-TSS (NR_029898) | -45 | MIR339 |
| chr7-138 | chr7 | 99679340 | 99679490 | 33.5 | promoter-TSS (NM_032924) | -44 | ZNF3 |
| chr2-148 | chr2 | 677406 | 677556 | 40.2 | promoter-TSS (NM_152834) | -42 | TMEM18 |
| chr9-121 | chr9 | 116840717 | 116840867 | 33.5 | promoter-TSS (NM_001633) | -40 | AMBP |
| chr12-71 | chr12 | 49351215 | 49351365 | 46.8 | promoter-TSS (NM_001659) | -38 | ARF3 |
| chr20-69 | chr20 | 17949453 | 17949603 | 26.8 | promoter-TSS (NM_014426) | -38 | SNX5 |
| chr9-115 | chr9 | 98637789 | 98637939 | 26.8 | promoter-TSS (NM_001010895) | -36 | ERCC6L2 |
| chr17-7 | chr17 | 79881404 | 79881554 | 207.5 | promoter-TSS (NM_032711) | -35 | MAFG |
| chr8-78 | chr8 | 144911512 | 144911662 | 46.8 | promoter-TSS (NM_001136033) | -31 | PUF60 |
| chr13-23 | chr13 | 21750693 | 21750843 | 33.5 | promoter-TSS (NM_001166017) | -27 | SKA3 |
| chr1-100 | chr1 | 225616507 | 225616657 | 53.5 | promoter-TSS (NM_002296) | -25 | LBR |
| chr11-144 | chr11 | 77705667 | 77705817 | 33.5 | promoter-TSS (NM_033547) | -25 | INTS4 |
| chr10-106 | chr10 | 70091669 | 70091819 | 46.8 | promoter-TSS (NM_012207) | -24 | HNRNPH3 |
| chr5-82 | chr5 | 75919208 | 75919358 | 33.5 | promoter-TSS (NM_001256566) | -24 | F2RL2 |
| chr19-115 | chr19 | 39936090 | 39936240 | 40.2 | promoter-TSS (NM_001130824) | -21 | SUPT5H |
| chr14-53 | chr14 | 103800244 | 103800394 | 40.2 | promoter-TSS (NM_001969) | -20 | EIF5 |
| chr11-110 | chr11 | 72983155 | 72983305 | 33.5 | promoter-TSS (NM_004154) | -17 | P2RY6 |
| chr17-89 | chr17 | 33390700 | 33390850 | 46.8 | promoter-TSS (NM_001017368) | -16 | RFFL |
| chr1-221 | chr1 | 156252613 | 156252763 | 26.8 | promoter-TSS (NR_026678) | -16 | TMEM79 |
| chr2-212 | chr2 | 70520807 | 70520957 | 33.5 | promoter-TSS (NM_003096) | -13 | SNRPG |
| chr16-109 | chr16 | 57481250 | 57481400 | 33.5 | promoter-TSS (NM_020313) | -12 | COQ9 |
| chr7-134 | chr7 | 75677257 | 75677407 | 33.5 | promoter-TSS (NM_016086) | -11 | STYXL1 |
| chr14-72 | chr14 | 23504360 | 23504510 | 33.5 | promoter-TSS (NM_002797) | -6 | PSMB5 |
| chr12-172 | chr12 | 133263874 | 133264024 | 33.5 | promoter-TSS (NM_018663) | -4 | POLE |
| chr17-75 | chr17 | 53045992 | 53046142 | 46.8 | promoter-TSS (NM_001162861) | -3 | COX11 |
| chrX-56 | chrX | 70473966 | 70474116 | 33.5 | promoter-TSS (NM_201599) | -3 | ZMYM3 |
| chr11-121 | chr11 | 13484766 | 13484916 | 26.8 | promoter-TSS (NM_032320) | -3 | BTBD10 |
| chr17-121 | chr17 | 57784786 | 57784936 | 40.2 | promoter-TSS (NM_016077) | -2 | VMP1 |
| chr6-101 | chr6 | 44355174 | 44355324 | 33.5 | promoter-TSS (NM_001253) | -2 | CDC5L |
| chr1-25 | chr1 | 94374938 | 94375088 | 113.8 | promoter-TSS (NM_002061) | -1 | GCLM |
| chr19-47 | chr19 | 10613406 | 10613556 | 66.9 | promoter-TSS (NM_012289) | 0 | KEAP1 |
| chr2-1 | chr2 | 232325073 | 232325223 | 508.6 | promoter-TSS (NR_004398) | 5 | SNORD82 |
| chr15-88 | chr15 | 75182340 | 75182490 | 33.5 | promoter-TSS (NM_002435) | 5 | MPI |
| chr12-64 | chr12 | 57081997 | 57082147 | 60.2 | promoter-TSS (NM_006601) | 6 | PTGES3 |
| chr12-166 | chr12 | 118454437 | 118454587 | 33.5 | promoter-TSS (NM_007370) | 6 | RFC5 |
| chr9-42 | chr9 | 34458486 | 34458636 | 60.2 | promoter-TSS (NM_001184945) | 7 | FAM219A |
| chr17-42 | chr17 | 74733517 | 74733667 | 73.6 | promoter-TSS (NM_001195427) | 9 | MFSD11 |
| chr11-142 | chr11 | 72145637 | 72145787 | 33.5 | promoter-TSS (NM_001258392) | 12 | CLPB |
| chr11-122 | chr11 | 31014140 | 31014290 | 33.5 | promoter-TSS (NM_020869) | 18 | DCDC5 |
| chr13-28 | chr13 | 52733900 | 52734050 | 40.2 | promoter-TSS (NM_001146099) | 21 | NEK3 |
| chr1-46 | chr1 | 248684895 | 248685045 | 80.3 | promoter-TSS (NM_001013355) | 22 | OR2G6 |
| chr20-83 | chr20 | 52199539 | 52199689 | 33.5 | promoter-TSS (NM_006526) | 22 | ZNF217 |
| chr6-62 | chr6 | 84140835 | 84140985 | 46.8 | promoter-TSS (NM_002395) | 28 | ME1 |
| chr20-44 | chr20 | 34330151 | 34330301 | 40.2 | promoter-TSS (NR_040724) | 32 | RBM39 |
| chr8-8 | chr8 | 30585376 | 30585526 | 113.8 | promoter-TSS (NM_001195104) | 35 | GSR |
| chr20-79 | chr20 | 42839435 | 42839585 | 33.5 | promoter-TSS (NM_016470) | 36 | C20orf111 |
| chr20-13 | chr20 | 48909219 | 48909369 | 100.4 | promoter-TSS (NR_034124) | 37 | LOC284751 |
| chr14-45 | chr14 | 65409507 | 65409657 | 33.5 | promoter-TSS (NR_046321) | 41 | GPX2 |
| chr22-14 | chr22 | 18189391 | 18189541 | 73.6 | promoter-TSS (NM_001270733) | 42 | BCL2L13 |
| chr7-140 | chr7 | 107531553 | 107531703 | 26.8 | promoter-TSS (NM_000108) | 42 | DLD |
| chr1-191 | chr1 | 31440965 | 31441115 | 33.5 | promoter-TSS (NR_003066) | 44 | SNORD85 |
| chr3-40 | chr3 | 47422462 | 47422612 | 66.9 | promoter-TSS (NM_015466) | 46 | PTPN23 |
| chr9-75 | chr9 | 123605173 | 123605323 | 46.8 | promoter-TSS (NM_001270427) | 51 | PSMD5 |
| chr2-171 | chr2 | 120517186 | 120517336 | 33.5 | promoter-TSS (NM_002830) | 54 | PTPN4 |
| chr12-125 | chr12 | 124773690 | 124773840 | 40.2 | promoter-TSS (NM_181709) | 55 | FAM101A |
| chr19-4 | chr19 | 6375729 | 6375879 | 281.1 | promoter-TSS (NM_004158) | 56 | PSPN |
| chr19-8 | chr19 | 15852184 | 15852334 | 187.4 | promoter-TSS (NM_013938) | 56 | OR10H3 |
| chr11-16 | chr11 | 5067740 | 5067890 | 113.8 | promoter-TSS (NM_001001916) | 59 | OR52J3 |
| chr7-38 | chr7 | 72299937 | 72300087 | 73.6 | promoter-TSS (NR_001588) | 60 | SBDSP1 |
| chr10-76 | chr10 | 49609681 | 49609831 | 66.9 | promoter-TSS (NM_002750) | 69 | MAPK8 |
| chr15-25 | chr15 | 59903978 | 59904128 | 73.6 | promoter-TSS (NM_004751) | 71 | GCNT3 |
| chr1-22 | chr1 | 202183284 | 202183434 | 127.2 | promoter-TSS (NM_001017404) | 76 | LGR6 |
| chrX-60 | chrX | 100662848 | 100662998 | 33.5 | promoter-TSS (NM_000169) | 78 | GLA |
| chr1-77 | chr1 | 110546574 | 110546724 | 60.2 | promoter-TSS (NM_001242676) | 79 | AHCYL1 |
| chr7-4 | chr7 | 56019618 | 56019768 | 214.2 | promoter-TSS (NM_015969) | 82 | MRPS17 |
| chr16-120 | chr16 | 83841524 | 83841674 | 33.5 | promoter-TSS (NM_001537) | 91 | HSBP1 |
| chr3-112 | chr3 | 25831349 | 25831499 | 33.5 | promoter-TSS (NM_001145391) | 106 | NGLY1 |
| chr2-195 | chr2 | 25194781 | 25194931 | 33.5 | promoter-TSS (NR_034113) | 107 | DNAJC27 |
| chr3-33 | chr3 | 168864213 | 168864363 | 73.6 | promoter-TSS (NM_001105077) | 112 | MECOM |
| chr17-53 | chr17 | 1619867 | 1620017 | 60.2 | promoter-TSS (NR_028505) | 125 | WDR81 |
| chr1-155 | chr1 | 110881549 | 110881699 | 40.2 | promoter-TSS (NM_001201545) | 169 | LOC440600 |
| chr17-10 | chr17 | 19281193 | 19281343 | 147.2 | promoter-TSS (NM_002749) | 227 | B9D1 |
| chr1-49 | chr1 | 54518730 | 54518880 | 80.3 | promoter-TSS (NR_039942) | 306 | TMEM59 |
| chr10-195 | chr10 | 115613444 | 115613594 | 26.8 | promoter-TSS (NM_198514) | 340 | DCLRE1A |
| chr19-62 | chr19 | 12780867 | 12781017 | 53.5 | promoter-TSS (NM_016145) | 425 | WDR83 |
| chr1-215 | chr1 | 151032732 | 151032882 | 33.5 | promoter-TSS (NM_020239) | 656 | MLLT11 |
| chr17-14 | chr17 | 1620477 | 1620627 | 147.2 | promoter-TSS (NR_028505) | 735 | WDR81 |
| chr8-85 | chr8 | 48873426 | 48873576 | 40.2 | promoter-TSS (NM_001081640) | 738 | MCM4 |
