# Supplementary material for: Genome-wide global identification of NRF2 binding sites in A549 non-small cell lung cancer cells by ChIP-Seq reveals NRF2 regulation of genes involved in focal adhesion pathways
Source: Aging (Albany NY). 2019 Dec 28;11(24):12600–23. doi: 10.18632/aging.102590 (PMC6949066; doi:10.18632/aging.102590)
Supplement: Supplementary Table 4 [file aging-11-102590-s001..docx]

**Supplementary Table 4. Overlapping genes from integrated analysis of NRF2 TFBS and downregulated genes in the NRF2-knockdown microarray (FC>1.25).**

**253 common overlapping genes in TFBS and GSE28230**

| ABCB6 |
| --- |
| ABCC1 |
| ABCC2 |
| ABHD4 |
| ACTR3B |
| ADAMTS12 |
| ADD2 |
| AGPAT9 |
| AK5 |
| AKAP11 |
| AKR1B1 |
| AKR1B10 |
| AKR1C1 |
| AKR1C2 |
| AKR1C3 |
| ALDH3A1 |
| ALDH3A2 |
| ALDOA |
| AMBP |
| ANAPC1 |
| AQP11 |
| AR |
| ASAP1 |
| BAG2 |
| BEND6 |
| BLVRB |
| BTBD7 |
| BTD |
| C15orf39 |
| C5orf30 |
| CA12 |
| CAPRIN1 |
| CAT |
| CAV2 |
| CBS |
| CCDC42B |
| CCDC77 |
| CCND3 |
| CCNL2 |
| CDC42BPA |
| CDK5RAP2 |
| CDRT1 |
| CEBPA |
| CEBPZ |
| CEP85 |
| CES1 |
| CLN8 |
| CNBP |
| CNP |
| CPLX2 |
| CTGF |
| CYB5A |
| CYB5R4 |
| CYP4F11 |
| CYP4F2 |
| CYP4F3 |
| DCDC5 |
| DCLK2 |
| DIS3L |
| DLG1 |
| DLG5 |
| DNA2 |
| DOCK10 |
| DSCC1 |
| DST |
| DTD2 |
| EGLN3 |
| EIF2S3 |
| EMP2 |
| EP400 |
| EPHX1 |
| ERGIC2 |
| ERMP1 |
| F2RL2 |
| FAM129B |
| FAM160B1 |
| FAM69A |
| FAM83C |
| FANCM |
| FASN |
| FBXO30 |
| FECH |
| FER1L4 |
| FERMT1 |
| FLNB |
| FOPNL |
| FTH1 |
| FTL |
| FTMT |
| FZD7 |
| GCLC |
| GCLM |
| GCNT3 |
| GLA |
| GPC1 |
| GPX2 |
| GSR |
| GTF2A1 |
| HGD |
| HJURP |
| HMOX1 |
| HNRNPD |
| HRG |
| HSBP1 |
| HSPH1 |
| HTATIP2 |
| IL6ST |
| IMPA2 |
| INADL |
| ISCA1 |
| JAKMIP3 |
| KCNE4 |
| KEAP1 |
| KIAA1598 |
| KIF14 |
| KLHL21 |
| KPNB1 |
| LBR |
| LOC344887 |
| LOC399815 |
| LRP12 |
| LRP6 |
| LRP8 |
| LRRC28 |
| LRRC58 |
| LRRC8B |
| LRRC8D |
| MAFG |
| MAGEB2 |
| MAP2 |
| MAPK8 |
| MASTL |
| MBOAT4 |
| MCM10 |
| MDH2 |
| ME1 |
| MEGF9 |
| MEMO1 |
| METTL21A |
| MFI2 |
| MGST1 |
| MMS22L |
| MPP6 |
| MRO |
| MSN |
| NAMPT |
| NCAPD3 |
| NCF2 |
| NDUFAF4 |
| NEDD4 |
| NEIL3 |
| NGLY1 |
| NINJ2 |
| NLN |
| NPR3 |
| NPRL3 |
| NQO1 |
| NQO2 |
| NR0B1 |
| NRCAM |
| NSMAF |
| NUMBL |
| NUP153 |
| NXPE3 |
| OR10H3 |
| OSGIN1 |
| OSGIN2 |
| P2RY6 |
| PABPC3 |
| PDCL |
| PFKP |
| PFN2 |
| PGD |
| PIR |
| POLE |
| PPCDC |
| PPIF |
| PRDX1 |
| PSMB5 |
| PTDSS1 |
| PTGES3 |
| PTGR1 |
| PTH2R |
| PTPRD |
| PWP1 |
| PYCR1 |
| RAB10 |
| RAP1GAP |
| RBBP8 |
| RFC5 |
| RNF115 |
| RNF213 |
| RPS6KA5 |
| SEL1L3 |
| SFN |
| SFXN5 |
| SIK1 |
| SIX3 |
| SLC1A2 |
| SLC1A5 |
| SLC38A6 |
| SLC48A1 |
| SLC5A11 |
| SLC6A6 |
| SLC7A11 |
| SLITRK4 |
| SLTM |
| SNTG1 |
| SPIN4 |
| SPP1 |
| SPSB1 |
| SQSTM1 |
| SRBD1 |
| SRXN1 |
| SSB |
| SSH1 |
| ST8SIA2 |
| STOX2 |
| TALDO1 |
| TBC1D14 |
| TBC1D8 |
| TBXAS1 |
| TCFL5 |
| TKT |
| TLR6 |
| TM4SF20 |
| TMEM18 |
| TMTC3 |
| TNPO1 |
| TOB1 |
| TPD52L1 |
| TPI1 |
| TRIM16L |
| TRIM52 |
| TSKU |
| TXN |
| TXNRD1 |
| UBR4 |
| UCHL1 |
| UCN3 |
| UGDH |
| UGT1A6 |
| UNKL |
| UXS1 |
| VCP |
| VSIG1 |
| WASF3 |
| ZBTB41 |
| ZMYM3 |
| ZNF273 |
| ZNF280C |
| ZNF718 |
| ZSWIM6 |
